# Supplementary material for: Interventions that enhance health services for parents and infants to improve child development and social and emotional well-being in high-income countries: a systematic review
Source: BMJ Open. 2018 Feb 8;8(2):e014899. doi: 10.1136/bmjopen-2016-014899 (PMC5829600; doi:10.1136/bmjopen-2016-014899)
Supplement: Supplementary file 1 [file bmjopen-2016-014899supp001.pdf]

Web Appendices

A. Medline search strategy

B. List of programme and organisation websites and journals for electronic journal of contents (eTOC) search

C. Full narrative summary of results

D. Effect direction plots, stratified by other characteristics

Web Appendix A. Medline search strategy

Database: Ovid MEDLINE(R) without Revisions
Search Strategy:
----
1 exp Health Personnel/
2 exp Nurses, Community Health/
3 health professional.tw.
4 exp Physician-Patient Relations/
5 (nurse* or physician* or doctor* or midwife* or midwive* or health visitor*).tw.
6 ((health* or medical or nurs* or hospital) adj (personnel or provider* or professional* or practitioner* or worker* or staff or specialist*)).tw.
7 (general practitioner* or gp or gps or clinician* or hospitalist* or psychologist* or social worker* or welfare worker*).tw.
8 ("Mothers aide" or "mothers help" or "maternity nurse" or "public health nurs*" or "specialist public health nurse" or "nursery nurse" or nanny or "early year educator*" or childcarer).tw.
9 ((paediatric or pediatric) adj clinic*).tw.
10 ((baby or child) adj clinic*).tw.
11 Primary Health Care/
12 exp House Calls/
13 home visit*.tw.
14 day care.mp.
15 clinic visit.tw.
16 surgery visit.tw.
17 clinician-patient interaction.tw.
18 early intervention*.tw.
19 ((parent* or mother*1 or father*1 or mum or dad or family or families) adj (group or support or training or education)).tw.
20 ((carer* or caregiver) adj (group or support or training or education)).tw.
21 ("early years staff" or "early years health professional*" or "early years professional*" or "early years educator*" or "early years practitioner*").tw.
22 (preschool educator or nursery staff).tw
23 parent educator*.tw.
24 (Family support or family support worker or support worker).tw.
25 (Community health worker or lay health worker).tw
26 Public health staff.tw.
27 Breakthrough to Literacy.mp
28 Family Foundations.mp.
29 Incredible Years.mp.
30 Parent Effectiveness Training.mp.
31 Primary Care Triple P.mp.
32 Triple P.mp.
33 Born to Learn.mp.
34 Mellow Parenting.mp.
35 "The Family Partnership Model".mp.
36 The Solihull Approach.mp.
37 "Parent child interaction therapy".mp.
38 "Nurturing Programme".mp.
39 "The Family Partnership Model".mp.
40 "Peers Early Education Partnership".mp.
41 "Webster Stratton Incredible Years".mp.

42 "Community Mothers Program".mp.
43 "Social Support and Family Health".mp.
44 "Limerick Lullaby".mp.
45 or/1-44 (ENHANCED PROFESSIONAL CONTACT TERMS)
46 exp Child Development/
47 exp language development/
48 Object Attachment/
49 Concept Formation/
50 Child Behavior Disorders/
51 ((child or infant) adj development).tw.
52 ((education* or cognitive or mental or language or physical or motor or social or emotional) adj3 (progress or development)).tw.
53 ((physical or emotional or social) adj (wellbeing or health)).tw.
54 (faltering growth or growth faltering or stunting).tw.
55 early child development.tw.
56 ((vocabulary or speech or language) adj development).tw.
57 Child behavio?r.tw.
58 Language/
59 Object Attachment/
60 social competence.tw.
61 social interaction.tw.
62 school readiness.tw.
63 or/46-62 (CHILD DEVELOPMENT TERMS)
64 45 and 63

Web Appendix B. List of programme and organisation websites and journals for electronic journal of contents (eTOC) search

Table B1. List of relevant programmes and organisations for which websites were searched

**Programme name**
1. Al's Pals
2. Breakthrough to Literacy
3. Bright Beginnings
4. Circle of Security
5. Community Mothers Program
6. Curiosity Corner
7. Developing Everyone's Learning and Thinking Abilities (DELTA)
8. Eager and Able to Learn
9. Family Check-Up
10. Family Foundations
11. Family Literacy programme
12. Family Nurturing Network: Family Connections
13. Family Partnership Model
14. Fun and Families
15. HIPPY
16. Holding Hands
17. I Can Problem Solve
18. Incredible Years
19. Infant Behavioral Assessment and Intervention Programme
20. Let's Begin with the Letter People
21. Let's Play in Tandem
22. LifeStart ("Growing Child")
23. Limerick Lullaby
24. Living with Children
25. Mellow Parenting
26. Minding the Baby
27. Noughts to Sixes (From Pram to Primary School), Family Caring Trust
28. Nurturing Programme
29. Parents as First Teachers or Parents as Teachers (Born to Learn)
30. Parent–child interaction therapy
31. Parent Effectiveness Training
32. Parenting Positively
33. Parents Plus Early Years
34. Peers Early Education Partnership
35. Preparing for Life<br>Child Development Initiative
36. Ready, Set, Leap!
37. Ready, Steady, Grow
38. Rock-a- Bye
39. Sheffield Raising Early Achievement in Literacy (REAL) project
40. Solihull Approach

41. Social Support and Family Health
42. Strengthening Families, Strengthening Communities
43. Supporting Parents on Kids Education (SPOKES)
**Organisation name**
1. Basic Skills Agency
2. Department of Education, UK
3. Early Childhood Australia
4. Education Endowment Fund
5. Early Intervention Foundation
6. European Commission (Education and Training)
7. European Early Childhood Education Research Association
8. Harvard Developing Child Programme
9. National Health Service (NHS)
10. National Institute for Health and Care Excellence (NICE)
11. National Institute of Health (USA)
12. Raising Children Network (Australia)
13. Sure Start/Flying Start
14. World Bank Early Child Development
15. World Health Organisation

Table B2. Journals included in electronic table of contents (eTOC) search

Archives of Diseases in Childhood

Child: Care, Health and Development

Child Development

Early Child Development and Care

Early Childhood Research Quarterly

Infant Mental Health Journal

Journal of Early Childhood Research

Pediatrics

Web Appendix C. Full narrative summary of results

Table C1: Motor Development Outcomes

Motor development outcomes		
**Studies comparing one intervention with usual care**		
**Study**	**Measure**	**Results**
Chang 2015 (Short term post intervention)	Griffith Mental Development Scale	**No difference in mean hand/eye coordination score** between intervention and control (mean 95.95, SD 10.25 compared with mean 94.62, SD 9.89); adjusted mean difference 0.73, 95% CI -0.83, 2.29; p value for comparison = 0.89)
Cupples 2011 (Immediately post intervention)	Bayley Scales of Infant Development (Psychomotor Development Index)	**No difference in mean scores** between intervention and control for PDI (MD [non-imputed] 1.64, 95% CI -0.94-4.21, p=0.21)
Wagner 2001 (Long term whilst programme ongoing)	Development Profile II	**No difference in mean physical development scores** between intervention and control in total sample (mean in intervention 2.9, mean in control 3.0, p>=0.05), or in samples stratified by income level (p>=0.05 for all tests)
**Motor development outcomes**		
**Studies comparing two interventions**		
**Study**	**Measure**	**Results**
Beeghly 1995 (Short term post-intervention)	Bayley Scales of Infant Development (Psychomotor Development Index)	**No difference in mean scores** for PDI in infant-centred (mean 109.77, SD 13.05) and mother-centred (mean 108.31, SD 12.43) groups at 3 months post-intervention (when children were 4 months, p>=0.05).
Doyle 2011 (Short term whilst intervention is ongoing)	Ages and Stages Questionnaire	**No differences** between high and low intensity programme using mean scores or cut-offs for 4 comparisons: gross motor scores, gross motor cut-off, fine motor scores, fine motor cut-offs (p values adjusted for multiple testing)
Doyle 2011 (Medium term whilst intervention is ongoing)	Ages and Stages Questionnaire	**No differences** between high and low intensity programme using mean scores or cut-offs for 4 comparisons: gross motor scores, gross motor cut-off, fine motor scores, fine motor cut-offs (p values adjusted for multiple testing)
Doyle 2011 (Long term whilst intervention is ongoing)	Ages and Stages Questionnaire (measured at 18, 24 and 36 months)	**No differences** between high and low intensity programme using mean scores or cut-offs for 4 comparisons at each time point: gross motor scores, gross motor cut-off, fine motor scores, fine motor cut-offs at any of the time points (p values adjusted for multiple testing)
Lobo 2012 (Short term whilst intervention ongoing)	Alberta Infant Motor Scale	**No difference for change in total mean scores** between in handling group compared with social experience group at 2-3 months (scores not given, p>=0.05).<br>**Greater change in mean score** for **prone** subscale in handling group compared with social experience group at 2-3 months of age (Cohen's correlation coefficient **r = -0.45, p<0.01**).<br>**No difference** for **supine** and **sitting** subscale at 2-3 months (scores not given, p>=0.05).<br>**Greater change in total mean score** in handling group compared with social experience group at 5 months of age (Cohen's correlation coefficient r = -0.46, p<0.01).<br>**Greater change in total mean score** for **prone** (r=-0.45, p<=0.01) and **sitting** (r=-0.34, p<=0.05) subscale in

		handling group at 5 months of age.<br>**No difference** for **supine** subscale at 5 months of age (scores not given, p>=0.05).
Lobo 2012<br>(Short term post-intevention)	Parent Milestone Report Form	Intervention group children reached for midlife objects before control children (no data provided, p<=0.05)<br>Intervention group children transferred objects from one hand to another **2.5 weeks earlier** (p<=0.05)<br>Intervention group children crept/crawled **5 weeks earlier** (p<=0.05)<br>Intervention group children walked supported **2.5 weeks earlier** (p<=0.05)<br>Intervention group children walked alone **6 weeks earlier** (p<=0.05)

Table C2: Language Development Outcomes

Language development outcomes		
**Studies comparing one intervention with usual care**		
**Study**	**Measure**	**Results**
Chang 2015 (Short term post intervention)	Griffith Mental Development Scale<br><br>MacArthur Bates Communicative Development Inventory (Short version, CDI)	**No difference in mean language scores** (Griffith scale) between intervention and control (mean 99.78, SD 14.01 compared with mean 99.90, SD 13.58); adjusted mean difference -0.54, 95% CI -2.81, 1.75; p value for comparison = 0.89)<br>**No difference in mean vocabulary scores (CDI)** between intervention and control (mean 38.00, SD 18.42 compared with mean 39.57, SD 20.49); adjusted mean difference -0.94, 95% CI -3.49, 1.61; p value for comparison = 0.89)
Christakis 2007 (Short term post intervention)	MacArthur Communicative Development Inventory	Whole sample: **No difference** between intervention and control (linear regression results: β for intervention variable raw score = 7.52, 95% CI -0.66-15.7; β for intervention variable percentile score = 8.40, 95% CI -3.00-19.90) at approx 4 months post intervention (children were 24 to 36 months).<br>Low and middle income sample: **Higher scores** in the intervention group (linear regression results: β for intervention variable raw score = 12.40, 95% CI 3.0-21.8, p=0.01; β for intervention variable percentile score = 14.94, 95% CI 1.37-27.60, p=0.03) at approx 4 months post intervention (children were 24 to 36 months).<br>High income sample: Results not presented in paper.
Drotar 2008 (Long term whilst intervention ongoing)	Systematic Analysis of Language Transcripts (SALT)	**No difference in mean scores** between intervention and control when children were 36 months (MD -3.91, 95% CI -14.32-6.77, p=0.48).
High 2000 (Short term post-intervention)	Modified MacArthur Communicative Development Inventory (short form)	**Higher mean scores** in intervention group for receptive vocabulary in total sample (p values between 0.008 and 0.003 in 3 tests), but no difference on expressive vocabulary (3 tests)<br>**No difference** in mean scores in receptive and expressive vocabulary between intervention and control groups in children aged 13-17 months at assessment (6 sub-group tests).<br>**Higher mean scores** in intervention group for receptive and expressive vocabulary in children aged 18-25 months at assessment (**p values between 0.01 and 0.003 in 6 sub-group tests**)
Tsiantis 2000 (Immediately post-intervention)	Bzoch-league Receptive Expressive Emergent Language Scale (REEL)	**Better performance in observed language** in comparison group children (no scores presented, but **p<0.0001**)<br>Results analysed by country, but not presented in paper.
Wagner 2002 (Long term whilst intervention is ongoing)	Development Profile II (DPII)	**No difference in mean scores** between intervention and control in communication development scores in whole sample (mean in intervention 4.9, mean in control 4.5, p>=0.05), or when stratified by income group.
Wiggins 2004 (Short term post-intervention)	Question on whether the mother has any worries about their child's speech	**Fewer mothers in the SHV group** expressed a worry about their child's speech than in the control group (risk ratio 0.46, 95% CI 0.23, 0.93)<br>**No difference** in the number of number of mothers expressing worries about speech between CGS and control (risk ratio 1.22, 95% CI 0.78, 1.92)

Language development outcomes		
**Studies comparing two interventions**		
**Study**	**Measure**	**Results**
Doyle 2011<br>(Short term whilst intervention is ongoing)	Ages and Stages Questionnaire	**No differences** between high and low intensity programme using mean scores or cut-offs for 2 comparisons: communication scores, communication cut-off (p values adjusted for multiple testing)
Doyle 2011<br>(Medium term whilst intervention is ongoing)	Ages and Stages Questionnaire<br><br>MacArthur-Bates Communicative Development Inventory	**No differences** between high and low intensity programme using mean scores or cut-offs for 2 comparisons: communication scores, communication cut-off (p values adjusted for multiple testing).<br>**No differences** between high and low intensity programme using mean scores for 4 sub-scores: first signs of understanding, words understood, words produced, first communicative gestures (p values adjusted for multiple testing)
Doyle 2011<br>(Long term whilst intervention is ongoing)	Ages and Stages Questionnaire (measured at 18, 24 and 36 months)<br>MacArthur-Bates Communicative Development Inventory (measured at 18 and 24 months)	**No differences** between high and low intensity programme using mean scores or cut-offs for 2 comparisons at any of the time points: communication scores, communication cut-off (p values adjusted for multiple testing).<br>**No differences** between high and low intensity programme using mean scores for 5 sub-scores at 18 months: first signs of understanding, can combine words, first communicative gestures, words produced, words understood, and 2 sub-scores at 24 months: can combine words, words produced (p values adjusted for multiple testing)
Johnston 2006<br>(Long term post-intervention)	Sub-scales of MacArthur Communicative Development Inventory	**Higher mean vocabulary score** in HS + PP group than HS only group at 24 months (aMD 4.08; 95% CI 0.69, 7.47; $p<0.05$).<br>**Higher percentage** combining >=2 words in HS + PP group than HS only group (rate ratio 1.05; 95% CI 1.02, 1.08; $p<0.05$).<br>**No difference** in length of child's longest phrase or variety of word endings between HS+PP and HS only group.
Landry 2006<br>(Short term post-intervention)	Coding of videotaped interactions between child and mother, and child with examiner	**Higher word usage scores** observed in the PALS group than the DAS group in interactions with mother ($p=0.02$) and examiner ($p=0.02$) at 3 months post-intervention when child was 13 months.

Table C3: Cognitive Development Outcomes

Cognitive development outcomes		
**Studies comparing one intervention with usual care**		
**Study**	**Measure**	**Results**
Chang 2015 (Short term post intervention)	Griffith Mental Development Scale	No effect in intervention group in unadjusted results (mean 92.69, SD 11.65 compared with mean 89.52, SD 10.56); unadjusted mean difference 3.12, 95% CI 0.86, 5.39; p value for comparison = 0.07); **higher mean cognitive scores (Griffith scale)** for adjusted mean difference (aMD 3.09, 95% CI 1.31, 4.87, p value = 0.007)
Cupples 2011 (Immedlately post-intervention)	Bayley Scales of Infant Development (Mental Development Index, MDI)	**No difference in mean scores** between intervention and control when children were 12 months old (MD [non-imputed] -0.81, 95% CI -2.81-1.16, p=0.42)
Drotar 2008 (Short-medium term whilst intervention ongoing)	Bayley Scales of Infant Development (Mental Development Index, MDI)<br>Assessor rating of mastery motivation (persistence, pleasure and competence with a play task)	**No difference in mean scores** between intervention and control when children were 18 months (MD 0.68, 95% CI -1.15-2.76, p=0.42).<br>**No difference in mean scores** between intervention and control for any of the 3 tasks when children were 18 months.
Drotar 2008 (Long term whilst intervention ongoing)	Bayley Scales of Infant Development (Mental Development Index, MDI)<br><br>Assessor rating of mastery motivation (persistence, pleasure and competence with a play task)<br><br><br><br><br>Kaufman Assessment Battery<br><br><br>Bracken Basic Concept Scale<br><br>Test of Early Reading Ability-2 (TERA-2)	**No difference in mean scores** between intervention and control for MDI (MD -0.78, 95% CI -3.63-2.32, p=0.67) when children were 24 months. **Higher mean score** in intervention than control in low SES sub-group (p for interaction < 0.003, mean for intervention 89.67 (SD16.20) & mean for control 82.16 (SD 17.24, p < 0.01).<br>**No difference in mean scores** between intervention and control for any of the 3 tasks when children were 24 months. **Higher mean score** for task competence in intervention group for low SES sub-group (p for interaction not shown, mean for intervention 584.49 (SD 37.97) & mean for control 556.21 (SD 57.18), p < 0.02.<br>**No difference** between intervention and control in 2 of 3 tests of mastery motivation (persistence and pleasure) when children were 36 months; **higher mean scores** in intervention group for task competence (MD 6.24, 95% CI 0.16-14.76, p=0.05).<br>**No difference in mean scores** between intervention and control for sequential processing, simultaneous processing or mental processing when children were 36 months (e.g. for mental processing MD 0.23, 95% CI -3.33-2.91, p=0.89).<br>**No difference in mean scores** between intervention and control when children were 36 months (MD 1.24, 95% CI -2.24-4.47, p=0.51).<br>**No difference in mean scores** between intervention and control when children were 36 months (MD 0.54, 95% CI -1.70-2.47, p=0.72).
Miller 2015 (Long term whilst intervention ongoing)	British Ability Scale (BASII)	**No difference in mean scores** between intervention and control on cognitive development (mean in intervention group -0.046 (SD 1.007) and mean in control group 0.018 (SD 0.994), Hedges g effect size -0.634, 95% CI -0.277, 0.149, p=0.557)
Wagner 2001 (Long term whilst intervention ongoing)	Development Profile II (DPII)	**No difference in mean scores** between intervention and control in cognitive development scores (mean in intervention 2.2, mean in control 2.4, p>=0.05), or in samples stratified by income.

Cognitive development outcomes		
**Studies comparing two interventions**		
**Study**	**Measure**	**Results**
Beeghly 1995 (Short term post intervention)	Bayley Scales of Infant Development (Mental Development Index, MDI)	**No difference in mean scores** in infant-centred (mean 109.38, SD 12.88) or mother-centred (mean 107.83, SD 10.69) groups at 3 months post-intervention (when children were 4 months, p>=0.05).
Doyle 2011 (Short term whilst intervention is ongoing)	Ages and Stages Questionnaire	**No differences** between high and low intensity programme using mean scores or cut-offs for 2 comparisons: problem solving score, problem solving cut-off (p values adjusted for multiple testing)
Doyle 2011 (Medium term whilst intervention is ongoing)	Ages and Stages Questionnaire<br><br>Development Profile 3	**No differences** between high and low intensity programme using mean scores or cut-offs (2 comparisons): problem solving score, problem solving cut-off (p values adjusted for multiple testing).<br>**No difference** in mean scores or for the proportion above the average cut-off (2 comparisons, p values not adjusted for multiple testing).
Doyle 2011 (Long term whilst intervention is ongoing)	Ages and Stages Questionnaire (measured at 18, 24 and 36 months)<br>Development Profile 3 (measured at 18, 24 and 36 months)	**No differences** between high and low intensity programme using mean scores or cut-offs for 2 comparisons at any of the time points: problem solving score, problem solving cut-off (p values adjusted for multiple testing).<br>At 18 months, **no difference** in mean scores or for the proportion above the average cut-off (p values not adjusted for multiple testing).<br>At 24 months, **no difference for the proportion above the average cut-off** (p values **not** adjusted for multiple testing); **higher mean standardised score** in high intensity group (mean 116.51 [SD 13.30] vs mean 112.57 [S 16.12], p<0.05, **not** adjusted for multiple testing).<br>At 36 months, **higher mean standardised score** in high intensity group (mean 114.66 [SD 14.39] vs mean 109.82 [SD 14.02], p<0.05, **not** adjusted for multiple testing) and **higher proportion above average cut-off** (0.53 [SD0.50] vs 0.36 [SD 0.48], p<0.05, **not** adjusted for multiple testing)
Landry 2006 (Short term post-intervention)	Coding of videotaped interactions between child and mother, and child with examiner	**Higher scores** for independent goal-directed play in the PALS group than in the DAS group (p=0.04) at 3 months post-intervention when child was 13 months.

Table C4: Social and Emotional Wellbeing Outcomes

Social and emotional wellbeing outcomes		
**Studies comparing one intervention with usual care**		
**Study**	**Measure**	**Results**
Cheng 2007 (Short term post-intervention)	Parent-Infant Relationship Global Assessment Scale	Of 37 mother-infant pairs with a "disturbed" relationship at baseline, a **higher percentage** of intervention group was classified as having an "adapted" relationship (63% [12 pairs] vs 28% [5 pairs], p=0.039) at 1 month post-intervention (when children were 10 months).<br>Of 48 mother-infant pairs with an "adapted" relationship at baseline, **no difference in percentage** of intervention vs control classified as "disturbed" (26% [7 pairs] vs 29% [8 pairs], p=0.821) at 1 month post-intervention (when children were 10 months).
Cheng 2007 (Long term post-intervention)	Child Behaviour Checklist	**No difference** between intervention and control in the odds of being in the upper quartile of the CBCL scores (total score aOR 0.40, 95% CI 0.12-1.43; internalizing score aOR0.60, 95% CI 0.23-1.68; externalizing score aOR 0.50, 95% CI 0.19-1.54) at 15 months post-intervention (when children were 24 months).
Christakis 2007 (Short term post-intervention)	Hyperactivity sub-domain of the Child Behavior Checklist	**No difference** in attention problems (score in 90th percentile or higher) between intervention and control in the whole sample (aOR 0.49, 95% CI 0.13-1.81) or low and middle income sample (aOR 0.48, 95% CI 0.12, 1.83) at approx 4 months post intervention (children were 24 to 36 months).
Cupples 2011 (Immediately post-intervention)	Bayley Scales of Infant Development (BSID-II)	**No difference in the mean scores** between intervention and comparison group (MD [non-imputed] -0.88, 95% CI -4.59-2.81, p=0.64)<br>**No difference in the mean scores** between intervention and control for the 3 sub-scores of the BSID-II behavioural scale.
Drotar 2008 (Short-medium term whilst intervention ongoing)	Bayley Behaviour Rating Scale	**No difference in mean scores** between intervention and control when children were 12 months (MD -0.33, 95% CI -1.95-1.64, p=0.87).
Drotar 2008 (Medium-long term whilst intervention ongoing)	Q-Sort measure of Security of Attachment	**No difference** between intervention and control when children were 18 months (no numerical data presented).
Drotar 2008 (Long term whilst intervention ongoing)	Bayley Behaviour Rating Scale<br><br>Child Behaviour Rating Scale<br>Social Skills Rating System	**No difference in mean scores** between intervention and control when children were 24 months (MD 0.43, 95% CI -1.72-3.15, p=0.56) or 36 months (MD -1.64, 95% CI -3.99-0.83, p=0.20).<br>**No difference** between intervention and control when children were 24 months (no numerical data presented).<br>**No difference in mean scores** between intervention and control when children were 36 months for parent. reported social skills (MD 3.33, 95% CI -0.45-5.90, p=0.09) or teacher reported social skills (MD 2.07, 95% CI -3.19-7.15, p=0.45).
Feinberg 2008 (Immediately post-intervention)	Infant Behaviour Questionnaire	**No difference** in parent-child dysfunctional interaction (beta calculated using linear regression) in intervention versus control group according to mother's report (beta -0.137, Cohen's d 0.34, p<0.10), but **intervention fathers had lower dysfunctional interaction scores** (beta = -0.291, Cohen's d = 0.70, p<0.05).<br>**No difference** in infant soothability score (beta calculated using linear regression) in intervention versus control

		group according to mother's report (beta 0.021, Cohen's d not calculated, p>=0.05), but **intervention children were easier to soothe** (had a higher score than control) according to father's reports (beta = 0.312, Cohen's d = 0.35, p<0.05).<br>Score for duration of attention **higher in intervention versus control infants** according to mother's report (F=4.33, Cohen's d = 0.34, p<0.05). Fathers reports of this variable not given in paper.
Feinberg 2008 (Short term post-intervention)	Observer coding of videotaped behaviours (self-soothing and sustained attention at play)	**More self-soothing behaviours** observed in intervention versus control group when infants were 12 months of age (6 months post-intervention, beta = 0.30, Cohen's d = 0.46, p<0.05).<br>**No difference** in sustained attention noted by coders between intervention and control children (beta = 0.05, Cohen's d = 0.08, p>=0.05).
Feinberg 2009 (Short term post-intervention)	Child Behaviour Checklist	Mean scores and SDs for each of the 7 comparisons presented but no accompanying P values; t tests conducted in Stata (assuming 81% follow-up in each group) indicate **no significant differences**.<br>Written results suggest that the model based estimates of intervention effects were significant but only when an interaction with child gender was included in the model - the presentation of these results is unclear and there appears to be an inconsistency between the results presented in the table and those in the text.
Griffith 2011 (Short term post-intervention)	Dyadic Parent-Child Interaction coding system	**No difference in mean scores** between intervention and control for child deviance behaviours in the parent-child interaction observations (negative physical behaviours, destructive behaviours, small talk; mean in intervention 3.18 (SD 3.40) and control 2.97 (SD 3.43), no p value for this comparison) at 3 months or less post-intervention.
Hiscock 2008 (Short term post-intervention)	Child Behaviour Checklist	**No difference in mean scores** between intervention and control for externalising (aMD 0.16, 95% CI -1.01-1.33, p=0.79) or internalising (aMD 0.49, 95% CI -0.20-1.18, p=0.16) behaviours 3 months post-intervention (when children were 18 months).
Hiscock 2008 (Medium term post-intervention)	Child Behaviour Checklist	**No difference in mean scores** between intervention and control for externalising (aMD -0.79, 95% CI -2.27-0.69, p=0.30) or internalising (aMD 0.19, 95% CI -0.77-1.15, p=0.70) behaviours 9 months post-intervention (when children were 24 months).
Hiscock 2008 (Long term post-intervention)	Child Behaviour Checklist	**No difference in mean scores** between intervention and control for externalising (aMD -0.8, 95% CI -2.2-0.6, p=0.26) or internalising (aMD -0.6, 95% CI -1.5-0.3, p=0.19) behaviours 21 months post-intervention (when children were 36 months).
Miller 2015 (Long term whilst intervention is ongoing)	Bayley Social-Emotional and Adaptive Behaviour Questionnaire	**No difference in mean scores** between intervention and control on socio-emotional development (mean in intervention group 0.087 (SD 0.987) and mean in control group -0.108 (SD 1.016, Hedges g effect size 0.195 (95% CI -0.046, 0.437, p=0.114)
Minkovitz 2003 (Long term whilst intervention is ongoing)	Subscales of Child Behaviour Checklist	**No difference in mean scores** for 3 sub-scores (aggressive behaviour, anxious or depressed, sleep problems) between intervention and control group. For example, for aggressive behaviour, the adjusted mean difference between intervention and control was 0.23 (95% CI -0.29, 0.79, p=0.16, higher scores indicate higher reporting of aggressive behaviour)
Niccols 2008 (Immediately post-intervention)	Attachment Q-set (completed by observers)	**No difference in the change in scores between pre-test and post-test** between intervention and comparison group in the intention-to-treat analysis (no numbers presented, text states "no significant differences")
Niccols 2008 (Short term post-intervention)	Attachment Q-set (completed by observers)	**No difference in the change in scores between pre-test and post-test** between intervention and comparison group in the intention-to-treat analysis 6 months post-intervention (no numbers presented, text states "no significant differences")

Niccols 2009 (Immediately post-intervention)	Eyberg Child Behavior Inventory<br><br>Observed parent-child interaction	**No difference in mean scores** between intervention and control for child behaviour problems (mean 48.87 (SD 6.22) vs mean score 50.68 (SD 7.23), p>=0.05)<br>**Higher mean number of observed** positive child behaviours in intervention group (mean 21.10 (SD 8.59) vs mean score 16.27 (SD 9.25), p<0.05), and **higher ratio** of child compliance to maternal requests in intervention group (mean 0.62 (SD 0.19) vs mean score 0.53 (SD 0.19), p<0.05).<br>**No difference in mean number** of observed negative behaviours in intervention versus control (mean 17.81 (SD 8.84) vs mean score 22.29 (SD 8.85), p>=0.05).
Niccols 2009 (Short term post-intervention)	Eyberg Child Behavior Inventory<br><br>Observed parent-child interaction	**Lower mean score** for child behaviour problems in intervention versus control (mean 48.87 (SD 6.22) vs mean score 50.68 (SD 7.23), p<0.017).<br>**Higher mean number** of observed positive child behaviours in intervention group than control (mean 22.61 (SD 8.97) vs mean score 16.69 (SD 8.40), p<0.05), and **higher ratio of** observed compliance to maternal requests in intervention group (mean 0.61 (SD 0.19) vs mean score 0.53 (SD 0.19), p<0.05).<br>**No difference** in mean number of observed negative behaviours between intervention and control (mean 18.86 (SD 7.53) vs mean score 21.29 (SD 7.78), p>=0.05)
Santelices 2010 (Immediately post-intervention)	Observed Strange Situation Procedure	**No difference** between intervention and control in infant attachment security (% classed as "secure" in intervention 72.1%, % classed as "secure" in control 55.2%, p=0.139)
Tsiantis 2000 (Short term whilst intervention is ongoing)	Bayley Scale of Psychosocial Development<br>Observed mother-child interaction	**No difference in mean scores** between intervention and control (mean 7.03 compared with mean 7.04, no numbers in analysis or statistics presented).<br>**Mother-child interaction rated as better** in intervention group in one of the four countries (no numbers in analysis, or scores presented, p for sub-group effect= 0.02).
Tsiantis 2000 (Medium term whilst intervention is ongoing)	Bayley Scale of Psychosocial Development<br>Observed mother-child interaction	**No difference in mean scores** between intervention and control (mean 6.82 compared with mean 7.06, no numbers in analysis or statistics presented)<br>**Mother-child interaction rated as better in comparison group** when all countries' data combined (no numbers in analysis presented, scores presented but not clear what they represent, p = 0.02)
Tsiantis 2000 (Long term whilst intervention is ongoing)	Bates Infant Characteristics Questionnaire	**No difference in total mean scores** in full sample between intervention and control (no numbers in analysis, scores or statistics presented for mean scores); 2 of 7 individual characteristic scores higher in intervention than control in full sample ("sober" p = 0.02, "factor 7" p <0.06).
Wagner 2001 (Long term whilst intervention is ongoing)	Nursing Child Assessment Satellite Training teaching scale (NCAST) used to observe mother-child interactions<br>Measure = Development Profile II (DPII)<br><br>Adaptive Social Behaviour Inventory	**No difference in mean scores** for observed mother-child interaction between intervention and control in total sample (2 comparisons), or when results stratified by income group (p>=0.05)<br><br>**No difference in mean self help or social development scores** between intervention and control in total sample, or when results stratified by income group (p>=0.05)<br>**No difference in mean scores** between intervention and control in total sample, or when results stratified by income group (p>=0.05)
Wiggins 2004 (Short term post-intervention)	Question on whether the mother has any worries about their child's behaviour	**No difference** in the number of number of mothers expressing worries about behaviour between supportive health visiting and control groups (risk ratio 0.74, 95% CI 0.37, 1.47)<br>**No difference** in the number of number of mothers expressing worries about behaviour between community group support and control groups (risk ratio 0.94, 95% CI 0.51, 1.73)

Social and emotional wellbeing outcomes		
**Studies comparing two interventions**		
**Study**	**Measure**	**Results**
Doyle 2011 (Short term whilst intervention is ongoing)	Ages and Stages Questionnaire<br><br>Child Characteristic Questionnaire	**No differences** between high and low intensity programme using mean scores or cut-offs for 4 comparisons: personal social score, personal social cut-off, social-emotional score, social-emotional cut-off (p values adjusted for multiple testing).<br>**No difference** between high and low intensity programme for score based on a 6 point scale of difficult temperament (p value adjusted for multiple testing).
Doyle 2011 (Medium term whilst intervention is ongoing)	Ages and Stages Questionnaire<br><br>Brief Child Toddler Social and Emotional Assessment<br>Temperament and Atypical Behaviour Score<br><br>Child Characteristic Questionnaire	**No differences** between high and low intensity programme using mean scores or cut-offs for 4 comparisons: personal social score, personal social cut-off, social-emotional score, social-emotional cut-off (p values adjusted for multiple testing)<br>**No differences** between high and low intensity programme using mean scores or cut-offs for 4 comparisons: competence score, competence cut-off, problem score, problem cut-off (p values adjusted for multiple testing)<br>**No differences** between high and low intensity programme using mean scores or binary variable (p values **not** adjusted for multiple testing)<br>**No difference** between high and low intensity programme for score based on a 6 point scale of difficult temperament (p value **not** adjusted for multiple testing)
Doyle 2011 (Long term whilst intervention is ongoing)	Ages and Stages Questionnaire (measured at 18, 24 and 36 months)<br><br>Brief Child Toddler Social and Emotional Assessment (measured at 18, 24 and 36 months)<br>Brief Child Toddler Social and Emotional Assessment subdomains (measured at 24 and 36 months)<br>Child Behaviour Checklist (CBCL, measured at 24 and 36 months)<br><br>Child Behaviour Checklist subdomains (CBCL, measured at 24 and 36 months)	**No differences** between high and low intensity programme using mean scores or cut-offs for 4 comparisons: personal social score, personal social cut-off, social-emotional score, social-emotional cut-off (p values adjusted for multiple testing)<br>**No differences** between high and low intensity programme using mean scores or cut-offs for 4 comparisons at 18 and 24 months: competence score, competence cut-off, problem score, problem cut-off, and for 2 comparisons at 36 months: competence score, problem score (p values adjusted for multiple testing)<br>**No differences in mean scores** between high and low intensity programme for 5 comparisons at 24 or 36 months: dysregulation, internal problems, red flag, external problems, autism (p value adjusted for multiple testing).<br>At 24 months, **no difference** between high and low intensity for 5 comparisons (CBCL total score; internalising problems score and cut-off, externalising problems score and cut-off); **higher proportion in the low intensity group above cut-off for total CBCL score** (0.00 [SD 0.00] vs 0.07 [0.26], p<0.01 adjusted for multiple testing)<br>At 36 months, **no difference** between high and low intensity for 6 comparisons (CBCL total score and cut-off; internalising problems score and cut-off, externalising problems score and cut-off)<br>**No difference** in scores between high and low intensity programmes for 8 comparisons at 24 or 36 months: sleep problems, other problems, emotionally reactive, attention problems, anxious/depressed, somatic complaints, aggressive, withdrawn (p values adjusted for multiple testing)
Johnston 2006 (Long term whilst intervention is ongoing)	Subscales of Child Behaviour Checklist	**Higher mean score** in HS+PP group than HS only group for anxiety or depression at 36 months (aMD 0.40; 95% CI 0.07, 0.73; p<0.05).<br>**No difference** in aggressive behaviour between HS+PP group and HS only group.

Landry 2006<br>(Short term post-intervention)	Coding of videotaped interactions between child and mother, and child with examiner	**No difference** in mean scores for cooperation with mothers (p=0.08) or a novel adults (results not presented).<br>Infants in PALS demonstrated "**significantly greater increase in their use of words**" when interacting with mothers (p=0.02) and a novel adult (p=0.02).<br>**No effect** on affect in interactions with mothers overall (results not presented); **lower negative affect** seen in infants in the PALS group when interacting with a novel adult (results not presented).<br>Also present some results for the interaction with birthweight but presentation of results incomplete (text suggests more positive effects of the PALS intervention in very low birthweight group)

Table C5: Overall Development Outcomes

**Overall development outcomes**		
**Studies comparing one intervention with usual care**		
**Study**	**Measure**	**Results**
Chang 2015 (Short term post intervention)	Griffith Mental Development Scale	**No difference in mean developmental quotient (Griffith)** between intervention and control (mean 96.14, SD 9.40 compared with mean 94.68, SD 8.25); adjusted mean difference 1.10, 95% CI -0.45, 2.65; p value for comparison = 0.72)
Griffith 2011 (Short term post-intervention)	Schedule of Growing Skills II (SGS II)	**No difference in mean scores** between intervention (mean 99.20, SD 18.27) and control (mean 102.38, SD 17.79) at around 3 months post-intervention (p=0.51)
Wiggins 2004 (Immediately post-intervention)	Question on mother's perception of whether her child's development was normal	**No difference** between "supportive home visiting" and control (risk ratio 0.88, 95% CI 0.39-1.99) or "community groups" and control (risk ratio 0.57, 95% CI 0.22-1.52) in mother's perception of whether her child's development was normal
Wiggins 2004 (Short term post-intervention)	Questions on mother's worries about different aspects of development	Comparison on general development not possible between SHV and control as no mothers expressed concerns in the SHV group.<br>**No difference** in the number of number of mothers expressing worries about general development between CGS and control (risk ratio 0.75, 95% CI 0.15, 1.92)<br>Mothers in the SHV group had **fewer mean number of worries** about their child's development than in the control group (MD -0.23, 95% CI -0.42 to -0.01)<br>**No difference in the mean number of worries** about their child's development between CGS and control (MD 0.13, 95% CI -0.10 to 0.36)
**Overall development outcomes**		
**Studies comparing one intervention with usual care**		
**Study**	**Measure**	**Results**
Doyle 2011 (Short term whilst intervention is ongoing)	Ages and Stages Questionnaire	**No difference in overall mean score** between high and low intensity programme (p value **not** adjusted for multiple testing) at 6 months
Doyle 2011 (Medium term whilst intervention is ongoing)	Ages and Stages Questionnaire	**No difference in overall mean score** between high and low intensity programme (p value **not** adjusted for multiple testing) at 12 months
Doyle 2011 (Long term whilst intervention is ongoing)	Ages and Stages Questionnaire (measured at 18, 24 and 36 months)	**No difference in overall mean score** between high and low intensity programme (p value **not** adjusted for multiple testing) at any of the time points

D. Effect direction plot, stratified by other characteristics
D1: Results stratified by age of children at intervention delivery

Study<br>Intervention intensity	Study design	Risk of bias	Adherence	Outcome measurement		Development outcomes					Additional detail on intervention effects or sub-group analyses
				Timing	When?	Motor	Lang	Cogn	SEWB	Overall	
**Studies comparing one intervention with usual care**											
**Intervention during antenatal and/or first year postpartum only**											
**Beeghly 1995**<br>2 low intensity	iRCT	Unclear	Not reported	Post	Short	○		○			Tested for interaction between intervention and parity, IUGR, "demographic" risk and maternal psychological risk; no significant interactions found.
**Cheng 2007**<br>Moderate	iRCT	Low	Not reported	Post<br>Post	Short<br>Long				 ○		For the short-term analysis, only sub-group analyses (by attachment quality) presented; results inconsistent.
**Cupples 2011**<br>High	iRCT	Low	Mean number of contacts = 8.5 (of 22 planned)	Post	Immed	○		○	○		
**Feinberg 2008**<br>Moderate	iRCT	Unclear	80% attended at least 3 of 4 antenatal sessions<br>60% attended at least 3 of 4 postnatal sessions	Post<br>Post<br>Post	Short<br>Medium<br>Long				○<br>○<br>○		3 of 5 comparisons in short term and 1 of 2 comparisons in medium term showed improved SEWB outcomes in intervention group.<br>Results presented in text suggest there may be interaction effects with gender, but there is incomplete reporting of the sub-group analyses.
**Lobo 2012**<br>2 moderate intensity	iRCT	Unclear	Excluded individuals who did not perform intervention on at least 60% of expected days	During<br>Post	Short<br>Short	○<br>●					4 of 8 comparisons showed improved motor outcomes in the intervention arm in the short-term whilst intervention was ongoing.<br>5 of 5 comparisons showed improved motor outcomes in the intervention arm post-intervention.
**Santelices 2011**<br>Moderate	iRCT	Unclear	Not reported	Post	Short				○		
**Wiggins 2004**<br>Moderate (SHV)	iRCT	Low	Mean number of visits = 7 (of 12 planned)	Post<br>Post	Immed<br>Short		 ●		 ○	○<br>●	1 of 1 comparison showed improved language outcome and 1 of 1 comparison showed improved overall development in intervention group.
**Wiggins 2004**<br>Low (CGS)	iRCT	Low	19% of women attended a group	Post<br>Post	Immed<br>Short		 ○		 ○	○<br>○	
**Intervention during first and second years postpartum**											
**Chang 2015**<br>Low	cRCT	Unclear	83% of mothers attended all visits	Post	Short	○	○	○		○	Improved cognitive outcome in intervention group on adjusting for potential confounders.
**Christakis 2007**<br>Low	iRCT	Unclear	Not reported	Post	Short		○		○		Sub-group results: 2 of 3 comparisons in low income group showed improved SEWB outcomes in intervention group; test for interaction not presented
**Doyle 2011**<br>1 high & 1 moderate	iRCT	Unclear	High: Mean number of visits = 46<br>Moderate: Not reported	During<br>During<br>During	Short<br>Medium<br>Long	○<br>○<br>○	○<br>○<br>○	○<br>○<br>○	○<br>○<br>○	○<br>○<br>○	3 of 12 comparisons showed improved cognitive outcomes, and 1 of 62 comparisons showed improved SEWB outcomes in the intervention arm, in the long-term whilst intervention was ongoing.

**Drotar 2008**<br>High	iRCT	Unclear	Not reported	During<br>During<br>During	Short<br>Medium<br>Long		 <br>○	 ○<br>○	○<br>○<br>○		1 of 12 comparisons showed improved SEWB outcomes in the intervention arm in the long-term. Incomplete reporting of the sub-group analyses.
**Griffith 2011**<br>High	iRCT	Low	60% attended 8 or more sessions (of 12 planned)	Post	Short				○	○	
**High 2000**<br>Low	iRCT	High	Mean number of visits = 3.4 (of 5 planned)	Post	Short		○				3 of 6 comparisons showed improved language outcomes in intervention group.<br>Sub-group results: no differences seen in 13-17 month olds; 6 of 6 comparisons in 18-25 month olds showed improved language outcomes in intervention group; no test for interaction presented.
**Hiscock 2008**<br>Low	cRCT	Low	49% of parents attended all sessions	Post<br>Post<br>Post	Short<br>Medium<br>Long				○<br>○<br>○		
**Johnston 2006**<br>2 high intensity	iRCT	Unclear	Not reported	During	Long		○		○		2 of 4 comparisons showed improved language outcomes, and 1 of 3 comparisons showed poorer SEWB outcomes, in the intervention arm in the long-term whilst intervention was ongoing.
**Landry 2006**<br>2 high intensity	iRCT	Unclear	91% of parents completed all 10 visits plus 2 assessment visits	Post	Short		●	●	○		2 of 2 comparisons showed improved language outcomes, and 1 of 1 comparison showed improved cognatic outcome in the intervention arm.<br>3 of 6 comparisons showed improved SEWB outcomes in the intervention arm.<br>Reporting of interaction with birthweight incomplete.
**Miller 2015**<br>High	iRCT	Low	Adherence data currently being analysed	During	Long			○	○		
**Minkovitz 2003**<br>High	iRCT	Unclear	79% of parents received 4 or more services (of 16)	During	Long				○		
**Niccols 2008**<br>Moderate	iRCT	Unclear	58% attended 4 or more sessions (of 8 planned)	Post<br>Post	Immed<br>Short				○<br>○		
**Niccols 2009**<br>Moderate	iRCT	Unclear	Not reported	Post<br>Post	Immed<br>Short				○<br>●		2 of 4 comparisons immediately post-intervention term showed improved SEWB outcomes in intervention group.<br>3 of 4 comparisons showed improved SEWB outcomes in intervention group in short-term.
**Tsiantis 2000**<br>Moderate	cRCT	Unclear	Not reported	Post<br>Post<br>Post	Short<br>Medium<br>Long		 <br>ø		○<br>○<br>○		2 of 2 comparisons showed poorer language outcomes in the intervention arm in the long-term.<br>1 of 2 comparisons showed poorer SEWB outcomes in the intervention arm in the medium-term.<br>1 of 8 comparisons showed improved SEWB outcomes in the intervention arm in the long-term.<br>Incomplete reporting of the sub-group analyses.
**Wagner 2001**<br>High	iRCT	Unclear	44% of families still receiving services at 2 yrs	During	Long	○	○	○	○		Results also stratified by income; no significant interactions reported.

D2: Results stratified by whether intervention delivery was delivered to all families or geographically targeted

Study<br>Intervention intensity	Study design	Risk of bias	Adherence	Outcome measurement		Development outcomes					Additional detail on intervention effects or sub-group analyses
				Timing	When?	Motor	Lang	Cogn	SEWB	Overall	
**Studies comparing one intervention with usual care**											
**Delivered to all families**											
**Beeghly 1995**<br>2 low intensity	iRCT	Unclear	Not reported	Post	Short	○		○			Tested for interaction between intervention and parity, IUGR, "demographic" risk and maternal psychological risk; no significant interactions found.
**Cheng 2007**<br>Moderate	iRCT	Low	Not reported	Post	Short						For the short-term analysis, only sub-group analyses (by attachment quality) presented; results inconsistent.
				Post	Long				○		
**Christakis 2007**<br>Low	iRCT	Unclear	Not reported	Post	Short		○		○		Sub-group results: 2 of 3 comparisons in low income group showed improved SEWB outcomes in intervention group; test for interaction not presented
**Drotar 2008**<br>High	iRCT	Unclear	Not reported	During	Short				○		1 of 12 comparisons showed improved SEWB outcomes in the intervention arm in the long-term. Incomplete reporting of the sub-group analyses.
				During	Medium			○	○		
				During	Long		○	○	○		
**Feinberg 2008**<br>Moderate	iRCT	Unclear	80% attended at least 3 of 4 antenatal sessions 60% attended at least 3 of 4 postnatal sessions	Post	Short				○		3 of 5 comparisons in short term and 1 of 2 comparisons in medium term showed improved SEWB outcomes in intervention group. Results presented in text suggest there may be interaction effects with gender, but there is incomplete reporting of the sub-group analyses.
				Post	Medium				○		
				Post	Long				○		
**Hiscock 2008**<br>Low	cRCT	Low	49% of parents attended all sessions	Post	Short				○		
				Post	Medium				○		
				Post	Long				○		
**Johnston 2006**<br>2 high intensity	iRCT	Unclear	Not reported	During	Long		○		○		2 of 4 comparisons showed improved language outcomes, and 1 of 3 comparisons showed poorer SEWB outcomes, in the intervention arm in the long-term whilst intervention was ongoing.
**Lobo 2012**<br>2 moderate intensity	iRCT	Unclear	Excluded individuals who did not perform intervention on at least 60% of expected days	During	Short	○					4 of 8 comparisons showed improved motor outcomes in the intervention arm in the short-term whilst intervention was ongoing.<br>5 of 5 comparisons showed improved motor outcomes in the intervention arm post-intervention.
				Post	Short	●					
**Miller 2015**<br>High	iRCT	Low	Adherence data currently being analysed	During	Long			○	○		
**Minkovitz 2003**<br>High	iRCT	Unclear	79% of parents received 4 or more services (of 16)	During	Long				○		
**Niccols 2008**	iRCT	Unclear	58% attended 4 or more	Post	Immed				○		

Moderate			sessions (of 8 planned)	Post	Short				○		
**Niccols 2009**<br>Moderate	iRCT	Unclear	Not reported	Post	Immed				○		2 of 4 comparisons immediately post-intervention term showed improved SEWB outcomes in intervention group.<br>3 of 4 comparisons showed improved SEWB outcomes in intervention group in short-term.
				Post	Short				●		
**Santelices 2011**<br>Moderate	iRCT	Unclear	Not reported	Post	Short				○		
**Tsiantis 2000**<br>Moderate	cRCT	Unclear	Not reported	Post	Short				○		2 of 2 comparisons showed poorer language outcomes in the intervention arm in the long-term.<br>1 of 2 comparisons showed poorer SEWB outcomes in the intervention arm in the medium-term.<br>1 of 8 comparisons showed improved SEWB outcomes in the intervention arm in the long-term.<br>Incomplete reporting of the sub-group analyses.
				Post	Medium				○		
				Post	Long		Ø		○		
**Geographically targeted**											
**Chang 2015**<br>Low	cRCT	Unclear	83% of mothers attended all visits	Post	Short	○	○	○		○	Improved cognitive outcome in intervention group on adjusting for potential confounders.
**Cupples 2011**<br>High	iRCT	Low	Mean number of contacts = 8.5 (of 22 planned)	Post	Immed	○		○	○		
**Doyle 2011**<br>1 high & 1 moderate	iRCT	Unclear	High: Mean number of visits = 46<br>Moderate: Not reported	During	Short	○	○	○	○	○	3 of 12 comparisons showed improved cognitive outcomes, and 1 of 62 comparisons showed improved SEWB outcomes in the intervention arm, in the long-term whilst intervention was ongoing.
				During	Medium	○	○	○	○	○	
				During	Long	○	○	○	○	○	
**Griffith 2011**<br>High	iRCT	Low	60% attended 8 or more sessions (of 12 planned)	Post	Short				○	○	
**High 2000**<br>Low	iRCT	High	Mean number of visits = 3.4 (of 5 planned)	Post	Short		○				3 of 6 comparisons showed improved language outcomes in intervention group.<br>Sub-group results: no differences seen in 13-17 month olds; 6 of 6 comparisons in 18-25 month olds showed improved language outcomes in intervention group; no test for interaction presented.
**Landry 2006**<br>2 high intensity	iRCT	Unclear	91% of parents completed all 10 visits plus 2 assessment visits	Post	Short		●	●	○		2 of 2 comparisons showed improved language outcomes, and 1 of 1 comparison showed improved cognatic outcome in the intervention arm.<br>3 of 6 comparisons showed improved SEWB outcomes in the intervention arm.<br>Interaction with birthweight examined, but reporting of results incomplete.
**Wagner 2001**<br>High	iRCT	Unclear	44% of families still receiving services at 2 yrs	During	Long	○	○	○	○		Results also stratified by income; no significant interactions reported.
**Wiggins 2004**<br>Moderate (SHV)	iRCT	Low	Mean number of visits = 7 (of 12 planned)	Post	Immed					○	1 of 1 comparison showed improved language outcome and 1 of 1 comparison showed improved overall development in intervention group.
				Post	Short		●		○	●	
**Wiggins 2004**<br>Low (CGS)	iRCT	Low	19% of women attended a group	Post	Immed					○	
				Post	Short		○		○	○	

D3: Results stratified by socio-economic status of the families included

Not enough information in a majority of papers to perform this comparison adequately.
