# Supplementary material for: Interventions that enhance health services for parents and infants to improve child development and social and emotional well-being in high-income countries: a systematic review
Source: BMJ Open. 2018 Feb 8;8(2):e014899. doi: 10.1136/bmjopen-2016-014899 (PMC5829600; doi:10.1136/bmjopen-2016-014899)
Supplement: Author's manuscript [file bmjopen-2016-014899.draft_revisions.pdf]

BMJ Open

Interventions that enhance health professional contact with parents and infants to improve child development and social and emotional wellbeing in high-income countries: A systematic review

Journal:	*BMJ Open*
Manuscript ID	bmjopen-2016-014899
Article Type:	Research
Date Submitted by the Author:	25-Oct-2016
Complete List of Authors:	Hurt, Lisa; Cardiff University School of Medicine, Division of Population Medicine<br>Paranjothy, Shantini; Cardiff University School of Medicine, Division of Population Medicine<br>Lucas, Patricia; Unversity of Bristol, School for Policy Studies<br>Watson, Debbie; University of Bristol, School for Policy Studies<br>Mann, Mala; Cardiff University, Specialist Unit for Review Evidence (SURE)<br>Griffiths, Lucy; UCL Great Ormond Street Institute of Child Health, Population, Policy and Practice Programme<br>Ginja, Samuel; Institute of Health and Society, Newcastle University<br>Paljarvi, Tapio; Cardiff University School of Medicine, Division of Population Medicine<br>Williams, Jo; Bristol City Council<br>Bellis, Mark; Bangor University, College of Health and Behavioural Sciences; Public Health Wales, Policy, Research and International Development<br>Lingam, Raghu; Institute of Health & Society, Newcastle University
**Primary Subject Heading**:	Evidence based practice
Secondary Subject Heading:	Health policy, Paediatrics, Public health, Epidemiology
Keywords:	Child development, Social and emotional wellbeing, Universal health services, Early intervention

SCHOLARONE™
Manuscripts

Interventions that enhance health professional contact with parents and infants to improve child development and social and emotional wellbeing in high-income countries: A systematic review

Lisa Hurt[1], Shantini Paranjothy[1], Patricia J Lucas[2], Debbie Watson[2], Mala Mann[3], Lucy J Griffiths[4], Samuel Ginja[5], Tapio Paljarvi[1], Jo Williams[6], Mark Bellis[7], Raghu Lingam[5]

[1] Division of Population Medicine, Cardiff University School of Medicine, 5th Floor, Neuadd Meirionnydd, Heath Park, Cardiff CF14 4YS

Lisa Hurt Lecturer

Shantini Paranjothy Mansel Talbot Professor of Preventive Medicine

Tapio Paljarvi Research Fellow

[2] School for Policy Studies, University of Bristol, 8 Priory Rd, Bristol BS8 1TZ

Patricia J Lucas Reader

Debbie Watson Reader

[3] Specialist Unit for Review Evidence, Cardiff University, Neuadd Meirionnydd, Heath Park, Cardiff CF14 4YS

Mala Mann Information Specialist/Systematic Reviewer

[4] Population, Policy and Practice Programme, UCL Great Ormond Street Institute of Child Health, 30 Guilford St, London WC1N 1EH

Lucy J Griffiths Senior Research Associate

[5] Institute of Health and Society, Newcastle University, Baddiley-Clark Building, Newcastle upon Tyne NE2 4AX

Samuel Ginja Research Assistant

Raghu Lingham Clinical Senior Lecturer/Honorary Consultant

[6] Bristol City Council, City Hall, PO Box 3176, Bristol BS3 9FS

Jo Williams Consultant in Child Public Health

[7] Public Health Wales, 2 Capital Quarter, Tyndall Street, Cardiff, CF10 4BZ

Mark Bellis Professor/Director of Policy, Research and Development

Correspondance to: L Hurt, hurtl@cardiff.ac.uk, 029 2068 7807

Word count: 4,000

For peer review only

Abstract

Background: Experiences in the first 1000 days of life have a critical influence on child development and health. Universal health services provide support for families, and new unevaluated components are often added to these.

Methods: We systematically reviewed the evidence for interventions in high-income countries to improve child development by enhancing universal health professional contact with parents from the antenatal period to two years postpartum. We searched 15 databases and trial registers for studies published in any language between 01/01/1996 and 01/04/2016, using subject headings and key words with the following search structure: [(health OR parenting professionals) OR known programme names] AND [child development OR motor OR cognitive OR emotional/behavioural OR language outcomes]. We also searched 58 programme or organisation websites and the electronic table of contents of eight journals.

Results: Primary outcomes were motor, cognitive, and language development, and social-emotional wellbeing, measured to three years of age. Results were reported using narrative synthesis, due to the heterogeneity in study populations, intervention design and outcome measurement. 22 of the 12,986 studies identified met eligibility criteria. Using GRADE criteria, the quality of evidence overall was moderate to low. There was limited evidence for intervention effectiveness: positive effects were seen in 1/6 studies for motor development, 4/11 for language development, 4/8 for cognitive development, and 3/19 for social-emotional wellbeing. However, most studies showing positive effects were at high/unclear risk of bias, within-study effects were inconsistent, and negative effects were also seen. Intervention content and intensity varied greatly, but this was not associated with effectiveness.

Conclusions: There is insufficient evidence that interventions currently available to enhance universal health services up to two years postpartum are effective for improving child development. There is an urgent need for robust evaluation of existing interventions, and to develop and evaluate novel interventions to enhance the universal offer.

For peer review only - http://bmjopen.bmj.com/site/about/guidelines.xhtml

Article summary

Strengths and limitations of this study

- To our knowledge, this is the first systematic review of interventions that enhance universal health service provision to improve child development outcomes including social and emotional wellbeing outcomes.
- We used a broad systematic search of the extensive literature in this field, and found 22 RCTs examining the effect of widely varying interventions (in both components and intensity) from the antenatal period to two years postpartum.
- It was not possible to conduct a meta-analysis due to the heterogeneity in the types of interventions and methods used to measure outcomes.
- However, the narrative review provides a comprehensive picture of the lack of an evidence-base in this field: very few positive effects on child outcomes were observed; these were mainly in trials classified as being at high or unclear risk of bias; and programmes of greater intensity (in terms of length, number or type of components) did not show more positive effects than programmes of lower intensity.
- More robust evaluations are needed of both existing and novel interventions to enhance the universal health service offer in the early years.

For peer review only

For peer review only - http://bmjopen.bmj.com/site/about/guidelines.xhtml

Introduction

Experiences in the first 1000 days of life have a crucial influence on child development and health.[1] Appropriate early child development (including physical, social and emotional, language and cognitive domains) has consistently been shown to be associated with good health and educational outcomes in childhood, and consequent health and employment outcomes in adulthood.[2-4] Adopting a life-course approach, including early intervention, is essential,[5] and investment is therefore needed in effective pre and postnatal services to optimise child health, wellbeing and developmental resilience.[6]

Universal health services have been developed to promote maternal and child health during pregnancy and the early years. The content of these varies across countries, but a recent review suggested that the best services in Europe are "*characterized by personalised ongoing support during pregnancy, choice in birth arrangements, postnatal support and advice, and paid parental leave for mothers and fathers*".[7] In most high income settings, early years services also work to a "proportionate universalism" model, where care is available to all, with enhanced support offered to families at high risk of adverse outcomes.[1]

There is high-quality global evidence to support the effectiveness of many components of universal early years services including elements of antenatal care and centre-based preschool provision.[7 8] Interventions to promote child development in the early years have also been developed. However, most have been targeted at and evaluated with high-risk families or children with an identified condition.[9-11] An unacceptably high proportion of children in both high and low income settings do not achieve expected early learning goals before they start school,[12] and it has been argued that targeted approaches alone may not therefore be not sufficient.[13] Universal interventions to enhance health professional contact with parents may be more effective in improving child development outcomes for several reasons. First, not all children who need support are identified by a targeted approach.[14] Targeting can lead to stigmatisation resulting in poor uptake or adherence.[15] Embedding interventions within a service, such as health visiting, which provides universal, ongoing and consistent support for parents, may also improve the interaction between health professionals and parents and improve access to care at a crucial time in their child's

For peer review only - http://bmjopen.bmj.com/site/about/guidelines.xhtml

development, leading to improvements in child development outcomes.[11] However, the effectiveness of such interventions is not known.

Previous reviews of early interventions fail to provide a full picture of interventions relevant to public health policy and practice because they do not provide a comprehensive examination of child development outcomes in the very early years (that is, the period during which the human brain develops most rapidly[16]). Neither does the evidence base to date include social and emotional wellbeing outcomes, nor are these consistently defined and articulated. The objective of this systematic review is to fill these gaps, by examining the effect of interventions designed to enhance universal health services provided to parents and children in the very early years (defined as the antenatal period to two years postpartum) on child development and social and emotional wellbeing outcomes.

Methods

Protocol and registration

The review protocol was developed in accordance with the Preferred Reporting Items for Systematic Reviews and Meta-Analysis (PRISMA) guidance. It was registered with Prospero (CRD42015015468).

Inclusion and exclusion criteria

We included randomised controlled trials (with individual or cluster randomisation), published or unpublished, that i) compared interventions to enhance health professional contact in the very early years with usual care, or ii) compared different interventions with each other. The interventions of interest were ones delivered alongside routine healthcare services that are the cornerstone of universal early years programmes in many countries (professional support, mainly provided by healthcare workers such as midwives or health visitors, most intensive during pregnancy and in the preschool years). We therefore included studies from the 76 countries and territories classified by the World Bank in July 2014 as "high-income economies". Studies published in any language were eligible for inclusion.

To capture the effects of interventions delivered in the very early years, we included programmes that were delivered at any time from the antenatal period to two years

For peer review only - http://bmjopen.bmj.com/site/about/guidelines.xhtml

postpartum. Given that some programmes continue beyond the child's second birthday, we specified that studies would be included if the mean age of the children at the start of the intervention was less than or equal to 24 months.

Studies that selected participants from the general population or from a specific geographic area (for example, an area-based programme defined on the basis of postcode or zip code) were included. Studies were excluded if they selected participants based on individual risk factors (for example, an individually-assessed income threshold for participating families, or parental illness) or specific clinical sub-groups (such as preterm babies or children with specific diagnoses).

Interventions

We included interventions that were provided within the framework of the existing healthcare system. They could be designed to augment universal routine healthcare provision for all children in different ways, for example by improving the skills or parental capacity of the parents or the family, improving the interaction between health professionals and parents, improving access to healthcare for the parents or the child, or including elements designed to promote a specific area of child development. These included training modules designed to be delivered to parents with the intention of improving child development outcomes, or any resources (such as printed materials, films, Apps) that health professionals or their support workers could use in their work with parents. Interventions could be delivered at home, in group-based settings (for example, in general, obstetric or paediatric practice, in hospitals or community settings), through telemedicine, or via a combination of different methods.

Outcomes

The outcomes were motor development (fine and gross), cognitive development, language development (receptive and expressive), social and emotional wellbeing; and global child development. We were primarily interested in studies that used validated tools to measure these outcomes, but also included descriptive indicators of child development as secondary outcomes. Studies were included if they measured outcomes at any time between three

For peer review only - http://bmjopen.bmj.com/site/about/guidelines.xhtml

months of age and three years postpartum (specifically, where the average age of the children at outcome measurement was 39 months or less).

Search strategy

We searched for articles published in any language between 01/01/1996 and 01/04/2016 in the following databases: Cochrane Central Register of Controlled Trials (CENTRAL); Medline; Embase; CINAHL; PsycINFO; Web of Science; Scopus; ASSIA; LiLACS; Sociological Abstracts; Social Services Abstract; and OpenGrey; and the following trial registers: ClinicalTrials.gov; UK Clinical Trials Gateway; and WHO International Clinical Trials Registry Platform. We used a combination of Medical Subject Headings and free text including terms for interventions to enhance professional contact combined with terms relating to child development outcomes. Terms for the interventions included those that listed the professional delivering the programme (including "health visitor", "community nurse", "nurse", "midwife", "general practitioner", "early years educator", "parent educator"), and programme names that were already known to the review team. The Medline search strategy is shown in Web Appendix A. We also searched websites of 58 relevant programmes and organisations, and the electronic table of contents (eTOC) of eight key journals for relevant studies published within the last two years (see Web Appendix B for a full list). Reference lists of included and key papers were reviewed, and authors contacted for additional data where necessary.

Study selection

All references identified by the searches were downloaded into Endnote and duplicates removed. Titles and abstracts were screened for inclusion by two reviewers independently (completed by LH, LJG and SP). Full text versions were obtained for the papers potentially meeting the inclusion criteria and were independently screened by two authors (completed by LH, LJG and SP). Disagreements were resolved through discussion and in consultation with others in the review group.

Data abstraction process

A data extraction form designed for the requirements of this review was used, which included details on the characteristics of the included studies, the interventions studied and assessment of risk of bias and GRADE criteria. Multiple publications and reports from the

For peer review only - http://bmjopen.bmj.com/site/about/guidelines.xhtml

same trial were linked, and compared for completeness and contradictions. Data from each paper were extracted by two reviewers independently (completed by LH, LJG, SG, SP and TP).

Analysis

Risk of bias was assessed following Cochrane guidelines.[17] Due to heterogeneity in i) the populations studied, ii) the design of the interventions and iii) the wide range of outcome measures used (both in terms of the child development domains and/or the instruments used to assess the outcomes), results were reported using narrative synthesis. We specified a priori that we would examine the results stratified by i) risk of bias, ii) the intensity of the intervention, iii) the age of the child at which the intervention was delivered, iv) whether the programme was universal or geographically targeted, and v) socio-demographic characteristics of the families in the trial. An assessment of the intensity of each intervention was conducted independently by two reviewers (completed by LH, LJG, SG, SP and TP) based on seven criteria: i) total number of visits; ii) total duration of the programme; iii) total number of contact hours; iv) frequency of visits; v) number of components; vi) whether components were delivered directly to parents and/or children; and vii) whether the components were delivered on a one-to-one basis or in a group session. Using these seven characteristics, we categorised the overall intensity for each intervention as "low", "moderate" or "high". Two review authors made this assessment using subjective determination rather than a predefined algorithm or a scoring system. The advantage of this was to allow for the diversity and complex combinations of components to be reflected in the categorisation (as used in [18]). Finally, the quality of the overall evidence for each outcome was assessed using GRADE criteria.[19]

Public Involvement

This work was conducted in collaboration with the Bristol Network for Early Years Health and Wellbeing (www.bonee.org) and a range of stakeholders have been involved in the design and conduct of this initiative. Parents were not involved in the design and conduct of the review, but we are discussing the results and interpretation with parents.

For peer review only - http://bmjopen.bmj.com/site/about/guidelines.xhtml

Results

15,280 records were identified in the database searches (Figure 1). Searches of relevant programme and organisation websites, and eTOC searches yielded 83 additional records. Once all searches were combined and duplicates removed, 12,986 records remained. After title and abstract screening, 12,644 records that were outside the scope of the review were excluded. Of the 342 records included in full text review, there were 22 RCTs that fulfilled our inclusion criteria (reported in 40 publications[20-59]). We also identified three relevant ongoing trials.[60-62] Reasons for exclusion are provided in Figure 1.

Trial characteristics

The 22 included trials are described in Table 1. Three were cluster randomised (clinics[22 43] or healthcare workers[55]), with the remainder randomising individual mothers, parents or mother-child dyads. Ten were conducted in the United States of America, three in the United Kingdom, two in Canada, two in Ireland, one in Australia, one in Chile, one in Japan, and one was a multi-site study conducted in four Southern European countries. Chang (2015) was conducted in Antigua, Jamaica and St Lucia, and is included because Antigua is classified as a high income country. All of the trials offered universal coverage of the intervention within a population, although in eight studies, the delivery of the intervention was geographically targeted (for example, recruitment occurred in hospitals known to serve areas of a city with high levels of relative disadvantage or the intervention was made available to all individuals within specific postcodes).[22 25 27 38 41 45 56 58] Three trials also included first time mothers only.

17 trials compared one intervention to usual care[22-25 33 34 38 41 43 47 48 51-53 55 56 58], although minor augmentations to usual care were made in six of these (for example, with some other information or services made available to parents who wished to access them[23 33 34 53 55 56]). One of the trials compared two different interventions with usual care.[58] In the remaining five trials, two interventions were compared with each other.[21 27 44-46] The timing of intervention delivery varied, from the first month of life only[21] to longer-term interventions, with eight studies including interventions that continued beyond the child's second birthday,[24 27 33 38 44 47 48 52] and the maximum intervention length being five years.[47] Studies ranged from 28 to 1593 participants: six included fewer than 100 participants; twelve

For peer review only - http://bmjopen.bmj.com/site/about/guidelines.xhtml

included between 100 and 500; and three included more than 500 participants. One trial did not report the number of subjects recruited or analysed.[55] In 17 of the 22 trials, outcome data were available for 75% or more of those randomised.

The mode of delivery of the intervention varied between trials. The intervention was delivered by health professionals in seven trials,[21-23 41 43 44 55] by other professionals (including "parent educators", "family visitors", or researchers) in eight trials,[27 33 45 47 51-53 56] by a mixture of health and other professionals in three trials,[34 38 48] and by peer mentors in one trial.[25] One trial examined one intervention delivered by health professionals and another delivered by community support groups.[58] In the remaining trials, one included materials delivered to parents by post,[24] and one examined training for parents by a physiotherapist to deliver a handling and positioning intervention.[46]

Six trials were classified as being at low risk of bias, one was at high risk, and 15 had an unclear risk (Figures 2 and 3).

Intervention characteristics

28 interventions were examined in total (see Table 2: 2a for studies that included one intervention; and 2b for studies that compared two interventions). Seven were of low intensity: short films followed by group discussions shown in health centre waiting rooms (Chang, 2a); sets of building blocks and activity hand-outs sent to parents by post (Christakis, 2a); "*literacy promoting anticipatory guidance*" by paediatricians (High, 2a); a brief parenting course (Hiscock, 2a); access to community groups (Wiggins, intervention 2, 2a); and two different methods for giving feedback to mothers on a neonatal behavioural assessment (Beeghly, 2b). Ten were of moderate intensity. These included one-to-one home visits (between five [Cheng, 2a] and twelve visits [Wiggins, 2a] in total), group sessions (up to eight in total [Feinberg, Niccols 2008, Niccols 2009, all 2a]), training for primary healthcare workers in interview techniques that encouraged consideration of child development (Tsiantis, 2a), training for parents in daily activities to promote motor development (Lobo, 2b) or a combination of different components (Santelices 2a, Doyle 2b). 11 interventions were of high intensity. They were classified as such because they included

For peer review only - http://bmjopen.bmj.com/site/about/guidelines.xhtml

multiple components (up to a maximum of 8), and regular contact with parents over a sustained period of time or intensively for a shorter period of time.

Uptake and adherence to intervention components was variable and inconsistently reported. For example, for low intensity interventions, this ranged from only 19% of the women accessing the intervention at all (community support groups[58]) to 83% accessing every session.[22] Patterns of adherence to the moderate and high intensity interventions also varied.

A full summary of the results including methods of outcome assessment for each trial is given in Web Appendix D. Many of the trials reported several measures of the same outcome and/or measured outcomes at different time points, resulting in multiple comparisons for each outcome. The findings are summarised by outcome in Table 3, and are described below.

Motor development outcomes

Six studies, including a total of 37 comparisons in 1276 participants, reported motor development outcomes using validated tools. The quality of the evidence was moderate. Three studies comparing one intervention with usual care showed no effect (972 participants, three comparisons), and three studies in which two interventions were compared (304 participants) showed no effect in 25 of 34 comparisons. The positive effects were all from one study of 28 infants who received a daily 15-minute handling and positioning intervention or a social interaction intervention for three weeks.[46]

Language development outcomes

Ten studies including a total of 43 comparisons examined this outcome, with nine using validated tools. The total number of participants for this outcome is unknown as one study did not report numbers,[55] but was in excess of 3,000. The quality of the evidence was low. Seven studies comparing one intervention with usual care showed no effect in ten comparisons, a positive effect in four comparisons and a negative effect (poorer language development in the intervention group) in two comparisons. Three studies which compared

For peer review only - http://bmjopen.bmj.com/site/about/guidelines.xhtml

two interventions (632 participants) found no effect in 23 comparisons and a positive effect in 4 comparisons.

Cognitive development outcomes

Eight studies, including a total of 40 comparisons in 2245 participants, examined cognitive development outcomes. All used validated tools, except for one where videotaped interactions were coded for "*independent goal-directed play*".[45] The quality of the evidence was low. In five studies (1729 participants) comparing one intervention with usual care, there was no effect in 18 of 20 comparisons, and a positive effect in two comparisons. Three studies which compared two interventions (516 participants) found no effect in 16 of 20 comparisons and a positive effect in four comparisons.

Social and emotional wellbeing outcomes

These outcomes were examined in 156 comparisons in 18 trials (total participant numbers unknown as one study did not report participant numbers,[55] but was in excess of 5,000). Many different outcomes were examined, with most assessed using validated tools (such as the Child Behaviour Checklist, the Infant Behaviour Questionnaire, the Parent-Infant Relationship Global Assessment, the Q-Sort Measure of the Security of Attachment, and social and emotional wellbeing scores from the Ages and Stages Questionnaire).

The quality of the evidence was low. In 15 studies comparing one intervention with usual care, there was no effect in 60 of 69 comparisons. In the three studies which compared two interventions (630 participants), there was no effect in 82 of 87 comparisons, a positive effect in four comparisons, and a negative effect in one comparison.

Overall development outcomes

Four studies including a total of 12 comparisons in 1565 participants examined global estimates of child development. The quality of the evidence was moderate. Three studies (1414 participants) comparing one intervention with usual care found no effect in seven of eight comparisons based on validated measures of global child development (Griffith Mental Development Scale,[22] and mean score from the Schedule of Growing Skills II[38]). The one positive effect in this group was in a study where mothers were asked whether they

For peer review only - http://bmjopen.bmj.com/site/about/guidelines.xhtml

perceived that their child's development was normal.[58] One study comparing two interventions (151 participants) found no effect in four comparisons (using the mean development score from the Ages and Stages Questionnaire).[27]

Sub-group effects

Sub-groups examined included families of different incomes, and children with different characteristics (for example, low birth-weight versus normal birth-weight infants, see Table 3). Some positive effects were seen, but the reporting of these analyses was generally incomplete, with an emphasis on positive effects. No conclusions can therefore be drawn on sub-groups in this review.

Stratification of results by risk of bias and intensity of interventions

Table 4 gives a summary of each study's results for each outcome (if examined) stratified by risk of bias and the intensity of the intervention. In the studies at low risk of bias, there was no intervention effect when either low or high intensity interventions were studied. Some positive effects were seen in the two trials of moderate intensity interventions, although in one, this was limited to sub-groups only (children with "disturbed" attachment at baseline),[23] and in the other, positive effects were not consistently seen.[58]

One study was classified as being at high risk of bias, and this examined a low intensity intervention.[41] Inconsistent positive effects were seen in this study, with most of these in one sub-group only.

The remaining studies were classified as being at unclear risk of bias, and there is no clear pattern the effects seen in these studies. Programme intensity does not appear to be associated with effectiveness in these studies.

No clear pattern in the results were seen when stratification by the other pre-specified variables was conducted (see Web Appendix E).

For peer review only - http://bmjopen.bmj.com/site/about/guidelines.xhtml

Discussion

The need for interventions to promote child development outcomes at a population-level has been clearly articulated. Using a broad systematic search of the extensive literature in this field, we found 22 RCTs examining the effect of interventions that enhance universal health services from the antenatal period to two years postpartum. The interventions varied greatly in their content and intensity, and uptake, adherence and fidelity were not consistently reported. The quality of evidence for motor development and overall child development was moderate, and the majority of comparisons showed no intervention effect. The quality of evidence for language development, cognitive development, and social and emotional wellbeing was low. The majority of the comparisons for these outcomes showed no effect, and where positive impacts were observed, within-study effects were inconsistent. We conclude that there is insufficient evidence to suggest that the interventions reviewed here are effective at improving child development outcomes. The low-to moderate quality of evidence overall suggests that further research is very likely to have an important impact on the conclusions that can be drawn.

The strength of our review was the broad search strategy, which encompassed many sources of information other than database searching. We are confident that we have identified most relevant studies (including three trials not yet published in peer-review journals). Although it was not possible to conduct a meta-analysis due to the heterogeneity in the types of interventions and methods used to measure outcomes, the narrative review provides a comprehensive picture of the lack of an evidence-base in this field. We do not report parental outcomes here, so cannot comment on whether parents benefitted from these enhancements.

To our knowledge this is the first systematic review of interventions which enhance universal health service provision to improve child development outcomes, including social and emotional wellbeing outcomes. Our conclusion is consistent with other reviews of early years interventions. For example, the Allen review[9] found that none of the universal interventions designed for use in the early years (defined as conception to school) had "best" quality evidence available to support them. A recent rapid review to update the evidence for components of the Healthy Child Programme in England also found few studies

For peer review only - http://bmjopen.bmj.com/site/about/guidelines.xhtml

of universal interventions aiming to promote child development outcomes in families with children in the 0-5 age range.[10] We reviewed a larger number of primary studies than either of these previous publications.

Understanding how universal provision can be enhanced to provide support for parents to achieve the best possible developmental outcomes for their children is necessary but challenging. Universal services consist of many components, many of these untested. Parents also access a wide variety of other forms of support, and the effects of these are poorly understood. Although the evidence-base examined in this review is limited, it does allow us to conclude that there is no convincing evidence that the interventions studied provide an additional benefit to the universal care currently provided in the settings included in these trials. There was also no evidence that interventions of high intensity confer more benefit than those of lower intensity. This is consistent with recent evidence for targeted interventions (such as the recent trial of the Family Nurse Partnership programme in the UK[63]).

Many interventions currently incorporated into health services have not been adequately evaluated, and we recommend further research to generate this evidence. We also note that adherence was poor across studies, and inconsistently reported. Future research should carefully report uptake, adherence and fidelity, to further develop our understanding of how to engage and retain families within programmes.[64 65] Research is also needed on whether new delivery platforms (such as technology-assisted interventions[66]) may provide a more engaging, feasible and cost-effective mechanism for providing support to parents. There is insufficient evidence that, where universal health services are available to families with very young children, additional elements or enhancements to these improve child outcomes. Early intervention to improve child development is a public health priority, but funding is scarce. There is an urgent need for more robust evaluation of existing interventions, and to develop and evaluate novel intervention packages to enhance the offer to all families.

For peer review only - http://bmjopen.bmj.com/site/about/guidelines.xhtml

Competing interests

All authors have completed the Unified Competing Interest form at www.icmje.org/coi_disclosure.pdf (available on request from the corresponding author) and declare no financial relationships with any organisations that might have an interest in the submitted work in the previous three years, and no other relationships or activities that could appear to have influenced the submitted work.

Contributorship statement

LH, SP, PL, DW, JW and RL conceived and designed the study. MM designed the search strategy (in consultation with other review authors) and performed the database searches. LH and LJG searched the websites and journal table of contents. LH and LJG selected and reviewed eligible reports. LH, LJG, SG, SP and TP extracted data. LH drafted the paper. All authors commented on and revised the paper, and approved the final version. LH is the guarantor for the paper.

Funding

This work was funded by Public Health Wales. The Director of Policy, Research and Development at Public Health Wales (Professor Mark Bellis) provided expert technical advice during discussions on the study design, the interpretation of the results, and the drafting of the paper, and is an author on the paper.

Data sharing statement

This paper reports on a systematic review. No original study data was obtained from the authors of the trials included in the review. Extra data (such as the results of the review stratified by characteristics other than those included in the report) is available by emailing Lisa Hurt.

For peer review only - http://bmjopen.bmj.com/site/about/guidelines.xhtml

References

1. Marmot M, Allen J, Goldblatt P, et al. Fair Society, Healthy Lives: The Marmot Review. London: University College London, 2010.

2. Rough E, Goldblatt P, Marmot M, et al. Inequalities in child health. In: Foyle G, Nathanson V, eds. Growing up in the UK - Ensuring a healthy future for our children. London: British Medical Association, 2013:37-56.

3. Shonkoff, J, Phillips, D (eds). *From Neurons to Neighborhoods: The Science of Early Childhood Development.* Washington, DC.: National Academy Press, 2000.

4. Stewart-Brown S, Fletcher L, Wadsworth M. Parent-child relationships and health problems in adulthood in three UK national birth cohort studies. European Journal of Public Health 2005;**15**(6):640-6.

5. World Health Organization. The Minsk Declaration: The Life-Course Approach in the Context of Health 2020. Denmark: WHO Regional Office for Europe, 2015.

6. Irwin L, Siddiqui A, Hertzman C. Early Child Development: A Powerful Equalizer. Final Report for the World Health Organization's Commission on the Social Determinants of Health. Vancouver, Canada: Human Early Learning Partnership, University of British Columbia., 2007.

7. Jensen B, Currie C, Dyson A, et al. European Review of Social Determinants of Health and the Health Divide in the WHO European Region. Copenhagen, Denmark: WHO Regional Office for Europe, 2013.

8. DiMario S, Basevi V, Gori G, et al. What is the effectiveness of antenatal care? (Supplement). Health Evidence Network report. Copenhage, Denmark: WHO Regional Office for Europe, 2005.

9. Allen G. Early Intervention: The Next Steps. Londo, UK: Cabinet Office, 2011.

10. Axford N, Barlow J, Coad J, et al. Rapid Review to Update Evidence for the Healthy Child Programme 0–5. London, UK: Public Health England, 2015.

11. Barlow J, Smailagic N, Ferriter M, et al. Group-based parent-training programmes for improving emotional and behavioural adjustment in children from birth to three years old. Cochrane Database of Systematic Reviews 2010;**Issue 3.**:Art. No.: CD003680.

12. Britto P. School Readiness: a conceptual framework. New York, USA: United Nations Children's Fund, 2012.

For peer review only - http://bmjopen.bmj.com/site/about/guidelines.xhtml

13. Sanders M, Kirby J. A Public-Health Approach to Improving Parenting and Promoting Children's Well-Being. Child Development Perspectives 2014;**8**(4):250-7.

14. Bayer J, Hiscock H, Morton-Allen E, et al. Prevention of mental health problems: rationale for a universal approach. Archives of Diseases in Childhood 2007;**92**:34-8.

15. Prinz R, Sanders M. Adopting a population-level approach to parenting and family support interventions. Clinical Psychology Review 2007;**27**(6):739-49.

16. Walker S, Wachs T, Grantham-McGregor S, et al. Inequality in early childhood: risk and protective factors for early child development. Lancet 2011;**378**:1325–38.

17. Higgins J, Green S, editors. *Cochrane Handbook for Systematic Reviews of Interventions Version 5.1.0 [updated March 2011]*. The Cochrane Collaboration, 2011.

18. Baker P, Francis D, Soares J, et al. Community wide interventions for increasing physical activity. Cochrane Database of Systematic Reviews 2015;**Issue 1**:Art. No.: CD008366.

19. Guyatt G, Oxman A, Akl E, et al. GRADE guidelines: 1. Introduction—GRADE evidence profiles and summary of findings tables Journal of Clinical Epidemiology 2011;**64**(4):383–94.

20. Bayer J, Hiscock H, Ukoumunne O, et al. Three-year-old outcomes of a brief universal parenting intervention to prevent behaviour problems: randomised controlled trial. Archives of Diseases in Childhood 2010;**95**:187–92.

21. Beeghly M, Brazelton T, Flannery K, et al. Specificity of preventative pediatric intervention effects in early infancy. Developmental and Behavioral Pediatrics 1995;**16**(3):158-66.

22. Chang S, Grantham-McGregor S, Powell C, et al. Integrating a Parenting Intervention With Routine Primary Health Care: A Cluster Randomized Trial. Pediatrics 2015;**136**(2):272-80.

23. Cheng S, Kondo N, Aoki Y, et al. The effectiveness of early intervention and the factors related to child behavioural problems at age 2: A randomized controlled trial. Early Human Development 2007;**83**:683–91.

24. Christakis D, Zimmerman F, Garrison M. Effect of Block Play on Language Acquisition and Attention in Toddlers. A Pilot Randomized Controlled Trial. Arch Pediatr Adolesc Med 2007;**161**(10):967-71.

For peer review only - http://bmjopen.bmj.com/site/about/guidelines.xhtml

25. Cupples M, Stewart M, Percy A, et al. A RCT of peer-mentoring for first-time mothers in socially disadvantaged areas (The MOMENTS Study). Archives of Diseases in Childhood 2011;**96**:252–58.

26. Doyle O. Barrington Lecture 2011-12. Breaking the Cycle of Deprivation: An Experimental Evaluation of an Early Childhood Intervention. Journal of the Statistical and Social Inquiry Society of Ireland 2012;**XLI**:92-111.

27. Doyle, O. and the Preparing for Life Evaulation Team. Preparing for Life: Early Childhood Intervention. Assessing the Early Impact of Preparing for Life at Six Months. Dublin, Ireland: UCD Geary Institute, 2011.

28. Doyle, O. and the Preparing for Life Evaulation Team. Preparing For Life: Early Childhood Intervention. Assessing the Impact of Preparing For Life at Twelve Months. Dublin, Ireland: UCD Geary Institute, 2012.

29. Doyle, O. and the Preparing for Life Evaulation Team. Preparing For Life:Early Childhood Intervention. Assessing the Impact of Preparing For Life at Eighteen Months. Dublin, Ireland: UCD Geary Institute, 2013.

30. Doyle, O. and the Preparing for Life Evaulation Team. Preparing For Life: Early Childhood Intervention. Assessing the Impact of Preparing For Life at Twenty-four Months. Dublin, Ireland: UCD Geary Institute, 2013.

31. Doyle, O. and the Preparing for Life Evaulation Team. Preparing For Life: Early Childhood Intervention. Assessing the Impact of Preparing For Life at Thirty-six Months. Dublin, Ireland: UCD Geary Institute, 2014.

32. Doyle O, McGlanaghy E, O'Farrelly C, et al. Can Targeted Intervention Mitigate Early Emotional and Behavioral Problems?: Generating Robust Evidence within Randomized Controlled Trials. PLOS One 2016;**11**(6):e0156397.

33. Drotar D, Robinson J, Jeavons L, et al. A randomized, controlled evaluation of early intervention: the Born to Learn curriculum. Child: Care, Health and Development 2008;**35**(5):643–49.

34. Feinberg M, Kan M. Establishing Family Foundations: Intervention Effects on Coparenting, Parent/Infant Well-Being, and Parent–Child Relations. Journal of Family Psychology 2008;**22**(2):253–63.

For peer review only - http://bmjopen.bmj.com/site/about/guidelines.xhtml

35. Feinberg M, Kan M, Goslin M. Enhancing Coparenting, Parenting, and Child Self-Regulation: Effects of Family Foundations 1 Year after Birth. Prevention Science 2009;**10**:276–85.

36. Feinberg M, Jones D, Kan M, et al. Effects of Family Foundations on Parents and Children: 3.5 Years After Baseline. Journal of Family Psychology 2010;**24**:532–42.

37. Feinberg M, Jones D, Roettger M, et al. Long-Term Follow-Up of a Randomized Trial of Family Foundations: Effects on Children's Emotional, Behavioral, and School Adjustment. Journal of Family Psychology 2014;**28**(6):821–31.

38. Griffith N. Evaluating the Incredible Years Toddler programme in disadvantaged areas of Wales. Bangor University, 2011.

39. Gridley N, Hutchings J, Baker-Henningham H. The Incredible Years Parent–Toddler Programme and parental language: a randomised controlled trial. Child: Care, Health and Development 2015;**41**(1):103–11.

40. Guyer B, Hughart N, Strobino D, et al. Assessing the Impact of Pediatric-Based Developmental Services on Infants, Families, and Clinicians: Challenges to Evaluating the Healthy Steps Program. Pediatrics 2000;**105**(3):e33.

41. High P, LaGasse L, Becker S, et al. Literacy Promotion in Primary Care Pediatrics: Can We Make a Difference? Pediatrics 2000;**105**(4):927–34.

42. Hiscock H, Bayer J, Wake M. Preventing Toddler Externalising Behaviour Problems: Pilot Evaluation of a Universal Parenting Program. International Journal of Mental Health Promotion 2005;**7**(2):54-60.

43. Hiscock H, Bayer J, Price A, et al. Universal parenting programme to prevent early childhood behavioural problems: cluster randomised trial. British Medical Journal 2008;**336**:318.

44. Johnston B, Huebner C, Anderson M, et al. Healthy Steps in an Integrated Delivery System. Child and Parent Outcomes at 30 Months. Arch Pediatr Adolesc Med 2006;**160**:793-800.

45. Landry S, Smith K, Swank P. Responsive Parenting: Establishing Early Foundations for Social, Communication, and Independent Problem-Solving Skills. Developmental Psychology 2006;**42**(4):627–42.

46. Lobo M, Galloway J. Enhanced Handling and Positioning in Early Infancy Advances Development Throughout the First Year. Child Development 2012;**83**(4):1290–302.

For peer review only - http://bmjopen.bmj.com/site/about/guidelines.xhtml

47. Miller S, Dunne L. A Randomised Controlled Trial Evaluation of the Lifestart Parenting Programme. Belfast, Northern Ireland: Queen's University, 2015.

48. Minkovitz C, Strobino D, Hughart N, et al. Early effects of the Healthy Steps for Young Children Program. Arch Pediatr Adolesc Med 2001;**155**:470-79.

49. Minkovitz C, Hughart N, Strobino D, et al. A practice-based intervention to enhance quality of care in the first 3 years of life. The Healthy Steps for Young Children Program Journal of the American Medical Association 2003;**290**(23):3081-91.

50. Zuckerman B, Parker S, Kaplan-Sanoff M, et al. Healthy Steps: A Case Study of Innovation in Pediatric Practice. Pediatrics 2004;**114**(3):820–26.

51. Niccols A. ‘Right from the Start’: randomized trial comparing an attachment group intervention to supportive home visiting. Journal of Child Psychology and Psychiatry 2008;**49**(7):754–64.

52. Niccols A. Immediate and short-term outcomes of the ‘COPEing with Toddler Behaviour’ parent group. Journal of Child Psychology and Psychiatry 2009;**50**(5):617–26.

53. Santelices M, Guzmán M, Aracena M, et al. Promoting secure attachment: evaluation of the effectiveness of an early intervention pilot programme with mother–infant dyads in Santiago, Chile. Child: Care, Health and Development 2010;**37**(2):203–10.

54. Tsiantis J, Dragonas T, Cox A, et al. Promotion of children’s early psychosocial development through primary health care services. Paediatric and Perinatal Epidemiology 1996;**10**:339-54.

55. Tsiantis J, Smith M, Dragonas T, et al. Early Mental Health Promotion in Children Through Primary Health Care Services: A Multi-Centre Implementation. Journal of Public Mental Health 2000;**2**(3):5-15.

56. Wagner M, Spiker D, Hernandez F, et al. Multisite Parents as Teachers evaluation. Experiences and outcomes for children and families. California, USA: SRI International, 2001.

57. Wagner M, Spiker D, Linn M. The Effectiveness of the Parents as Teachers Program with Low-Income Parents and Children. Topics in Early Childhood Special Education 2002;**22**(2):67-81.

58. Wiggins M, Oakley A, Roberts I, et al. The Social Support and Family Health Study: a randomised controlled trial and economic evaluation of two alternative forms of

For peer review only

For peer review only - http://bmjopen.bmj.com/site/about/guidelines.xhtml

postnatal support for mothers living in disadvantaged inner-city areas. Health Technology Assessment 2004;**8**(32):1-134.

59. Wiggins M, Oakley A, Roberts I, et al. Postnatal support for mothers living in disadvantaged inner city areas: a randomised controlled trial. J Epidemiol Community Health 2005;**59**:288–95.

60. Hiscock H, Bayer J, Lycett K, et al. Preventing mental health problems in children: the Families in Mind population-based cluster randomised controlled trial. BMC Public Health 2012;**12**:420.

61. Pontoppidan M. The effectiveness of the Incredible Years™ Parents and Babies Program as a universal prevention intervention for parents of infants in Denmark: study protocol for a pilot randomized controlled trial. Trials 2015;**16**:386.

62. Sawyer A, Lynch J, Bowering K, et al. An equivalence evaluation of a nurse-moderated group-based internet support program for new mothers versus standard care: a pragmatic preference randomised controlled trial. BMC Pediatrics 2014;**14**:119.

63. Robling M, Bekkers M-J, Bell K, et al. Effectiveness of a nurse-led intensive home-visitation programme for first-time teenage mothers (Building Blocks): a pragmatic randomised controlled trial. The Lancet 2016;**387**(10014):146–55.

64. Ingoldsby I. Review of interventions to improve family engagement and retention in parent and child mental health programs. J Child Fam Stud 2010;**19**(5):629–45.

65. Cullen S, Cullen M-A, Lindsay G. Universal parenting programme provision in England; Barriers to parent engagement in the CANparent trial, 2012-2014. Children and Society 2016;**30**:71-81.

66. Hall C, Bierman K. Technology-assisted interventions for parents of young children: Emerging practices, current research, and future directions. Early Childhood Research Quarterly 2015;**33**:21-32.

For peer review only - http://bmjopen.bmj.com/site/about/guidelines.xhtml

Table 1: Characteristics of the included studies

Study<br>Setting	Type and aim of study	Comparison group	i) Who received intervention, and<br>ii) When?	Sample size<br>i) Randomised<br>ii) In analysis (% of randomised)	Who delivered intervention?	Universal or geographically targeted?
**Beeghly 1995**<br>USA	Individual RCT, to compare the effectiveness of two one-to-one clinic-based interventions (infant-centred vs mother centred) on motor and cognitive development post-intervention	Two interventions compared	i) Mothers and infant<br>ii) When child was 3, 14 and 30 days of age	i) 163<br>ii) 125 (77%)	Health professionals	Universal
**Chang 2015**<br>Antigua, Jamaica & St Lucia	Cluster RCT, to examine the effectiveness of a group-based intervention on language and cognitive development post-intervention	Usual care	i) Mothers<br>ii) When child was 3, 6, 9, 12 and 18 months	i) 30 health centres randomised (501 women enrolled)<br>ii) 426 (85%)	Health professionals	Geographically targeted
**Cheng 2007**<br>Japan	Individual RCT, to examine the effectiveness of an individual home-based intervention on social and emotional wellbeing post-intervention	Usual care, with a counselling service made available to all	i) Mothers<br>ii) When child was 5-9 months	i) 95<br>ii) 85 (89%)	Health professionals	Universal
**Christakis 2007**<br>USA	Individual RCT, to examine the effectiveness of giving 2 sets of building blocks and a newsletter of activities to complete with them on language development and social and emotional wellbeing post-intervention	Usual care	i) Families<br>ii) When child was 18-30 months	i) 175<br>ii) 140 (80%)	No contact with families post-recruitment	Universal
**Cupples 2011**<br>UK (Northern Ireland)	Individual RCT, to examine the effectiveness of one-to-one contact with trained peer mentors on motor, cognitive development and social and emotional wellbeing post-intervention	Usual care	i) Mothers<br>ii) From 20 weeks of pregnancy to 12 months postpartum	i) 343<br>ii) 280 (82%)	Peer mentors	Geographically targeted (First time mothers only)
**Doyle 2011**<br>Ireland	Individual RCT, to compare the effectiveness of the "high support" versus "low support" versions of the multi-component "Preparing for Life" programme on motor, language, and cognitive development and social and emotional wellbeing whilst intervention was ongoing	Two interventions compared	i) Parents<br>ii) From pregnancy to school entry	i) 233<br>ii) 173 (74% at 6 months)<br>165 (71% at 12 months)<br>154 (66% at 18 months)<br>166 (71% at 24 months)<br>151 (65% at 36 months)	Other professionals	Geographically targeted
**Drotar 2008**<br>USA	Individual RCT, to examine the effectiveness of the multi-component "Born to Learn" programme on language and cognitive development and social and emotional wellbeing whilst intervention was ongoing	Usual care, plus handouts and offer of a different group meeting	i) Parents and child<br>ii) Recruited between birth and 9 months; programme continued to age 3	i) 527<br>ii) 410 (78%, although inconsistent numbers presented in tables)	Other professionals	Universal
**Feinberg 2008**<br>USA	Individual RCT, to examine the effectiveness of a group-based intervention ("Family Foundations") on social and emotional wellbeing post-intervention	Usual care plus brochure on childcare options	i) Parents<br>ii) Recruited during pregnancy, continued to age 4-6 months	i) 169<br>ii) 152 (90% at 6 months)<br>154 (91% at 12 months)<br>137 (81% at 36 months)	Mix of health and other professionals	Universal (First time mothers only)

For peer review only

Study Setting	Type and aim of study	Comparison group	i) Who received intervention, and ii) When?	Sample size i) Randomised ii) In analysis (% of randomised)	Who delivered intervention?	Universal or geographically targeted?
**Griffith 2011** UK (Wales)	Individual RCT, to examine the effectiveness of a group-based parenting intervention ("Incredible Years Toddler Programme") on social and emotional wellbeing and overall development post-intervention	Usual care (waiting list control group)	i) Parents ii) Children 12-36 months at baseline (mean age 21 months)	i) 89 ii) 89 (100%)	Mix of health and other professionals	Geographically targeted
**High 2000** USA	Individual RCT, to examine the effectiveness of a one-to-one clinic-based intervention on language development post-intervention	Usual care	i) Parents ii) Children 5-11 months at baseline	i) 205 ii) 153 (75%)	Health professionals	Geographically targeted
**Hiscock 2008** Australia	Cluster RCT, to examine the effectiveness of a group-based intervention ("Toddlers Without Tears") on social and emotional wellbeing post-intervention	Usual care	i) Parents ii) When child was 8, 12 and 15 months	i) 40 maternal and child health centres randomised (733 women enrolled) ii) 672 (92% at 18 months) 656 (89% at 24 months) 589 (80% at 36 months)	Health professionals	Universal
**Johnston 2006** USA	Individual RCT, to compare the effectiveness of the multi-component "Healthy Steps" programme with "Healthy Steps" plus "PrePare" on language development and social and emotional wellbeing post-intervention	Two interventions compared	i) Mothers ii) Recruited during pregnancy, continued to age 3	i) 303 ii) 239 (79%)	Health professionals	Universal
**Landry 2006** USA	Individual RCT, to compare the effectiveness of two different models of home visits, on language and cognitive development and social and emotional wellbeing post-intervention	Two interventions compared	i) Mothers ii) When child was 6-10 months	i) 264 ii) 240 (91%)	Other professionals	Geographically targeted
**Lobo 2012** USA	Individual RCT, to compare the effectiveness of a "handling and positioning" intervention with a "social interaction" intervention on motor development whilst the intervention was ongoing and post-intervention	Two interventions compared	i) Parents ii) For 3 weeks, from when child was 2 months of age	i) 28 ii) 28 (100%)	Intervention delivered by parents after training	Universal
**Miller 2015** Ireland	Individual RCT, to examine the effectiveness of the multi-component "Lifestart" programme on cognitive development and social and emotional wellbeing whilst intervention was ongoing	Usual care	i) Parents ii) Recruited when child < 12 months, continued to age 5	i) 435 ii) 347 (80% at 36 months)	Other professionals	Universal (although parents self-referred)
**Minkovitz 2003** USA	Individual RCT, to examine the effectiveness of the multi-component "Healthy Steps" programme on social and emotional wellbeing in the long term whilst intervention was ongoing	Usual care	i) Families ii) Recruited at birth or 1st well-child visit, continued to age 3	i) 2235 ii) 1593 (71%)	Mix of health and other professionals	Universal

For peer review only

Study<br>Setting	Type and aim of study	Comparison group	i) Who received intervention, and ii) When?	Sample size<br>i) Randomised<br>ii) In analysis	Who delivered intervention?	Universal or geographically targeted?
**Niccols 2008**<br>Canada	Individual RCT, to examine the effectiveness of a group-based intervention ("Right from the Start") on social and emotional wellbeing post-intervention	Usual care	i) Mothers<br>ii) Children 1-24 months at baseline	i) 76<br>ii) 73 (96% immediately post intervention)<br>64 (84% at 6 months)	Other professionals	Universal (although parents self-referred)
**Niccols 2009**<br>Canada	Individual RCT, to examine the effectiveness of a group-based intervention ("COPEing with Toddler Behaviour") on social and emotional wellbeing post-intervention	Usual care (waiting list control group)	i) Mothers<br>ii) Children 12-36 months at baseline (mean age 24 months)	i) 79<br>ii) 74 (94% immediately post-intervention)<br>71 (90% at 1 month)	Other professionals	Universal (although parents self-referred)
**Santelices 2011**<br>Chile	Individual RCT, to examine the efficacy of a multi-component intervention ("Promoting Secure Attachment") on social and emotional wellbeing post-intervention	Usual care, plus one lecture by a psychologist	i) Mothers<br>ii) Recruited during late pregnancy, continued to age 1	i) 100<br>ii) 72 (72%)	Other professionals	Universal (First time mothers only)
**Tsiantis 2000**<br>Cyprus, Greece, Portugal, Yugoslavia	Cluster RCT, to examine the effectiveness of training primary health care workers to use semi-structured interviews to promote language development and social and emotional wellbeing whilst intervention is ongoing	Usual care (healthcare workers in this group received one lecture)	i) Mothers<br>ii) Recruited during pregnancy, continued to age 2	i) 80 primary healthcare workers (number of women randomised not reported, "recruitment did not achieve target figures")<br>ii) Not reported	Health professionals	Universal
**Wagner 2001**<br>USA	Individual RCT, to examine the effectiveness of a multi-component intervention ("Parents as Teachers") on motor, language, and cognitive development and social and emotional wellbeing whilst intervention is ongoing	Usual care, plus age-appropriate toys "at regular intervals" and an annual child assessment	i) Mothers<br>ii) Recruited during pregnancy, continued to age 2	i) 665<br>ii) 266 (40%)	Other professionals	Geographically targeted
**Wiggins 2004**<br>UK (England)	Individual RCT, to examine the effectiveness of two postnatal social support interventions ("Support Health Visitors" and "Community Group Support") on language development, social and emotional wellbeing overall development post-intervention	Both interventions compared with usual care	i) Mothers<br>ii) Recruited when child was ~ 10 weeks, continued to age 1	i)731<br>ii) 657 (90% at 12 months)<br>601 (82% at 18 months)	1: Health professionals<br>2: Non health professionals	Geographically targeted

For peer review only

Table 2: Description of intervention components and intensity

Study	Description	Components*										Number, frequency, duration of contact	Adherence
		1	2	3	4	5	6	7	8	9	10		
**2a. Studies comparing one intervention with usual care**													
**Low intensity**													
**Chang 2015**	3 three-minute films demonstrating *"behaviours central to promoting child development"* shown as women waited for 3, 6, 9, 12 and 18 month vaccine visits, followed by group discussions with a community health worker. Cards given to reinforce messages, plus picture book at 9 and 12 months and puzzle at 18 months.			✓	✓			✓			✓	5, every 3-6 months, over 15 months	83% of mothers attended all visits
**Christakis 2007**	Parents were sent 2 sets of building blocks with accompanying newsletters containing suggested activities in the post				✓	✓		✓				2, bi-monthly, 3 months	Not reported
**High 2000**	Paediatricians gave books, handouts and "*literacy promoting anticipatory guidance*" to parents at routine well-child visits		✓		✓			✓				5, every 3 months, over 12 months	Mean number of visits = 3.4
**Hiscock 2008**	"*Universal anticipatory guidance*" with strategies for behavioural difficulties: handout at 8 months; 2 two-hour group sessions at 12 and 15 months			✓	✓							3, every 3-4 months, over 7 months	49% of parents attended all sessions
**Wiggins 2004 Intervention 2**	Access given to mothers to community group support that already existed and which provided drop in sessions and/or telephone support and/or home visits (different services provided by each of the 8 groups who agreed to take part in the study); participants selected whether to make contact and attend groups	✓		✓					✓			Variable	19% of women attended a community group
**Moderate intensity**													
**Cheng 2007**	5 one-to-one home visits of one hour *"aimed at improving the quality of mother-infant relationship"*; tailored encouragement and advice given following observation of mothers playing with infants	✓										5, monthly, over 5 months	Not reported
**Feinberg 2008**	Four prenatal and four postnatal interactive group sessions, designed *"to enhance coparenting"*			✓								8, every 6 weeks, over 11 months	80% attended at least 3 antenatal; 60% attended at least 3 postnatal sessions
**Niccols 2008**	8 two-hour group sessions using a "*coping modeling problem solving approach*", to enhance caregiver skills in "*reading infant cues and responding sensitively*" plus homework			✓								8, weekly, over 2 months	58% attended 4 or more sessions

* 1 = One-to-one home visits, 2 = One-to-one clinic visits, 3 = Group sessions, 4 = Handouts, 5 = Activities to perform at home, 6 = Developmental screening, 7 = Toys and/or books, 8 = Telephone support, 9 = Access to community resources, 10 = Other

For peer review only

Study	Description	Components[*]										Number, frequency, duration of contact	Adherence
		1	2	3	4	5	6	7	8	9	10		
**Moderate intensity**													
**Niccols 2009**	8 two-hour group sessions, using a "*coping modeling problem solving approach*", to train parents on effective parenting styles and strategies, plus homework.			✓		✓						8, weekly, over 2 months	Not reported
**Santelices 2011**	6 two-hour group sessions during pregnancy on "*maternal sensitivity...and to promote the development of a secure and healthy bond between mother and child*", and 4 hour-long one-to-one sessions postpartum to observe interactions and give feedback	✓	✓	✓								10, variable, over 16 months	Not reported
**Tsiantis 2000**	Primary health care workers trained to use a semi-structured interview technique during 6-8 routine visits to discuss age-appropriate child development topics.		✓									6-8, variable, over 36 months	Not reported
**Wiggins 2004 Intervention 1**	Supportive home visits conducted postnatally by five very experienced health visitors, adapted to each woman's needs to address her concerns and questions	✓										12, monthly, over 12 months	Mean number of visits = 7
**High intensity**													
**Cupples 2011**	Trained peer mentors provided one-to-one support on "health-related" topics via home visit or phone call	✓							✓			22, every 2-4 weeks, over 17 months	Mean number of contacts = 8.5
**Drotar 2008**	One-to-one home visits; monthly parent group sessions; annual developmental and health screening; access to resource network	✓		✓			✓			✓		27, every 2-4 weeks, over 36 months	Not reported
**Griffith 2011**	12 two-hour group sessions including watching videos, group discussions and role play, to help understand and manage child behaviour; homework tasks to complete.			✓		✓						12, weekly, over 3 months	60% attended 8 or more sessions
**Miller 2015**	30-60 minute monthly home visit by a Lifestart family visitor, and a monthly magazine ("Growing Child") of age-appropriate activities	✓			✓							35, monthly, over 36 months	Adherence data currently being analysed
**Minkovitz 2003**	Enhanced well-child care (12 visits, including access to Reach Out and Read literacy programme); 6 home visits in 3 years; telephone line; developmental screening; written guidance; monthly parent groups; links to community resources	✓	✓	✓	✓		✓		✓	✓		16, variable, over 36 months	79% of parents received 4 or more services
**Wagner 2001**	Monthly home visits and parent group meetings to provide information on child development and demonstrate age-appropriate activities. Periodic developmental screening and, if needed, referrals to community services provided.	✓		✓			✓			✓		24, monthly, over 24 months	44% of families still receiving services at 2 yrs

[*] 1 = One-to-one home visits, 2 = One-to-one clinic visits, 3 = Group sessions, 4 = Handouts, 5 = Activities to perform at home, 6 = Developmental screening, 7 = Toys and/or books, 8 = Telephone support, 9 = Access to community resources, 10 = Other

For peer review only

Study	Description	Components*										Number, frequency, duration of contact	Adherence
		1	2	3	4	5	6	7	8	9	10		
**2b. Studies comparing two interventions with each other**													
**Beeghly 1995** **1: Low intensity**	3 individual 45 minute sessions where mother observed a Neonatal Behavioral Assessment (NBAS) and discussed findings with a paediatrician (including exploring the care-giving that might promote the mother-child relationship)		✓									3, at 3, 14 and 30 days old	Not reported
**Beeghly 1995** **2: Low intensity**	3 individual 45 minute sessions, where mother discussed her perceptions of motherhood and concerns with a paediatrician, and was given feedback about an NBAS that was conducted in a different room		✓									3, at 3, 14 and 30 days old	Not reported
**Lobo 2012** **1: Moderate intensity**	Parents taught a positioning and handling programme during a home visit by a physiotherapist to be completed for 15 minutes daily for 3 weeks. 6 assessment home visits also completed. Caregivers given manual and a session diary.	✓			✓	✓						6, every 2 weeks, over 3 months	Excluded individuals who did not perform intervention on at least 60% of expected days
**Lobo 2012** **2: Moderate intensity**	Parents asked to engage their child in 15 minutes of face-to-face interaction daily for 3 weeks. This group also had 6 assessment visits.	✓										6, every 2 weeks, over 3 months	
**Doyle 2011** **1: Moderate intensity**	Access to a support worker; annual packs containing toys and books (worth 100 Euros); facilitated access to one year of preschool; stress control and healthy eating sessions		✓	✓ (2)				✓		✓	✓	Variable, over 36+ months	Not reported
**Doyle 2011** **2: High intensity**	Home visits from a trained mentor; tip sheets; Triple P Positive Parenting group sessions; baby massage; annual packs containing toys and books (worth 100 Euros); facilitated access to one year of preschool; stress control and healthy eating sessions	✓		✓ (3)	✓			✓		✓	✓	Variable, weekly, over 36+ months	Mean number of visits = 46
**Johnson 2006** **Intervention 1: High intensity**	Enhanced well-child care (6 visits, including Reach Out and Read literacy programme); 6 home visits in 3 years; telephone line; developmental screening; written guidance; monthly parent groups; links to community resources	✓	✓	✓	✓		✓		✓	✓		Variable, monthly, over 36+ months	Not reported
**Johnson 2006** **Intervention 2: High intensity**	As above, plus 3 additional home visits during 2nd half of pregnancy	✓	✓	✓	✓		✓		✓	✓		Variable, monthly, over 36+ months	Not reported
**Landry 2006** **Intervention 1: High intensity**	Playing and Learning Strategies: One-to-one home visits of 1.5 hours to discuss the child's current development and behaviour, feedback on videotaped interactions with child; and planning with mothers of how to increase their "*responsive*" behaviours	✓										10, weekly, over 3 months	91% of parents completed all 10 visits plus pre and post assessment visits
**Landry 2006** **Intervention 2: High intensity**	Developmental Assessment Screening: One-to-one home visits of 1.5 hours consisting of developmental screening and discussions on child development. Handouts on common issues (e.g. sleep, feeding) given.	✓			✓		✓					10, weekly, over 3 months	

* 1 = One-to-one home visits, 2 = One-to-one clinic visits, 3 = Group sessions, 4 = Handouts, 5 = Activities to perform at home, 6 = Developmental screening, 7 = Toys and/or books, 8 = Telephone support, 9 = Access to community resources, 10 = Other

For peer review only

Table 3: Summary of Findings

**Population:** Parents from antenatal period to 2 years postpartum<br>**Settings:** Universal programmes offered within defined populations<br>**Intervention:** Programmes to improve child development outcomes by enhancing health professional contact<br>**Comparison:** Usual care, or two different interventions compared		
**Number of studies**<br>**Total number of participants***<br>**Total number of comparisons†**	**Results**	**Quality of the evidence**
Outcome: Motor Development		
**Comparison:** Intervention with usual care		
3 studies[22 25 56]<br>972 participants, 3 comparisons	**No effect** in the 3 comparisons.<br>**No effect** in the 2 sub-groups examined in 1 study	MODERATE (downgraded one level because of risk of bias)
**Comparison:** Two interventions (Beeghly 1995, Doyle 2011, Lobo 2012)		
3 studies[21 27 46]<br>304 participants, 34 comparisons (20 in Doyle, 13 in Lobo)	**No effect** in 25 of the comparisons; **positive effect** in 9 comparisons (all in 1 study)<br>**No effect** in the 2 sub-groups examined in 1 study	MODERATE (downgraded one level because of risk of bias)
Outcome: Language Development		
**Comparison:** Intervention with usual care		
7 studies[‡ 22 24 33 41 54 56 58]<br>Participant numbers not known[§], 16 comparisons (6 in High)	**No effect** in 10 of the comparisons; **positive effect** in 4 comparisons (2 studies); **negative effect** in 2 tests (1 study)<br>Sub-group effects reported in 4 studies, with some positive effects seen, but reporting of sub-groups unclear and incomplete	LOW (downgraded two levels because of risk of bias and inconsistency[**])
**Comparison:** Two interventions		
3 studies[27 44 45]<br>632 participants, 27 comparisons (21 in Doyle)	**No effect** in 23 of the comparisons; **positive effect** in 4 tests (2 studies)<br>No sub-group effects examined	LOW (downgraded two levels because of risk of bias and inconsistency[**])
Outcome: Cognitive Development		
**Comparison:** Intervention with usual care		
5 studies[22 25 33 47 56]<br>1729 participants, 20 comparisons (16 in Drotar)	**No effect** in 18 of the comparisons; **positive effect** in 2 comparisons (2 studies)<br>Sub-group effects examined in 2 studies, with some positive effects seen, but reporting of sub-groups unclear and incomplete	LOW (downgraded two levels because of risk of bias and inconsistency[**])

**Comparison:** Two interventions		
3 studies[21 27 45]<br>516 participants, 20 comparisons (18 in Doyle)	**No effect** in 16 of the comparisons; **positive effect** in 4 comparisons (2 studies)<br>**No effect** in the 4 tests of interaction performed in 1 study	LOW (downgraded two levels because of risk of bias and inconsistency[**])
Outcome: Social and emotional wellbeing		
**Comparison:** Intervention with usual care		
15 studies[‡ 23-25 33 34 38 43 47 49 51-54 56 58]<br>Participant numbers not known[§], 69 comparisons (7 in Drotar, 14 in Feinberg, 8 in Niccols 2009, 16 in Tsiantis, 5 in Wagner)	**No effect** in 60 of the comparisons; **positive effect** in 9 comparisons (2 studies)<br>Sub-group effects examined in 4 studies, with some positive effects seen, but reporting of sub-groups unclear and incomplete	LOW (downgraded two levels because of risk of bias and inconsistency[**])
**Comparison:** Two interventions		
3 studies[27 44 45]<br>630 participants, 87 comparisons (78 in Doyle, 7 in Landry)	**No effect** in 82 of the comparisons; **positive effect** in 4 comparisons (2 studies); **negative effect** in 1 test (in 1 study)<br>Sub-group effects examined in 1 study, with some positive effects seen, but reporting of sub-groups unclear and incomplete	LOW (downgraded two levels because of risk of bias and inconsistency[**])
Outcome: Overall Child Development		
**Comparison:** Intervention with usual care		
3 studies[‡ 22 38 58]<br>1414 participants, 8 comparisons	**No effect** in 7 of the comparisons; **positive effect** in 1 comparison (1 study)<br>No sub-groups examined	MODERATE (downgraded ONE level because of Inconsistency[**])
**Comparison:** Two interventions		
1 study[27]<br>151 participants, 4 comparisons	**No effect** in the 4 comparisons	MODERATE (downgraded one level because of risk of bias)

[*] As the number of participants can vary within studies (e.g. where outcomes are measured at several different time points), the total number of participants noted here is calculated from the total numbers analysed at the last time point in each study; this is therefore a conservative estimate of the total number of participants for each outcome.
[†] Total number of comparisons performed for the specified outcome in the whole study population across all of the studies; studies in which 5 or more comparisons are conducted on the same outcome (either at one time point or across different time points) are referenced
[‡] Includes both comparisons in Wiggins (supportive health visiting with usual care, and community groups with usual care)
[§] Total participant numbers not reported in Tsiantis
[**] Inconsistency noted where i) positive, negative and no effects are reported for an outcome, and/or ii) there is a different effect seen in 25% or more of comparisons, and/or iii) different effects are reported within a study, and/or iv) most of the positive or negative effects are seen in sub-groups only and the reporting of sub-group effects is incomplete or inconsistent

GRADE Working Group grades of evidence
High quality: Further research is very unlikely to change our confidence in the summary of the effects.
Moderate quality: Further research is likely to have an important impact on our confidence in the summary of the effects.
Low quality: Further research is very likely to have an important impact on our confidence in the summary of the effects.
Very low quality: We are very uncertain about the summary of the effects.

For peer review only

Table 4: Results for effects on motor, language, and cognitive development, social and emotional wellbeing and overall development by risk of bias and intervention intensity (in whole study sample unless stated otherwise)

Study<br>Intensity of intervention	Motor development outcomes	Language development outcomes	Cognitive development outcomes	Social and emotional wellbeing outcomes	Overall child development
**4a. Studies comparing one intervention with usual care**					
**Low risk**					
**Hiscock 2008**<br>Low intensity	-	-	-	Short, medium and long term post-intervention:<br>No effect (6 tests)	-
**Wiggins 2004**<br>Low intensity (CGS)	-	Short term post-intervention:<br>No effect (1 test)	-	Short term post-intervention:<br>No effect (1 test)	Immediately post-intervention:<br>No effect (1 test)<br>Short term post-intervention:<br>No effect (2 tests)
**Cheng 2007**<br>Moderate intensity	-	-	-	Short term post-intervention:<br>No comparisons in whole sample;<br>Positive effect in 1 of 2 sub-group tests<br>Long term post-intervention:<br>No effect in whole sample (3 tests)	-
**Wiggins 2004**<br>Moderate intensity (SHV)	-	Short term post-intervention:<br>Positive effect (1 test)	-	Short term post-intervention:<br>No effect (1 test)	Immediately post-intervention:<br>No effect (1 test)<br>Short term post-intervention:<br>No effect in 1 & positive effect in 1 of 2 tests
**Cupples 2011**<br>High intensity	Immediately post-intervention:<br>No effect (1 test)	-	Immediately post-intervention:<br>No effect (1 test)	Immediately post-intervention:<br>No effect (4 tests)	-
**Griffith 2011**<br>High intensity	-	-	-	Short-term post-intervention:<br>No effect (1 test)	Short-term post-intervention:<br>No effect (1 test)
**Miller 2015**<br>High intensity	-	-	Long-term whilst intervention ongoing:<br>No effect (1 test)	Long-term whilst intervention ongoing:<br>No effect (1 test)	-
**High risk**					
**High 2000**<br>Low intensity	-	Short-term post-intervention:<br>No effect in 3 & positive effect in 3 of 6 tests<br>No effect in 6 & positive effect in 6 of 12 tests in sub-groups	-	-	-

Study<br>Intensity of intervention	Motor development outcomes	Language development outcomes	Cognitive development outcomes	Social and emotional wellbeing outcomes	Overall child development
**4a. Studies comparing one intervention with usual care (continued)**					
**Unclear risk**					
**Chang 2015**<br>**Low intensity**	Short term post-intervention:<br>No effect (1 test)	Short term post-intervention:<br>No effect (2 tests)	Short term post-intervention:<br>No effect in unadjusted test; positive effect on adjusting for multiple potential confounders	-	Short term post-intervention:<br>No effect (1 test)
**Christakis 2007**<br>**Low intensity**	-	Short term post-intervention:<br>No effect (2 tests)<br>Positive effect in 2 sub-group tests (results only presented for middle and low income group)	-	Short term post-intervention:<br>No effect in whole sample (1 test) or sub-groups (1 test)	-
**Feinberg 2008**<br>**Moderate intensity**	-	-	-	Short term post-intervention:<br>No effect in 2 & positive effect in 3 of 5 tests<br>Medium term post-intervention:<br>No effect in 1 & positive effect in 1 of 2 tests<br>Long term post-intervention:<br>No effect in whole sample (7 tests)<br>5 significant interactions (of 7 tested, suggesting intervention effects in boys)	-
**Niccols 2008**<br>**Moderate intensity**	-	-	-	Immediately and short term post-intervention:<br>No effect (2 tests)	-
**Niccols 2009**<br>**Moderate intensity**	-	-	-	Immediately and short term post-intervention:<br>No effect in 3 & positive effect in 5 of 8 tests	-
**Santelices 2011**<br>**Moderate intensity**	-	-	-	Immediately to short term post-intervention:<br>No effect (1 test)	-

For peer review only

Study<br>Intensity of intervention	Motor development outcomes	Language development outcomes	Cognitive development outcomes	Social and emotional wellbeing outcomes	Overall child development
**4a. Studies comparing one intervention with usual care (continued)**					
**Unclear risk**					
**Tsiantis 2000**<br>Moderate intensity	-	Long term whilst intervention ongoing:<br>Negative effect in 2 of 2 tests<br>Sub-group (country-specific) results not presented	-	Short term whilst intervention ongoing:<br>No effect in whole sample (2 tests)<br>Positive effect in 1 of 8 tests in sub-groups<br>Medium term post-intervention:<br>No effect in 1 & negative effect in 1 of 2 tests in whole sample<br>Long term whilst intervention ongoing:<br>No effect in 7 & positive effect in 1 of 8 tests<br>Inconsistent reporting of sub-groups, 2 positive effects mentioned but total number of tests not specified	-
**Drotar 2008**<br>High intensity	-	Long term whilst intervention ongoing:<br>No effect (1 test)	Short-medium term whilst intervention ongoing:<br>No effect (5 tests)<br>Long term whilst intervention ongoing:<br>No effect in 11 & positive effect in 1 of 12 tests<br>No effect in 10 & positive effect in 2 of 12 sub-group tests	Short, medium and long term whilst intervention ongoing:<br>No effect (6 tests)	-
**Minkovitz 2003**<br>High intensity	-	-	-	Long-term whilst intervention ongoing:<br>No effect (3 tests)	-
**Wagner 2001**<br>High intensity	Long term whilst intervention ongoing:<br>No effect in whole sample (1 test) or sub-groups (2 tests)	Long term whilst intervention ongoing:<br>No effect in whole sample (1 test) or sub-groups (2 tests)	Long term whilst intervention ongoing:<br>No effect in whole sample (1 test) or sub-groups (2 tests)	Long term whilst intervention ongoing:<br>No effect in whole sample (5 tests) or sub-groups (10 tests)	-

For peer review only

Study<br>Intensity of intervention	Motor development outcomes	Language development outcomes	Cognitive development outcomes	Social and emotional wellbeing outcomes	Overall child development
**4b. Studies comparing two interventions**					
**Unclear risk**					
**Beeghly 1995**<br>**2 low intensity intvns**	Short term post-intervention:<br>No effect in whole sample (1 test) or sub-groups (1 test)	-	Short term post-intervention:<br>No effect in whole sample (1 test) or sub-groups (1 test)	-	-
**Lobo 2012**<br>**2 moderate intensity interventions**<br>(*13*)	Short term whilst intervention ongoing:<br>No effect in 4 & positive effect in 4 of 8 tests<br>Short to medium term post-intervention:<br>Positive effect in 5 of 5 tests	-	-	-	-
**Doyle 2011**<br>**2 intvns: high** & **moderate**<br>(*141*)	Short, medium and long term whilst intervention ongoing:<br>No effect (20 tests)	Short, medium and long term whilst intervention ongoing:<br>No effect (21 tests)	Short, and medium term whilst intervention ongoing:<br>No effect (6 tests)<br>Long term whilst intervention ongoing:<br>No effect in 9 & positive effect in 3 of 12 tests	Short, and medium term whilst intervention ongoing:<br>No effect (16 tests)<br>Long term whilst intervention ongoing:<br>No effect in 61 & positive effect in 1 of 62 tests	Short, medium and long term whilst intervention ongoing:<br>No effect (4 tests)
**Johnston 2006**<br>**2 high intensity intvns**<br>(*7*)	-	Long term whilst intervention ongoing:<br>No effect in 2 & positive effect in 2 of 4 tests	-	Long term whilst intervention ongoing:<br>No effect in 2 & negative effect in 1 of 3 tests	-
**Landry 2006**<br>**2 high intensity intvns**<br>(*6*)	-	Short term post-intervention:<br>Positive effect in 2 of 2 tests	Short term post-intervention:<br>Positive effect (1 test)	Short term post-intervention:<br>No effect in 3 & positive effect in 3 of 6 tests	-

For peer review only

Figure 1: PRISMA flow diagram

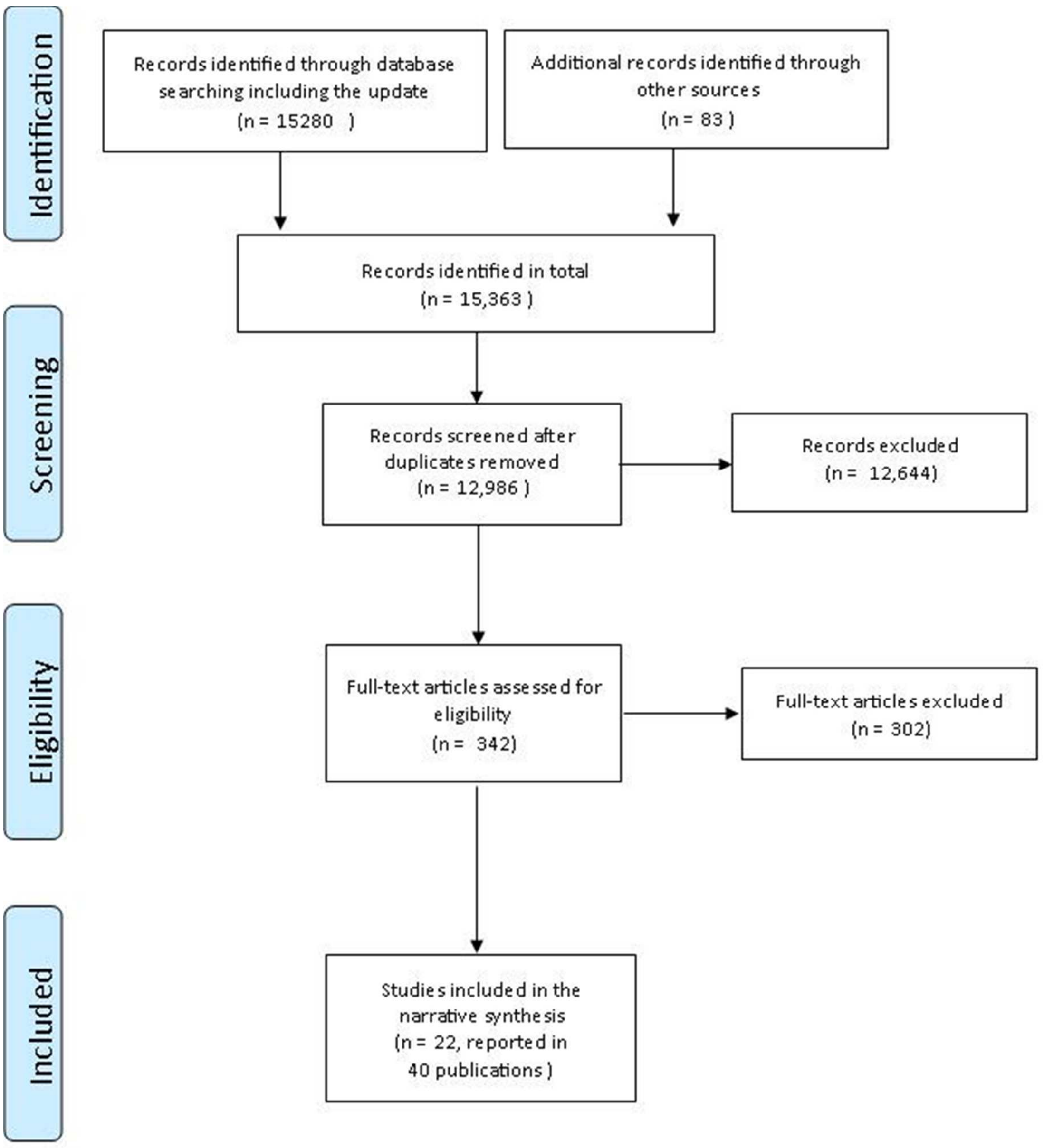

Reason for exclusion at full text screening:
Ongoing study, n = 3
Quasi-experimental (control group but no randomisation), n = 10
Pre-post test comparison only, n = 5
Not a primary study (reviews, editorials, programme descriptions), n = 67
Not conducted in a high income country, n = 3
Intervention delivered in childcare settings, n = 14
Targeted programme (child factors), n = 37
Targeted programme (adult or family risk factors), n = 88
Mean age of children at intervention >24 months, n = 53
Mean age of children at outcome > 36 months, n = 5
No child development outcomes, n = 17

Figure 2: Risk of bias graph: review authors' judgements about each risk of bias item presented as percentages across all included studies

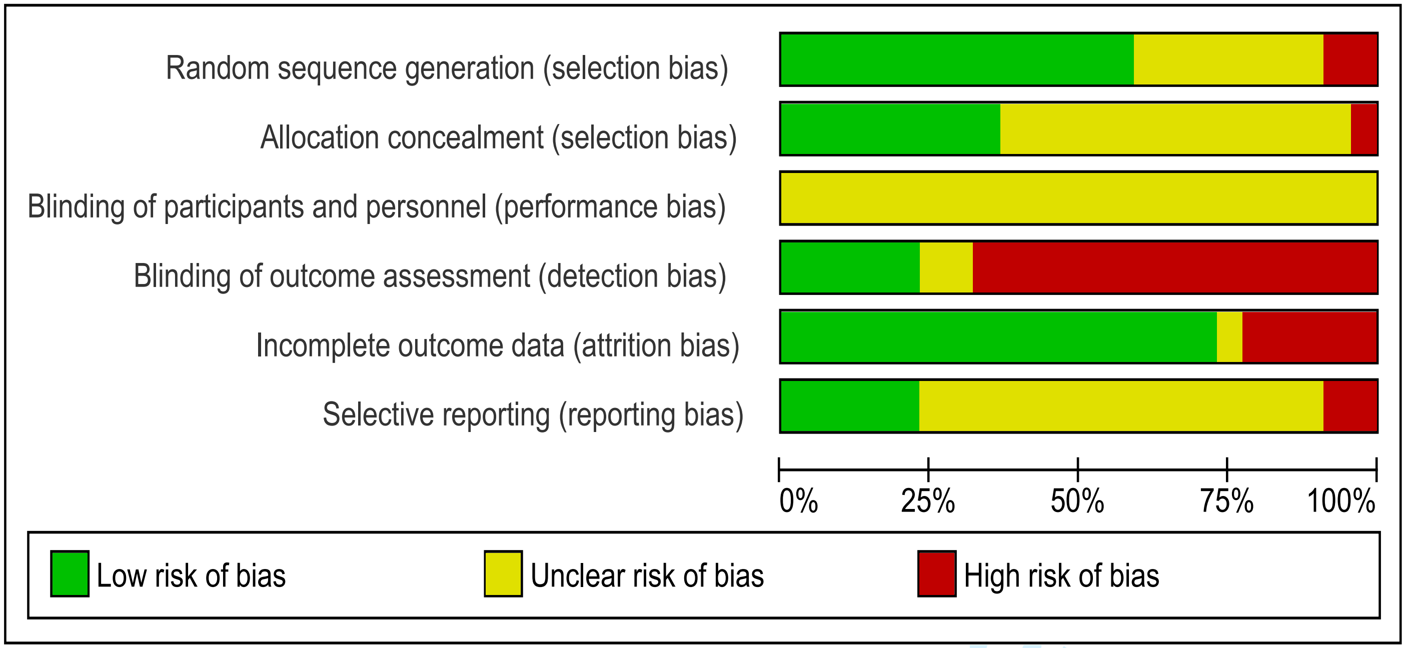

Figure 3: Risk of bias summary: Review authors' judgements about each risk of bias item for each included study

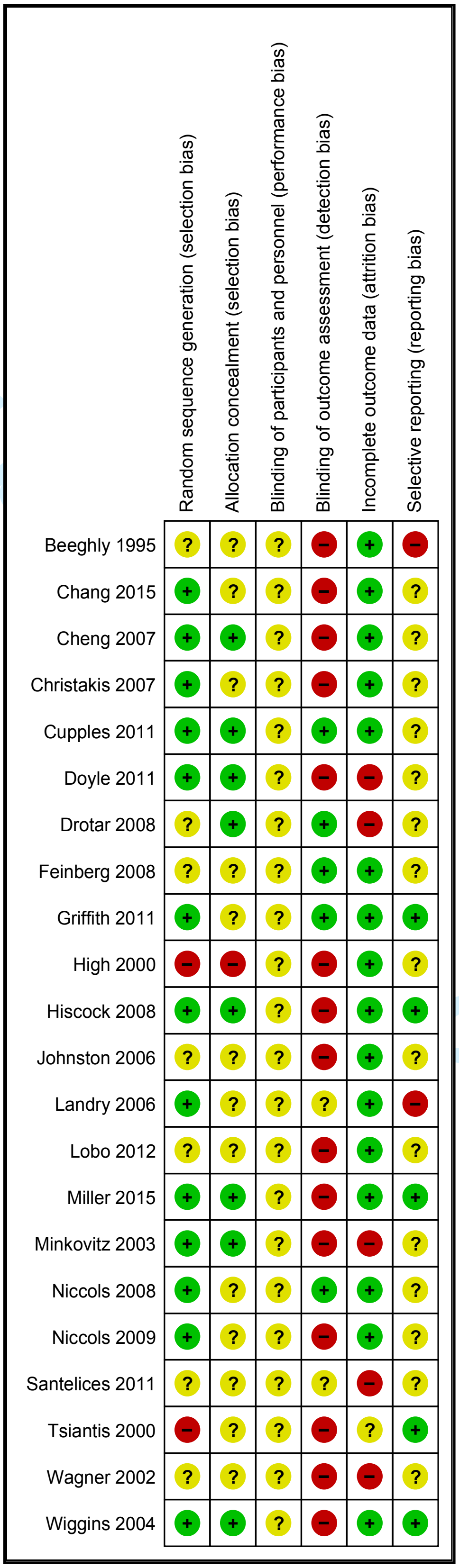

For peer review only

Web Appendices

A. Medline search strategy

B. List of programme and organisation websites and journals for electronic journal of contents (eTOC) search

C. Full narrative summary of results

D. Summary of results, stratified by other characteristics

For peer review only

Web Appendix A. Medline search strategy

Database: Ovid MEDLINE(R) without Revisions
Search Strategy:
----
1 exp Health Personnel/
2 exp Nurses, Community Health/
3 health professional.tw.
4 exp Physician-Patient Relations/
5 (nurse* or physician* or doctor* or midwife* or midwive* or health visitor*).tw.
6 ((health* or medical or nurs* or hospital) adj (personnel or provider* or professional* or practitioner* or worker* or staff or specialist*)).tw.
7 (general practitioner* or gp or gps or clinician* or hospitalist* or psychologist* or social worker* or welfare worker*).tw.
8 ("Mothers aide" or "mothers help" or "maternity nurse" or "public health nurs*" or "specialist public health nurse" or "nursery nurse" or nanny or "early year educator*" or childcarer).tw.
9 ((paediatric or pediatric) adj clinic*).tw.
10 ((baby or child) adj clinic*).tw.
11 Primary Health Care/
12 exp House Calls/
13 home visit*.tw.
14 day care.mp.
15 clinic visit.tw.
16 surgery visit.tw.
17 clinician-patient interaction.tw.
18 early intervention*.tw.
19 ((parent* or mother*1 or father*1 or mum or dad or family or families) adj (group or support or training or education)).tw.
20 ((carer* or caregiver) adj (group or support or training or education)).tw.
21 ("early years staff" or "early years health professional*" or "early years professional*" or "early years educator*" or "early years practitioner*").tw.
22 (preschool educator or nursery staff).tw
23 parent educator*.tw.
24 (Family support or family support worker or support worker).tw.
25 (Community health worker or lay health worker).tw
26 Public health staff.tw.
27 Breakthrough to Literacy.mp
28 Family Foundations.mp.
29 Incredible Years.mp.
30 Parent Effectiveness Training.mp.
31 Primary Care Triple P.mp.
32 Triple P.mp.
33 Born to Learn.mp.
34 Mellow Parenting.mp.
35 "The Family Partnership Model".mp.
36 The Solihull Approach.mp.
37 "Parent child interaction therapy".mp.
38 "Nurturing Programme".mp.
39 "The Family Partnership Model".mp.
40 "Peers Early Education Partnership".mp.
41 "Webster Stratton Incredible Years".mp.

For peer review only

42 "Community Mothers Program".mp.
43 "Social Support and Family Health".mp.
44 "Limerick Lullaby".mp.
45 or/1-44 (ENHANCED PROFESSIONAL CONTACT TERMS)
46 exp Child Development/
47 exp language development/
48 Object Attachment/
49 Concept Formation/
50 Child Behavior Disorders/
51 ((child or infant) adj development).tw.
52 ((education* or cognitive or mental or language or physical or motor or social or emotional) adj3 (progress or development)).tw.
53 ((physical or emotional or social) adj (wellbeing or health)).tw.
54 (faltering growth or growth faltering or stunting).tw.
55 early child development.tw.
56 ((vocabulary or speech or language) adj development).tw.
57 Child behavio?r.tw.
58 Language/
59 Object Attachment/
60 social competence.tw.
61 social interaction.tw.
62 school readiness.tw.
63 or/46-62 (CHILD DEVELOPMENT TERMS)
64 45 and 63

For peer review only

Web Appendix B. List of programme and organisation websites and journals for electronic journal of contents (eTOC) search

Table B1. List of relevant programmes and organisations for which websites were searched

**Programme name**
1. Al's Pals
2. Breakthrough to Literacy
3. Bright Beginnings
4. Circle of Security
5. Community Mothers Program
6. Curiosity Corner
7. Developing Everyone's Learning and Thinking Abilities (DELTA)
8. Eager and Able to Learn
9. Family Check-Up
10. Family Foundations
11. Family Literacy programme
12. Family Nurturing Network: Family Connections
13. Family Partnership Model
14. Fun and Families
15. HIPPY
16. Holding Hands
17. I Can Problem Solve
18. Incredible Years
19. Infant Behavioral Assessment and Intervention Programme
20. Let's Begin with the Letter People
21. Let's Play in Tandem
22. LifeStart ("Growing Child")
23. Limerick Lullaby
24. Living with Children
25. Mellow Parenting
26. Minding the Baby
27. Noughts to Sixes (From Pram to Primary School), Family Caring Trust
28. Nurturing Programme
29. Parents as First Teachers or Parents as Teachers (Born to Learn)
30. Parent–child interaction therapy
31. Parent Effectiveness Training
32. Parenting Positively
33. Parents Plus Early Years
34. Peers Early Education Partnership
35. Preparing for Life<br>Child Development Initiative
36. Ready, Set, Leap!
37. Ready, Steady, Grow
38. Rock-a- Bye
39. Sheffield Raising Early Achievement in Literacy (REAL) project
40. Solihull Approach

For peer review only

41. Social Support and Family Health
42. Strengthening Families, Strengthening Communities
43. Supporting Parents on Kids Education (SPOKES)
**Organisation name**
1. Basic Skills Agency
2. Department of Education, UK
3. Early Childhood Australia
4. Education Endowment Fund
5. Early Intervention Foundation
6. European Commission (Education and Training)
7. European Early Childhood Education Research Association
8. Harvard Developing Child Programme
9. National Health Service (NHS)
10. National Institute for Health and Care Excellence (NICE)
11. National Institute of Health (USA)
12. Raising Children Network (Australia)
13. Sure Start/Flying Start
14. World Bank Early Child Development
15. World Health Organisation

B2. Journals included in electronic table of contents (eTOC) search

Archives of Diseases in Childhood

Child: Care, Health and Development

Child Development

Early Child Development and Care

Early Childhood Research Quarterly

Infant Mental Health Journal

Journal of Early Childhood Research

Pediatrics

For peer review only

Web Appendix C. Full narrative summary of results

Table C1: Motor Development Outcomes, stratified by timing of outcome measurement

Motor development outcomes		
**Studies comparing one intervention with usual care**		
**Study**	**Measure**	**Results**
Chang 2015 (Short term post intervention)	Griffith Mental Development Scale	**No difference in mean hand/eye coordination score** between intervention and control (mean 95.95, SD 10.25 compared with mean 94.62, SD 9.89); adjusted mean difference 0.73, 95% CI -0.83, 2.29; p value for comparison = 0.89)
Cupples 2011 (Immediately post intervention)	Bayley Scales of Infant Development (Psychomotor Development Index)	**No difference in mean scores** between intervention and control for PDI (MD [non-imputed] 1.64, 95% CI -0.94-4.21, p=0.21)
Wagner 2001 (Long term whilst programme ongoing)	Development Profile II	**No difference in mean physical development scores** between intervention and control in total sample (mean in intervention 2.9, mean in control 3.0, p>=0.05), or in samples stratified by income level (p>=0.05 for all tests)
**Motor development outcomes**		
**Studies comparing two interventions**		
**Study**	**Measure**	**Results**
Beeghly 1995 (Short term post-intervention)	Bayley Scales of Infant Development (Psychomotor Development Index)	**No difference in mean scores** for PDI in infant-centred (mean 109.77, SD 13.05) and mother-centred (mean 108.31, SD 12.43) groups at 3 months post-intervention (when children were 4 months, p>=0.05).
Doyle 2011 (Short term whilst intervention is ongoing)	Ages and Stages Questionnaire	**No differences** between high and low intensity programme using mean scores or cut-offs for 4 comparisons: gross motor scores, gross motor cut-off, fine motor scores, fine motor cut-offs (p values adjusted for multiple testing)
Doyle 2011 (Medium term whilst intervention is ongoing)	Ages and Stages Questionnaire	**No differences** between high and low intensity programme using mean scores or cut-offs for 4 comparisons: gross motor scores, gross motor cut-off, fine motor scores, fine motor cut-offs (p values adjusted for multiple testing)
Doyle 2011 (Long term whilst intervention is ongoing)	Ages and Stages Questionnaire (measured at 18, 24 and 36 months)	**No differences** between high and low intensity programme using mean scores or cut-offs for 4 comparisons at each time point: gross motor scores, gross motor cut-off, fine motor scores, fine motor cut-offs at any of the time points (p values adjusted for multiple testing)
Lobo 2012 (Short term whilst intervention ongoing)	Alberta Infant Motor Scale	**No difference for change in total mean scores** between in handling group compared with social experience group at 2-3 months (scores not given, p>=0.05).<br>**Greater change in mean score** for **prone** subscale in handling group compared with social experience group at 2-3 months of age (Cohen's correlation coefficient **r = -0.45, p<0.01**).<br>**No difference** for **supine** and **sitting** subscale at 2-3 months (scores not given, p>=0.05).<br>**Greater change in total mean score** in handling group compared with social experience group at 5 months of age (Cohen's correlation coefficient r = -0.46, p<0.01).<br>**Greater change in total mean score** for **prone** (r=-0.45, p<=0.01) and **sitting** (r=-0.34, p<=0.05) subscale in

For peer review only - http://bmjopen.bmj.com/site/about/guidelines.xhtml

		handling group at 5 months of age.<br>**No difference** for **supine** subscale at 5 months of age (scores not given, p>=0.05).
Lobo 2012<br>(Short term post-intevention)	Parent Milestone Report Form	Intervention group children reached for midlife objects before control children (no data provided, p<=0.05)<br>Intervention group children transferred objects from one hand to another **2.5 weeks earlier** (p<=0.05)<br>Intervention group children crept/crawled **5 weeks earlier** (p<=0.05)<br>Intervention group children walked supported **2.5 weeks earlier** (p<=0.05)<br>Intervention group children walked alone **6 weeks earlier** (p<=0.05)

For peer review only

Table C2: Language Development Outcomes

Language development outcomes		
**Studies comparing one intervention with usual care**		
**Study**	**Measure**	**Results**
Chang 2015 (Short term post intervention)	Griffith Mental Development Scale<br><br>MacArthur Bates Communicative Development Inventory (Short version, CDI)	**No difference in mean language scores** (Griffith scale) between intervention and control (mean 99.78, SD 14.01 compared with mean 99.90, SD 13.58); adjusted mean difference -0.54, 95% CI -2.81, 1.75; p value for comparison = 0.89)<br>**No difference in mean vocabulary scores (CDI)** between intervention and control (mean 38.00, SD 18.42 compared with mean 39.57, SD 20.49); adjusted mean difference -0.94, 95% CI -3.49, 1.61; p value for comparison = 0.89)
Christakis 2007 (Short term post intervention)	MacArthur Communicative Development Inventory	Whole sample: **No difference** between intervention and control (linear regression results: β for intervention variable raw score = 7.52, 95% CI -0.66-15.7; β for intervention variable percentile score = 8.40, 95% CI -3.00-19.90) at approx 4 months post intervention (children were 24 to 36 months).<br>Low and middle income sample: **Higher scores** in the intervention group (linear regression results: β for intervention variable raw score = 12.40, 95% CI 3.0-21.8, p=0.01; β for intervention variable percentile score = 14.94, 95% CI 1.37-27.60, p=0.03) at approx 4 months post intervention (children were 24 to 36 months).
Drotar 2008 (Long term whilst intervention ongoing)	Systematic Analysis of Language Transcripts (SALT)	**No difference in mean scores** between intervention and control when children were 36 months (MD -3.91, 95% CI -14.32-6.77, p=0.48).
High 2000 (Short term post-intervention)	Modified MacArthur Communicative Development Inventory (short form)	**Higher mean scores** in intervention group for receptive vocabulary in total sample (p values between 0.008 and 0.003 in 3 tests), but no difference on expressive vocabulary (3 tests)<br>**No difference** in mean scores in receptive and expressive vocabulary between intervention and control groups in children aged 13-17 months at assessment (6 sub-group tests).<br>**Higher mean scores** in intervention group for receptive and expressive vocabulary in children aged 18-25 months at assessment (**p values between 0.01 and 0.003 in 6 sub-group tests**)
Tsiantis 2000 (Immediately post-intervention)	Bzoch-league Receptive Expressive Emergent Language Scale (REEL)	**Better performance in observed language** in comparison group children (no scores presented, but **p<0.0001**)<br>Results analysed by country, but not presented in paper.
Wagner 2002 (Long term whilst intervention is ongoing)	Development Profile II (DPII)	**No difference in mean scores** between intervention and control in communication development scores in whole sample (mean in intervention 4.9, mean in control 4.5, p>=0.05), or when stratified by income group.
Wiggins 2004 (Short term post-intervention)	Question on whether the mother has any worries about their child's speech	**Fewer mothers in the SHV group** expressed a worry about their child's speech than in the control group (risk ratio 0.46, 95% CI 0.23, 0.93)<br>**No difference** in the number of number of mothers expressing worries about speech between CGS and control (risk ratio 1.22, 95% CI 0.78, 1.92)

For peer review only

Language development outcomes		
**Studies comparing two interventions**		
**Study**	**Measure**	**Results**
Doyle 2011 (Short term whilst intervention is ongoing)	Ages and Stages Questionnaire	**No differences** between high and low intensity programme using mean scores or cut-offs for 2 comparisons: communication scores, communication cut-off (p values adjusted for multiple testing)
Doyle 2011 (Medium term whilst intervention is ongoing)	Ages and Stages Questionnaire<br><br>MacArthur-Bates Communicative Development Inventory	**No differences** between high and low intensity programme using mean scores or cut-offs for 2 comparisons: communication scores, communication cut-off (p values adjusted for multiple testing).<br>**No differences** between high and low intensity programme using mean scores for 4 sub-scores: first signs of understanding, words understood, words produced, first communicative gestures (p values adjusted for multiple testing)
Doyle 2011 (Long term whilst intervention is ongoing)	Ages and Stages Questionnaire (measured at 18, 24 and 36 months)<br>MacArthur-Bates Communicative Development Inventory (measured at 18 and 24 months)	**No differences** between high and low intensity programme using mean scores or cut-offs for 2 comparisons at any of the time points: communication scores, communication cut-off (p values adjusted for multiple testing).<br>**No differences** between high and low intensity programme using mean scores for 5 sub-scores at 18 months: first signs of understanding, can combine words, first communicative gestures, words produced, words understood, and 2 sub-scores at 24 months: can combine words, words produced (p values adjusted for multiple testing)
Johnston 2006 (Long term post-intervention)	Sub-scales of MacArthur Communicative Development Inventory	**Higher mean vocabulary score** in HS + PP group than HS only group at 24 months (aMD 4.08; 95% CI 0.69, 7.47; $p<0.05$).<br>**Higher percentage** combining >=2 words in HS + PP group than HS only group (rate ratio 1.05; 95% CI 1.02, 1.08; $p<0.05$).<br>**No difference** in length of child's longest phrase or variety of word endings between HS+PP and HS only group.
Landry 2006 (Short term post-intervention)	Coding of videotaped interactions between child and mother, and child with examiner	**Higher word usage scores** observed in the PALS group than the DAS group in interactions with mother (p=0.02) and examiner (p=0.02) at 3 months post-intervention when child was 13 months.

For peer review only

Table C3: Cognitive Development Outcomes

Cognitive development outcomes		
**Studies comparing one intervention with usual care**		
**Study**	**Measure**	**Results**
Chang 2015 (Short term post intervention)	Griffith Mental Development Scale	No effect in intervention group in unadjusted results (mean 92.69, SD 11.65 compared with mean 89.52, SD 10.56); unadjusted mean difference 3.12, 95% CI 0.86, 5.39; p value for comparison = 0.07); **higher mean cognitive scores (Griffith scale)** for adjusted mean difference (aMD 3.09, 95% CI 1.31, 4.87, p value = 0.007)
Cupples 2011 (Immedlately post-intervention)	Bayley Scales of Infant Development (Mental Development Index, MDI)	**No difference in mean scores** between intervention and control when children were 12 months old (MD [non-imputed] -0.81, 95% CI -2.81-1.16, p=0.42)
Drotar 2008 (Short-medium term whilst intervention ongoing)	Bayley Scales of Infant Development (Mental Development Index, MDI)<br>Assessor rating of mastery motivation (persistence, pleasure and competence with a play task)	**No difference in mean scores** between intervention and control when children were 18 months (MD 0.68, 95% CI -1.15-2.76, p=0.42).<br>**No difference in mean scores** between intervention and control for any of the 3 tasks when children were 18 months.
Drotar 2008 (Long term whilst intervention ongoing)	Bayley Scales of Infant Development (Mental Development Index, MDI)<br><br>Assessor rating of mastery motivation (persistence, pleasure and competence with a play task)<br><br><br><br>Kaufman Assessment Battery<br><br>Bracken Basic Concept Scale<br><br>Test of Early Reading Ability-2 (TERA-2)	**No difference in mean scores** between intervention and control for MDI (MD -0.78, 95% CI -3.63-2.32, p=0.67) when children were 24 months. **Higher mean score** in intervention than control in low SES sub-group (p for interaction < 0.003, mean for intervention 89.67 (SD16.20) & mean for control 82.16 (SD 17.24, p < 0.01).<br>**No difference in mean scores** between intervention and control for any of the 3 tasks when children were 24 months. **Higher mean score** for task competence in intervention group for low SES sub-group (p for interaction not shown, mean for intervention 584.49 (SD 37.97) & mean for control 556.21 (SD 57.18), p < 0.02.<br>**No difference** between intervention and control in 2 of 3 tests of mastery motivation (persistence and pleasure) when children were 36 months; **higher mean scores** in intervention group for task competence (MD 6.24, 95% CI 0.16-14.76, p=0.05).<br>**No difference in mean scores** between intervention and control for sequential processing, simultaneous processing or mental processing when children were 36 months (e.g. for mental processing MD 0.23, 95% CI -3.33-2.91, p=0.89).<br>**No difference in mean scores** between intervention and control when children were 36 months (MD 1.24, 95% CI -2.24-4.47, p=0.51).<br>**No difference in mean scores** between intervention and control when children were 36 months (MD 0.54, 95% CI -1.70-2.47, p=0.72).
Miller 2015 (Long term whilst intervention ongoing)	British Ability Scale (BASII)	**No difference in mean scores** between intervention and control on cognitive development (mean in intervention group -0.046 (SD 1.007) and mean in control group 0.018 (SD 0.994), Hedges g effect size -0.634, 95% CI -0.277, 0.149, p=0.557)
Wagner 2001 (Long term whilst intervention ongoing)	Development Profile II (DPII)	**No difference in mean scores** between intervention and control in cognitive development scores (mean in intervention 2.2, mean in control 2.4, p>=0.05), or in samples stratified by income.

For peer review only - http://bmjopen.bmj.com/site/about/guidelines.xhtml

Cognitive development outcomes		
**Studies comparing two interventions**		
**Study**	**Measure**	**Results**
Beeghly 1995 (Short term post intervention)	Bayley Scales of Infant Development (Mental Development Index, MDI)	**No difference in mean scores** in infant-centred (mean 109.38, SD 12.88) or mother-centred (mean 107.83, SD 10.69) groups at 3 months post-intervention (when children were 4 months, p>=0.05).
Doyle 2011 (Short term whilst intervention is ongoing)	Ages and Stages Questionnaire	**No differences** between high and low intensity programme using mean scores or cut-offs for 2 comparisons: problem solving score, problem solving cut-off (p values adjusted for multiple testing)
Doyle 2011 (Medium term whilst intervention is ongoing)	Ages and Stages Questionnaire<br><br>Development Profile 3	**No differences** between high and low intensity programme using mean scores or cut-offs (2 comparisons): problem solving score, problem solving cut-off (p values adjusted for multiple testing).<br>**No difference** in mean scores or for the proportion above the average cut-off (2 comparisons, p values not adjusted for multiple testing).
Doyle 2011 (Long term whilst intervention is ongoing)	Ages and Stages Questionnaire (measured at 18, 24 and 36 months)<br>Development Profile 3 (measured at 18, 24 and 36 months)	**No differences** between high and low intensity programme using mean scores or cut-offs for 2 comparisons at any of the time points: problem solving score, problem solving cut-off (p values adjusted for multiple testing).<br>At 18 months, **no difference** in mean scores or for the proportion above the average cut-off (p values not adjusted for multiple testing).<br>At 24 months, **no difference for the proportion above the average cut-off** (p values **not** adjusted for multiple testing); **higher mean standardised score** in high intensity group (mean 116.51 [SD 13.30] vs mean 112.57 [S 16.12], p<0.05, **not** adjusted for multiple testing).<br>At 36 months, **higher mean standardised score** in high intensity group (mean 114.66 [SD 14.39] vs mean 109.82 [SD 14.02], p<0.05, **not** adjusted for multiple testing) and **higher proportion above average cut-off** (0.53 [SD0.50] vs 0.36 [SD 0.48], p<0.05, **not** adjusted for multiple testing)
Landry 2006 (Short term post-intervention)	Coding of videotaped interactions between child and mother, and child with examiner	**Higher scores** for independent goal-directed play in the PALS group than in the DAS group (p=0.04) at 3 months post-intervention when child was 13 months.

For peer review only

Table C4: Social and Emotional Wellbeing Outcomes

Social and emotional wellbeing outcomes		
**Studies comparing one intervention with usual care**		
**Study**	**Measure**	**Results**
Cheng 2007 (Short term post-intervention)	Parent-Infant Relationship Global Assessment Scale	Of 37 mother-infant pairs with a "disturbed" relationship at baseline, a **higher percentage** of intervention group was classified as having an "adapted" relationship (63% [12 pairs] vs 28% [5 pairs], p=0.039) at 1 month post-intervention (when children were 10 months).<br>Of 48 mother-infant pairs with an "adapted" relationship at baseline, **no difference in percentage** of intervention vs control classified as "disturbed" (26% [7 pairs] vs 29% [8 pairs], p=0.821) at 1 month post-intervention (when children were 10 months).
Cheng 2007 (Long term post-intervention)	Child Behaviour Checklist	**No difference** between intervention and control in the odds of being in the upper quartile of the CBCL scores (total score aOR 0.40, 95% CI 0.12-1.43; internalizing score aOR0.60, 95% CI 0.23-1.68; externalizing score aOR 0.50, 95% CI 0.19-1.54) at 15 months post-intervention (when children were 24 months).
Christakis 2007 (Short term post-intervention)	Hyperactivity sub-domain of the Child Behavior Checklist	**No difference** in attention problems (score in 90th percentile or higher) between intervention and control in the whole sample (aOR 0.49, 95% CI 0.13-1.81) or low and middle income sample (aOR 0.48, 95% CI 0.12, 1.83) at approx 4 months post intervention (children were 24 to 36 months).
Cupples 2011 (Immediately post-intervention)	Bayley Scales of Infant Development (BSID-II)	**No difference in the mean scores** between intervention and comparison group (MD [non-imputed] -0.88, 95% CI -4.59-2.81, p=0.64)<br>**No difference in the mean scores** between intervention and control for the 3 sub-scores of the BSID-II behavioural scale.
Drotar 2008 (Short-medium term whilst intervention ongoing)	Bayley Behaviour Rating Scale	**No difference in mean scores** between intervention and control when children were 12 months (MD -0.33, 95% CI -1.95-1.64, p=0.87).
Drotar 2008 (Medium-long term whilst intervention ongoing)	Q-Sort measure of Security of Attachment	**No difference** between intervention and control when children were 18 months (no numerical data presented).
Drotar 2008 (Long term whilst intervention ongoing)	Bayley Behaviour Rating Scale<br><br>Child Behaviour Rating Scale<br>Social Skills Rating System	**No difference in mean scores** between intervention and control when children were 24 months (MD 0.43, 95% CI -1.72-3.15, p=0.56) or 36 months (MD -1.64, 95% CI -3.99-0.83, p=0.20).<br>**No difference** between intervention and control when children were 24 months (no numerical data presented).<br>**No difference in mean scores** between intervention and control when children were 36 months for parent. reported social skills (MD 3.33, 95% CI -0.45-5.90, p=0.09) or teacher reported social skills (MD 2.07, 95% CI -3.19-7.15, p=0.45).
Feinberg 2008 (Immediately post-intervention)	Infant Behaviour Questionnaire	**No difference** in parent-child dysfunctional interaction (beta calculated using linear regression) in intervention versus control group according to mother's report (beta -0.137, Cohen's d 0.34, p<0.10), but **intervention fathers had lower dysfunctional interaction scores** (beta = -0.291, Cohen's d = 0.70, p<0.05).<br>**No difference** in infant soothability score (beta calculated using linear regression) in intervention versus control

For peer review only

		group according to mother's report (beta 0.021, Cohen's d not calculated, p>=0.05), but **intervention children were easier to soothe** (had a higher score than control) according to father's reports (beta = 0.312, Cohen's d = 0.35, p<0.05).<br>Score for duration of attention **higher in intervention versus control infants** according to mother's report (F=4.33, Cohen's d = 0.34, p<0.05). Fathers reports of this variable not given in paper.
Feinberg 2008 (Short term post-intervention)	Observer coding of videotaped behaviours (self-soothing and sustained attention at play)	**More self-soothing behaviours** observed in intervention versus control group when infants were 12 months of age (6 months post-intervention, beta = 0.30, Cohen's d = 0.46, p<0.05).<br>**No difference** in sustained attention noted by coders between intervention and control children (beta = 0.05, Cohen's d = 0.08, p>=0.05).
Feinberg 2009 (Short term post-intervention)	Child Behaviour Checklist	Mean scores and SDs for each of the 7 comparisons presented but no accompanying P values; t tests conducted in Stata (assuming 81% follow-up in each group) indicate **no significant differences**.<br>Written results suggest that the model based estimates of intervention effects were significant but only when an interaction with child gender was included in the model - the presentation of these results is unclear and there appears to be an inconsistency between the results presented in the table and those in the text.
Griffith 2011 (Short term post-intervention)	Dyadic Parent-Child Interaction coding system	**No difference in mean scores** between intervention and control for child deviance behaviours in the parent-child interaction observations (negative physical behaviours, destructive behaviours, small talk; mean in intervention 3.18 (SD 3.40) and control 2.97 (SD 3.43), no p value for this comparison) at 3 months or less post-intervention.
Hiscock 2008 (Short term post-intervention)	Child Behaviour Checklist	**No difference in mean scores** between intervention and control for externalising (aMD 0.16, 95% CI -1.01-1.33, p=0.79) or internalising (aMD 0.49, 95% CI -0.20-1.18, p=0.16) behaviours 3 months post-intervention (when children were 18 months).
Hiscock 2008 (Medium term post-intervention)	Child Behaviour Checklist	**No difference in mean scores** between intervention and control for externalising (aMD -0.79, 95% CI -2.27-0.69, p=0.30) or internalising (aMD 0.19, 95% CI -0.77-1.15, p=0.70) behaviours 9 months post-intervention (when children were 24 months).
Hiscock 2008 (Long term post-intervention)	Child Behaviour Checklist	**No difference in mean scores** between intervention and control for externalising (aMD -0.8, 95% CI -2.2-0.6, p=0.26) or internalising (aMD -0.6, 95% CI -1.5-0.3, p=0.19) behaviours 21 months post-intervention (when children were 36 months).
Miller 2015 (Long term whilst intervention is ongoing)	Bayley Social-Emotional and Adaptive Behaviour Questionnaire	**No difference in mean scores** between intervention and control on socio-emotional development (mean in intervention group 0.087 (SD 0.987) and mean in control group -0.108 (SD 1.016, Hedges g effect size 0.195 (95% CI -0.046, 0.437, p=0.114)
Minkovitz 2003 (Long term whilst intervention is ongoing)	Subscales of Child Behaviour Checklist	**No difference in mean scores** for 3 sub-scores (aggressive behaviour, anxious or depressed, sleep problems) between intervention and control group. For example, for aggressive behaviour, the adjusted mean difference between intervention and control was 0.23 (95% CI -0.29, 0.79, p=0.16, higher scores indicate higher reporting of aggressive behaviour)
Niccols 2008 (Immediately post-intervention)	Attachment Q-set (completed by observers)	**No difference in the change in scores between pre-test and post-test** between intervention and comparison group in the intention-to-treat analysis (no numbers presented, text states "no significant differences")
Niccols 2008 (Short term post-intervention)	Attachment Q-set (completed by observers)	**No difference in the change in scores between pre-test and post-test** between intervention and comparison group in the intention-to-treat analysis 6 months post-intervention (no numbers presented, text states "no significant differences")

For peer review only

Niccols 2009 (Immediately post-intervention)	Eyberg Child Behavior Inventory<br><br>Observed parent-child interaction	**No difference in mean scores** between intervention and control for child behaviour problems (mean 48.87 (SD 6.22) vs mean score 50.68 (SD 7.23), p>=0.05)<br>**Higher mean number of observed** positive child behaviours in intervention group (mean 21.10 (SD 8.59) vs mean score 16.27 (SD 9.25), p<0.05), and **higher ratio** of child compliance to maternal requests in intervention group (mean 0.62 (SD 0.19) vs mean score 0.53 (SD 0.19), p<0.05).<br>**No difference in mean number** of observed negative behaviours in intervention versus control (mean 17.81 (SD 8.84) vs mean score 22.29 (SD 8.85), p>=0.05).
Niccols 2009 (Short term post-intervention)	Eyberg Child Behavior Inventory<br><br>Observed parent-child interaction	**Lower mean score** for child behaviour problems in intervention versus control (mean 48.87 (SD 6.22) vs mean score 50.68 (SD 7.23), p<0.017).<br>**Higher mean number** of observed positive child behaviours in intervention group than control (mean 22.61 (SD 8.97) vs mean score 16.69 (SD 8.40), p<0.05), and **higher ratio of** observed compliance to maternal requests in intervention group (mean 0.61 (SD 0.19) vs mean score 0.53 (SD 0.19), p<0.05).<br>**No difference** in mean number of observed negative behaviours between intervention and control (mean 18.86 (SD 7.53) vs mean score 21.29 (SD 7.78), p>=0.05)
Santelices 2010 (Immediately post-intervention)	Observed Strange Situation Procedure	**No difference** between intervention and control in infant attachment security (% classed as "secure" in intervention 72.1%, % classed as "secure" in control 55.2%, p=0.139)
Tsiantis 2000 (Short term whilst intervention is ongoing)	Bayley Scale of Psychosocial Development<br>Observed mother-child interaction	**No difference in mean scores** between intervention and control (mean 7.03 compared with mean 7.04, no numbers in analysis or statistics presented).<br>**Mother-child interaction rated as better** in intervention group in one of the four countries (no numbers in analysis, or scores presented, p for sub-group effect= 0.02).
Tsiantis 2000 (Medium term whilst intervention is ongoing)	Bayley Scale of Psychosocial Development<br>Observed mother-child interaction	**No difference in mean scores** between intervention and control (mean 6.82 compared with mean 7.06, no numbers in analysis or statistics presented)<br>**Mother-child interaction rated as better in comparison group** when all countries' data combined (no numbers in analysis presented, scores presented but not clear what they represent, p = 0.02)
Tsiantis 2000 (Long term whilst intervention is ongoing)	Bates Infant Characteristics Questionnaire	**No difference in total mean scores** in full sample between intervention and control (no numbers in analysis, scores or statistics presented for mean scores); 2 of 7 individual characteristic scores higher in intervention than control in full sample ("sober" p = 0.02, "factor 7" p <0.06).
Wagner 2001 (Long term whilst intervention is ongoing)	Nursing Child Assessment Satellite Training teaching scale (NCAST) used to observe mother-child interactions<br>Measure = Development Profile II (DPII)<br><br>Adaptive Social Behaviour Inventory	**No difference in mean scores** for observed mother-child interaction between intervention and control in total sample (2 comparisons), or when results stratified by income group (p>=0.05)<br><br>**No difference in mean self help or social development scores** between intervention and control in total sample, or when results stratified by income group (p>=0.05)<br>**No difference in mean scores** between intervention and control in total sample, or when results stratified by income group (p>=0.05)
Wiggins 2004 (Short term post-intervention)	Question on whether the mother has any worries about their child's behaviour	**No difference** in the number of number of mothers expressing worries about behaviour between supportive health visiting and control groups (risk ratio 0.74, 95% CI 0.37, 1.47)<br>**No difference** in the number of number of mothers expressing worries about behaviour between community group support and control groups (risk ratio 0.94, 95% CI 0.51, 1.73)

For peer review only

Social and emotional wellbeing outcomes		
**Studies comparing two interventions**		
**Study**	**Measure**	**Results**
Doyle 2011 (Short term whilst intervention is ongoing)	Ages and Stages Questionnaire<br><br>Child Characteristic Questionnaire	**No differences** between high and low intensity programme using mean scores or cut-offs for 4 comparisons: personal social score, personal social cut-off, social-emotional score, social-emotional cut-off (p values adjusted for multiple testing).<br>**No difference** between high and low intensity programme for score based on a 6 point scale of difficult temperament (p value adjusted for multiple testing).
Doyle 2011 (Medium term whilst intervention is ongoing)	Ages and Stages Questionnaire<br><br>Brief Child Toddler Social and Emotional Assessment<br>Temperament and Atypical Behaviour Score<br><br>Child Characteristic Questionnaire	**No differences** between high and low intensity programme using mean scores or cut-offs for 4 comparisons: personal social score, personal social cut-off, social-emotional score, social-emotional cut-off (p values adjusted for multiple testing)<br>**No differences** between high and low intensity programme using mean scores or cut-offs for 4 comparisons: competence score, competence cut-off, problem score, problem cut-off (p values adjusted for multiple testing)<br>**No differences** between high and low intensity programme using mean scores or binary variable (p values **not** adjusted for multiple testing)<br>**No difference** between high and low intensity programme for score based on a 6 point scale of difficult temperament (p value **not** adjusted for multiple testing)
Doyle 2011 (Long term whilst intervention is ongoing)	Ages and Stages Questionnaire (measured at 18, 24 and 36 months)<br><br>Brief Child Toddler Social and Emotional Assessment (measured at 18, 24 and 36 months)<br>Brief Child Toddler Social and Emotional Assessment subdomains (measured at 24 and 36 months)<br>Child Behaviour Checklist (CBCL, measured at 24 and 36 months)<br><br>Child Behaviour Checklist subdomains (CBCL, measured at 24 and 36 months)	**No differences** between high and low intensity programme using mean scores or cut-offs for 4 comparisons: personal social score, personal social cut-off, social-emotional score, social-emotional cut-off (p values adjusted for multiple testing)<br>**No differences** between high and low intensity programme using mean scores or cut-offs for 4 comparisons at 18 and 24 months: competence score, competence cut-off, problem score, problem cut-off, and for 2 comparisons at 36 months: competence score, problem score (p values adjusted for multiple testing)<br>**No differences in mean scores** between high and low intensity programme for 5 comparisons at 24 or 36 months: dysregulation, internal problems, red flag, external problems, autism (p value adjusted for multiple testing).<br>At 24 months, **no difference** between high and low intensity for 5 comparisons (CBCL total score; internalising problems score and cut-off, externalising problems score and cut-off); **higher proportion in the low intensity group above cut-off for total CBCL score** (0.00 [SD 0.00] vs 0.07 [0.26], p<0.01 adjusted for multiple testing)<br>At 36 months, **no difference** between high and low intensity for 6 comparisons (CBCL total score and cut-off; internalising problems score and cut-off, externalising problems score and cut-off)<br>**No difference** in scores between high and low intensity programmes for 8 comparisons at 24 or 36 months: sleep problems, other problems, emotionally reactive, attention problems, anxious/depressed, somatic complaints, aggressive, withdrawn (p values adjusted for multiple testing)
Johnston 2006 (Long term whilst intervention is ongoing)	Subscales of Child Behaviour Checklist	**Higher mean score** in HS+PP group than HS only group for anxiety or depression at 36 months (aMD 0.40; 95% CI 0.07, 0.73; p<0.05).<br>**No difference** in aggressive behaviour between HS+PP group and HS only group.

For peer review only - http://bmjopen.bmj.com/site/about/guidelines.xhtml

Landry 2006<br>(Short term post-intervention)	Coding of videotaped interactions between child and mother, and child with examiner	**No difference** in mean scores for cooperation with mothers (p=0.08) or a novel adults (results not presented).<br>Infants in PALS demonstrated "**significantly greater increase in their use of words**" when interacting with mothers (p=0.02) and a novel adult (p=0.02).<br>**No effect** on affect in interactions with mothers overall (results not presented); **lower negative affect** seen in infants in the PALS group when interacting with a novel adult (results not presented).<br>Also present some results for the interaction with birthweight but presentation of results incomplete (text suggests more positive effects of the PALS intervention in very low birthweight group)

For peer review only

Table C5: Overall Development Outcomes

Overall development outcomes		
**Studies comparing one intervention with usual care**		
**Study**	**Measure**	**Results**
Chang 2015 (Short term post intervention)	Griffith Mental Development Scale	**No difference in mean developmental quotient (Griffith)** between intervention and control (mean 96.14, SD 9.40 compared with mean 94.68, SD 8.25); adjusted mean difference 1.10, 95% CI -0.45, 2.65; p value for comparison = 0.72)
Griffith 2011 (Short term post-intervention)	Schedule of Growing Skills II (SGS II)	**No difference in mean scores** between intervention (mean 99.20, SD 18.27) and control (mean 102.38, SD 17.79) at around 3 months post-intervention (p=0.51)
Wiggins 2004 (Immediately post-intervention)	Question on mother's perception of whether her child's development was normal	**No difference** between “supportive home visiting” and control (risk ratio 0.88, 95% CI 0.39-1.99) or “community groups” and control (risk ratio 0.57, 95% CI 0.22-1.52) in mother's perception of whether her child's development was normal
Wiggins 2004 (Short term post-intervention)	Questions on mother’s worries about different aspects of development	Comparison on general development not possible between SHV and control as no mothers expressed concerns in the SHV group.<br>**No difference** in the number of number of mothers expressing worries about general development between CGS and control (risk ratio 0.75, 95% CI 0.15, 1.92)<br>Mothers in the SHV group had **fewer mean number of worries** about their child's development than in the control group (MD -0.23, 95% CI -0.42 to -0.01)<br>**No difference in the mean number of worries** about their child's development between CGS and control (MD 0.13, 95% CI -0.10 to 0.36)
**Overall development outcomes**		
**Studies comparing one intervention with usual care**		
**Study**	**Measure**	**Results**
Doyle 2011 (Short term whilst intervention is ongoing)	Ages and Stages Questionnaire	**No difference in overall mean score** between high and low intensity programme (p value **not** adjusted for multiple testing) at 6 months
Doyle 2011 (Medium term whilst intervention is ongoing)	Ages and Stages Questionnaire	**No difference in overall mean score** between high and low intensity programme (p value **not** adjusted for multiple testing) at 12 months
Doyle 2011 (Long term whilst intervention is ongoing)	Ages and Stages Questionnaire (measured at 18, 24 and 36 months)	**No difference in overall mean score** between high and low intensity programme (p value **not** adjusted for multiple testing) at any of the time points

For peer review only

D. Summary of results, stratified by other characteristics

D1: Results stratified by age of children at intervention delivery

Study<br>Intensity of intervention	Motor development outcomes	Language development outcomes	Cognitive development outcomes	Social and emotional wellbeing outcomes	Overall child development
**Studies comparing one intervention with usual care**					
**Intervention during antenatal and/or first year postpartum only**					
Beeghly 1995	Short term post-intervention:<br>No effect in whole sample (1 test) or sub-groups (1 test)	-	Short term post-intervention:<br>No effect in whole sample (1 test) or sub-groups (1 test)	-	-
Cheng 2007	-	-	-	Short term post-intervention:<br>No comparisons in whole sample; Positive effect in 1 of 2 sub-group tests<br>Long term post-intervention:<br>No effect in whole sample (3 tests)	-
Cupples 2011	Immediately post-intervention:<br>No effect (1 test)	-	Immediately post-intervention:<br>No effect (1 test)	Immediately post-intervention:<br>No effect (4 tests)	-
Feinberg 2008	-	-	-	Short term post-intervention:<br>No effect in 2 & positive effect in 3 of 5 tests<br>Medium term post-intervention:<br>No effect in 1 & positive effect in 1 of 2 tests<br>Long term post-intervention:<br>No effect in whole sample (7 tests)<br>5 significant interactions (of 7 tested, suggesting intervention effects in boys)	-
Lobo 2012	Short term whilst intervention ongoing:<br>No effect in 4 & positive effect in 4 of 8 tests<br>Short to medium term post-intervention:<br>Positive effect in 5 of 5 tests	-	-	-	-
Santelices 2011	-	-	-	Immediately to short term post-intervention:<br>No effect (1 test)	-

For peer review only

Wiggins 2004<br>Moderate intensity intvn	-	Short term post-intervention:<br>Positive effect (1 test)	-	Short term post-intervention:<br>No effect (1 test)	Immediately post-intervention:<br>No effect (1 test)<br>Short term post-intervention:<br>No effect in 1 & positive effect in 1 of 2 tests
Wiggins 2004<br>Low intensity intvn	-	Short term post-intervention:<br>No effect (1 test)	-	Short term post-intervention:<br>No effect (1 test)	Immediately post-intervention:<br>No effect (1 test)<br>Short term post-intervention:<br>No effect (2 tests)
**Intervention during first and second years postpartum**					
Chang 2015	Short term post-intervention:<br>No effect (1 test)	Short term post-intervention:<br>No effect (2 tests)	Short term post-intervention:<br>No effect in unadjusted test; positive effect on adjusting for multiple potential confounders	-	Short term post-intervention:<br>No effect (1 test)
Christakis 2007	-	Short term post-intervention:<br>No effect (2 tests)<br>Positive effect in 2 sub-group tests (results only presented for middle and low income group)	-	Short term post-intervention:<br>No effect in whole sample (1 test) or sub-groups (1 test)	-
Doyle 2011	Short, medium and long term whilst intervention ongoing:<br>No effect (20 tests)	Short, medium and long term whilst intervention ongoing:<br>No effect (21 tests)	Short, and medium term whilst intervention ongoing:<br>No effect (6 tests)<br>Long term whilst intervention ongoing:<br>No effect in 9 & positive effect in 3 of 12 tests	Short, and medium term whilst intervention ongoing:<br>No effect (16 tests)<br>Long term whilst intervention ongoing:<br>No effect in 61 & positive effect in 1 of 62 tests	Short, medium and long term whilst intervention ongoing:<br>No effect (4 tests)
Drotar 2008	-	Long term whilst intervention ongoing:<br>No effect (1 test)	Short-medium term whilst intervention ongoing:<br>No effect (5 tests)<br>Long term whilst intervention ongoing:<br>No effect in 11 & positive effect in 1 of 12 tests<br>No effect in 10 & positive effect in 2 of 12 sub-group tests	Short, medium and long term whilst intervention ongoing:<br>No effect (6 tests)	-
Griffith 2011	-	-	-	Short-term post-intervention:<br>No effect (1 test)	Short-term post-intervention:<br>No effect (1 test)
High 2000	-	Short-term post-intervention:<br>No effect in 3 & positive effect in 3 of 6 tests<br>No effect in 6 & positive effect in 6 of	-	-	-

For peer review only

		12 tests in sub-groups			
Hiscock 2008	-	-	-	Short, medium and long term post-intervention:<br>No effect (6 tests)	-
Johnston 2006	-	Long term whilst intervention ongoing:<br>No effect in 2 & positive effect in 2 of 4 tests	-	Long term whilst intervention ongoing:<br>No effect in 2 & negative effect in 1 of 3 tests	-
Landry 2006	-	Short term post-intervention:<br>Positive effect in 2 of 2 tests	Short term post-intervention:<br>Positive effect (1 test)	Short term post-intervention:<br>No effect in 3 & positive effect in 3 of 6 tests	-
Miller 2015	-	-	Long-term whilst intervention ongoing:<br>No effect (1 test)	Long-term whilst intervention ongoing:<br>No effect (1 test)	-
Minkovitz 2003	-	-	-	Long-term whilst intervention ongoing:<br>No effect (3 tests)	-
Niccols 2008	-	-	-	Immediately and short term post-intervention:<br>No effect (2 tests)	-
Niccols 2009	-	-	-	Immediately and short term post-intervention:<br>No effect in 3 & positive effect in 5 of 8 tests	-
Tsiantis 2000	-	Long term whilst intervention ongoing:<br>Negative effect in 2 of 2 tests<br>Sub-group (country-specific) results not presented	-	Short term whilst intervention ongoing:<br>No effect in whole sample (2 tests)<br>Positive effect in 1 of 8 tests in sub-groups<br>Medium term post-intervention:<br>No effect in 1 & negative effect in 1 of 2 tests in whole sample<br>Long term whilst intervention ongoing:<br>No effect in 7 & positive effect in 1 of 8 tests<br>Inconsistent reporting of sub-groups, 2 positive effects mentioned but total number of tests not specified	-

For peer review only

Wagner 2001	Long term whilst intervention ongoing:<br>No effect in whole sample (1 test) or sub-groups (2 tests)	Long term whilst intervention ongoing:<br>No effect in whole sample (1 test) or sub-groups (2 tests)	Long term whilst intervention ongoing:<br>No effect in whole sample (1 test) or sub-groups (2 tests)	Long term whilst intervention ongoing:<br>No effect in whole sample (5 tests) or sub-groups (10 tests)	-

For peer review only

D2: Results stratified by whether intervention delivery was universal or geographically targeted

Study<br>Intensity of intervention	Motor development outcomes	Language development outcomes	Cognitive development outcomes	Social and emotional wellbeing outcomes	Overall child development
**Studies comparing one intervention with usual care**					
**Universal**					
Beeghly 1995	Short term post-intervention:<br>No effect in whole sample (1 test) or sub-groups (1 test)	-	Short term post-intervention:<br>No effect in whole sample (1 test) or sub-groups (1 test)	-	-
Cheng 2007	-	-	-	Short term post-intervention:<br>No comparisons in whole sample; Positive effect in 1 of 2 sub-group tests<br>Long term post-intervention:<br>No effect in whole sample (3 tests)	-
Christakis 2007	-	Short term post-intervention:<br>No effect (2 tests)<br>Positive effect in 2 sub-group tests (results only presented for middle and low income group)	-	Short term post-intervention:<br>No effect in whole sample (1 test) or sub-groups (1 test)	-
Drotar 2008	-	Long term whilst intervention ongoing:<br>No effect (1 test)	Short-medium term whilst intervention ongoing:<br>No effect (5 tests)<br>Long term whilst intervention ongoing:<br>No effect in 11 & positive effect in 1 of 12 tests<br>No effect in 10 & positive effect in 2 of 12 sub-group tests	Short, medium and long term whilst intervention ongoing:<br>No effect (6 tests)	-
Feinberg 2008	-	-	-	Short term post-intervention:<br>No effect in 2 & positive effect in 3 of 5 tests<br>Medium term post-intervention:<br>No effect in 1 & positive effect in 1 of 2 tests<br>Long term post-intervention:<br>No effect in whole sample (7 tests)<br>5 significant interactions (of 7 tested, suggesting intervention effects in boys)	-

For peer review only

For peer review only - http://bmjopen.bmj.com/site/about/guidelines.xhtml

Hiscock 2008	-	-	-	Short, medium and long term post-intervention:<br>No effect (6 tests)	-
Johnston 2006	-	Long term whilst intervention ongoing:<br>No effect in 2 & positive effect in 2 of 4 tests	-	Long term whilst intervention ongoing:<br>No effect in 2 & negative effect in 1 of 3 tests	-
Lobo 2012	Short term whilst intervention ongoing:<br>No effect in 4 & positive effect in 4 of 8 tests<br>Short to medium term post-intervention:<br>Positive effect in 5 of 5 tests	-	-	-	-
Miller 2015	-	-	Long-term whilst intervention ongoing:<br>No effect (1 test)	Long-term whilst intervention ongoing:<br>No effect (1 test)	-
Minkovitz 2003	-	-	-	Long-term whilst intervention ongoing:<br>No effect (3 tests)	-
Niccols 2008	-	-	-	Immediately and short term post-intervention:<br>No effect (2 tests)	-
Niccols 2009	-	-	-	Immediately and short term post-intervention:<br>No effect in 3 & positive effect in 5 of 8 tests	-
Santelices 2011	-	-	-	Immediately to short term post-intervention:<br>No effect (1 test)	-
Tsiantis 2000	-	Long term whilst intervention ongoing:<br>Negative effect in 2 of 2 tests<br>Sub-group (country-specific) results not presented	-	Short term whilst intervention ongoing:<br>No effect in whole sample (2 tests)<br>Positive effect in 1 of 8 tests in sub-groups<br>Medium term post-intervention:<br>No effect in 1 & negative effect in 1 of 2 tests in whole sample<br>Long term whilst intervention	-

For peer review only

				ongoing:<br>No effect in 7 & positive effect in 1 of 8 tests<br>Inconsistent reporting of sub-groups, 2 positive effects mentioned but total number of tests not specified	
**Geographically targeted**					
Chang 2015	Short term post-intervention:<br>No effect (1 test)	Short term post-intervention:<br>No effect (2 tests)	Short term post-intervention:<br>No effect in unadjusted test; positive effect on adjusting for multiple potential confounders	-	Short term post-intervention:<br>No effect (1 test)
Cupples 2011	Immediately post-intervention:<br>No effect (1 test)	-	Immediately post-intervention:<br>No effect (1 test)	Immediately post-intervention:<br>No effect (4 tests)	-
Doyle 2011	Short, medium and long term whilst intervention ongoing:<br>No effect (20 tests)	Short, medium and long term whilst intervention ongoing:<br>No effect (21 tests)	Short, and medium term whilst intervention ongoing:<br>No effect (6 tests)<br>Long term whilst intervention ongoing:<br>No effect in 9 & positive effect in 3 of 12 tests	Short, and medium term whilst intervention ongoing:<br>No effect (16 tests)<br>Long term whilst intervention ongoing:<br>No effect in 61 & positive effect in 1 of 62 tests	Short, medium and long term whilst intervention ongoing:<br>No effect (4 tests)
Griffith 2011	-	-	-	Short-term post-intervention:<br>No effect (1 test)	Short-term post-intervention:<br>No effect (1 test)
High 2000	-	Short-term post-intervention:<br>No effect in 3 & positive effect in 3 of 6 tests<br>No effect in 6 & positive effect in 6 of 12 tests in sub-groups	-	-	-
Landry 2006	-	Short term post-intervention:<br>Positive effect in 2 of 2 tests	Short term post-intervention:<br>Positive effect (1 test)	Short term post-intervention:<br>No effect in 3 & positive effect in 3 of 6 tests	-
Wagner 2001	Long term whilst intervention ongoing:<br>No effect in whole sample (1 test) or sub-groups (2 tests)	Long term whilst intervention ongoing:<br>No effect in whole sample (1 test) or sub-groups (2 tests)	Long term whilst intervention ongoing:<br>No effect in whole sample (1 test) or sub-groups (2 tests)	Long term whilst intervention ongoing:<br>No effect in whole sample (5 tests) or sub-groups (10 tests)	-
Wiggins 2004<br>Moderate intensity intvn	-	Short term post-intervention:<br>Positive effect (1 test)	-	Short term post-intervention:<br>No effect (1 test)	Immediately post-intervention:<br>No effect (1 test)<br>Short term post-intervention:<br>No effect in 1 & positive effect in 1 of 2 tests
Wiggins 2004<br>Low intensity intvn	-	Short term post-intervention:<br>No effect (1 test)	-	Short term post-intervention:<br>No effect (1 test)	Immediately post-intervention:<br>No effect (1 test)

For peer review only

					Short term post-intervention:<br>No effect (2 tests)

For peer review only

D3: Results stratified by socio-economic status of the families included

Not enough information in a majority of papers to perform this comparison adequately.

For peer review only

PRISMA 2009 Checklist

Section/topic	#	Checklist item	Reported on page #
**TITLE**			
Title	1	Identify the report as a systematic review, meta-analysis, or both.	6
**ABSTRACT**			
Structured summary	2	Provide a structured summary including, as applicable: background; objectives; data sources; study eligibility criteria, participants, and interventions; study appraisal and synthesis methods; results; limitations; conclusions and implications of key findings; systematic review registration number.	2-3
**INTRODUCTION**			
Rationale	3	Describe the rationale for the review in the context of what is already known.	5-6
Objectives	4	Provide an explicit statement of questions being addressed with reference to participants, interventions, comparisons, outcomes, and study design (PICOS).	6
**METHODS**			
Protocol and registration	5	Indicate if a review protocol exists, if and where it can be accessed (e.g., Web address), and, if available, provide registration information including registration number.	6 and protocol uploaded
Eligibility criteria	6	Specify study characteristics (e.g., PICOS, length of follow-up) and report characteristics (e.g., years considered, language, publication status) used as criteria for eligibility, giving rationale.	6-7
Information sources	7	Describe all information sources (e.g., databases with dates of coverage, contact with study authors to identify additional studies) in the search and date last searched.	8
Search	8	Present full electronic search strategy for at least one database, including any limits used, such that it could be repeated.	Web appendix 1
Study selection	9	State the process for selecting studies (i.e., screening, eligibility, included in systematic review, and, if applicable, included in the meta-analysis).	8
Data collection process	10	Describe method of data extraction from reports (e.g., piloted forms, independently, in duplicate) and any processes for obtaining and confirming data from investigators.	8
Data items	11	List and define all variables for which data were sought (e.g., PICOS, funding sources) and any assumptions and simplifications made.	8
Risk of bias in individual studies	12	Describe methods used for assessing risk of bias of individual studies (including specification of whether this was done at the study or outcome level), and how this information is to be used in any data synthesis.	8

For peer review only

PRISMA 2009 Checklist

Section/topic	#	Checklist item	Reported on page #
Summary measures	13	State the principal summary measures (e.g., risk ratio, difference in means).	N/A (narrative synthesis)
Synthesis of results	14	Describe the methods of handling data and combining results of studies, if done, including measures of consistency (e.g., I^2) for each meta-analysis.	N/A (narrative synthesis)
Risk of bias across studies	15	Specify any assessment of risk of bias that may affect the cumulative evidence (e.g., publication bias, selective reporting within studies).	8
Additional analyses	16	Describe methods of additional analyses (e.g., sensitivity or subgroup analyses, meta-regression), if done, indicating which were pre-specified.	9
**RESULTS**			
Study selection	17	Give numbers of studies screened, assessed for eligibility, and included in the review, with reasons for exclusions at each stage, ideally with a flow diagram.	9 and Figure 1
Study characteristics	18	For each study, present characteristics for which data were extracted (e.g., study size, PICOS, follow-up period) and provide the citations.	9 and Table 1
Risk of bias within studies	19	Present data on risk of bias of each study and, if available, any outcome level assessment (see item 12).	Figures 2 and 3
Results of individual studies	20	For all outcomes considered (benefits or harms), present, for each study: (a) simple summary data for each intervention group (b) effect estimates and confidence intervals, ideally with a forest plot.	12-13 (narrative synthesis)
Synthesis of results	21	Present results of each meta-analysis done, including confidence intervals and measures of consistency.	N/A (narrative synthesis)
Risk of bias across studies	22	Present results of any assessment of risk of bias across studies (see Item 15).	12-13 and Table 3
Additional analysis	23	Give results of additional analyses, if done (e.g., sensitivity or subgroup analyses, meta-regression [see Item 16]).	13 and Web Appendix D

For peer review only

PRISMA 2009 Checklist

**DISCUSSION**			
Summary of evidence	24	Summarize the main findings including the strength of evidence for each main outcome; consider their relevance to key groups (e.g., healthcare providers, users, and policy makers).	14
Limitations	25	Discuss limitations at study and outcome level (e.g., risk of bias), and at review-level (e.g., incomplete retrieval of identified research, reporting bias).	15
Conclusions	26	Provide a general interpretation of the results in the context of other evidence, and implications for future research.	16
**FUNDING**			
Funding	27	Describe sources of funding for the systematic review and other support (e.g., supply of data); role of funders for the systematic review.	17

From: Moher D, Liberati A, Tetzlaff J, Altman DG, The PRISMA Group (2009). Preferred Reporting Items for Systematic Reviews and Meta-Analyses: The PRISMA Statement. PLoS Med 6(7): e1000097. doi:10.1371/journal.pmed1000097

For more information, visit: www.prisma-statement.org.

For peer review only

For peer review only - http://bmjopen.bmj.com/site/about/guidelines.xhtml

BMJ Open

Interventions that enhance health service contact with parents and infants to improve child development and social and emotional wellbeing in high-income countries: A systematic review

Journal:	*BMJ Open*
Manuscript ID	bmjopen-2016-014899.R1
Article Type:	Research
Date Submitted by the Author:	24-Apr-2017
Complete List of Authors:	Hurt, Lisa; Cardiff University School of Medicine, Division of Population Medicine<br>Paranjothy, Shantini; Cardiff University School of Medicine, Division of Population Medicine<br>Lucas, Patricia; Unversity of Bristol, School for Policy Studies<br>Watson, Debbie; University of Bristol, School for Policy Studies<br>Mann, Mala; Cardiff University, Specialist Unit for Review Evidence (SURE)<br>Griffiths, Lucy; UCL Great Ormond Street Institute of Child Health, Population, Policy and Practice Programme<br>Ginja, Samuel; Institute of Health and Society, Newcastle University<br>Paljarvi, Tapio; Cardiff University School of Medicine, Division of Population Medicine<br>Williams, Jo; Bristol City Council<br>Bellis, Mark; Bangor University, College of Health and Behavioural Sciences; Public Health Wales, Policy, Research and International Development<br>Lingam, Raghu; Institute of Health & Society, Newcastle University
**Primary Subject Heading**:	Evidence based practice
Secondary Subject Heading:	Health policy, Paediatrics, Public health, Epidemiology
Keywords:	Child development, Social and emotional wellbeing, Universal health services, Early intervention

SCHOLARONE™
Manuscripts

Interventions that enhance health service contact with parents and infants to improve child development and social and emotional wellbeing in high-income countries: A systematic review

Lisa Hurt[1], Shantini Paranjothy[1], Patricia J Lucas[2], Debbie Watson[2], Mala Mann[3], Lucy J Griffiths[4], Samuel Ginja[5], Tapio Paljarvi[1], Jo Williams[6], Mark Bellis[7], Raghu Lingam[5]

[1] Division of Population Medicine, Cardiff University School of Medicine, 5th Floor, Neuadd Meirionnydd, Heath Park, Cardiff CF14 4YS

Lisa Hurt Lecturer

Shantini Paranjothy Mansel Talbot Professor of Preventive Medicine

Tapio Paljarvi Research Fellow

[2] School for Policy Studies, University of Bristol, 8 Priory Rd, Bristol BS8 1TZ

Patricia J Lucas Reader

Debbie Watson Reader

[3] Specialist Unit for Review Evidence, Cardiff University, Neuadd Meirionnydd, Heath Park, Cardiff CF14 4YS

Mala Mann Information Specialist/Systematic Reviewer

[4] Population, Policy and Practice Programme, UCL Great Ormond Street Institute of Child Health, 30 Guilford St, London WC1N 1EH

Lucy J Griffiths Senior Research Associate

[5] Institute of Health and Society, Newcastle University, Baddiley-Clark Building, Newcastle upon Tyne NE2 4AX

Samuel Ginja Research Associate

Raghu Lingam Clinical Senior Lecturer/Honorary Consultant

[6] Bristol City Council, City Hall, PO Box 3176, Bristol BS3 9FS

Jo Williams Consultant in Child Public Health

[7] Public Health Wales, 2 Capital Quarter, Tyndall Street, Cardiff, CF10 4BZ

Mark Bellis Professor/Director of Policy, Research and Development

Correspondence to: L Hurt, hurtl@cardiff.ac.uk, 029 2068 7807

Word count: 5,409

For peer review only

For peer review only - http://bmjopen.bmj.com/site/about/guidelines.xhtml

Abstract

Background: Experiences in the first 1000 days of life have a critical influence on child development and health. Health services that provide support for families need evidence about how best to improve their provision.

Methods: We systematically reviewed the evidence for interventions in high-income countries to improve child development by enhancing health service contact with parents from the antenatal period to 24 months postpartum. We searched 15 databases and trial registers for studies published in any language between 01/01/1996 and 01/04/2016, using subject headings and key words with the following search structure: [(health OR parenting professionals) OR known programme names] AND [child development OR motor OR cognitive OR emotional/behavioural OR language outcomes]. We also searched 58 programme or organisation websites and the electronic table of contents of eight journals.

Results: Primary outcomes were motor, cognitive, and language development, and social-emotional wellbeing, measured to 39 months of age (to allow the interventions time to produce demonstrable effects). Results were reported using narrative synthesis, due to the variation in study populations, intervention design and outcome measurement. 22 of the 12,986 studies identified met eligibility criteria. Using GRADE criteria, the quality of evidence overall was moderate to low. There was limited evidence for intervention effectiveness: positive effects were seen in 1/6 studies for motor development, 4/11 for language development, 4/8 for cognitive development, and 3/19 for social-emotional wellbeing. However, most studies showing positive effects were at high/unclear risk of bias, within-study effects were inconsistent, and negative effects were also seen. Intervention content and intensity varied greatly, but this was not associated with effectiveness.

Conclusions: There is insufficient evidence that interventions currently available to enhance health service contacts up to 24 months postpartum are effective for improving child development. There is an urgent need for robust evaluation of existing interventions, and to develop and evaluate novel interventions to enhance the offer to all families.

Registration: The protocol for this systematic review was registered in the International Prospective Register of Systematic Reviews (PROSPERO CRD42015015468) on 12 January 2015.

For peer review only

For peer review only - http://bmjopen.bmj.com/site/about/guidelines.xhtml

Article summary

Strengths and limitations of this study

- To our knowledge, this is the first systematic review of interventions that enhance health service contacts to improve child development outcomes including social and emotional wellbeing outcomes.
- We used a broad systematic search of the extensive literature in this field, and searched many sources in addition to database searches.
- We reviewed a larger number of primary studies than previous reviews of interventions in the early years. Our conclusion is consistent with these reviews.
- It was not possible to conduct a meta-analysis due to the variation in the types of interventions and methods used to measure outcomes.
- We do not report parental outcomes, and cannot comment on whether parents benefitted from these enhancements.

For peer review only - http://bmjopen.bmj.com/site/about/guidelines.xhtml

Introduction

Experiences in the first 1000 days of life have a crucial influence on child development and health.[1] Appropriate early child development (including physical, social and emotional, language and cognitive domains) has consistently been shown to be associated with good health and educational outcomes in childhood, and consequent health and employment outcomes in adulthood.[2-4] Adopting a life-course approach, including early intervention, is essential,[5] and investment is therefore needed in effective pre and postnatal services to optimise child health, wellbeing and developmental resilience.[6]

The content of health services to promote maternal and child health during pregnancy and the early years varies across countries. A recent review suggested that the best services in Europe are "*characterized by personalised ongoing support during pregnancy, choice in birth arrangements, postnatal support and advice, and paid parental leave for mothers and fathers*".[7] In most high income settings, early years services also work to a "proportionate universalism" model where care is available to all, irrespective of need, with enhanced support offered to families at high risk of adverse outcomes.[1]

There is high-quality global evidence to support the effectiveness of many components of early years services including elements of antenatal care and centre-based preschool provision.[7 8] Interventions to promote child development in the early years have also been developed. However, most have been targeted at and evaluated with high-risk families or children with an identified condition.[9-11] An unacceptably high proportion of children in both high and low income settings do not achieve expected early learning goals before they start school,[12] and it has been argued that targeted approaches alone may not be sufficient.[13] Interventions to enhance contacts with all parents in existing services may be more effective in improving child development outcomes for several reasons. First, not all children who need support are identified by a targeted approach.[14] Targeting can lead to stigmatisation resulting in poor uptake or adherence.[15] Embedding interventions within an existing service, such as health visiting, which provides ongoing and consistent support for parents, may also improve the interaction between health professionals and parents and improve access to care at a crucial time in their child's development, leading to improvements in child development outcomes.[11] A review of interventions in low and middle income settings

For peer review only - http://bmjopen.bmj.com/site/about/guidelines.xhtml

noted that there was great diversity in both the scope and focus of research in this area, and concluded that parents in such settings "*need to be supported in providing nurturing care and protection in order for young children to achieve their developmental potential*".[16] However, the effectiveness of such interventions to enhance existing multi-disciplinary services in high income settings is not known.

Previous reviews of early interventions in high income settings fail to provide a full picture of interventions relevant to public health policy and practice because they do not provide a comprehensive examination of child development outcomes in the very early years (that is, the period during which the human brain develops most rapidly[17]). Neither does the evidence base to date include social and emotional wellbeing outcomes, nor are these consistently defined and articulated. The objective of this systematic review is to fill these gaps, by examining the effect of interventions designed to enhance health service contacts with all parents and children in the very early years (defined as the antenatal period to 24 months postpartum) on child development and social and emotional wellbeing outcomes. Our research question was developed in partnership with local policy makers, and provides evidence for policy.[18]

Methods

Protocol and registration

The protocol for this systematic review was registered in the International Prospective Register of Systematic Reviews (PROSPERO CRD42015015468) on 12 January 2015. This review is reported in accordance with PRISMA guidelines.[19]

Inclusion and exclusion criteria

We included randomised controlled trials (with individual or cluster randomisation) in any language that were published or unpublished. The interventions of interest were ones delivered within existing multi-disciplinary healthcare services that are the cornerstone of early years programmes and are available to all. The interventions may be delivered by a range of staff within these services. We included studies from the 76 countries and territories classified by the World Bank in July 2014 as "high-income economies". Studies published in any language were eligible for inclusion.

For peer review only - http://bmjopen.bmj.com/site/about/guidelines.xhtml

To capture the effects of interventions delivered in the very early years, we included programmes that were delivered at any time from the antenatal period to 24 months postpartum. Given that some programmes continue beyond the child's second birthday, we specified that studies would be included if the mean age of the children at the start of the intervention was less than or equal to 24 months. To allow time for these interventions to produce demonstrable effects, we included studies that examined outcomes to 39 months of age (given that not all studies would manage to assess children on their 3rd birthday exactly).

Studies that selected participants from the general population or included all individuals from a specific neighbourhood (for example, an area-based programme defined on the basis of postcode or zip code, known as "geographically targeted" programmes in this review) were included. Studies were excluded if they selected participants based on individual risk factors (for example, an individually-assessed income threshold for participating families, or parental illness) or specific clinical sub-groups (such as preterm babies or children with specific diagnoses).

Interventions

We included interventions that were provided within the framework of the existing healthcare system. They could be designed to augment routine healthcare provision for all children in different ways, for example by improving the skills or parental capacity of the parents or the family, improving the interaction between health professionals and parents, improving access to healthcare for the parents or the child, or including elements designed to promote a specific area of child development. These included training modules designed to be delivered to parents with the intention of improving child development outcomes, or any resources (such as printed materials, films, Apps) that health professionals or their support workers could use in their work with parents. Interventions could be delivered at home, in group-based settings (for example, in general, obstetric or paediatric practice, in hospitals or community settings), through telemedicine, or via a combination of different methods.

For peer review only

For peer review only - http://bmjopen.bmj.com/site/about/guidelines.xhtml

There is an argument that these different approaches should be separated into different systematic reviews (or indeed separated by outcome). We, like others,[10 16] chose to include these in a single review to avoid divisions that were arbitrary from a developmental or service delivery perspective, and to avoid multiple overlapping, small and/or empty reviews in a field with limited literature.

Outcomes

The outcomes were motor development (fine and gross), cognitive development, language development (receptive and expressive), social and emotional wellbeing; and global child development. We included studies that used validated tools to measure these outcomes. Where unvalidated tools were used, we considered to be secondary outcomes. Studies were included if they measured outcomes at any time between three months of age and 39 months postpartum (specifically, where the average age of the children at outcome measurement was 39 months or less).

Search strategy

We searched for articles published in any language between 01/01/1996 and 01/04/2016 in the following databases: Cochrane Central Register of Controlled Trials (CENTRAL); Medline; Embase; CINAHL; PsycINFO; Web of Science; Scopus; ASSIA; LiLACS; Sociological Abstracts; Social Services Abstract; and OpenGrey; and the following trial registers: ClinicalTrials.gov; UK Clinical Trials Gateway; and WHO International Clinical Trials Registry Platform. Given our focus on enhancement of existing health services, we restricted to studies published within 20 years of our study inception since health service change has been substantial in the mid to late 20th century. We used a combination of Medical Subject Headings and free text including terms for interventions to enhance health service contacts combined with terms relating to child development outcomes. Terms for the interventions included those that listed the professional delivering the programme (including "health visitor", "community nurse", "nurse", "midwife", "general practitioner", "early years educator", "parent educator"), and programme names that were already known to the review team. The Medline search strategy is shown in Web Appendix A. We also searched websites of 58 relevant programmes and organisations, and the electronic table of contents (eTOC) of eight key journals for relevant studies published within the last two years (see Web Appendix B

For peer review only - http://bmjopen.bmj.com/site/about/guidelines.xhtml

for a full list). Reference lists of included and key papers were reviewed, and authors contacted for additional data where necessary.

Study selection

All references identified by the searches were downloaded into Endnote and duplicates removed. Titles and abstracts were screened for inclusion independently by two of three reviewers (LH and LJG or SP). Full text versions were obtained for the papers potentially meeting the inclusion criteria and were screened independently by two of three reviewers (LH and LJG or SP). Disagreements were resolved through discussion and in consultation with others in the review group.

Data abstraction process

A data extraction form designed for the requirements of this review was used, which included details on the characteristics of the included studies, the interventions studied and assessment of risk of bias and GRADE criteria. Multiple publications and reports from the same trial were linked, and compared for completeness and contradictions. Data from each paper were extracted independently and in duplicate (completed by LH and LJG or SG or SP or TP).

Analysis

Risk of bias was assessed following Cochrane guidelines.[20] Due to variation in i) the populations studied, ii) the design of the interventions and iii) the wide range of outcome measures used (both in terms of the child development domains and/or the instruments used to assess the outcomes), it was not possible to conduct a meta-analysis and results were reported using narrative synthesis. We specified a priori that we would examine the results stratified by i) risk of bias, ii) the intensity of the intervention, iii) the age of the child at which the intervention was delivered, iv) whether the programme was available to all or geographically targeted, and v) socio-demographic characteristics of the families in the trial. We selected these variables as we hypothesised that they would help to identify the characteristics of the interventions most likely to be effective (for example, if high intensity interventions were more effective than low intensity ones) or the populations in which they

For peer review only - http://bmjopen.bmj.com/site/about/guidelines.xhtml

were most likely to be effective (for example, if programmes recruiting from defined neighbourhoods were more effective than those made available to all).

An assessment of the intensity of each intervention was conducted independently and in duplicate (completed by LH and LJG or SG or SP or TP) based on seven criteria: i) total number of visits; ii) total duration of the programme; iii) total number of contact hours; iv) frequency of visits; v) number of components; vi) whether components were delivered directly to parents and/or children; and vii) whether the components were delivered on a one-to-one basis or in a group session. Using these seven characteristics, we categorised the overall intensity for each intervention as “low”, “moderate” or “high”. Two review authors made this assessment using subjective determination rather than a predefined algorithm or a scoring system (as used in [21]), to allow for the diversity and complex combinations of components to be reflected in the categorisation. Finally, the quality of the overall evidence for each outcome was assessed using GRADE criteria.[22]

Public Involvement

This work was conducted in collaboration with the Bristol Network for Early Years Health and Wellbeing (www.bonee.org) and a range of stakeholders have been involved in the design and conduct of this initiative. Parents were not involved in the design and conduct of the review, but we are discussing the results and interpretation with parents.

Results

15,280 records were identified in the database searches (Figure 1). Searches of relevant programme and organisation websites, and eTOC searches yielded 83 additional records. Once all searches were combined and duplicates removed, 12,986 records remained. After title and abstract screening, 12,644 records that were outside the scope of the review were excluded (the vast majority of these because their intervention was targeted at families at high-risk of adverse outcomes or at children with identified conditions). Of the 342 records included in full text review, there were 22 RCTs that fulfilled our inclusion criteria (reported in 40 publications[23-62]). We also identified three relevant ongoing trials.[63-65] Reasons for exclusion are provided in Figure 1.

For peer review only - http://bmjopen.bmj.com/site/about/guidelines.xhtml

Trial characteristics

The 22 included trials are described in Table 1. Three were cluster randomised (clinics[25 46] or healthcare workers[58]), with the remainder randomising individual mothers, parents or mother-child dyads. Ten were conducted in the United States of America, three in the United Kingdom, two in Canada, two in Ireland, one in Australia, one in Chile, one in Japan, and one was a multi-site study conducted in four Southern European countries. Chang (2015) was conducted in Antigua, Jamaica and St Lucia, and is included because Antigua is classified as a high-income country.

17 trials compared one intervention to usual care[25-28 36 37 41 44 46 50 51 54-56 58 59 61], although minor augmentations to usual care were made in six of these (for example, with some other information or services made available to parents who wished to access them[26 36 37 56 58 59]). One of the trials compared two different interventions with usual care.[61] In the remaining five trials, two interventions were compared with each other.[24 30 47-49] The timing of intervention delivery varied, from the first month of life only[24] to longer-term interventions, with eight studies including interventions that continued beyond the child's second birthday,[27 30 36 41 47 50 51 55] and the maximum intervention length being five years.[50] Studies ranged from 28 to 1593 participants: six included fewer than 100 participants; twelve included between 100 and 500; and three included more than 500 participants. One trial did not report the number of subjects recruited or analysed.[58] In 17 of the 22 trials, outcome data were available for 75% or more of those randomised. All of the trials offered coverage of the intervention to all families in the general population or within a neighbourhood or defined population (for example, recruitment occurred in hospitals serving areas with high levels of social disadvantage or the intervention was made available to all individuals within specific postcodes).[25 28 30 41 44 48 59 61] Three trials also included first time mothers only.

Six trials were classified as being at low risk of bias (all compared interventions with usual care), one was at high risk, and 15 had an unclear risk (Figures 2 and 3).

Intervention characteristics

28 interventions were examined in total (see Table 2: 2a for studies that included one intervention; and 2b for studies that compared two interventions). Most papers described

For peer review only - http://bmjopen.bmj.com/site/about/guidelines.xhtml

the body of literature on which the intervention development had been based, but provided less detail on the proposed mechanisms of action of the intervention. Seven were of low intensity: short films followed by group discussions shown in health centre waiting rooms (Chang, 2a); sets of building blocks and activity hand-outs sent to parents by post (Christakis, 2a); "*literacy promoting anticipatory guidance*" by paediatricians (High, 2a); a brief parenting course (Hiscock, 2a); access to community groups (Wiggins, intervention 2, 2a); and two different methods for giving feedback to mothers on a neonatal behavioural assessment (Beeghly, 2b). Ten were of moderate intensity. These included one-to-one home visits (between five [Cheng, 2a] and twelve visits [Wiggins, 2a] in total), group sessions (up to eight in total [Feinberg, Niccols 2008, Niccols 2009, all 2a]), training for primary healthcare workers in interview techniques that encouraged consideration of child development (Tsiantis, 2a), training for parents in daily activities to promote motor development (Lobo, 2b) or a combination of different components (Santelices 2a, Doyle 2b). 11 interventions were of high intensity. They were classified as such because they included multiple components (up to a maximum of 8), and regular contact with parents over a sustained period of time or intensively for a shorter period of time. In the five studies that included two interventions, the interventions were of the same intensity in all but one (Doyle, which compared a medium intensity intervention with one of high intensity), and the aim of the studies was to compare different models of care with each other.

The mode of delivery of the intervention varied between trials. The intervention was delivered by health professionals in seven trials,[24-26 44 46 47 58] by other professionals (including "parent educators", "family visitors", or researchers) in eight trials,[30 36 48 50 54-56 59] by a mixture of health and other professionals in three trials,[37 41 51] and by peer mentors in one trial.[28] One trial examined one intervention delivered by health professionals and another delivered by community support groups.[61] In the remaining trials, one included materials delivered to parents by post,[27] and one examined training for parents by a physiotherapist to deliver a handling and positioning intervention.[49]

A full narrative summary of the results, including the tools used to assess the outcome in each trial and the estimates of intervention effects, is given in Web Appendix C. Many of the trials reported several measures of the same outcome and/or measured outcomes at

For peer review only - http://bmjopen.bmj.com/site/about/guidelines.xhtml

different time points, resulting in multiple comparisons for each outcome. The findings are summarised by outcome in Table 3, and are described below. Effect estimates are given in the text below only for the studies found to be at low risk of bias. An effect direction plot[66] provides a visual display of the results across all outcome domains, ordered by risk of bias and the intensity of the intervention (Table 4).

Motor development outcomes

Six studies, including a total of 37 comparisons in 1276 participants, reported motor development outcomes using validated tools. The quality of the evidence was moderate. Three studies comparing one intervention with usual care showed no effect (972 participants, three comparisons), and three studies in which two interventions were compared (304 participants) showed no effect in 25 of 34 comparisons. The positive effects were all from one study of 28 infants who received a daily 15-minute handling and positioning intervention or a social interaction intervention for three weeks.[49] In addition to its small sample size, this study had an unclear risk of bias. Only one study at low risk of bias examined motor development outcomes.[28] This study found no difference in the mean scores for the psychomotor scores of the Bayley Scale of Infant Development between the intervention and control groups (mean difference 1.64, 95% CI -0.94 – 4.21, p=0.21).

Language development outcomes

Ten studies including a total of 43 comparisons examined this outcome, with nine using validated tools. The total number of participants for this outcome is unknown as one study did not report numbers,[58] but was in excess of 3,000. The quality of the evidence was low. Seven studies comparing one intervention with usual care showed no effect in ten comparisons, a positive effect in four comparisons and a negative effect (poorer language development in the intervention group) in two comparisons. Three studies which compared two interventions (632 participants) found no difference between the interventions in 23 comparisons and a positive effect in 4 comparisons. Only one study at low risk of bias examined language development outcomes.[61] This study compared two different interventions with usual care (supportive health visiting [SHV, moderate intensity] and community groups [CGS, low intensity). This study found that fewer mothers in the SHV group expressed a worry about their child's speech than in the control group (risk ratio 0.46,

For peer review only - http://bmjopen.bmj.com/site/about/guidelines.xhtml

95% CI 0.23 - 0.93), and no difference in the number of number of mothers expressing worries about speech between CGS and control (risk ratio 1.22, 95% CI 0.78 - 1.92).

Cognitive development outcomes

Eight studies, including a total of 40 comparisons in 2245 participants, examined cognitive development outcomes. All used validated tools, except for one where videotaped interactions were coded for "*independent goal-directed play*".[48] The quality of the evidence was low. In five studies (1729 participants) comparing one intervention with usual care, there was no effect in 18 of 20 comparisons, and a positive effect in two comparisons. Three studies which compared two interventions (516 participants) found no difference between the interventions in 16 of 20 comparisons and a positive effect in four comparisons. Two studies at low risk of bias examined cognitive development outcomes. One study[28] found no difference in the mean scores in the intervention and control group for the mental development scores of the Bayley Scale of Infant Development (mean difference -0.81, 95% CI -2.81 – 1.16, p=0.42). The other[50] found no difference in mean scores between intervention and control on cognitive development using the British Ability Scale (mean score in intervention group -0.05 (SD 1.01) and in control group 0.03 (SD 0.99), Hedges g effect size -0.63, 95% CI -0.28 - 0.15, p=0.56).

Social and emotional wellbeing outcomes

These outcomes were examined in 156 comparisons in 18 trials (total participant numbers unknown as one study did not report participant numbers,[58] but was in excess of 5,000). Many different outcomes were examined, with most assessed using validated tools (such as the Child Behaviour Checklist, the Infant Behaviour Questionnaire, the Parent-Infant Relationship Global Assessment, the Q-Sort Measure of the Security of Attachment, and social and emotional wellbeing scores from the Ages and Stages Questionnaire).

The quality of the evidence was low. In 15 studies comparing one intervention with usual care, there was no effect in 60 of 69 comparisons. In the three studies which compared two interventions (630 participants), there was no difference between the interventions in 82 of 87 comparisons, a positive effect in four comparisons, and a negative effect in one comparison. Six studies at low risk of bias examined social and emotional wellbeing

For peer review only - http://bmjopen.bmj.com/site/about/guidelines.xhtml

outcomes, and none found a difference between intervention and control groups. The largest of these[46] found no difference in mean scores between intervention and control for externalising or internalising behaviours measured using the Child Behaviour Checklist at 3, 9 or 21 months post-intervention. For example, the adjusted mean difference for externalising behaviours at 3 months was 0.16 (95% CI -1.01 - 1.33, p=0.79), at 9 months was -0.79 (95% CI -2.27 - 0.69, p=0.30), and at 21 months was -0.80 (95% CI -2.2 - 0.6, p=0.26).

Overall child development outcomes

Four studies including a total of 12 comparisons in 1565 participants examined global estimates of child development. The quality of the evidence was moderate. Three studies (1414 participants) comparing one intervention with usual care found no effect in seven of eight comparisons based on validated measures of global child development (Griffith Mental Development Scale,[25] and mean score from the Schedule of Growing Skills II[41]). Two studies at low risk of bias examined this outcome. In one study[61], there was no difference between SHV and control (risk ratio 0.88, 95% CI 0.39-1.99) or CGS and control (risk ratio 0.57, 95% CI 0.22-1.52) in the mother's perception of whether her child's development was normal. However, mothers in the SHV group had fewer mean number of worries about their child's development than in the control group (mean difference -0.23, 95% CI -0.42 - -0.01), but there was no difference in the mean number of worries about their child's development between CGS and control (mean difference 0.13, 95% CI -0.10 - 0.36). The other study, comparing two interventions (151 participants), found no difference between the interventions in four comparisons (using the mean development score from the Ages and Stages Questionnaire).[30]

Sub-group effects reported within studies

Sub-groups examined within the individual studies included families of different incomes, and children with different characteristics (for example, low birth-weight versus normal birth-weight infants, see Tables 3 and 4). Some positive effects were seen, but the reporting of these analyses was generally incomplete, with an emphasis on positive intervention effects. No conclusions can therefore be drawn on sub-groups in this review.

For peer review only - http://bmjopen.bmj.com/site/about/guidelines.xhtml

Stratification of results across studies by risk of bias and intensity of interventions

Table 4 gives the effect direction plot, summarizing the results for each outcome, ordered by risk of bias and the intensity of the intervention. In the studies at low risk of bias, there was no intervention effect when either low or high intensity interventions were studied. Some positive effects were seen in the two trials of moderate intensity interventions, although in one, this was limited to sub-groups only (children with "disturbed" attachment at baseline),[26] and in the other, positive effects were not consistently seen.[61]

One study was classified as being at high risk of bias, and this examined a low intensity intervention.[44] Inconsistent positive intervention effects were seen in this study, with most of these in one sub-group only. The remaining studies were classified as being at unclear risk of bias, and there is no clear pattern the effects seen in these studies. Programme intensity does not appear to be associated with effectiveness in these studies, in that there is no evidence that higher intensity interventions are associated with more intervention effects.

Table 4 also summarises the uptake and adherence to intervention components. These factors were variable across studies and inconsistently reported. For example, for low intensity interventions, this ranged from only 19% of the women accessing the intervention at all (community support groups[61]) to 83% accessing every session.[25] Patterns of adherence to the moderate and high intensity interventions also varied.

No clear pattern in the results were seen when stratification by the other pre-specified variables was conducted (see Web Appendix D).

Discussion

The need for interventions to promote child development outcomes in all families has been clearly articulated. Using a broad systematic search of the extensive literature in this field, we found 22 RCTs examining the effect of interventions that enhance health service contacts from the antenatal period to 24 months postpartum. The interventions varied greatly in their content and intensity, and uptake, adherence and fidelity were not consistently reported. The quality of evidence for motor development and overall child development was moderate, and the majority of comparisons showed no intervention

For peer review only - http://bmjopen.bmj.com/site/about/guidelines.xhtml

For peer review only

effect. The quality of evidence for language development, cognitive development, and social and emotional wellbeing was low. The majority of the comparisons for these outcomes showed no effect, and where positive impacts were observed, within-study effects were inconsistent. Studies that compared one intervention with usual care did not demonstrate more positive intervention effects than studies comparing two interventions. We conclude that there is insufficient evidence to suggest that the interventions reviewed here are effective at improving child development outcomes. The low-to-moderate quality of evidence overall suggests that there is a need for high quality robust trials to inform current health service delivery in this area.

The strength of our review was the broad search strategy, which encompassed many sources of information other than database searching. We are confident that we have identified most relevant studies (including three trials not yet published in peer-review journals). Although it was not possible to conduct a meta-analysis due to the variation in the types of interventions and methods used to measure outcomes, the narrative review – supplemented with the effect direction plot - provides a comprehensive picture of the limited evidence-base in this field.

To our knowledge this is the first systematic review of interventions which aim to enhance health service contacts to improve child development outcomes, including social and emotional wellbeing outcomes. Our conclusion is consistent with other reviews of early years interventions. For example, the Allen review[9] found that none of the interventions designed for universal use in the early years (defined as conception to school) had "best" quality evidence available to support them. A recent rapid review to update the evidence for components of the Healthy Child Programme in England also found few studies of interventions aiming to promote child development outcomes in all families with children in the 0-5 age range.[10] We reviewed a larger number of primary studies than either of these previous publications. Previous studies have also examined the effects of programmes such as these on parental knowledge, attitudes or practices. We did not systematically review parental outcomes here, so cannot comment on whether parents benefitted from these interventions. However, we can conclude that – in these studies – any effects on the parents did not, in turn, lead to consistent improvements in child development outcomes.

For peer review only - http://bmjopen.bmj.com/site/about/guidelines.xhtml

Understanding how health service contacts can be enhanced to provide support for parents to achieve the best possible developmental outcomes for their children is necessary but challenging. Maternal and child health services consist of many components, many of these untested. Parents also access a wide variety of other forms of support, and the effects of these are poorly understood. Although the evidence-base examined in this review is limited, it does allow us to conclude that there is no convincing evidence that the interventions studied provide an additional benefit to the care currently provided in the settings included in these trials. There was also no evidence that interventions of high intensity confer more benefit than those of lower intensity as no dose-response relationship was evident: programmes of greater intensity (in terms of length, number or type of components) did not show more positive intervention effects than programmes of lower intensity. This is consistent with recent evidence for targeted interventions (such as the recent trial of the Family Nurse Partnership programme in the UK[67]) and has implications for commissioners of early years health services.

Many interventions currently incorporated into health services have not been adequately evaluated, and we recommend further research to generate this evidence. The methodological quality of many of the studies – or the reporting of their methods - was poor (as shown in Figures 2 and 3). Eight of 22 trials provided no detail on how their randomisation sequence was generated, and one reported using an inappropriate method. 13 provided no detail of allocation concealment, and one reported using an inappropriate method. Ten relied on parental reporting of outcomes only, and a further five used a mix of parental reporting and observations. Although blinding of outcome assessment can be a challenge in studies that rely on parental reporting of their child's development, validated measures of assessing children's development without using parental report (for example, coding of videotaped interactions as used in [26 54 55 58 69]) exist and we would encourage their use in research of this kind. 15 did not publish a protocol or provide evidence of trial registration, and two did not report on all outcomes described in the Methods section of the paper. Improvements in trial registration and a priori specification of analysis plans are needed in trials in this field.

For peer review only - http://bmjopen.bmj.com/site/about/guidelines.xhtml

We also note that adherence was poor across studies, and inconsistently reported. Future research should carefully report uptake, adherence and fidelity (particularly whether parents have received the intervention in sufficient dose), to further develop our understanding of the mechanism of action of these programmes and how to engage and retain families.[68 69] Involvement of parents from the design stage onwards is essential to improve engagement of families within these important research studies.[70] Recent work has shown that monetary incentives can also increase participant retention in RCTs.[71] Research is also needed on whether new delivery platforms (such as technology-assisted interventions[72]) may provide a more engaging, feasible and cost-effective mechanism for providing support to parents.

There have been calls for new public health models of interventions to enhance early child development within existing healthcare systems.[73] As shown in our review, however, the current evidence base for interventions delivered to all families is lacking. It is unclear from the literature reviewed why programmes had limited impact on child developmental outcomes. However, many of the interventions relied on parents to change their behaviours and action in relation to their children and were educational in tone, but did not have a theoretical framework or a sound basis in behaviour change mechanisms.[74] Additionally, authors did not always report on a clear formative research phase or logic model. Future studies should follow guidance on the development and evaluation of complex interventions (such as the Medical Research Council's guidance[75]) The results of all phases of intervention development also need to be published alongside trial results, as current studies alone do not allow us to fully understand why interventions have not produced expected effects.

Currently, there is insufficient evidence that, where health services are available to all families with very young children, additional elements or enhancements to these improve child development outcomes. Early intervention to improve child development is a public health priority, but funding is scarce. There is an urgent need for more robust evaluation of existing interventions, and to develop and evaluate novel intervention packages to enhance the offer to all families.

For peer review only - http://bmjopen.bmj.com/site/about/guidelines.xhtml

Competing interests

All authors have completed the Unified Competing Interest form at www.icmje.org/coi_disclosure.pdf (available on request from the corresponding author) and declare no financial relationships with any organisations that might have an interest in the submitted work in the previous three years, and no other relationships or activities that could appear to have influenced the submitted work.

Contributorship statement

LH, SP, PL, DW, JW and RL conceived and designed the study. MM designed the search strategy (in consultation with other review authors) and performed the database searches. LH and LJG searched the websites and journal table of contents. LH and LJG selected and reviewed eligible reports. LH, LJG, SG, SP and TP extracted data. LH drafted the paper. All authors commented on and revised the paper, and approved the final version. LH is the guarantor for the paper.

Funding

This work was funded by Public Health Wales. The Director of Policy, Research and Development at Public Health Wales (Professor Mark Bellis) provided expert technical advice during discussions on the study design, the interpretation of the results, and the drafting of the paper, and is an author on the paper.

Data sharing statement

This paper reports on a systematic review. No original study data was obtained from the authors of the trials included in the review. Extra data (such as the results of the review stratified by characteristics other than those included in the report) is available by emailing Lisa Hurt.

For peer review only

References

1. Marmot M, Allen J, Goldblatt P, et al. Fair Society, Healthy Lives: The Marmot Review. London: University College London, 2010.
2. Rough E, Goldblatt P, Marmot M, et al. Inequalities in child health. In: Foyle G, Nathanson V, eds. Growing up in the UK - Ensuring a healthy future for our children. London: British Medical Association, 2013:37-56.
3. Shonkoff, J, Phillips, D (eds). *From Neurons to Neighborhoods: The Science of Early Childhood Development.* Washington, DC.: National Academy Press, 2000.
4. Stewart-Brown S, Fletcher L, Wadsworth M. Parent-child relationships and health problems in adulthood in three UK national birth cohort studies. European Journal of Public Health 2005;**15**(6):640-6.
5. World Health Organization. The Minsk Declaration: The Life-Course Approach in the Context of Health 2020. Denmark: WHO Regional Office for Europe, 2015.
6. Irwin L, Siddiqui A, Hertzman C. Early Child Development: A Powerful Equalizer. Final Report for the World Health Organization's Commission on the Social Determinants of Health. Vancouver, Canada: Human Early Learning Partnership, University of British Columbia., 2007.
7. Jensen B, Currie C, Dyson A, et al. European Review of Social Determinants of Health and the Health Divide in the WHO European Region. Copenhagen, Denmark: WHO Regional Office for Europe, 2013.
8. DiMario S, Basevi V, Gori G, et al. What is the effectiveness of antenatal care? (Supplement). Health Evidence Network report. Copenhage, Denmark: WHO Regional Office for Europe, 2005.
9. Allen G. Early Intervention: The Next Steps. Londo, UK: Cabinet Office, 2011.
10. Axford N, Barlow J, Coad J, et al. Rapid Review to Update Evidence for the Healthy Child Programme 0–5. London, UK: Public Health England, 2015.
11. Barlow J, Smailagic N, Ferriter M, et al. Group-based parent-training programmes for improving emotional and behavioural adjustment in children from birth to three years old. Cochrane Database of Systematic Reviews 2010;**Issue 3.**:Art. No.: CD003680.
12. Britto P. School Readiness: a conceptual framework. New York, USA: United Nations Children’s Fund, 2012.

For peer review only

For peer review only - http://bmjopen.bmj.com/site/about/guidelines.xhtml

13. Sanders M, Kirby J. A Public-Health Approach to Improving Parenting and Promoting Children's Well-Being. Child Development Perspectives 2014;**8**(4):250-7.

14. Bayer J, Hiscock H, Morton-Allen E, et al. Prevention of mental health problems: rationale for a universal approach. Archives of Diseases in Childhood 2007;**92**:34-8.

15. Prinz R, Sanders M. Adopting a population-level approach to parenting and family support interventions. Clinical Psychology Review 2007;**27**(6):739-49.

16. Britto P, Lye S, Proulx K, et al. Nurturing care: promoting early childhood development. Lancet 2017;**389**:91-102.

17. Walker S, Wachs T, Grantham-McGregor S, et al. Inequality in early childhood: risk and protective factors for early child development. Lancet 2011;**378**:1325–38.

18. Nutley S, Walter I, Davies H. *Using evidence: How research can inform public services.* Bristol: Policy Press, University of Bristol, 2007.

19. Liberati A, Altman D, Tetzlaff J, et al. The PRISMA statement for reporting systematic reviews and meta-analyses of studies that evaluate health care interventions: explanation and elaboration. PLoS Med 2009;**6**(7):e1000100.

20. Higgins J, Green S, editors. *Cochrane Handbook for Systematic Reviews of Interventions Version 5.1.0 [updated March 2011].* The Cochrane Collaboration, 2011.

21. Baker P, Francis D, Soares J, et al. Community wide interventions for increasing physical activity. Cochrane Database of Systematic Reviews 2015;**Issue 1**:Art. No.: CD008366.

22. Guyatt G, Oxman A, Akl E, et al. GRADE guidelines: 1. Introduction—GRADE evidence profiles and summary of findings tables Journal of Clinical Epidemiology 2011;**64**(4):383–94.

23. Bayer J, Hiscock H, Ukoumunne O, et al. Three-year-old outcomes of a brief universal parenting intervention to prevent behaviour problems: randomised controlled trial. Archives of Diseases in Childhood 2010;**95**:187–92.

24. Beeghly M, Brazelton T, Flannery K, et al. Specificity of preventative pediatric intervention effects in early infancy. Developmental and Behavioral Pediatrics 1995;**16**(3):158-66.

25. Chang S, Grantham-McGregor S, Powell C, et al. Integrating a Parenting Intervention With Routine Primary Health Care: A Cluster Randomized Trial. Pediatrics 2015;**136**(2):272-80.

For peer review only - http://bmjopen.bmj.com/site/about/guidelines.xhtml

26. Cheng S, Kondo N, Aoki Y, et al. The effectiveness of early intervention and the factors related to child behavioural problems at age 2: A randomized controlled trial. Early Human Development 2007;**83**:683–91.

27. Christakis D, Zimmerman F, Garrison M. Effect of Block Play on Language Acquisition and Attention in Toddlers. A Pilot Randomized Controlled Trial. Arch Pediatr Adolesc Med 2007;**161**(10):967-71.

28. Cupples M, Stewart M, Percy A, et al. A RCT of peer-mentoring for first-time mothers in socially disadvantaged areas (The MOMENTS Study). Archives of Diseases in Childhood 2011;**96**:252–58.

29. Doyle O. Barrington Lecture 2011-12. Breaking the Cycle of Deprivation: An Experimental Evaluation of an Early Childhood Intervention. Journal of the Statistical and Social Inquiry Society of Ireland 2012;**XLI**:92-111.

30. Doyle, O. and the Preparing for Life Evaulation Team. Preparing for Life: Early Childhood Intervention. Assessing the Early Impact of Preparing for Life at Six Months. Dublin, Ireland: UCD Geary Institute, 2011.

31. Doyle, O. and the Preparing for Life Evaulation Team. Preparing For Life: Early Childhood Intervention. Assessing the Impact of Preparing For Life at Twelve Months. Dublin, Ireland: UCD Geary Institute, 2012.

32. Doyle, O. and the Preparing for Life Evaulation Team. Preparing For Life:Early Childhood Intervention. Assessing the Impact of Preparing For Life at Eighteen Months. Dublin, Ireland: UCD Geary Institute, 2013.

33. Doyle, O. and the Preparing for Life Evaulation Team. Preparing For Life: Early Childhood Intervention. Assessing the Impact of Preparing For Life at Twenty-four Months. Dublin, Ireland: UCD Geary Institute, 2013.

34. Doyle, O. and the Preparing for Life Evaulation Team. Preparing For Life: Early Childhood Intervention. Assessing the Impact of Preparing For Life at Thirty-six Months. Dublin, Ireland: UCD Geary Institute, 2014.

35. Doyle O, McGlanaghy E, O'Farrelly C, et al. Can Targeted Intervention Mitigate Early Emotional and Behavioral Problems?: Generating Robust Evidence within Randomized Controlled Trials. PLOS One 2016;**11**(6):e0156397.

For peer review only

For peer review only - http://bmjopen.bmj.com/site/about/guidelines.xhtml

36. Drotar D, Robinson J, Jeavons L, et al. A randomized, controlled evaluation of early intervention: the Born to Learn curriculum. Child: Care, Health and Development 2008;**35**(5):643–49.

37. Feinberg M, Kan M. Establishing Family Foundations: Intervention Effects on Coparenting, Parent/Infant Well-Being, and Parent–Child Relations. Journal of Family Psychology 2008;**22**(2):253–63.

38. Feinberg M, Kan M, Goslin M. Enhancing Coparenting, Parenting, and Child Self-Regulation: Effects of Family Foundations 1 Year after Birth. Prevention Science 2009;**10**:276–85.

39. Feinberg M, Jones D, Kan M, et al. Effects of Family Foundations on Parents and Children: 3.5 Years After Baseline. Journal of Family Psychology 2010;**24**:532–42.

40. Feinberg M, Jones D, Roettger M, et al. Long-Term Follow-Up of a Randomized Trial of Family Foundations: Effects on Children's Emotional, Behavioral, and School Adjustment. Journal of Family Psychology 2014;**28**(6):821–31.

41. Griffith N. Evaluating the Incredible Years Toddler programme in disadvantaged areas of Wales. Bangor University, 2011.

42. Gridley N, Hutchings J, Baker-Henningham H. The Incredible Years Parent–Toddler Programme and parental language: a randomised controlled trial. Child: Care, Health and Development 2015;**41**(1):103–11.

43. Guyer B, Hughart N, Strobino D, et al. Assessing the Impact of Pediatric-Based Developmental Services on Infants, Families, and Clinicians: Challenges to Evaluating the Healthy Steps Program. Pediatrics 2000;**105**(3):e33.

44. High P, LaGasse L, Becker S, et al. Literacy Promotion in Primary Care Pediatrics: Can We Make a Difference? Pediatrics 2000;**105**(4):927–34.

45. Hiscock H, Bayer J, Wake M. Preventing Toddler Externalising Behaviour Problems: Pilot Evaluation of a Universal Parenting Program. International Journal of Mental Health Promotion 2005;**7**(2):54-60.

46. Hiscock H, Bayer J, Price A, et al. Universal parenting programme to prevent early childhood behavioural problems: cluster randomised trial. British Medical Journal 2008;**336**:318.

For peer review only - http://bmjopen.bmj.com/site/about/guidelines.xhtml

47. Johnston B, Huebner C, Anderson M, et al. Healthy Steps in an Integrated Delivery System. Child and Parent Outcomes at 30 Months. Arch Pediatr Adolesc Med 2006;**160**:793-800.

48. Landry S, Smith K, Swank P. Responsive Parenting: Establishing Early Foundations for Social, Communication, and Independent Problem-Solving Skills. Developmental Psychology 2006;**42**(4):627–42.

49. Lobo M, Galloway J. Enhanced Handling and Positioning in Early Infancy Advances Development Throughout the First Year. Child Development 2012;**83**(4):1290–302.

50. Miller S, Dunne L. A Randomised Controlled Trial Evaluation of the Lifestart Parenting Programme. Belfast, Northern Ireland: Queen's University, 2015.

51. Minkovitz C, Strobino D, Hughart N, et al. Early effects of the Healthy Steps for Young Children Program. Arch Pediatr Adolesc Med 2001;**155**:470-79.

52. Minkovitz C, Hughart N, Strobino D, et al. A practice-based intervention to enhance quality of care in the first 3 years of life. The Healthy Steps for Young Children Program Journal of the American Medical Association 2003;**290**(23):3081-91.

53. Zuckerman B, Parker S, Kaplan-Sanoff M, et al. Healthy Steps: A Case Study of Innovation in Pediatric Practice. Pediatrics 2004;**114**(3):820–26.

54. Niccols A. 'Right from the Start': randomized trial comparing an attachment group intervention to supportive home visiting. Journal of Child Psychology and Psychiatry 2008;**49**(7):754–64.

55. Niccols A. Immediate and short-term outcomes of the 'COPEing with Toddler Behaviour' parent group. Journal of Child Psychology and Psychiatry 2009;**50**(5):617–26.

56. Santelices M, Guzmán M, Aracena M, et al. Promoting secure attachment: evaluation of the effectiveness of an early intervention pilot programme with mother–infant dyads in Santiago, Chile. Child: Care, Health and Development 2010;**37**(2):203–10.

57. Tsiantis J, Dragonas T, Cox A, et al. Promotion of children's early psychosocial development through primary health care services. Paediatric and Perinatal Epidemiology 1996;**10**:339-54.

58. Tsiantis J, Smith M, Dragonas T, et al. Early Mental Health Promotion in Children Through Primary Health Care Services: A Multi-Centre Implementation. Journal of Public Mental Health 2000;**2**(3):5-15.

For peer review only

For peer review only - http://bmjopen.bmj.com/site/about/guidelines.xhtml

59. Wagner M, Spiker D, Hernandez F, et al. Multisite Parents as Teachers evaluation. Experiences and outcomes for children and families. California, USA: SRI International, 2001.

60. Wagner M, Spiker D, Linn M. The Effectiveness of the Parents as Teachers Program with Low-Income Parents and Children. Topics in Early Childhood Special Education 2002;**22**(2):67-81.

61. Wiggins M, Oakley A, Roberts I, et al. The Social Support and Family Health Study: a randomised controlled trial and economic evaluation of two alternative forms of postnatal support for mothers living in disadvantaged inner-city areas. Health Technology Assessment 2004;**8**(32):1-134.

62. Wiggins M, Oakley A, Roberts I, et al. Postnatal support for mothers living in disadvantaged inner city areas: a randomised controlled trial. J Epidemiol Community Health 2005;**59**:288–95.

63. Hiscock H, Bayer J, Lycett K, et al. Preventing mental health problems in children: the Families in Mind population-based cluster randomised controlled trial. BMC Public Health 2012;**12**:420.

64. Pontoppidan M. The effectiveness of the Incredible Years™ Parents and Babies Program as a universal prevention intervention for parents of infants in Denmark: study protocol for a pilot randomized controlled trial. Trials 2015;**16**:386.

65. Sawyer A, Lynch J, Bowering K, et al. An equivalence evaluation of a nurse-moderated group-based internet support program for new mothers versus standard care: a pragmatic preference randomised controlled trial. BMC Pediatrics 2014;**14**:119.

66. Thomson H, Thomas S. The effect direction plot: visual display of non-standardised effects across multiple outcome domains. Res Syn Meth 2013;**4**:95–101.

67. Robling M, Bekkers M-J, Bell K, et al. Effectiveness of a nurse-led intensive home-visitation programme for first-time teenage mothers (Building Blocks): a pragmatic randomised controlled trial. The Lancet 2016;**387**(10014):146–55.

68. Ingoldsby I. Review of interventions to improve family engagement and retention in parent and child mental health programs. J Child Fam Stud 2010;**19**(5):629–45.

69. Cullen S, Cullen M-A, Lindsay G. Universal parenting programme provision in England; Barriers to parent engagement in the CANparent trial, 2012-2014. Children and Society 2016;**30**:71-81.

For peer review only - http://bmjopen.bmj.com/site/about/guidelines.xhtml

70. Hutton E, Coxon K. Involving parents as service users in an interprofessional research project. Journal of Interprofessional Care 2008;**22**(6):661-63.

71. Brueton V, Tierney J, Stenning S, et al. Strategies to improve retention in randomised trials: a Cochrane systematic review and meta-analysis. BMJ Open 2014;**4**:e003821.

72. Hall C, Bierman K. Technology-assisted interventions for parents of young children: Emerging practices, current research, and future directions. Early Childhood Research Quarterly 2015;**33**:21-32.

73. Reilly S, McKean C, Morgan A, et al. Identifying and managing common childhood language and speech impairments. BMJ 2015;**350**:h2318.

74. Michie S, van Stralen M, West R. The behaviour change wheel: A new method for characterising and designing behaviour change interventions. Implement Sci 2011;**6**:42.

75. Craig P, Dieppe P, Macintyre S, et al. Developing and evaluating complex interventions: the new Medical Research Council guidance. BMJ 2008;**337**:a1655.

For peer review only - http://bmjopen.bmj.com/site/about/guidelines.xhtml

Table 1: Characteristics of the included studies

Study<br>Setting	Type and aim of study	Comparison group	i) Who received intervention, and<br>ii) When?	Sample size<br>i) Randomised<br>ii) In analysis (% of randomised)	Universal or geographically targeted?	Outcome domains measured*
**Beeghly 1995**<br>USA	Individual RCT, to compare the effectiveness of two one-to-one clinic-based interventions (infant-centred vs mother centred) on motor and cognitive development post-intervention	Two interventions compared	i) Mothers and infant<br>ii) When child was 3, 14 and 30 days of age	i) 163<br>ii) 125 (77%)	Universal	Motor<br>Cognitive
**Chang 2015**<br>Antigua, Jamaica & St Lucia	Cluster RCT, to examine the effectiveness of a group-based intervention on language and cognitive development post-intervention	Usual care	i) Mothers<br>ii) When child was 3, 6, 9, 12 and 18 months	i) 30 health centres randomised (501 women enrolled)<br>ii) 426 (85%)	Geographically targeted	Motor<br>Language<br>Cognitive<br>Overall
**Cheng 2007**<br>Japan	Individual RCT, to examine the effectiveness of an individual home-based intervention on social and emotional wellbeing post-intervention	Usual care, with a counselling service made available to all	i) Mothers<br>ii) When child was 5-9 months	i) 95<br>ii) 85 (89%)	Universal	SEWB
**Christakis 2007**<br>USA	Individual RCT, to examine the effectiveness of giving 2 sets of building blocks and a newsletter of activities to complete with them on language development and social and emotional wellbeing post-intervention	Usual care	i) Families<br>ii) When child was 18-30 months	i) 175<br>ii) 140 (80%)	Universal	Language<br>SEWB
**Cupples 2011**<br>UK (Northern Ireland)	Individual RCT, to examine the effectiveness of one-to-one contact with trained peer mentors on motor, cognitive development and social and emotional wellbeing post-intervention	Usual care	i) Mothers<br>ii) From 20 weeks of pregnancy to 12 months postpartum	i) 343<br>ii) 280 (82%)	Geographically targeted (First time mothers only)	Motor<br>Cognitive<br>SEWB
**Doyle 2011**<br>Ireland	Individual RCT, to compare the effectiveness of the "high support" versus "low support" versions of the multi-component "Preparing for Life" programme on motor, language, and cognitive development and social and emotional wellbeing whilst intervention was ongoing	Two interventions compared	i) Parents<br>ii) From pregnancy to school entry	i) 233<br>ii) 173 (74% at 6 months)<br>165 (71% at 12 months)<br>154 (66% at 18 months)<br>166 (71% at 24 months)<br>151 (65% at 36 months)	Geographically targeted	Motor<br>Language<br>Cognitive<br>SEWB<br>Overall
**Drotar 2008**<br>USA	Individual RCT, to examine the effectiveness of the multi-component "Born to Learn" programme on language and cognitive development and social and emotional wellbeing whilst intervention was ongoing	Usual care, plus handouts and offer of a different group meeting	i) Parents and child<br>ii) Recruited between birth and 9 months; programme continued to age 3	i) 527<br>ii) 410 (78%, although inconsistent numbers presented in tables)	Universal	Language<br>Cognitive†<br>SEWB
**Feinberg 2008**<br>USA	Individual RCT, to examine the effectiveness of a group-based intervention ("Family Foundations") on social and emotional wellbeing post-intervention	Usual care plus brochure on childcare options	i) Parents<br>ii) Recruited during pregnancy, continued to age 4-6 months	i) 169<br>ii) 152 (90% at 6 months)<br>154 (91% at 12 months)<br>137 (81% at 36 months)	Universal (First time mothers only)	SEWB†

For peer review only - http://bmjopen.bmj.com/site/about/guidelines.xhtml

Study<br>Setting	Type and aim of study	Comparison group	i) Who received intervention, and<br>ii) When?	Sample size<br>i) Randomised<br>ii) In analysis (% of randomised)	Universal or geographically targeted?	Outcome domains measured*
**Griffith 2011**<br>UK (Wales)	Individual RCT, to examine the effectiveness of a group-based parenting intervention ("Incredible Years Toddler Programme") on social and emotional wellbeing and overall development post-intervention	Usual care (waiting list control group)	i) Parents<br>ii) Children 12-36 months at baseline (mean age 21 months)	i) 89<br>ii) 89 (100%)	Geographically targeted	SEWB<br>Overall
**High 2000**<br>USA	Individual RCT, to examine the effectiveness of a one-to-one clinic-based intervention on language development post-intervention	Usual care	i) Parents<br>ii) Children 5-11 months at baseline	i) 205<br>ii) 153 (75%)	Geographically targeted	Language
**Hiscock 2008**<br>Australia	Cluster RCT, to examine the effectiveness of a group-based intervention ("Toddlers Without Tears") on social and emotional wellbeing post-intervention	Usual care	i) Parents<br>ii) When child was 8, 12 and 15 months	i) 40 maternal and child health centres randomised (733 women enrolled)<br>ii) 672 (92% at 18 months)<br>656 (89% at 24 months)<br>589 (80% at 36 months)	Universal	SEWB
**Johnston 2006**<br>USA	Individual RCT, to compare the effectiveness of the multi-component "Healthy Steps" programme with "Healthy Steps" plus "PrePare" on language development and social and emotional wellbeing post-intervention	Two interventions compared	i) Mothers<br>ii) Recruited during pregnancy, continued to age 3	i) 303<br>ii) 239 (79%)	Universal	Language<br>SEWB
**Landry 2006**<br>USA	Individual RCT, to compare the effectiveness of two different models of home visits, on language and cognitive development and social and emotional wellbeing post-intervention	Two interventions compared	i) Mothers<br>ii) When child was 6-10 months	i) 264<br>ii) 240 (91%)	Geographically targeted	Language‡<br>Cognitive‡<br>SEWB‡
**Lobo 2012**<br>USA	Individual RCT, to compare the effectiveness of a "handling and positioning" intervention with a "social interaction" intervention on motor development whilst the intervention was ongoing and post-intervention	Two interventions compared	i) Parents<br>ii) For 3 weeks, from when child was 2 months of age	i) 28<br>ii) 28 (100%)	Universal	Motor
**Miller 2015**<br>Ireland	Individual RCT, to examine the effectiveness of the multi-component "Lifestart" programme on cognitive development and social and emotional wellbeing whilst intervention was ongoing	Usual care	i) Parents<br>ii) Recruited when child < 12 months, continued to age 5	i) 435<br>ii) 347 (80% at 36 months)	Universal (although parents self-referred)	Cognitive<br>SEWB
**Minkovitz 2003**<br>USA	Individual RCT, to examine the effectiveness of the multi-component "Healthy Steps" programme on social and emotional wellbeing in the long term whilst intervention was ongoing	Usual care	i) Families<br>ii) Recruited at birth or 1st well-child visit, continued to age 3	i) 2235<br>ii) 1593 (71%)	Universal	SEWB

For peer review only - http://bmjopen.bmj.com/site/about/guidelines.xhtml

Study<br>Setting	Type and aim of study	Comparison group	i) Who received intervention, and<br>ii) When?	Sample size<br>i) Randomised<br>ii) In analysis	Universal or geographically targeted?	Outcome domains measured*
**Niccols 2008**<br>Canada	Individual RCT, to examine the effectiveness of a group-based intervention ("Right from the Start") on social and emotional wellbeing post-intervention	Usual care	i) Mothers<br>ii) Children 1-24 months at baseline	i) 76<br>ii) 73 (96% immediately post intervention)<br>64 (84% at 6 months)	Universal (although parents self-referred)	SEWB
**Niccols 2009**<br>Canada	Individual RCT, to examine the effectiveness of a group-based intervention ("COPEing with Toddler Behaviour") on social and emotional wellbeing post-intervention	Usual care (waiting list control group)	i) Mothers<br>ii) Children 12-36 months at baseline (mean age 24 months)	i) 79<br>ii) 74 (94% immediately post-intervention)<br>71 (90% at 1 month)	Universal (although parents self-referred)	SEWB†
**Santelices 2011**<br>Chile	Individual RCT, to examine the efficacy of a multi-component intervention ("Promoting Secure Attachment") on social and emotional wellbeing post-intervention	Usual care, plus one lecture by a psychologist	i) Mothers<br>ii) Recruited during late pregnancy, continued to age 1	i) 100<br>ii) 72 (72%)	Universal (First time mothers only)	SEWB
**Tsiantis 2000**<br>Cyprus, Greece, Portugal, Yugoslavia	Cluster RCT, to examine the effectiveness of training primary health care workers to use semi-structured interviews to promote language development and social and emotional wellbeing whilst intervention is ongoing	Usual care (healthcare workers in this group received one lecture)	i) Mothers<br>ii) Recruited during pregnancy, continued to age 2	i) 80 primary healthcare workers (number of women randomised not reported, "recruitment did not achieve target figures")<br>ii) Not reported	Universal	Language<br>SEWB†
**Wagner 2001**<br>USA	Individual RCT, to examine the effectiveness of a multi-component intervention ("Parents as Teachers") on motor, language, and cognitive development and social and emotional wellbeing whilst intervention is ongoing	Usual care, plus age-appropriate toys "at regular intervals" and an annual child assessment	i) Mothers<br>ii) Recruited during pregnancy, continued to age 2	i) 665<br>ii) 266 (40%)	Geographically targeted	Motor<br>Language<br>Cognitive<br>SEWB
**Wiggins 2004**<br>UK (England)	Individual RCT, to examine the effectiveness of two postnatal social support interventions ("Support Health Visitors" [SHV] and "Community Group Support" [CGS]) on language development, social and emotional wellbeing overall development post-intervention	Both interventions compared with usual care	i) Mothers<br>ii) Recruited when child was ~ 10 weeks, continued to age 1	i) 731 (SHV 183, CGS 184, control 364)<br>ii) SHV comparison:<br>493 (91% at 12 months)<br>443 (81% at 18 months)<br>CGS comparison:<br>492 (90% at 12 months)<br>456 (83% at 18 months)	Geographically targeted	Language§<br>SEWB§<br>Overall§

Abbreviations: RCT = randomised controlled trial; SEWB = social and emotional wellbeing
* Used a validated questionnaire for measuring outcome unless indicated otherwise (although the use of the instrument may not always have been validated in the target population)
† Used a combination of validated questionnaires and coding of videotaped activities and behaviours (no validated coding framework described)
‡ Used coding of videotaped activities and behaviours (no validated coding framework described)
§ No validated measure used; asked parents whether they perceived their child's development to be normal and whether they had worries about specific areas of development (including speech and behaviour)

For peer review only - http://bmjopen.bmj.com/site/about/guidelines.xhtml

Table 2: Description of intervention components and intensity

Study	Description	Components*										Contacts: Number Frequency Duration	Who delivered the intervention?
		1	2	3	4	5	6	7	8	9	10		
**2a. Studies comparing one intervention with usual care**													
**Low intensity**													
**Chang 2015**	3 three-minute films demonstrating *"behaviours central to promoting child development*" shown as women waited for 3, 6, 9, 12 and 18 month vaccine visits, followed by group discussions with a community health worker. Cards given to reinforce messages, plus picture book at 9 and 12 months and puzzle at 18 months.			✓	✓			✓			✓	5<br>Every 3-6 months<br>Over 15 months	Health professionals
**Christakis 2007**	Parents were sent 2 sets of building blocks with accompanying newsletters containing suggested activities in the post				✓	✓		✓				2<br>Bi-monthly<br>3 months	No contact with families post-recruitment
**High 2000**	Paediatricians gave books, handouts and "*literacy promoting anticipatory guidance*" to parents at routine well-child visits		✓		✓			✓				5<br>Every 3 months<br>Over 12 months	Health professionals
**Hiscock 2008**	"*Universal anticipatory guidance*" with strategies for behavioural difficulties: handout at 8 months; 2 two-hour group sessions at 12 and 15 months			✓	✓							3<br>Every 3-4 months<br>Over 7 months	Health professionals
**Wiggins 2004 Intervention 2**	Access given to mothers to community group support that already existed and which provided drop in sessions and/or telephone support and/or home visits (different services provided by each of the 8 groups who agreed to take part in the study); participants selected whether to make contact and attend groups	✓		✓					✓			Variable	Other professionals
**Moderate intensity**													
**Cheng 2007**	5 one-to-one home visits of one hour "*aimed at improving the quality of mother-infant relationship*"; tailored encouragement and advice given following observation of mothers playing with infants	✓										5<br>Monthly<br>Over 5 months	Health professionals
**Feinberg 2008**	Four prenatal and four postnatal interactive group sessions, designed "*to enhance coparenting*"			✓								8<br>Every 6 weeks<br>Over 11 months	Mix of health and other professionals
**Niccols 2008**	8 two-hour group sessions using a "*coping modeling problem solving approach*", to enhance caregiver skills in "*reading infant cues and responding sensitively*" plus homework			✓								8<br>Weekly<br>Over 2 months	Other professionals

* 1 = One-to-one home visits, 2 = One-to-one clinic visits, 3 = Group sessions, 4 = Handouts, 5 = Activities to perform at home, 6 = Developmental screening, 7 = Toys and/or books, 8 = Telephone support, 9 = Access to community resources, 10 = Other

For peer review only - http://bmjopen.bmj.com/site/about/guidelines.xhtml

Study	Description	Components*										Contacts: Number Frequency Duration	Who delivered the intervention?
		1	2	3	4	5	6	7	8	9	10		
Moderate intensity													
**Niccols 2009**	8 two-hour group sessions, using a "*coping modeling problem solving approach*", to train parents on effective parenting styles and strategies, plus homework.			✓		✓						8<br>Weekly<br>Over 2 months	Other professionals
**Santelices 2011**	6 two-hour group sessions during pregnancy on "*maternal sensitivity...and to promote the development of a secure and healthy bond between mother and child*", and 4 hour-long one-to-one sessions postpartum to observe interactions and give feedback	✓	✓	✓								10<br>Variable<br>Over 16 months	Other professionals
**Tsiantis 2000**	Primary health care workers trained to use a semi-structured interview technique during 6-8 routine visits to discuss age-appropriate child development topics.		✓									6-8<br>Variable<br>Over 36 months	Health professionals
**Wiggins 2004 Intervention 1**	Supportive home visits conducted postnatally by five very experienced health visitors, adapted to each woman's needs to address her concerns and questions	✓										12<br>Monthly<br>Over 12 months	Health professionals
High intensity													
**Cupples 2011**	Trained peer mentors provided one-to-one support on "health-related" topics via home visit or phone call	✓							✓			22<br>Every 2-4 weeks<br>Over 17 months	Peer mentors
**Drotar 2008**	One-to-one home visits; monthly parent group sessions; annual developmental and health screening; access to resource network	✓		✓			✓			✓		27<br>Every 2-4 weeks<br>Over 36 months	Other professionals
**Griffith 2011**	12 two-hour group sessions including watching videos, group discussions and role play, to help understand and manage child behaviour; homework tasks to complete.			✓		✓						12<br>Weekly<br>Over 3 months	Mix of health and other professionals
**Miller 2015**	30-60 minute monthly home visit by a Lifestart family visitor, and a monthly magazine ("Growing Child") of age-appropriate activities	✓			✓							35<br>Monthly<br>Over 36 months	Other professionals
**Minkovitz 2003**	Enhanced well-child care (12 visits, including access to Reach Out and Read literacy programme); 6 home visits in 3 years; telephone line; developmental screening; written guidance; monthly parent groups; links to community resources	✓	✓	✓	✓		✓		✓	✓		16<br>Variable<br>Over 36 months	Mix of health and other professionals
**Wagner 2001**	Monthly home visits and parent group meetings to provide information on child development and demonstrate age-appropriate activities. Periodic developmental screening and, if needed, referrals to community services provided.	✓		✓			✓			✓		24<br>Monthly<br>Over 24 months	Other professionals

* 1 = One-to-one home visits, 2 = One-to-one clinic visits, 3 = Group sessions, 4 = Handouts, 5 = Activities to perform at home, 6 = Developmental screening, 7 = Toys and/or books, 8 = Telephone support, 9 = Access to community resources, 10 = Other

For peer review only

Study	Description	Components*										Contacts: Number Frequency Duration	Who delivered the intervention?
		1	2	3	4	5	6	7	8	9	10		
**2b. Studies comparing two interventions with each other**													
**Beeghly 1995** **1: Low intensity**	3 individual 45 minute sessions where mother observed a Neonatal Behavioral Assessment (NBAS) and discussed findings with a paediatrician (including exploring the care-giving that might promote the mother-child relationship)		✓									3 At 3, 14 and 30 days old	Health professionals
**Beeghly 1995** **2: Low intensity**	3 individual 45 minute sessions, where mother discussed her perceptions of motherhood and concerns with a paediatrician, and was given feedback about an NBAS that was conducted in a different room		✓									3 At 3, 14 and 30 days old	
**Lobo 2012** **1: Moderate intensity**	Parents taught a positioning and handling programme during a home visit by a physiotherapist to be completed for 15 minutes daily for 3 weeks. 6 assessment home visits also completed. Caregivers given manual and a session diary.	✓			✓	✓						6 Every 2 weeks Over 3 months	Intervention delivered by parents after training
**Lobo 2012** **2: Moderate intensity**	Parents asked to engage their child in 15 minutes of face-to-face interaction daily for 3 weeks. This group also had 6 assessment visits.	✓										6 Every 2 weeks Over 3 months	
**Doyle 2011** **1: Moderate intensity**	Access to a support worker; annual packs containing toys and books (worth 100 Euros); facilitated access to one year of preschool; stress control and healthy eating sessions		✓	✓ (2)				✓		✓	✓	Variable Variable Over 36+ months	Other professionals
**Doyle 2011** **2: High intensity**	Home visits from a trained mentor; tip sheets; Triple P Positive Parenting group sessions; baby massage; annual packs containing toys and books (worth 100 Euros); facilitated access to one year of preschool; stress control and healthy eating sessions	✓		✓ (3)	✓			✓		✓	✓	Variable Weekly Over 36+ months	
**Johnson 2006** **Intervention 1: High intensity**	Enhanced well-child care (6 visits, including Reach Out and Read literacy programme); 6 home visits in 3 years; telephone line; developmental screening; written guidance; monthly parent groups; links to community resources	✓	✓	✓	✓		✓		✓	✓		Variable Monthly Over 36+ months	Health professionals
**Johnson 2006** **Intervention 2: High intensity**	As above, plus 3 additional home visits during 2nd half of pregnancy	✓	✓	✓	✓		✓		✓	✓		Variable Monthly Over 36+ months	
**Landry 2006** **Intervention 1: High intensity**	Playing and Learning Strategies: One-to-one home visits of 1.5 hours to discuss the child's current development and behaviour, feedback on videotaped interactions with child; and planning with mothers of how to increase their "*responsive*" behaviours	✓										10 Weekly Over 3 months	Other professionals
**Landry 2006** **Intervention 2: High intensity**	Developmental Assessment Screening: One-to-one home visits of 1.5 hours consisting of developmental screening and discussions on child development. Handouts on common issues (e.g. sleep, feeding) given.	✓			✓		✓					10 Weekly Over 3 months	

* 1 = One-to-one home visits, 2 = One-to-one clinic visits, 3 = Group sessions, 4 = Handouts, 5 = Activities to perform at home, 6 = Developmental screening, 7 = Toys and/or books, 8 = Telephone support, 9 = Access to community resources, 10 = Other

For peer review only - http://bmjopen.bmj.com/site/about/guidelines.xhtml

Table 3: Summary of Findings

**Population:** Parents from antenatal period to 2 years postpartum<br>**Settings:** Universal programmes offered within defined populations<br>**Intervention:** Programmes to improve child development outcomes by enhancing health professional contact<br>**Comparison:** Usual care, or two different interventions compared		
**Number of studies**<br>**Total number of participants***<br>**Total number of comparisons**†	**Results**	**Quality of the evidence**
Outcome: Motor Development		
**Comparison:** Intervention with usual care		
3 studies[22 25 56]<br>972 participants, 3 comparisons	**No effect** in the 3 comparisons.<br>**No effect** in the 2 sub-groups examined in 1 study.	MODERATE (downgraded one level because of risk of bias)
**Comparison:** Two interventions		
3 studies[21 27 46]<br>304 participants, 34 comparisons (20 in Doyle, 13 in Lobo)	**No difference** in 25 comparisons; **better outcomes in the more intensive intervention group** in 9 comparisons (all in 1 study).<br>**No difference** in the 2 sub-groups examined in 1 study.	MODERATE (downgraded one level because of risk of bias)
Outcome: Language Development		
**Comparison:** Intervention with usual care		
7 studies[‡ 22 24 33 41 54 56 58]<br>Participant numbers not known[§], 16 comparisons (6 in High)	**No effect** in 10 comparisons; **better outcomes in intervention than control** in 4 comparisons (2 studies); **worse outcomes in intervention than control group** in 2 tests (1 study).<br>Sub-group effects reported in 4 studies, with some better outcomes in intervention than control, but reporting of sub-groups unclear and incomplete.	LOW (downgraded two levels because of risk of bias and inconsistency[††])
**Comparison:** Two interventions		
3 studies[27 44 45]<br>632 participants, 27 comparisons (21 in Doyle)	**No difference between the two interventions** in 23 comparisons; **better outcomes in the more intensive intervention group** in 4 tests (2 studies). No sub-group effects examined.	LOW (downgraded two levels because of risk of bias and inconsistency[††])
Outcome: Cognitive Development		
**Comparison:** Intervention with usual care		
5 studies[22 25 33 47 56]<br>1729 participants, 20 comparisons (16 in Drotar)	**No effect** in 18 comparisons; **better outcomes in intervention than control** in 2 comparisons (2 studies). Sub-group effects examined in 2 studies, with some better outcomes in intervention than controls seen, but reporting of sub-groups unclear and incomplete.	LOW (downgraded two levels because of risk of bias and inconsistency[††])
**Comparison:** Two interventions		
3 studies[21 27 45]<br>516 participants, 20 comparisons (18 in Doyle)	**No difference between the two interventions** in 16 comparisons; **better outcomes in the more intensive intervention group** in 4 comparisons (2 studies).<br>**No significant interaction** in the 4 tests performed in 1 study.	LOW (downgraded two levels because of risk of bias and inconsistency[††])

For peer review only - http://bmjopen.bmj.com/site/about/guidelines.xhtml

Outcome: Social and emotional wellbeing		
**Comparison:** Intervention with usual care		
15 studies[‡ 23-25 33 34 38 43 47 49 51-54 56 58]<br>Participant numbers not known[§], 69 comparisons (7 in Drotar, 14 in Feinberg, 8 in Niccols 2009, 16 in Tsiantis, 5 in Wagner)	**No effect** in 60 comparisons ([**]in 1 study); **better outcomes in intervention than control** in 9 comparisons (2 studies).<br>Sub-group effects examined in 4 studies, with some better outcomes in intervention than control seen, but reporting of sub-groups unclear and incomplete.	LOW (downgraded two levels because of risk of bias and inconsistency[††])
**Comparison:** Two interventions		
3 studies[27 44 45]<br>630 participants, 87 comparisons (78 in Doyle, 7 in Landry)	**No difference between the two interventions** in 82 comparisons; **better outcomes in one intervention compared with another** in 4 comparisons (2 studies); **worse outcome in the more intensive intervention group** in 1 test (in 1 study).<br>Sub-group effects examined in 1 study, with some better outcomes in one intervention compared with another, but reporting of sub-groups unclear and incomplete.	LOW (downgraded two levels because of risk of bias and inconsistency[††])
Outcome: Overall Child Development		
**Comparison:** Intervention with usual care		
3 studies[‡ 22 38 58]<br>1414 participants, 8 comparisons	**No effect** in 7 comparisons; **better outcomes in intervention than control** in 1 comparison (1 study). No sub-groups examined.	MODERATE (downgraded one level because of inconsistency[††])
**Comparison:** Two interventions		
1 study[27]<br>151 participants, 4 comparisons	**No difference between the two interventions** in the 4 comparisons.	MODERATE (downgraded one level because of risk of bias)

[*] As the number of participants can vary within studies (e.g. where outcomes are measured at several different time points), the total number of participants noted here is calculated from the total numbers analysed at the last time point in each study; this is therefore a conservative estimate of the total number of participants for each outcome.

[†] Total number of comparisons performed for the specified outcome in the whole study population across all of the studies; studies in which 5 or more comparisons are conducted on the same outcome (either at one time point or across different time points) are referenced

[‡] Includes both comparisons in Wiggins (supportive health visiting with usual care, and community groups with usual care)

[§] Total participant numbers not reported in Tsiantis

[**] The comparison reported is the ratio or difference in estimates between intervention and control group, or between the two intervention groups, at the specified follow-up point unless otherwise noted ([**] indicates where the difference in change between intervention and control is used instead).

[††] Inconsistency noted where i) positive, negative and no effects are reported for an outcome, and/or ii) there is a different effect seen in more than 30% of comparisons across studies, and/or iii) different effects are reported within a study, and/or iv) most of the positive or negative effects are seen in sub-groups only and the reporting of sub-group effects is incomplete or inconsistent

GRADE Working Group grades of evidence
High quality: Further research is very unlikely to change our confidence in the summary of the effects.
Moderate quality: Further research is likely to have an important impact on our confidence in the summary of the effects.
Low quality: Further research is very likely to have an important impact on our confidence in the summary of the effects.
Very low quality: We are very uncertain about the summary of the effects.

For peer review only - http://bmjopen.bmj.com/site/about/guidelines.xhtml

Table 4: Effect direction plot, ordered by risk of bias and intensity of intervention (key given in footnote)

Study<br>Intervention intensity	Study design	Risk of bias	Adherence	Outcome measurement		Development outcomes					Additional detail on intervention effects or sub-group analyses
				Timing	When?	Motor	Lang	Cogn	SEWB	Overall	
**4a. Studies comparing one intervention with usual care**											
**Hiscock 2008**<br>Low	cRCT	Low	49% of parents attended all sessions	Post<br>Post<br>Post	Short<br>Medium<br>Long				○<br>○<br>○		
**Wiggins 2004**<br>Low (CGS)	iRCT	Low	19% of women attended a group	Post<br>Post	Immed<br>Short		 ○		 ○	○<br>○	
**Cheng 2007**<br>Moderate	iRCT	Low	Not reported	Post<br>Post	Short<br>Long				 ○		For the short-term analysis, only sub-group analyses (by attachment quality) presented; results inconsistent.
**Wiggins 2004**<br>Moderate (SHV)	iRCT	Low	Mean number of visits = 7 (of 12 planned)	Post<br>Post	Immed<br>Short		 ●		 ○	○<br>●	1 of 1 comparison showed improved language outcome and 1 of 1 comparison showed improved overall development in intervention group.
**Cupples 2011**<br>High	iRCT	Low	Mean number of contacts = 8.5 (of 22 planned)	Post	Immed	○		○	○		
**Griffith 2011**<br>High	iRCT	Low	60% attended 8 or more sessions (of 12 planned)	Post	Short				○	○	
**Miller 2015**<br>High	iRCT	Low	Adherence data currently being analysed	During	Long			○	○		
**High 2000**<br>Low	iRCT	High	Mean number of visits = 3.4 (of 5 planned)	Post	Short		○				3 of 6 comparisons showed improved language outcomes in intervention group.<br>Sub-group results: no differences seen in 13-17 month olds; 6 of 6 comparisons in 18-25 month olds showed improved language outcomes in intervention group; no test for interaction presented.
**Chang 2015**<br>Low	cRCT	Unclear	83% of mothers attended all visits	Post	Short	○	○	○		○	Improved cognitive outcome in intervention group on adjusting for potential confounders.
**Christakis 2007**<br>Low	iRCT	Unclear	Not reported	Post	Short		○		○		Sub-group results: 2 of 3 comparisons in low income group showed improved SEWB outcomes in intervention group; test for interaction not presented
**Feinberg 2008**<br>Moderate	iRCT	Unclear	80% attended at least 3 of 4 antenatal sessions<br>60% attended at least 3 of 4 postnatal sessions	Post<br>Post<br>Post	Short<br>Medium<br>Long				○<br>○<br>○		3 of 5 comparisons in short term and 1 of 2 comparisons in medium term showed improved SEWB outcomes in intervention group.<br>Results presented in text suggest there may be interaction effects with gender, but there is incomplete reporting of the sub-group analyses.
**Niccols 2008**<br>Moderate	iRCT	Unclear	58% attended 4 or more sessions (of 8 planned)	Post<br>Post	Immed<br>Short				○<br>○		

For peer review only

For peer review only - http://bmjopen.bmj.com/site/about/guidelines.xhtml

**Niccols 2009**<br>Moderate	iRCT	Unclear	Not reported	Post<br>Post	Immed<br>Short				○<br>•		2 of 4 comparisons immediately post-intervention term showed improved SEWB outcomes in intervention group.<br>3 of 4 comparisons showed improved SEWB outcomes in intervention group in short-term.
**Santelices 2011**<br>Moderate	iRCT	Unclear	Not reported	Post	Short				○		
**Tsiantis 2000**<br>Moderate	cRCT	Unclear	Not reported	Post<br>Post<br>Post	Short<br>Medium<br>Long	 	 <br>ø		○<br>○<br>○		2 of 2 comparisons showed poorer language outcomes in the intervention arm in the long-term.<br>1 of 2 comparisons showed poorer SEWB outcomes in the intervention arm in the medium-term.<br>1 of 8 comparisons showed improved SEWB outcomes in the intervention arm in the long-term.<br>Incomplete reporting of the sub-group analyses.
**Drotar 2008**<br>High	iRCT	Unclear	Not reported	During<br>During<br>During	Short<br>Medium<br>Long		 <br>○	 ○<br>○	○<br>○<br>○		1 of 12 comparisons showed improved SEWB outcomes in the intervention arm in the long-term.<br>Incomplete reporting of the sub-group analyses.
**Minkovitz 2003**<br>High	iRCT	Unclear	79% of parents received 4 or more services (of 16)	During	Long				○		
**Wagner 2001**<br>High	iRCT	Unclear	44% of families still receiving services at 2 yrs	During	Long	○	○	○	○		Results also stratified by income; no significant interactions reported.
**4b. Studies comparing two interventions**											
**Beeghly 1995**<br>2 low intensity	iRCT	Unclear	Not reported	Post	Short	○		○			Tested for interaction between intervention and parity, IUGR, "demographic" risk and maternal psychological risk; no significant interactions found.
**Lobo 2012**<br>2 moderate intensity	iRCT	Unclear	Excluded individuals who did not perform intervention on at least 60% of expected days	During<br>Post	Short<br>Short	○<br>•					4 of 8 comparisons showed improved motor outcomes in the intervention arm in the short-term whilst intervention was ongoing.<br>5 of 5 comparisons showed improved motor outcomes in the intervention arm post-intervention.
**Doyle 2011**<br>1 high & 1 moderate	iRCT	Unclear	High: Mean number of visits = 46<br>Moderate: Not reported	During<br>During<br>During	Short<br>Medium<br>Long	○<br>○<br>○	○<br>○<br>○	○<br>○<br>○	○<br>○<br>○	○<br>○<br>○	3 of 12 comparisons showed improved cognitive outcomes, and 1 of 62 comparisons showed improved SEWB outcomes in the intervention arm, in the long-term whilst intervention was ongoing.
**Johnston 2006**<br>2 high intensity	iRCT	Unclear	Not reported	During	Long		○		○		2 of 4 comparisons showed improved language outcomes, and 1 of 3 comparisons showed poorer SEWB outcomes, in the intervention arm in the long-term whilst intervention was ongoing.
**Landry 2006**<br>2 high intensity	iRCT	Unclear	91% of parents completed all 10 visits plus 2 assessment visits	Post	Short		•	•	○		2 of 2 comparisons showed improved language outcomes, and 1 of 1 comparison showed improved cognatic outcome in the intervention arm.<br>3 of 6 comparisons showed improved SEWB outcomes in the intervention arm.<br>Interaction with birthweight examined, but reporting of results incomplete.

For peer review only - http://bmjopen.bmj.com/site/about/guidelines.xhtml

Key: ● = outcome reported, statistically significant differences in favour of intervention found in 70% or more of comparisons within a study; Ø = outcome reported, statistically significant differences in favour of control found in 70% or more of comparisons; ○ = outcome reported, no statistically significant differences found, or found in <70% of comparisons; ◊ = outcome reported, inconsistent results (defined as in Table 3); [blank box] = outcome not reported

Size of the symbol indicates the total sample size included in analysis: ●Ø○◊ = >500; ●Ø○◊ = 100-500; ●ø○◊ = <100

Abbreviations: CGS = community groups; SHV = supportive health visiting; cRCT = cluster randomised controlled trial; iRCT = individually-randomised controlled trial; Immed = immediate; Lang = language; Cogn = cognitive; SEWB = social and emotional wellbeing

Outcome measurement i) Timing: During = whilst intervention is ongoing; Post = after intervention is completed

ii) When?: Immediate = <1 month; Short = 1-6 months; Medium = >6-12 months; Long = >12 months

For peer review only

Figure 1: PRISMA flow diagram

Footnote to Figure 1

Reason for exclusion at full text screening:
Ongoing study, n = 3
Quasi-experimental (control group but no randomisation), n = 10
Pre-post test comparison only, n = 5
Not a primary study (reviews, editorials, programme descriptions), n = 67
Not conducted in a high income country, n = 3
Intervention delivered in childcare settings, n = 14
Targeted programme (child factors), n = 37
Targeted programme (adult or family risk factors), n = 88
Mean age of children at intervention >24 months, n = 53
Mean age of children at outcome > 36 months, n = 5
No child development outcomes, n = 17

For peer review only

Figure 2: **Risk of bias graph: review authors' judgements about each risk of bias item presented as percentages across all included studies**

Figure 3: **Risk of bias summary: Review authors' judgements about each risk of bias item for each included study**

For peer review only - http://bmjopen.bmj.com/site/about/guidelines.xhtml

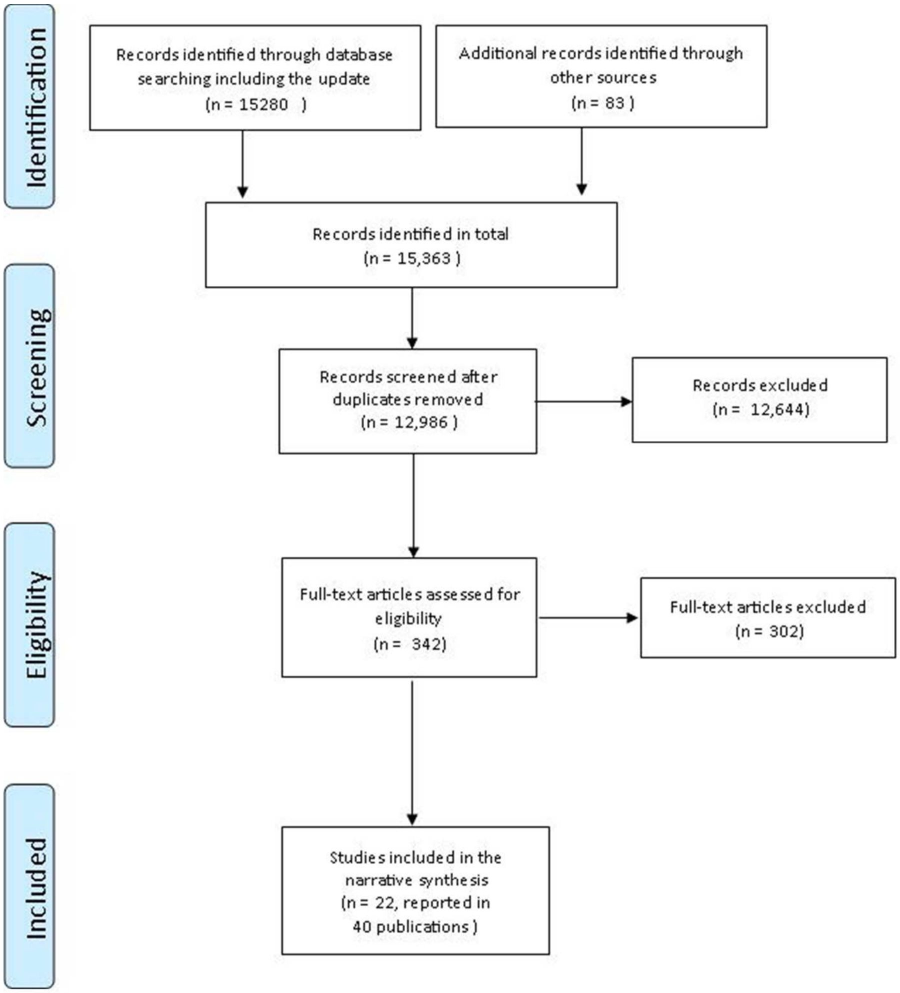

PRISMA flow diagram

173x224mm (96 x 96 DPI)

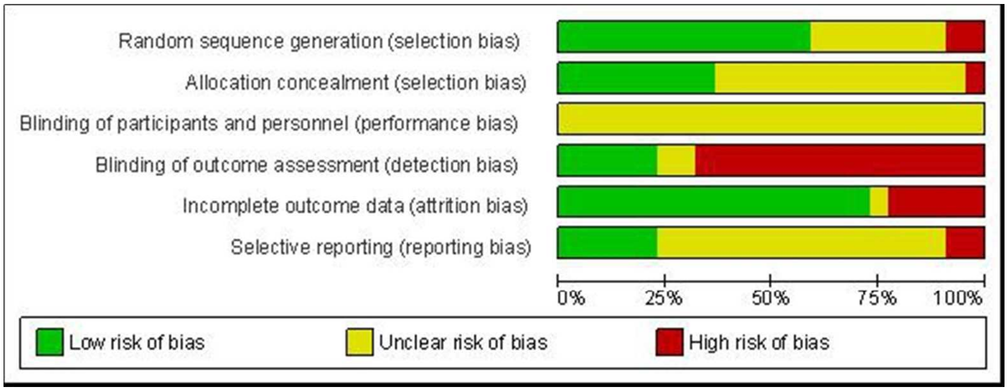

Risk of bias graph: review authors' judgements about each risk of bias item presented as percentages across all included studies

160x61mm (96 x 96 DPI)

For peer review only

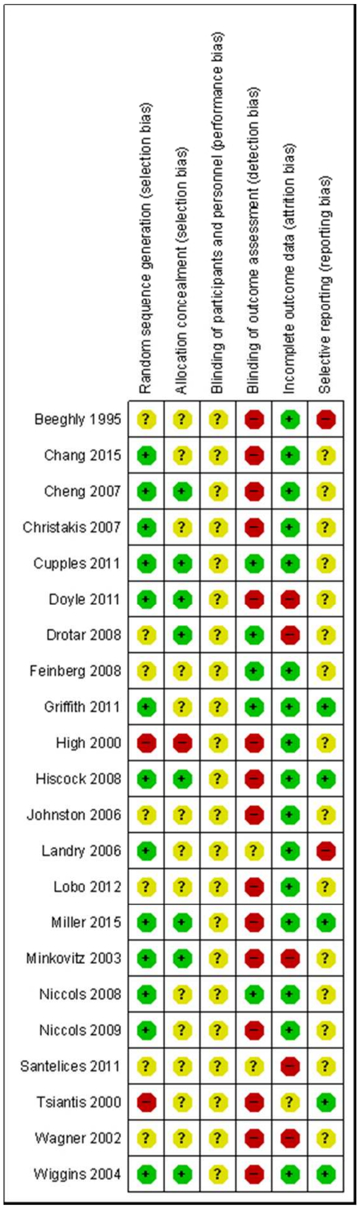

Risk of bias summary: Review authors' judgements about each risk of bias item for each included study

78x265mm (96 x 96 DPI)

Web Appendices

A. Medline search strategy

B. List of programme and organisation websites and journals for electronic journal of contents (eTOC) search

C. Full narrative summary of results

D. Effect direction plots, stratified by other characteristics

For peer review only

Web Appendix A. Medline search strategy

Database: Ovid MEDLINE(R) without Revisions
Search Strategy:
----
1 exp Health Personnel/
2 exp Nurses, Community Health/
3 health professional.tw.
4 exp Physician-Patient Relations/
5 (nurse* or physician* or doctor* or midwife* or midwive* or health visitor*).tw.
6 ((health* or medical or nurs* or hospital) adj (personnel or provider* or professional* or practitioner* or worker* or staff or specialist*)).tw.
7 (general practitioner* or gp or gps or clinician* or hospitalist* or psychologist* or social worker* or welfare worker*).tw.
8 ("Mothers aide" or "mothers help" or "maternity nurse" or "public health nurs*" or "specialist public health nurse" or "nursery nurse" or nanny or "early year educator*" or childcarer).tw.
9 ((paediatric or pediatric) adj clinic*).tw.
10 ((baby or child) adj clinic*).tw.
11 Primary Health Care/
12 exp House Calls/
13 home visit*.tw.
14 day care.mp.
15 clinic visit.tw.
16 surgery visit.tw.
17 clinician-patient interaction.tw.
18 early intervention*.tw.
19 ((parent* or mother*1 or father*1 or mum or dad or family or families) adj (group or support or training or education)).tw.
20 ((carer* or caregiver) adj (group or support or training or education)).tw.
21 ("early years staff" or "early years health professional*" or "early years professional*" or "early years educator*" or "early years practitioner*").tw.
22 (preschool educator or nursery staff).tw
23 parent educator*.tw.
24 (Family support or family support worker or support worker).tw.
25 (Community health worker or lay health worker).tw
26 Public health staff.tw.
27 Breakthrough to Literacy.mp
28 Family Foundations.mp.
29 Incredible Years.mp.
30 Parent Effectiveness Training.mp.
31 Primary Care Triple P.mp.
32 Triple P.mp.
33 Born to Learn.mp.
34 Mellow Parenting.mp.
35 "The Family Partnership Model".mp.
36 The Solihull Approach.mp.
37 "Parent child interaction therapy".mp.
38 "Nurturing Programme".mp.
39 "The Family Partnership Model".mp.
40 "Peers Early Education Partnership".mp.
41 "Webster Stratton Incredible Years".mp.

For peer review only

42 "Community Mothers Program".mp.
43 "Social Support and Family Health".mp.
44 "Limerick Lullaby".mp.
45 or/1-44 (ENHANCED PROFESSIONAL CONTACT TERMS)
46 exp Child Development/
47 exp language development/
48 Object Attachment/
49 Concept Formation/
50 Child Behavior Disorders/
51 ((child or infant) adj development).tw.
52 ((education* or cognitive or mental or language or physical or motor or social or emotional) adj3 (progress or development)).tw.
53 ((physical or emotional or social) adj (wellbeing or health)).tw.
54 (faltering growth or growth faltering or stunting).tw.
55 early child development.tw.
56 ((vocabulary or speech or language) adj development).tw.
57 Child behavio?r.tw.
58 Language/
59 Object Attachment/
60 social competence.tw.
61 social interaction.tw.
62 school readiness.tw.
63 or/46-62 (CHILD DEVELOPMENT TERMS)
64 45 and 63

For peer review only

Web Appendix B. List of programme and organisation websites and journals for electronic journal of contents (eTOC) search

Table B1. List of relevant programmes and organisations for which websites were searched

**Programme name**
1. Al's Pals
2. Breakthrough to Literacy
3. Bright Beginnings
4. Circle of Security
5. Community Mothers Program
6. Curiosity Corner
7. Developing Everyone's Learning and Thinking Abilities (DELTA)
8. Eager and Able to Learn
9. Family Check-Up
10. Family Foundations
11. Family Literacy programme
12. Family Nurturing Network: Family Connections
13. Family Partnership Model
14. Fun and Families
15. HIPPY
16. Holding Hands
17. I Can Problem Solve
18. Incredible Years
19. Infant Behavioral Assessment and Intervention Programme
20. Let's Begin with the Letter People
21. Let's Play in Tandem
22. LifeStart ("Growing Child")
23. Limerick Lullaby
24. Living with Children
25. Mellow Parenting
26. Minding the Baby
27. Noughts to Sixes (From Pram to Primary School), Family Caring Trust
28. Nurturing Programme
29. Parents as First Teachers or Parents as Teachers (Born to Learn)
30. Parent–child interaction therapy
31. Parent Effectiveness Training
32. Parenting Positively
33. Parents Plus Early Years
34. Peers Early Education Partnership
35. Preparing for Life<br>Child Development Initiative
36. Ready, Set, Leap!
37. Ready, Steady, Grow
38. Rock-a- Bye
39. Sheffield Raising Early Achievement in Literacy (REAL) project
40. Solihull Approach

For peer review only

41. Social Support and Family Health
42. Strengthening Families, Strengthening Communities
43. Supporting Parents on Kids Education (SPOKES)
**Organisation name**
1. Basic Skills Agency
2. Department of Education, UK
3. Early Childhood Australia
4. Education Endowment Fund
5. Early Intervention Foundation
6. European Commission (Education and Training)
7. European Early Childhood Education Research Association
8. Harvard Developing Child Programme
9. National Health Service (NHS)
10. National Institute for Health and Care Excellence (NICE)
11. National Institute of Health (USA)
12. Raising Children Network (Australia)
13. Sure Start/Flying Start
14. World Bank Early Child Development
15. World Health Organisation

For peer review only

Table B2. Journals included in electronic table of contents (eTOC) search

Archives of Diseases in Childhood

Child: Care, Health and Development

Child Development

Early Child Development and Care

Early Childhood Research Quarterly

Infant Mental Health Journal

Journal of Early Childhood Research

Pediatrics

For peer review only

Web Appendix C. Full narrative summary of results

Table C1: Motor Development Outcomes

Motor development outcomes		
**Studies comparing one intervention with usual care**		
**Study**	**Measure**	**Results**
Chang 2015 (Short term post intervention)	Griffith Mental Development Scale	**No difference in mean hand/eye coordination score** between intervention and control (mean 95.95, SD 10.25 compared with mean 94.62, SD 9.89); adjusted mean difference 0.73, 95% CI -0.83, 2.29; p value for comparison = 0.89)
Cupples 2011 (Immediately post intervention)	Bayley Scales of Infant Development (Psychomotor Development Index)	**No difference in mean scores** between intervention and control for PDI (MD [non-imputed] 1.64, 95% CI -0.94-4.21, p=0.21)
Wagner 2001 (Long term whilst programme ongoing)	Development Profile II	**No difference in mean physical development scores** between intervention and control in total sample (mean in intervention 2.9, mean in control 3.0, p>=0.05), or in samples stratified by income level (p>=0.05 for all tests)
**Motor development outcomes**		
**Studies comparing two interventions**		
**Study**	**Measure**	**Results**
Beeghly 1995 (Short term post-intervention)	Bayley Scales of Infant Development (Psychomotor Development Index)	**No difference in mean scores** for PDI in infant-centred (mean 109.77, SD 13.05) and mother-centred (mean 108.31, SD 12.43) groups at 3 months post-intervention (when children were 4 months, p>=0.05).
Doyle 2011 (Short term whilst intervention is ongoing)	Ages and Stages Questionnaire	**No differences** between high and low intensity programme using mean scores or cut-offs for 4 comparisons: gross motor scores, gross motor cut-off, fine motor scores, fine motor cut-offs (p values adjusted for multiple testing)
Doyle 2011 (Medium term whilst intervention is ongoing)	Ages and Stages Questionnaire	**No differences** between high and low intensity programme using mean scores or cut-offs for 4 comparisons: gross motor scores, gross motor cut-off, fine motor scores, fine motor cut-offs (p values adjusted for multiple testing)
Doyle 2011 (Long term whilst intervention is ongoing)	Ages and Stages Questionnaire (measured at 18, 24 and 36 months)	**No differences** between high and low intensity programme using mean scores or cut-offs for 4 comparisons at each time point: gross motor scores, gross motor cut-off, fine motor scores, fine motor cut-offs at any of the time points (p values adjusted for multiple testing)
Lobo 2012 (Short term whilst intervention ongoing)	Alberta Infant Motor Scale	**No difference for change in total mean scores** between in handling group compared with social experience group at 2-3 months (scores not given, p>=0.05).<br>**Greater change in mean score** for **prone** subscale in handling group compared with social experience group at 2-3 months of age (Cohen's correlation coefficient **r = -0.45, p<0.01**).<br>**No difference** for **supine** and **sitting** subscale at 2-3 months (scores not given, p>=0.05).<br>**Greater change in total mean score** in handling group compared with social experience group at 5 months of age (Cohen's correlation coefficient r = -0.46, p<0.01).<br>**Greater change in total mean score** for **prone** (r=-0.45, p<=0.01) and **sitting** (r=-0.34, p<=0.05) subscale in

For peer review only - http://bmjopen.bmj.com/site/about/guidelines.xhtml

		handling group at 5 months of age.<br>**No difference** for **supine** subscale at 5 months of age (scores not given, p>=0.05).
Lobo 2012<br>(Short term post-intevention)	Parent Milestone Report Form	Intervention group children reached for midlife objects before control children (no data provided, p<=0.05)<br>Intervention group children transferred objects from one hand to another **2.5 weeks earlier** (p<=0.05)<br>Intervention group children crept/crawled **5 weeks earlier** (p<=0.05)<br>Intervention group children walked supported **2.5 weeks earlier** (p<=0.05)<br>Intervention group children walked alone **6 weeks earlier** (p<=0.05)

For peer review only

Table C2: Language Development Outcomes

Language development outcomes		
**Studies comparing one intervention with usual care**		
**Study**	**Measure**	**Results**
Chang 2015 (Short term post intervention)	Griffith Mental Development Scale<br><br>MacArthur Bates Communicative Development Inventory (Short version, CDI)	**No difference in mean language scores** (Griffith scale) between intervention and control (mean 99.78, SD 14.01 compared with mean 99.90, SD 13.58); adjusted mean difference -0.54, 95% CI -2.81, 1.75; p value for comparison = 0.89)<br>**No difference in mean vocabulary scores (CDI)** between intervention and control (mean 38.00, SD 18.42 compared with mean 39.57, SD 20.49); adjusted mean difference -0.94, 95% CI -3.49, 1.61; p value for comparison = 0.89)
Christakis 2007 (Short term post intervention)	MacArthur Communicative Development Inventory	Whole sample: **No difference** between intervention and control (linear regression results: β for intervention variable raw score = 7.52, 95% CI -0.66-15.7; β for intervention variable percentile score = 8.40, 95% CI -3.00-19.90) at approx 4 months post intervention (children were 24 to 36 months).<br>Low and middle income sample: **Higher scores** in the intervention group (linear regression results: β for intervention variable raw score = 12.40, 95% CI 3.0-21.8, p=0.01; β for intervention variable percentile score = 14.94, 95% CI 1.37-27.60, p=0.03) at approx 4 months post intervention (children were 24 to 36 months).<br>High income sample: Results not presented in paper.
Drotar 2008 (Long term whilst intervention ongoing)	Systematic Analysis of Language Transcripts (SALT)	**No difference in mean scores** between intervention and control when children were 36 months (MD -3.91, 95% CI -14.32-6.77, p=0.48).
High 2000 (Short term post-intervention)	Modified MacArthur Communicative Development Inventory (short form)	**Higher mean scores** in intervention group for receptive vocabulary in total sample (p values between 0.008 and 0.003 in 3 tests), but no difference on expressive vocabulary (3 tests)<br>**No difference** in mean scores in receptive and expressive vocabulary between intervention and control groups in children aged 13-17 months at assessment (6 sub-group tests).<br>**Higher mean scores** in intervention group for receptive and expressive vocabulary in children aged 18-25 months at assessment (**p values between 0.01 and 0.003 in 6 sub-group tests**)
Tsiantis 2000 (Immediately post-intervention)	Bzoch-league Receptive Expressive Emergent Language Scale (REEL)	**Better performance in observed language** in comparison group children (no scores presented, but **p<0.0001**)<br>Results analysed by country, but not presented in paper.
Wagner 2002 (Long term whilst intervention is ongoing)	Development Profile II (DPII)	**No difference in mean scores** between intervention and control in communication development scores in whole sample (mean in intervention 4.9, mean in control 4.5, p>=0.05), or when stratified by income group.
Wiggins 2004 (Short term post-intervention)	Question on whether the mother has any worries about their child's speech	**Fewer mothers in the SHV group** expressed a worry about their child's speech than in the control group (risk ratio 0.46, 95% CI 0.23, 0.93)<br>**No difference** in the number of number of mothers expressing worries about speech between CGS and control (risk ratio 1.22, 95% CI 0.78, 1.92)

For peer review only

Language development outcomes		
**Studies comparing two interventions**		
**Study**	**Measure**	**Results**
Doyle 2011 (Short term whilst intervention is ongoing)	Ages and Stages Questionnaire	**No differences** between high and low intensity programme using mean scores or cut-offs for 2 comparisons: communication scores, communication cut-off (p values adjusted for multiple testing)
Doyle 2011 (Medium term whilst intervention is ongoing)	Ages and Stages Questionnaire<br><br>MacArthur-Bates Communicative Development Inventory	**No differences** between high and low intensity programme using mean scores or cut-offs for 2 comparisons: communication scores, communication cut-off (p values adjusted for multiple testing).<br>**No differences** between high and low intensity programme using mean scores for 4 sub-scores: first signs of understanding, words understood, words produced, first communicative gestures (p values adjusted for multiple testing)
Doyle 2011 (Long term whilst intervention is ongoing)	Ages and Stages Questionnaire (measured at 18, 24 and 36 months)<br>MacArthur-Bates Communicative Development Inventory (measured at 18 and 24 months)	**No differences** between high and low intensity programme using mean scores or cut-offs for 2 comparisons at any of the time points: communication scores, communication cut-off (p values adjusted for multiple testing).<br>**No differences** between high and low intensity programme using mean scores for 5 sub-scores at 18 months: first signs of understanding, can combine words, first communicative gestures, words produced, words understood, and 2 sub-scores at 24 months: can combine words, words produced (p values adjusted for multiple testing)
Johnston 2006 (Long term post-intervention)	Sub-scales of MacArthur Communicative Development Inventory	**Higher mean vocabulary score** in HS + PP group than HS only group at 24 months (aMD 4.08; 95% CI 0.69, 7.47; p<0.05).<br>**Higher percentage** combining >=2 words in HS + PP group than HS only group (rate ratio 1.05; 95% CI 1.02, 1.08; p<0.05).<br>**No difference** in length of child's longest phrase or variety of word endings between HS+PP and HS only group.
Landry 2006 (Short term post-intervention)	Coding of videotaped interactions between child and mother, and child with examiner	**Higher word usage scores** observed in the PALS group than the DAS group in interactions with mother (p=0.02) and examiner (p=0.02) at 3 months post-intervention when child was 13 months.

For peer review only

Table C3: Cognitive Development Outcomes

Cognitive development outcomes		
**Studies comparing one intervention with usual care**		
**Study**	**Measure**	**Results**
Chang 2015 (Short term post intervention)	Griffith Mental Development Scale	No effect in intervention group in unadjusted results (mean 92.69, SD 11.65 compared with mean 89.52, SD 10.56); unadjusted mean difference 3.12, 95% CI 0.86, 5.39; p value for comparison = 0.07); **higher mean cognitive scores (Griffith scale)** for adjusted mean difference (aMD 3.09, 95% CI 1.31, 4.87, p value = 0.007)
Cupples 2011 (Immedlately post-intervention)	Bayley Scales of Infant Development (Mental Development Index, MDI)	**No difference in mean scores** between intervention and control when children were 12 months old (MD [non-imputed] -0.81, 95% CI -2.81-1.16, p=0.42)
Drotar 2008 (Short-medium term whilst intervention ongoing)	Bayley Scales of Infant Development (Mental Development Index, MDI)<br>Assessor rating of mastery motivation (persistence, pleasure and competence with a play task)	**No difference in mean scores** between intervention and control when children were 18 months (MD 0.68, 95% CI -1.15-2.76, p=0.42).<br>**No difference in mean scores** between intervention and control for any of the 3 tasks when children were 18 months.
Drotar 2008 (Long term whilst intervention ongoing)	Bayley Scales of Infant Development (Mental Development Index, MDI)<br><br>Assessor rating of mastery motivation (persistence, pleasure and competence with a play task)<br><br><br><br><br>Kaufman Assessment Battery<br><br><br>Bracken Basic Concept Scale<br><br>Test of Early Reading Ability-2 (TERA-2)	**No difference in mean scores** between intervention and control for MDI (MD -0.78, 95% CI -3.63-2.32, p=0.67) when children were 24 months. **Higher mean score** in intervention than control in low SES sub-group (p for interaction < 0.003, mean for intervention 89.67 (SD16.20) & mean for control 82.16 (SD 17.24, p < 0.01).<br>**No difference in mean scores** between intervention and control for any of the 3 tasks when children were 24 months. **Higher mean score** for task competence in intervention group for low SES sub-group (p for interaction not shown, mean for intervention 584.49 (SD 37.97) & mean for control 556.21 (SD 57.18), p < 0.02.<br>**No difference** between intervention and control in 2 of 3 tests of mastery motivation (persistence and pleasure) when children were 36 months; **higher mean scores** in intervention group for task competence (MD 6.24, 95% CI 0.16-14.76, p=0.05).<br>**No difference in mean scores** between intervention and control for sequential processing, simultaneous processing or mental processing when children were 36 months (e.g. for mental processing MD 0.23, 95% CI -3.33-2.91, p=0.89).<br>**No difference in mean scores** between intervention and control when children were 36 months (MD 1.24, 95% CI -2.24-4.47, p=0.51).<br>**No difference in mean scores** between intervention and control when children were 36 months (MD 0.54, 95% CI -1.70-2.47, p=0.72).
Miller 2015 (Long term whilst intervention ongoing)	British Ability Scale (BASII)	**No difference in mean scores** between intervention and control on cognitive development (mean in intervention group -0.046 (SD 1.007) and mean in control group 0.018 (SD 0.994), Hedges g effect size -0.634, 95% CI -0.277, 0.149, p=0.557)
Wagner 2001 (Long term whilst intervention ongoing)	Development Profile II (DPII)	**No difference in mean scores** between intervention and control in cognitive development scores (mean in intervention 2.2, mean in control 2.4, p>=0.05), or in samples stratified by income.

For peer review only

Cognitive development outcomes		
**Studies comparing two interventions**		
**Study**	**Measure**	**Results**
Beeghly 1995 (Short term post intervention)	Bayley Scales of Infant Development (Mental Development Index, MDI)	**No difference in mean scores** in infant-centred (mean 109.38, SD 12.88) or mother-centred (mean 107.83, SD 10.69) groups at 3 months post-intervention (when children were 4 months, p>=0.05).
Doyle 2011 (Short term whilst intervention is ongoing)	Ages and Stages Questionnaire	**No differences** between high and low intensity programme using mean scores or cut-offs for 2 comparisons: problem solving score, problem solving cut-off (p values adjusted for multiple testing)
Doyle 2011 (Medium term whilst intervention is ongoing)	Ages and Stages Questionnaire<br><br>Development Profile 3	**No differences** between high and low intensity programme using mean scores or cut-offs (2 comparisons): problem solving score, problem solving cut-off (p values adjusted for multiple testing).<br>**No difference** in mean scores or for the proportion above the average cut-off (2 comparisons, p values not adjusted for multiple testing).
Doyle 2011 (Long term whilst intervention is ongoing)	Ages and Stages Questionnaire (measured at 18, 24 and 36 months)<br>Development Profile 3 (measured at 18, 24 and 36 months)	**No differences** between high and low intensity programme using mean scores or cut-offs for 2 comparisons at any of the time points: problem solving score, problem solving cut-off (p values adjusted for multiple testing).<br>At 18 months, **no difference** in mean scores or for the proportion above the average cut-off (p values not adjusted for multiple testing).<br>At 24 months, **no difference for the proportion above the average cut-off** (p values **not** adjusted for multiple testing); **higher mean standardised score** in high intensity group (mean 116.51 [SD 13.30] vs mean 112.57 [S 16.12], p<0.05, **not** adjusted for multiple testing).<br>At 36 months, **higher mean standardised score** in high intensity group (mean 114.66 [SD 14.39] vs mean 109.82 [SD 14.02], p<0.05, **not** adjusted for multiple testing) and **higher proportion above average cut-off** (0.53 [SD0.50] vs 0.36 [SD 0.48], p<0.05, **not** adjusted for multiple testing)
Landry 2006 (Short term post-intervention)	Coding of videotaped interactions between child and mother, and child with examiner	**Higher scores** for independent goal-directed play in the PALS group than in the DAS group (p=0.04) at 3 months post-intervention when child was 13 months.

For peer review only

Table C4: Social and Emotional Wellbeing Outcomes

Social and emotional wellbeing outcomes		
**Studies comparing one intervention with usual care**		
**Study**	**Measure**	**Results**
Cheng 2007 (Short term post-intervention)	Parent-Infant Relationship Global Assessment Scale	Of 37 mother-infant pairs with a "disturbed" relationship at baseline, a **higher percentage** of intervention group was classified as having an "adapted" relationship (63% [12 pairs] vs 28% [5 pairs], p=0.039) at 1 month post-intervention (when children were 10 months).<br>Of 48 mother-infant pairs with an "adapted" relationship at baseline, **no difference in percentage** of intervention vs control classified as "disturbed" (26% [7 pairs] vs 29% [8 pairs], p=0.821) at 1 month post-intervention (when children were 10 months).
Cheng 2007 (Long term post-intervention)	Child Behaviour Checklist	**No difference** between intervention and control in the odds of being in the upper quartile of the CBCL scores (total score aOR 0.40, 95% CI 0.12-1.43; internalizing score aOR0.60, 95% CI 0.23-1.68; externalizing score aOR 0.50, 95% CI 0.19-1.54) at 15 months post-intervention (when children were 24 months).
Christakis 2007 (Short term post-intervention)	Hyperactivity sub-domain of the Child Behavior Checklist	**No difference** in attention problems (score in 90th percentile or higher) between intervention and control in the whole sample (aOR 0.49, 95% CI 0.13-1.81) or low and middle income sample (aOR 0.48, 95% CI 0.12, 1.83) at approx 4 months post intervention (children were 24 to 36 months).
Cupples 2011 (Immediately post-intervention)	Bayley Scales of Infant Development (BSID-II)	**No difference in the mean scores** between intervention and comparison group (MD [non-imputed] -0.88, 95% CI -4.59-2.81, p=0.64)<br>**No difference in the mean scores** between intervention and control for the 3 sub-scores of the BSID-II behavioural scale.
Drotar 2008 (Short-medium term whilst intervention ongoing)	Bayley Behaviour Rating Scale	**No difference in mean scores** between intervention and control when children were 12 months (MD -0.33, 95% CI -1.95-1.64, p=0.87).
Drotar 2008 (Medium-long term whilst intervention ongoing)	Q-Sort measure of Security of Attachment	**No difference** between intervention and control when children were 18 months (no numerical data presented).
Drotar 2008 (Long term whilst intervention ongoing)	Bayley Behaviour Rating Scale<br><br>Child Behaviour Rating Scale<br>Social Skills Rating System	**No difference in mean scores** between intervention and control when children were 24 months (MD 0.43, 95% CI -1.72-3.15, p=0.56) or 36 months (MD -1.64, 95% CI -3.99-0.83, p=0.20).<br>**No difference** between intervention and control when children were 24 months (no numerical data presented).<br>**No difference in mean scores** between intervention and control when children were 36 months for parent. reported social skills (MD 3.33, 95% CI -0.45-5.90, p=0.09) or teacher reported social skills (MD 2.07, 95% CI -3.19-7.15, p=0.45).
Feinberg 2008 (Immediately post-intervention)	Infant Behaviour Questionnaire	**No difference** in parent-child dysfunctional interaction (beta calculated using linear regression) in intervention versus control group according to mother's report (beta -0.137, Cohen's d 0.34, p<0.10), but **intervention fathers had lower dysfunctional interaction scores** (beta = -0.291, Cohen's d = 0.70, p<0.05).<br>**No difference** in infant soothability score (beta calculated using linear regression) in intervention versus control

For peer review only

		group according to mother's report (beta 0.021, Cohen's d not calculated, p>=0.05), but **intervention children were easier to soothe** (had a higher score than control) according to father's reports (beta = 0.312, Cohen's d = 0.35, p<0.05).<br>Score for duration of attention **higher in intervention versus control infants** according to mother's report (F=4.33, Cohen's d = 0.34, p<0.05). Fathers reports of this variable not given in paper.
Feinberg 2008<br>(Short term post-intervention)	Observer coding of videotaped behaviours (self-soothing and sustained attention at play)	**More self-soothing behaviours** observed in intervention versus control group when infants were 12 months of age (6 months post-intervention, beta = 0.30, Cohen's d = 0.46, p<0.05).<br>**No difference** in sustained attention noted by coders between intervention and control children (beta = 0.05, Cohen's d = 0.08, p>=0.05).
Feinberg 2009<br>(Short term post-intervention)	Child Behaviour Checklist	Mean scores and SDs for each of the 7 comparisons presented but no accompanying P values; t tests conducted in Stata (assuming 81% follow-up in each group) indicate **no significant differences**.<br>Written results suggest that the model based estimates of intervention effects were significant but only when an interaction with child gender was included in the model - the presentation of these results is unclear and there appears to be an inconsistency between the results presented in the table and those in the text.
Griffith 2011<br>(Short term post-intervention)	Dyadic Parent-Child Interaction coding system	**No difference in mean scores** between intervention and control for child deviance behaviours in the parent-child interaction observations (negative physical behaviours, destructive behaviours, small talk; mean in intervention 3.18 (SD 3.40) and control 2.97 (SD 3.43), no p value for this comparison) at 3 months or less post-intervention.
Hiscock 2008<br>(Short term post-intervention)	Child Behaviour Checklist	**No difference in mean scores** between intervention and control for externalising (aMD 0.16, 95% CI -1.01-1.33, p=0.79) or internalising (aMD 0.49, 95% CI -0.20-1.18, p=0.16) behaviours 3 months post-intervention (when children were 18 months).
Hiscock 2008<br>(Medium term post-intervention)	Child Behaviour Checklist	**No difference in mean scores** between intervention and control for externalising (aMD -0.79, 95% CI -2.27-0.69, p=0.30) or internalising (aMD 0.19, 95% CI -0.77-1.15, p=0.70) behaviours 9 months post-intervention (when children were 24 months).
Hiscock 2008<br>(Long term post-intervention)	Child Behaviour Checklist	**No difference in mean scores** between intervention and control for externalising (aMD -0.8, 95% CI -2.2-0.6, p=0.26) or internalising (aMD -0.6, 95% CI -1.5-0.3, p=0.19) behaviours 21 months post-intervention (when children were 36 months).
Miller 2015<br>(Long term whilst intervention is ongoing)	Bayley Social-Emotional and Adaptive Behaviour Questionnaire	**No difference in mean scores** between intervention and control on socio-emotional development (mean in intervention group 0.087 (SD 0.987) and mean in control group -0.108 (SD 1.016, Hedges g effect size 0.195 (95% CI -0.046, 0.437, p=0.114)
Minkovitz 2003<br>(Long term whilst intervention is ongoing)	Subscales of Child Behaviour Checklist	**No difference in mean scores** for 3 sub-scores (aggressive behaviour, anxious or depressed, sleep problems) between intervention and control group. For example, for aggressive behaviour, the adjusted mean difference between intervention and control was 0.23 (95% CI -0.29, 0.79, p=0.16, higher scores indicate higher reporting of aggressive behaviour)
Niccols 2008<br>(Immediately post-intervention)	Attachment Q-set (completed by observers)	**No difference in the change in scores between pre-test and post-test** between intervention and comparison group in the intention-to-treat analysis (no numbers presented, text states "no significant differences")
Niccols 2008<br>(Short term post-intervention)	Attachment Q-set (completed by observers)	**No difference in the change in scores between pre-test and post-test** between intervention and comparison group in the intention-to-treat analysis 6 months post-intervention (no numbers presented, text states "no significant differences")

For peer review only

Niccols 2009 (Immediately post-intervention)	Eyberg Child Behavior Inventory<br><br>Observed parent-child interaction	**No difference in mean scores** between intervention and control for child behaviour problems (mean 48.87 (SD 6.22) vs mean score 50.68 (SD 7.23), p>=0.05)<br>**Higher mean number of observed** positive child behaviours in intervention group (mean 21.10 (SD 8.59) vs mean score 16.27 (SD 9.25), p<0.05), and **higher ratio** of child compliance to maternal requests in intervention group (mean 0.62 (SD 0.19) vs mean score 0.53 (SD 0.19), p<0.05).<br>**No difference in mean number** of observed negative behaviours in intervention versus control (mean 17.81 (SD 8.84) vs mean score 22.29 (SD 8.85), p>=0.05).
Niccols 2009 (Short term post-intervention)	Eyberg Child Behavior Inventory<br><br>Observed parent-child interaction	**Lower mean score** for child behaviour problems in intervention versus control (mean 48.87 (SD 6.22) vs mean score 50.68 (SD 7.23), p<0.017).<br>**Higher mean number** of observed positive child behaviours in intervention group than control (mean 22.61 (SD 8.97) vs mean score 16.69 (SD 8.40), p<0.05), and **higher ratio of** observed compliance to maternal requests in intervention group (mean 0.61 (SD 0.19) vs mean score 0.53 (SD 0.19), p<0.05).<br>**No difference** in mean number of observed negative behaviours between intervention and control (mean 18.86 (SD 7.53) vs mean score 21.29 (SD 7.78), p>=0.05)
Santelices 2010 (Immediately post-intervention)	Observed Strange Situation Procedure	**No difference** between intervention and control in infant attachment security (% classed as "secure" in intervention 72.1%, % classed as "secure" in control 55.2%, p=0.139)
Tsiantis 2000 (Short term whilst intervention is ongoing)	Bayley Scale of Psychosocial Development<br>Observed mother-child interaction	**No difference in mean scores** between intervention and control (mean 7.03 compared with mean 7.04, no numbers in analysis or statistics presented).<br>**Mother-child interaction rated as better** in intervention group in one of the four countries (no numbers in analysis, or scores presented, p for sub-group effect= 0.02).
Tsiantis 2000 (Medium term whilst intervention is ongoing)	Bayley Scale of Psychosocial Development<br>Observed mother-child interaction	**No difference in mean scores** between intervention and control (mean 6.82 compared with mean 7.06, no numbers in analysis or statistics presented)<br>**Mother-child interaction rated as better in comparison group** when all countries' data combined (no numbers in analysis presented, scores presented but not clear what they represent, p = 0.02)
Tsiantis 2000 (Long term whilst intervention is ongoing)	Bates Infant Characteristics Questionnaire	**No difference in total mean scores** in full sample between intervention and control (no numbers in analysis, scores or statistics presented for mean scores); 2 of 7 individual characteristic scores higher in intervention than control in full sample ("sober" p = 0.02, "factor 7" p <0.06).
Wagner 2001 (Long term whilst intervention is ongoing)	Nursing Child Assessment Satellite Training teaching scale (NCAST) used to observe mother-child interactions<br>Measure = Development Profile II (DPII)<br><br>Adaptive Social Behaviour Inventory	**No difference in mean scores** for observed mother-child interaction between intervention and control in total sample (2 comparisons), or when results stratified by income group (p>=0.05)<br><br>**No difference in mean self help or social development scores** between intervention and control in total sample, or when results stratified by income group (p>=0.05)<br>**No difference in mean scores** between intervention and control in total sample, or when results stratified by income group (p>=0.05)
Wiggins 2004 (Short term post-intervention)	Question on whether the mother has any worries about their child's behaviour	**No difference** in the number of number of mothers expressing worries about behaviour between supportive health visiting and control groups (risk ratio 0.74, 95% CI 0.37, 1.47)<br>**No difference** in the number of number of mothers expressing worries about behaviour between community group support and control groups (risk ratio 0.94, 95% CI 0.51, 1.73)

For peer review only

Social and emotional wellbeing outcomes		
**Studies comparing two interventions**		
**Study**	**Measure**	**Results**
Doyle 2011 (Short term whilst intervention is ongoing)	Ages and Stages Questionnaire<br><br>Child Characteristic Questionnaire	**No differences** between high and low intensity programme using mean scores or cut-offs for 4 comparisons: personal social score, personal social cut-off, social-emotional score, social-emotional cut-off (p values adjusted for multiple testing).<br>**No difference** between high and low intensity programme for score based on a 6 point scale of difficult temperament (p value adjusted for multiple testing).
Doyle 2011 (Medium term whilst intervention is ongoing)	Ages and Stages Questionnaire<br><br>Brief Child Toddler Social and Emotional Assessment<br>Temperament and Atypical Behaviour Score<br><br>Child Characteristic Questionnaire	**No differences** between high and low intensity programme using mean scores or cut-offs for 4 comparisons: personal social score, personal social cut-off, social-emotional score, social-emotional cut-off (p values adjusted for multiple testing)<br>**No differences** between high and low intensity programme using mean scores or cut-offs for 4 comparisons: competence score, competence cut-off, problem score, problem cut-off (p values adjusted for multiple testing)<br>**No differences** between high and low intensity programme using mean scores or binary variable (p values **not** adjusted for multiple testing)<br>**No difference** between high and low intensity programme for score based on a 6 point scale of difficult temperament (p value **not** adjusted for multiple testing)
Doyle 2011 (Long term whilst intervention is ongoing)	Ages and Stages Questionnaire (measured at 18, 24 and 36 months)<br><br>Brief Child Toddler Social and Emotional Assessment (measured at 18, 24 and 36 months)<br>Brief Child Toddler Social and Emotional Assessment subdomains (measured at 24 and 36 months)<br>Child Behaviour Checklist (CBCL, measured at 24 and 36 months)<br><br>Child Behaviour Checklist subdomains (CBCL, measured at 24 and 36 months)	**No differences** between high and low intensity programme using mean scores or cut-offs for 4 comparisons: personal social score, personal social cut-off, social-emotional score, social-emotional cut-off (p values adjusted for multiple testing)<br>**No differences** between high and low intensity programme using mean scores or cut-offs for 4 comparisons at 18 and 24 months: competence score, competence cut-off, problem score, problem cut-off, and for 2 comparisons at 36 months: competence score, problem score (p values adjusted for multiple testing)<br>**No differences in mean scores** between high and low intensity programme for 5 comparisons at 24 or 36 months: dysregulation, internal problems, red flag, external problems, autism (p value adjusted for multiple testing).<br>At 24 months, **no difference** between high and low intensity for 5 comparisons (CBCL total score; internalising problems score and cut-off, externalising problems score and cut-off); **higher proportion in the low intensity group above cut-off for total CBCL score** (0.00 [SD 0.00] vs 0.07 [0.26], p<0.01 adjusted for multiple testing).<br>At 36 months, **no difference** between high and low intensity for 6 comparisons (CBCL total score and cut-off; internalising problems score and cut-off, externalising problems score and cut-off)<br>**No difference** in scores between high and low intensity programmes for 8 comparisons at 24 or 36 months: sleep problems, other problems, emotionally reactive, attention problems, anxious/depressed, somatic complaints, aggressive, withdrawn (p values adjusted for multiple testing)
Johnston 2006 (Long term whilst intervention is ongoing)	Subscales of Child Behaviour Checklist	**Higher mean score** in HS+PP group than HS only group for anxiety or depression at 36 months (aMD 0.40; 95% CI 0.07, 0.73; p<0.05).<br>**No difference** in aggressive behaviour between HS+PP group and HS only group.

For peer review only - http://bmjopen.bmj.com/site/about/guidelines.xhtml

Landry 2006<br>(Short term post-intervention)	Coding of videotaped interactions between child and mother, and child with examiner	**No difference** in mean scores for cooperation with mothers (p=0.08) or a novel adults (results not presented).<br>Infants in PALS demonstrated "**significantly greater increase in their use of words**" when interacting with mothers (p=0.02) and a novel adult (p=0.02).<br>**No effect** on affect in interactions with mothers overall (results not presented); **lower negative affect** seen in infants in the PALS group when interacting with a novel adult (results not presented).<br>Also present some results for the interaction with birthweight but presentation of results incomplete (text suggests more positive effects of the PALS intervention in very low birthweight group)

For peer review only

Table C5: Overall Development Outcomes

Overall development outcomes		
**Studies comparing one intervention with usual care**		
**Study**	**Measure**	**Results**
Chang 2015 (Short term post intervention)	Griffith Mental Development Scale	**No difference in mean developmental quotient (Griffith)** between intervention and control (mean 96.14, SD 9.40 compared with mean 94.68, SD 8.25); adjusted mean difference 1.10, 95% CI -0.45, 2.65; p value for comparison = 0.72)
Griffith 2011 (Short term post-intervention)	Schedule of Growing Skills II (SGS II)	**No difference in mean scores** between intervention (mean 99.20, SD 18.27) and control (mean 102.38, SD 17.79) at around 3 months post-intervention (p=0.51)
Wiggins 2004 (Immediately post-intervention)	Question on mother's perception of whether her child's development was normal	**No difference** between “supportive home visiting” and control (risk ratio 0.88, 95% CI 0.39-1.99) or “community groups” and control (risk ratio 0.57, 95% CI 0.22-1.52) in mother's perception of whether her child's development was normal
Wiggins 2004 (Short term post-intervention)	Questions on mother’s worries about different aspects of development	Comparison on general development not possible between SHV and control as no mothers expressed concerns in the SHV group.<br>**No difference** in the number of number of mothers expressing worries about general development between CGS and control (risk ratio 0.75, 95% CI 0.15, 1.92)<br>Mothers in the SHV group had **fewer mean number of worries** about their child's development than in the control group (MD -0.23, 95% CI -0.42 to -0.01)<br>**No difference in the mean number of worries** about their child's development between CGS and control (MD 0.13, 95% CI -0.10 to 0.36)
**Overall development outcomes**		
**Studies comparing one intervention with usual care**		
**Study**	**Measure**	**Results**
Doyle 2011 (Short term whilst intervention is ongoing)	Ages and Stages Questionnaire	**No difference in overall mean score** between high and low intensity programme (p value **not** adjusted for multiple testing) at 6 months
Doyle 2011 (Medium term whilst intervention is ongoing)	Ages and Stages Questionnaire	**No difference in overall mean score** between high and low intensity programme (p value **not** adjusted for multiple testing) at 12 months
Doyle 2011 (Long term whilst intervention is ongoing)	Ages and Stages Questionnaire (measured at 18, 24 and 36 months)	**No difference in overall mean score** between high and low intensity programme (p value **not** adjusted for multiple testing) at any of the time points

For peer review only - http://bmjopen.bmj.com/site/about/guidelines.xhtml

D. Effect direction plot, stratified by other characteristics

D1: Results stratified by age of children at intervention delivery

Study<br>Intervention intensity	Study design	Risk of bias	Adherence	Outcome measurement		Development outcomes					Additional detail on intervention effects or sub-group analyses
				Timing	When?	Motor	Lang	Cogn	SEWB	Overall	
**Studies comparing one intervention with usual care**											
**Intervention during antenatal and/or first year postpartum only**											
**Beeghly 1995**<br>2 low intensity	iRCT	Unclear	Not reported	Post	Short	○		○			Tested for interaction between intervention and parity, IUGR, "demographic" risk and maternal psychological risk; no significant interactions found.
**Cheng 2007**<br>Moderate	iRCT	Low	Not reported	Post<br>Post	Short<br>Long				 ○		For the short-term analysis, only sub-group analyses (by attachment quality) presented; results inconsistent.
**Cupples 2011**<br>High	iRCT	Low	Mean number of contacts = 8.5 (of 22 planned)	Post	Immed	○		○	○		
**Feinberg 2008**<br>Moderate	iRCT	Unclear	80% attended at least 3 of 4 antenatal sessions<br>60% attended at least 3 of 4 postnatal sessions	Post<br>Post<br>Post	Short<br>Medium<br>Long				○<br>○<br>○		3 of 5 comparisons in short term and 1 of 2 comparisons in medium term showed improved SEWB outcomes in intervention group.<br>Results presented in text suggest there may be interaction effects with gender, but there is incomplete reporting of the sub-group analyses.
**Lobo 2012**<br>2 moderate intensity	iRCT	Unclear	Excluded individuals who did not perform intervention on at least 60% of expected days	During<br>Post	Short<br>Short	○<br>●					4 of 8 comparisons showed improved motor outcomes in the intervention arm in the short-term whilst intervention was ongoing.<br>5 of 5 comparisons showed improved motor outcomes in the intervention arm post-intervention.
**Santelices 2011**<br>Moderate	iRCT	Unclear	Not reported	Post	Short				○		
**Wiggins 2004**<br>Moderate (SHV)	iRCT	Low	Mean number of visits = 7 (of 12 planned)	Post<br>Post	Immed<br>Short		 ●		 ○	○<br>●	1 of 1 comparison showed improved language outcome and 1 of 1 comparison showed improved overall development in intervention group.
**Wiggins 2004**<br>Low (CGS)	iRCT	Low	19% of women attended a group	Post<br>Post	Immed<br>Short		 ○		 ○	○<br>○	
**Intervention during first and second years postpartum**											
**Chang 2015**<br>Low	cRCT	Unclear	83% of mothers attended all visits	Post	Short	○	○	○		○	Improved cognitive outcome in intervention group on adjusting for potential confounders.
**Christakis 2007**<br>Low	iRCT	Unclear	Not reported	Post	Short		○		○		Sub-group results: 2 of 3 comparisons in low income group showed improved SEWB outcomes in intervention group; test for interaction not presented
**Doyle 2011**<br>1 high & 1 moderate	iRCT	Unclear	High: Mean number of visits = 46<br>Moderate: Not reported	During<br>During<br>During	Short<br>Medium<br>Long	○<br>○<br>○	○<br>○<br>○	○<br>○<br>○	○<br>○<br>○	○<br>○<br>○	3 of 12 comparisons showed improved cognitive outcomes, and 1 of 62 comparisons showed improved SEWB outcomes in the intervention arm, in the long-term whilst intervention was ongoing.

**Drotar 2008**<br>High	iRCT	Unclear	Not reported	During<br>During<br>During	Short<br>Medium<br>Long		 <br>○	 ○<br>○	○<br>○<br>○		1 of 12 comparisons showed improved SEWB outcomes in the intervention arm in the long-term. Incomplete reporting of the sub-group analyses.
**Griffith 2011**<br>High	iRCT	Low	60% attended 8 or more sessions (of 12 planned)	Post	Short				○	○	
**High 2000**<br>Low	iRCT	High	Mean number of visits = 3.4 (of 5 planned)	Post	Short		○				3 of 6 comparisons showed improved language outcomes in intervention group.<br>Sub-group results: no differences seen in 13-17 month olds; 6 of 6 comparisons in 18-25 month olds showed improved language outcomes in intervention group; no test for interaction presented.
**Hiscock 2008**<br>Low	cRCT	Low	49% of parents attended all sessions	Post<br>Post<br>Post	Short<br>Medium<br>Long				○<br>○<br>○		
**Johnston 2006**<br>2 high intensity	iRCT	Unclear	Not reported	During	Long		○		○		2 of 4 comparisons showed improved language outcomes, and 1 of 3 comparisons showed poorer SEWB outcomes, in the intervention arm in the long-term whilst intervention was ongoing.
**Landry 2006**<br>2 high intensity	iRCT	Unclear	91% of parents completed all 10 visits plus 2 assessment visits	Post	Short		●	●	○		2 of 2 comparisons showed improved language outcomes, and 1 of 1 comparison showed improved cognatic outcome in the intervention arm.<br>3 of 6 comparisons showed improved SEWB outcomes in the intervention arm.<br>Reporting of interaction with birthweight incomplete.
**Miller 2015**<br>High	iRCT	Low	Adherence data currently being analysed	During	Long			○	○		
**Minkovitz 2003**<br>High	iRCT	Unclear	79% of parents received 4 or more services (of 16)	During	Long				○		
**Niccols 2008**<br>Moderate	iRCT	Unclear	58% attended 4 or more sessions (of 8 planned)	Post<br>Post	Immed<br>Short				○<br>○		
**Niccols 2009**<br>Moderate	iRCT	Unclear	Not reported	Post<br>Post	Immed<br>Short				○<br>●		2 of 4 comparisons immediately post-intervention term showed improved SEWB outcomes in intervention group.<br>3 of 4 comparisons showed improved SEWB outcomes in intervention group in short-term.
**Tsiantis 2000**<br>Moderate	cRCT	Unclear	Not reported	Post<br>Post<br>Post	Short<br>Medium<br>Long		 <br>ø		○<br>○<br>○		2 of 2 comparisons showed poorer language outcomes in the intervention arm in the long-term.<br>1 of 2 comparisons showed poorer SEWB outcomes in the intervention arm in the medium-term.<br>1 of 8 comparisons showed improved SEWB outcomes in the intervention arm in the long-term.<br>Incomplete reporting of the sub-group analyses.
**Wagner 2001**<br>High	iRCT	Unclear	44% of families still receiving services at 2 yrs	During	Long	○	○	○	○		Results also stratified by income; no significant interactions reported.

For peer review only

D2: Results stratified by whether intervention delivery was delivered to all families or geographically targeted

Study<br>Intervention intensity	Study design	Risk of bias	Adherence	Outcome measurement		Development outcomes					Additional detail on intervention effects or sub-group analyses
				Timing	When?	Motor	Lang	Cogn	SEWB	Overall	
**Studies comparing one intervention with usual care**											
**Delivered to all families**											
**Beeghly 1995**<br>2 low intensity	iRCT	Unclear	Not reported	Post	Short	○		○			Tested for interaction between intervention and parity, IUGR, "demographic" risk and maternal psychological risk; no significant interactions found.
**Cheng 2007**<br>Moderate	iRCT	Low	Not reported	Post	Short						For the short-term analysis, only sub-group analyses (by attachment quality) presented; results inconsistent.
				Post	Long				○		
**Christakis 2007**<br>Low	iRCT	Unclear	Not reported	Post	Short		○		○		Sub-group results: 2 of 3 comparisons in low income group showed improved SEWB outcomes in intervention group; test for interaction not presented
**Drotar 2008**<br>High	iRCT	Unclear	Not reported	During	Short				○		1 of 12 comparisons showed improved SEWB outcomes in the intervention arm in the long-term. Incomplete reporting of the sub-group analyses.
				During	Medium			○	○		
				During	Long		○	○	○		
**Feinberg 2008**<br>Moderate	iRCT	Unclear	80% attended at least 3 of 4 antenatal sessions<br>60% attended at least 3 of 4 postnatal sessions	Post	Short				○		3 of 5 comparisons in short term and 1 of 2 comparisons in medium term showed improved SEWB outcomes in intervention group.<br>Results presented in text suggest there may be interaction effects with gender, but there is incomplete reporting of the sub-group analyses.
				Post	Medium				○		
				Post	Long				○		
**Hiscock 2008**<br>Low	cRCT	Low	49% of parents attended all sessions	Post	Short				○		
				Post	Medium				○		
				Post	Long				○		
**Johnston 2006**<br>2 high intensity	iRCT	Unclear	Not reported	During	Long		○		○		2 of 4 comparisons showed improved language outcomes, and 1 of 3 comparisons showed poorer SEWB outcomes, in the intervention arm in the long-term whilst intervention was ongoing.
**Lobo 2012**<br>2 moderate intensity	iRCT	Unclear	Excluded individuals who did not perform intervention on at least 60% of expected days	During	Short	○					4 of 8 comparisons showed improved motor outcomes in the intervention arm in the short-term whilst intervention was ongoing.<br>5 of 5 comparisons showed improved motor outcomes in the intervention arm post-intervention.
				Post	Short	●					
**Miller 2015**<br>High	iRCT	Low	Adherence data currently being analysed	During	Long			○	○		
**Minkovitz 2003**<br>High	iRCT	Unclear	79% of parents received 4 or more services (of 16)	During	Long				○		
**Niccols 2008**	iRCT	Unclear	58% attended 4 or more	Post	Immed				○		

For peer review only - http://bmjopen.bmj.com/site/about/guidelines.xhtml

Moderate			sessions (of 8 planned)	Post	Short				○		
**Niccols 2009** Moderate	iRCT	Unclear	Not reported	Post	Immed				○		2 of 4 comparisons immediately post-intervention term showed improved SEWB outcomes in intervention group. 3 of 4 comparisons showed improved SEWB outcomes in intervention group in short-term.
				Post	Short				●		
**Santelices 2011** Moderate	iRCT	Unclear	Not reported	Post	Short				○		
**Tsiantis 2000** Moderate	cRCT	Unclear	Not reported	Post	Short				○		2 of 2 comparisons showed poorer language outcomes in the intervention arm in the long-term. 1 of 2 comparisons showed poorer SEWB outcomes in the intervention arm in the medium-term. 1 of 8 comparisons showed improved SEWB outcomes in the intervention arm in the long-term. Incomplete reporting of the sub-group analyses.
				Post	Medium				○		
				Post	Long		ø		○		
**Geographically targeted**											
**Chang 2015** Low	cRCT	Unclear	83% of mothers attended all visits	Post	Short	○	○	○		○	Improved cognitive outcome in intervention group on adjusting for potential confounders.
**Cupples 2011** High	iRCT	Low	Mean number of contacts = 8.5 (of 22 planned)	Post	Immed	○		○	○		
**Doyle 2011** 1 high & 1 moderate	iRCT	Unclear	High: Mean number of visits = 46<br>Moderate: Not reported	During	Short	○	○	○	○	○	3 of 12 comparisons showed improved cognitive outcomes, and 1 of 62 comparisons showed improved SEWB outcomes in the intervention arm, in the long-term whilst intervention was ongoing.
				During	Medium	○	○	○	○	○	
				During	Long	○	○	○	○	○	
**Griffith 2011** High	iRCT	Low	60% attended 8 or more sessions (of 12 planned)	Post	Short				○	○	
**High 2000** Low	iRCT	High	Mean number of visits = 3.4 (of 5 planned)	Post	Short		○				3 of 6 comparisons showed improved language outcomes in intervention group. Sub-group results: no differences seen in 13-17 month olds; 6 of 6 comparisons in 18-25 month olds showed improved language outcomes in intervention group; no test for interaction presented.
**Landry 2006** 2 high intensity	iRCT	Unclear	91% of parents completed all 10 visits plus 2 assessment visits	Post	Short		●	●	○		2 of 2 comparisons showed improved language outcomes, and 1 of 1 comparison showed improved cognatic outcome in the intervention arm. 3 of 6 comparisons showed improved SEWB outcomes in the intervention arm. Interaction with birthweight examined, but reporting of results incomplete.
**Wagner 2001** High	iRCT	Unclear	44% of families still receiving services at 2 yrs	During	Long	○	○	○	○		Results also stratified by income; no significant interactions reported.
**Wiggins 2004** Moderate (SHV)	iRCT	Low	Mean number of visits = 7 (of 12 planned)	Post	Immed					○	1 of 1 comparison showed improved language outcome and 1 of 1 comparison showed improved overall development in intervention group.
				Post	Short		●		○	●	
**Wiggins 2004** Low (CGS)	iRCT	Low	19% of women attended a group	Post	Immed					○	
				Post	Short		○		○	○	

For peer review only

D3: Results stratified by socio-economic status of the families included

Not enough information in a majority of papers to perform this comparison adequately.

For peer review only

PRISMA 2009 Checklist

Section/topic	#	Checklist item	Reported on page #
**TITLE**			
Title	1	Identify the report as a systematic review, meta-analysis, or both.	6
**ABSTRACT**			
Structured summary	2	Provide a structured summary including, as applicable: background; objectives; data sources; study eligibility criteria, participants, and interventions; study appraisal and synthesis methods; results; limitations; conclusions and implications of key findings; systematic review registration number.	2-3
**INTRODUCTION**			
Rationale	3	Describe the rationale for the review in the context of what is already known.	5-6
Objectives	4	Provide an explicit statement of questions being addressed with reference to participants, interventions, comparisons, outcomes, and study design (PICOS).	6
**METHODS**			
Protocol and registration	5	Indicate if a review protocol exists, if and where it can be accessed (e.g., Web address), and, if available, provide registration information including registration number.	6 and protocol uploaded
Eligibility criteria	6	Specify study characteristics (e.g., PICOS, length of follow-up) and report characteristics (e.g., years considered, language, publication status) used as criteria for eligibility, giving rationale.	6-7
Information sources	7	Describe all information sources (e.g., databases with dates of coverage, contact with study authors to identify additional studies) in the search and date last searched.	8
Search	8	Present full electronic search strategy for at least one database, including any limits used, such that it could be repeated.	Web appendix 1
Study selection	9	State the process for selecting studies (i.e., screening, eligibility, included in systematic review, and, if applicable, included in the meta-analysis).	8
Data collection process	10	Describe method of data extraction from reports (e.g., piloted forms, independently, in duplicate) and any processes for obtaining and confirming data from investigators.	8
Data items	11	List and define all variables for which data were sought (e.g., PICOS, funding sources) and any assumptions and simplifications made.	8
Risk of bias in individual studies	12	Describe methods used for assessing risk of bias of individual studies (including specification of whether this was done at the study or outcome level), and how this information is to be used in any data synthesis.	8

For peer review only

PRISMA 2009 Checklist

Section/topic	#	Checklist item	Reported on page #
Summary measures	13	State the principal summary measures (e.g., risk ratio, difference in means).	N/A (narrative synthesis)
Synthesis of results	14	Describe the methods of handling data and combining results of studies, if done, including measures of consistency (e.g., I^2) for each meta-analysis.	N/A (narrative synthesis)
Risk of bias across studies	15	Specify any assessment of risk of bias that may affect the cumulative evidence (e.g., publication bias, selective reporting within studies).	8
Additional analyses	16	Describe methods of additional analyses (e.g., sensitivity or subgroup analyses, meta-regression), if done, indicating which were pre-specified.	9
**RESULTS**			
Study selection	17	Give numbers of studies screened, assessed for eligibility, and included in the review, with reasons for exclusions at each stage, ideally with a flow diagram.	9 and Figure 1
Study characteristics	18	For each study, present characteristics for which data were extracted (e.g., study size, PICOS, follow-up period) and provide the citations.	9 and Table 1
Risk of bias within studies	19	Present data on risk of bias of each study and, if available, any outcome level assessment (see item 12).	Figures 2 and 3
Results of individual studies	20	For all outcomes considered (benefits or harms), present, for each study: (a) simple summary data for each intervention group (b) effect estimates and confidence intervals, ideally with a forest plot.	12-13 (narrative synthesis)
Synthesis of results	21	Present results of each meta-analysis done, including confidence intervals and measures of consistency.	N/A (narrative synthesis)
Risk of bias across studies	22	Present results of any assessment of risk of bias across studies (see Item 15).	12-13 and Table 3
Additional analysis	23	Give results of additional analyses, if done (e.g., sensitivity or subgroup analyses, meta-regression [see Item 16]).	13 and Web Appendix D

For peer review only

PRISMA 2009 Checklist

**DISCUSSION**			
Summary of evidence	24	Summarize the main findings including the strength of evidence for each main outcome; consider their relevance to key groups (e.g., healthcare providers, users, and policy makers).	14
Limitations	25	Discuss limitations at study and outcome level (e.g., risk of bias), and at review-level (e.g., incomplete retrieval of identified research, reporting bias).	15
Conclusions	26	Provide a general interpretation of the results in the context of other evidence, and implications for future research.	16
**FUNDING**			
Funding	27	Describe sources of funding for the systematic review and other support (e.g., supply of data); role of funders for the systematic review.	17

From: Moher D, Liberati A, Tetzlaff J, Altman DG, The PRISMA Group (2009). Preferred Reporting Items for Systematic Reviews and Meta-Analyses: The PRISMA Statement. PLoS Med 6(7): e1000097. doi:10.1371/journal.pmed1000097

For more information, visit: **www.prisma-statement.org**.

For peer review only

BMJ Open

Interventions that enhance health services for parents and infants to improve child development and social and emotional wellbeing in high-income countries: A systematic review

Journal:	*BMJ Open*
Manuscript ID	bmjopen-2016-014899.R2
Article Type:	Research
Date Submitted by the Author:	14-Jul-2017
Complete List of Authors:	Hurt, Lisa; Cardiff University School of Medicine, Division of Population Medicine<br>Paranjothy, Shantini; Cardiff University School of Medicine, Division of Population Medicine<br>Lucas, Patricia; Unversity of Bristol, School for Policy Studies<br>Watson, Debbie; University of Bristol, School for Policy Studies<br>Mann, Mala; Cardiff University, Specialist Unit for Review Evidence (SURE)<br>Griffiths, Lucy; UCL Great Ormond Street Institute of Child Health, Population, Policy and Practice Programme<br>Ginja, Samuel; Institute of Health and Society, Newcastle University<br>Paljarvi, Tapio; Cardiff University School of Medicine, Division of Population Medicine<br>Williams, Jo; Bristol City Council<br>Bellis, Mark; Bangor University, College of Health and Behavioural Sciences; Public Health Wales, Policy, Research and International Development<br>Lingam, Raghu; Institute of Health & Society, Newcastle University
**Primary Subject Heading**:	Evidence based practice
Secondary Subject Heading:	Health policy, Paediatrics, Public health, Epidemiology
Keywords:	Child development, Social and emotional wellbeing, Universal health services, Early intervention

SCHOLARONE™
Manuscripts

Interventions that enhance health services for parents and infants to improve child development and social and emotional wellbeing in high-income countries: A systematic review

Lisa Hurt[1], Shantini Paranjothy[1], Patricia J Lucas[2], Debbie Watson[2], Mala Mann[3], Lucy J Griffiths[4], Samuel Ginja[5], Tapio Paljarvi[1], Jo Williams[6], Mark Bellis[7], Raghu Lingam[5]

[1] Division of Population Medicine, Cardiff University School of Medicine, 5th Floor, Neuadd Meirionnydd, Heath Park, Cardiff CF14 4YS

Lisa Hurt Lecturer

Shantini Paranjothy Mansel Talbot Professor of Preventive Medicine

Tapio Paljarvi Research Fellow

[2] School for Policy Studies, University of Bristol, 8 Priory Rd, Bristol BS8 1TZ

Patricia J Lucas Reader

Debbie Watson Reader

[3] Specialist Unit for Review Evidence, Cardiff University, Neuadd Meirionnydd, Heath Park, Cardiff CF14 4YS

Mala Mann Information Specialist/Systematic Reviewer

[4] Population, Policy and Practice Programme, UCL Great Ormond Street Institute of Child Health, 30 Guilford St, London WC1N 1EH

Lucy J Griffiths Senior Research Associate

[5] Institute of Health and Society, Newcastle University, Baddiley-Clark Building, Newcastle upon Tyne NE2 4AX

Samuel Ginja Research Associate

Raghu Lingam Clinical Senior Lecturer/Honorary Consultant

[6] Bristol City Council, City Hall, PO Box 3176, Bristol BS3 9FS

Jo Williams Consultant in Child Public Health

[7] Public Health Wales, 2 Capital Quarter, Tyndall Street, Cardiff, CF10 4BZ

Mark Bellis Professor/Director of Policy, Research and Development

Correspondence to: L Hurt, hurtl@cardiff.ac.uk, 029 2068 7807

Word count: 5,520

For peer review only - http://bmjopen.bmj.com/site/about/guidelines.xhtml

Abstract

Background: Experiences in the first 1000 days of life have a critical influence on child development and health. Health services that provide support for families need evidence about how best to improve their provision.

Methods: We systematically reviewed the evidence for interventions in high-income countries to improve child development by enhancing health service contact with parents from the antenatal period to 24 months postpartum. We searched 15 databases and trial registers for studies published in any language between 01/01/1996 and 01/04/2016. We also searched 58 programme or organisation websites and the electronic table of contents of eight journals.

Results: Primary outcomes were motor, cognitive, and language development, and social-emotional wellbeing, measured to 39 months of age (to allow the interventions time to produce demonstrable effects). Results were reported using narrative synthesis, due to the variation in study populations, intervention design and outcome measurement. 22 of the 12,986 studies identified met eligibility criteria. Using GRADE criteria, the quality of evidence overall was moderate to low. There was limited evidence for intervention effectiveness: positive effects were seen in 1/6 studies for motor development, 4/11 for language development, 4/8 for cognitive development, and 3/19 for social-emotional wellbeing. However, most studies showing positive effects were at high/unclear risk of bias, within-study effects were inconsistent, and negative effects were also seen. Intervention content and intensity varied greatly, but this was not associated with effectiveness.

Conclusions: There is insufficient evidence that interventions currently available to enhance health service contacts up to 24 months postpartum are effective for improving child development. There is an urgent need for robust evaluation of existing interventions, and to develop and evaluate novel interventions to enhance the offer to all families.

Registration: The protocol for this systematic review was registered in the International Prospective Register of Systematic Reviews (PROSPERO CRD42015015468) on 12 January 2015.

For peer review only - http://bmjopen.bmj.com/site/about/guidelines.xhtml

Article summary

Strengths and limitations of this study

- To our knowledge, this is the first systematic review of interventions that enhance health services to improve child development outcomes including social and emotional wellbeing outcomes.
- We used a broad systematic search of the extensive literature in this field, and searched many sources in addition to database searches.
- We reviewed a larger number of primary studies than previous reviews of interventions in the early years. Our conclusion is consistent with these reviews.
- It was not possible to conduct a meta-analysis due to the variation in the types of interventions and methods used to measure outcomes.
- We do not report parental outcomes, and cannot comment on whether parents benefitted from these enhancements.

For peer review only

For peer review only - http://bmjopen.bmj.com/site/about/guidelines.xhtml

Introduction

Experiences in the first 1000 days of life have a crucial influence on child development and health.[1] Appropriate early child development (including physical, social and emotional, language and cognitive domains) has consistently been shown to be associated with good health and educational outcomes in childhood, and consequent health and employment outcomes in adulthood.[2-4] Adopting a life-course approach, including early intervention, is essential,[5] and investment is therefore needed in effective pre and postnatal services to optimise child health, wellbeing and developmental resilience.[6]

The content of health services to promote maternal and child health, delivered during pregnancy and the early years through primary care and home visits, varies across countries. A recent review suggested that the best services in Europe are "*characterized by personalised ongoing support during pregnancy, choice in birth arrangements, postnatal support and advice, and paid parental leave for mothers and fathers*".[7] In most high income settings, early years services also work to a "proportionate universalism" model where care is available to all, irrespective of need, with enhanced support offered to families at high risk of adverse outcomes.[1]

There is high-quality global evidence to support the effectiveness of many components of early years services including elements of antenatal care and centre-based preschool provision.[7] [8] Interventions to promote child development by enhancing routine health services in the early years have also been developed. However, most have been targeted at and evaluated with high-risk families or children with an identified condition.[9-11] An unacceptably high proportion of children in both high and low income settings do not achieve expected early learning goals before they start school,[12] and it has been argued that targeted approaches alone may not be sufficient.[13] Interventions to enhance contacts with all parents in existing services may be more effective in improving child development outcomes for several reasons. First, not all children who need support are identified by a targeted approach.[14] Targeting can lead to stigmatisation resulting in poor uptake or adherence.[15] Embedding interventions within an existing service, such as health visiting, which provides ongoing and consistent support for parents, may also improve the interaction between health professionals and parents and improve access to care at a

For peer review only - http://bmjopen.bmj.com/site/about/guidelines.xhtml

crucial time in their child's development, leading to improvements in child development outcomes.[11] A review of interventions in low and middle income settings noted that there was great diversity in both the scope and focus of research in this area, and concluded that parents in such settings "*need to be supported in providing nurturing care and protection in order for young children to achieve their developmental potential*".[16] However, the effectiveness of such interventions to enhance existing multi-disciplinary services in high income settings is not known.

Previous reviews of early interventions in high income settings fail to provide a full picture of interventions relevant to public health policy and practice because they do not provide a comprehensive examination of child development outcomes in the very early years (that is, the period during which the human brain develops most rapidly[17]). Neither does the evidence base to date include social and emotional wellbeing outcomes, nor are these consistently defined and articulated. The objective of this systematic review is to fill these gaps, by examining the effect of interventions designed to enhance health service contacts with all parents and children in the very early years (defined as the antenatal period to 24 months postpartum) on child development and social and emotional wellbeing outcomes. Our research question was developed in partnership with local policy makers, and provides evidence for policy.[18]

Methods

Protocol and registration

The protocol for this systematic review was registered in the International Prospective Register of Systematic Reviews (PROSPERO CRD42015015468) on 12 January 2015. This review is reported in accordance with PRISMA guidelines.[19]

Inclusion and exclusion criteria

We included randomised controlled trials (with individual or cluster randomisation) in any language that were published or unpublished. The interventions of interest were ones delivered within existing multi-disciplinary healthcare services that are the cornerstone of early years programmes and are available to all. The interventions may be delivered by a range of staff within these services. We included studies from the 76 countries and

For peer review only - http://bmjopen.bmj.com/site/about/guidelines.xhtml

territories classified by the World Bank in July 2014 as "high-income economies". Studies published in any language were eligible for inclusion.

To capture the effects of interventions delivered in the very early years, we included programmes that were delivered at any time from the antenatal period to 24 months postpartum. Given that some programmes continue beyond the child's second birthday, we specified that studies would be included if the mean age of the children at the start of the intervention was less than or equal to 24 months. To allow time for these interventions to produce demonstrable effects, we included studies that examined outcomes to 39 months of age (given that not all studies would manage to assess children on their 3rd birthday exactly).

Studies that selected participants from the general population or included all individuals from a specific neighbourhood (for example, an area-based programme defined on the basis of postcode or zip code, known as "geographically targeted" programmes in this review) were included. Studies were excluded if they selected participants based on individual risk factors (for example, an individually-assessed income threshold for participating families, or parental illness) or specific clinical sub-groups (such as preterm babies or children with specific diagnoses).

Interventions

We included interventions that were provided within the framework of the existing healthcare system. They could be designed to augment routine healthcare provision for all children in different ways, for example by improving the skills or parental capacity of the parents or the family, improving the interaction between health professionals and parents, improving access to healthcare for the parents or the child, or including elements designed to promote a specific area of child development. These included training modules designed to be delivered to parents with the intention of improving child development outcomes, or any resources (such as printed materials, films, Apps) that health professionals or their support workers could use in their work with parents. Interventions could be delivered at home, in group-based settings (for example, in general, obstetric or paediatric practice, in

For peer review only - http://bmjopen.bmj.com/site/about/guidelines.xhtml

hospitals or community settings), through telemedicine, or via a combination of different methods.

There is an argument that these different approaches should be separated into different systematic reviews (or indeed separated by outcome). We, like others,[10 16] chose to include these in a single review to avoid divisions that were arbitrary from a developmental or service delivery perspective, and to avoid multiple overlapping, small and/or empty reviews in a field with limited literature.

Outcomes

The outcomes were motor development (fine and gross), cognitive development, language development (receptive and expressive), social and emotional wellbeing; and global child development. We included studies that used validated tools to measure these outcomes. Where unvalidated tools were used, we considered to be secondary outcomes. Studies were included if they measured outcomes at any time between three months of age and 39 months postpartum (specifically, where the average age of the children at outcome measurement was 39 months or less).

Search strategy

We searched for articles published in any language between 01/01/1996 and 01/04/2016 in the following databases: Cochrane Central Register of Controlled Trials (CENTRAL); Medline; Embase; CINAHL; PsycINFO; Web of Science; Scopus; ASSIA; LiLACS; Sociological Abstracts; Social Services Abstract; and OpenGrey; and the following trial registers: ClinicalTrials.gov; UK Clinical Trials Gateway; and WHO International Clinical Trials Registry Platform. Given our focus on enhancement of existing health services, we restricted to studies published within 20 years of our study inception since health service change has been substantial in the mid to late 20th century. We used a combination of Medical Subject Headings and free text including terms for interventions to enhance health service contacts combined with terms relating to child development outcomes. Terms for the interventions included those that listed the professional delivering the programme (including “health visitor”, “community nurse”, “nurse”, “midwife”, “general practitioner”, “early years educator”, “parent educator”), and programme names that were already known to the review team. The

For peer review only - http://bmjopen.bmj.com/site/about/guidelines.xhtml

Medline search strategy is shown in Web Appendix A. We also searched websites of 58 relevant programmes and organisations, and the electronic table of contents (eTOC) of eight key journals for relevant studies published within the last two years (see Web Appendix B for a full list). Reference lists of included and key papers were reviewed, and authors contacted for additional data where necessary.

Study selection

All references identified by the searches were downloaded into Endnote and duplicates removed. Titles and abstracts were screened for inclusion independently by two of three reviewers (LH and LJG or SP). Full text versions were obtained for the papers potentially meeting the inclusion criteria and were screened independently by two of three reviewers (LH and LJG or SP). Disagreements were resolved through discussion and in consultation with others in the review group.

Data abstraction process

A data extraction form designed for the requirements of this review was used, which included details on the characteristics of the included studies, the interventions studied and assessment of risk of bias and GRADE criteria. Multiple publications and reports from the same trial were linked, and compared for completeness and contradictions. Data from each paper were extracted independently and in duplicate (completed by LH and LJG or SG or SP or TP).

Analysis

Risk of bias was assessed following Cochrane guidelines.[20] Due to variation in i) the populations studied, ii) the design of the interventions and iii) the wide range of outcome measures used (both in terms of the child development domains and/or the instruments used to assess the outcomes), it was not possible to conduct a meta-analysis and results were reported using narrative synthesis. We specified a priori that we would examine the results stratified by i) risk of bias, ii) the intensity of the intervention, iii) the age of the child at which the intervention was delivered, iv) whether the programme was available to all or geographically targeted, and v) socio-demographic characteristics of the families in the trial. We selected these variables as we hypothesised that they would help to identify the

For peer review only - http://bmjopen.bmj.com/site/about/guidelines.xhtml

characteristics of the interventions most likely to be effective (for example, if high intensity interventions were more effective than low intensity ones) or the populations in which they were most likely to be effective (for example, if programmes recruiting from defined neighbourhoods were more effective than those made available to all).

An assessment of the intensity of each intervention was conducted independently and in duplicate (completed by LH and LJG or SG or SP or TP) based on seven criteria: i) total number of visits; ii) total duration of the programme; iii) total number of contact hours; iv) frequency of visits; v) number of components; vi) whether components were delivered directly to parents and/or children; and vii) whether the components were delivered on a one-to-one basis or in a group session. Using these seven characteristics, we categorised the overall intensity for each intervention as “low”, “moderate” or “high”. Two review authors made this assessment using subjective determination rather than a predefined algorithm or a scoring system (as used in [21]), to allow for the diversity and complex combinations of components to be reflected in the categorisation. Finally, the quality of the overall evidence for each outcome was assessed using GRADE criteria.[22]

Public Involvement

This work was conducted in collaboration with the Bristol Network for Early Years Health and Wellbeing (www.bonee.org) and a range of stakeholders have been involved in the design and conduct of this initiative. Parents were not involved in the design and conduct of the review, but we are discussing the results and interpretation with parents.

Results

15,280 records were identified in the database searches (Figure 1). Searches of relevant programme and organisation websites, and eTOC searches yielded 83 additional records. Once all searches were combined and duplicates removed, 12,986 records remained. After title and abstract screening, 12,644 records that were outside the scope of the review were excluded (the vast majority of these because their intervention was targeted at families at high-risk of adverse outcomes or at children with identified conditions). Of the 342 records included in full text review, there were 22 RCTs that fulfilled our inclusion criteria (reported

For peer review only - http://bmjopen.bmj.com/site/about/guidelines.xhtml

in 40 publications[23-62]). We also identified three relevant ongoing trials.[63-65] Reasons for exclusion are provided in Figure 1.

Trial characteristics

The 22 included trials are described in Table 1. Three were cluster randomised (clinics[25 46] or healthcare workers[58]), with the remainder randomising individual mothers, parents or mother-child dyads. Ten were conducted in the United States of America, three in the United Kingdom, two in Canada, two in Ireland, one in Australia, one in Chile, one in Japan, and one was a multi-site study conducted in four Southern European countries. Chang (2015) was conducted in Antigua, Jamaica and St Lucia, and is included because Antigua is classified as a high-income country.

17 trials compared one intervention to usual care[25-28 36 37 41 44 46 50 51 54-56 58 59 61], although minor augmentations to usual care were made in six of these (for example, with some other information or services made available to parents who wished to access them[26 36 37 56 58 59]). One of the trials compared two different interventions with usual care.[61] In the remaining five trials, two interventions were compared with each other.[24 30 47-49] The timing of intervention delivery varied, from the first month of life only[24] to longer-term interventions, with eight studies including interventions that continued beyond the child's second birthday,[27 30 36 41 47 50 51 55] and the maximum intervention length being five years.[50] Studies ranged from 28 to 1593 participants: six included fewer than 100 participants; twelve included between 100 and 500; and three included more than 500 participants. One trial did not report the number of subjects recruited or analysed.[58] In 17 of the 22 trials, outcome data were available for 75% or more of those randomised. All of the trials offered coverage of the intervention to all families in the general population or within a neighbourhood or defined population (for example, recruitment occurred in hospitals serving areas with high levels of social disadvantage or the intervention was made available to all individuals within specific postcodes).[25 28 30 41 44 48 59 61] Three trials also included first time mothers only.

Six trials were classified as being at low risk of bias (all compared interventions with usual care), one was at high risk, and 15 had an unclear risk (Figures 2 and 3).

For peer review only - http://bmjopen.bmj.com/site/about/guidelines.xhtml

Intervention characteristics

28 interventions were examined in total (see Table 2: 2a for studies that included one intervention; and 2b for studies that compared two interventions). Most papers described the body of literature on which the intervention development had been based, but provided less detail on the proposed mechanisms of action of the intervention. Seven were of low intensity: short films followed by group discussions shown in health centre waiting rooms (Chang, 2a); sets of building blocks and activity hand-outs sent to parents by post (Christakis, 2a); "*literacy promoting anticipatory guidance*" by paediatricians (High, 2a); a brief parenting course (Hiscock, 2a); access to community groups (Wiggins, intervention 2, 2a); and two different methods for giving feedback to mothers on a neonatal behavioural assessment (Beeghly, 2b). Ten were of moderate intensity. These included one-to-one home visits (between five [Cheng, 2a] and twelve visits [Wiggins, 2a] in total), group sessions (up to eight in total [Feinberg, Niccols 2008, Niccols 2009, all 2a]), training for primary healthcare workers in interview techniques that encouraged consideration of child development (Tsiantis, 2a), training for parents in daily activities to promote motor development (Lobo, 2b) or a combination of different components (Santelices 2a, Doyle 2b). 11 interventions were of high intensity. They were classified as such because they included multiple components (up to a maximum of 8), and regular contact with parents over a sustained period of time or intensively for a shorter period of time. In the five studies that included two interventions, the interventions were of the same intensity in all but one (Doyle, which compared a medium intensity intervention with one of high intensity), and the aim of the studies was to compare different models of care with each other.

The mode of delivery of the intervention varied between trials. The intervention was delivered by health professionals in seven trials,[24-26 44 46 47 58] by other professionals (including "parent educators", "family visitors", or researchers) in eight trials,[30 36 48 50 54-56 59] by a mixture of health and other professionals in three trials,[37 41 51] and by peer mentors in one trial.[28] One trial examined one intervention delivered by health professionals and another delivered by community support groups.[61] In the remaining trials, one included materials delivered to parents by post,[27] and one examined training for parents by a physiotherapist to deliver a handling and positioning intervention.[49]

For peer review only - http://bmjopen.bmj.com/site/about/guidelines.xhtml

A full narrative summary of the results, including the tools used to assess the outcome in each trial and the estimates of intervention effects, is given in Web Appendix C. Many of the trials reported several measures of the same outcome and/or measured outcomes at different time points, resulting in multiple comparisons for each outcome. The findings are summarised by outcome in Table 3, and are described below. Effect estimates are given in the text below only for the studies found to be at low risk of bias. An effect direction plot[66] provides a visual display of the results across all outcome domains, ordered by risk of bias and the intensity of the intervention (Table 4).

Motor development outcomes

Six studies, including a total of 37 comparisons in 1276 participants, reported motor development outcomes using validated tools. The quality of the evidence was moderate. Three studies comparing one intervention with usual care showed no effect (972 participants, three comparisons), and three studies in which two interventions were compared (304 participants) showed no effect in 25 of 34 comparisons. The positive effects were all from one study of 28 infants who received a daily 15-minute handling and positioning intervention or a social interaction intervention for three weeks.[49] In addition to its small sample size, this study had an unclear risk of bias. Only one study at low risk of bias examined motor development outcomes.[28] This study found no difference in the mean scores for the psychomotor scores of the Bayley Scale of Infant Development between the intervention and control groups (mean difference 1.64, 95% CI -0.94 – 4.21, p=0.21).

Language development outcomes

Ten studies including a total of 43 comparisons examined this outcome, with nine using validated tools. The total number of participants for this outcome is unknown as one study did not report numbers,[58] but was in excess of 3,000. The quality of the evidence was low. Seven studies comparing one intervention with usual care showed no effect in ten comparisons, a positive effect in four comparisons and a negative effect (poorer language development in the intervention group) in two comparisons. Three studies which compared two interventions (632 participants) found no difference between the interventions in 23 comparisons and a positive effect in 4 comparisons. Only one study at low risk of bias examined language development outcomes.[61] This study compared two different

For peer review only - http://bmjopen.bmj.com/site/about/guidelines.xhtml

interventions with usual care (supportive health visiting [SHV, moderate intensity] and community groups [CGS, low intensity). This study found that fewer mothers in the SHV group expressed a worry about their child's speech than in the control group (risk ratio 0.46, 95% CI 0.23 - 0.93), and no difference in the number of number of mothers expressing worries about speech between CGS and control (risk ratio 1.22, 95% CI 0.78 - 1.92).

Cognitive development outcomes

Eight studies, including a total of 40 comparisons in 2245 participants, examined cognitive development outcomes. All used validated tools, except for one where videotaped interactions were coded for "*independent goal-directed play*".[48] The quality of the evidence was low. In five studies (1729 participants) comparing one intervention with usual care, there was no effect in 18 of 20 comparisons, and a positive effect in two comparisons. Three studies which compared two interventions (516 participants) found no difference between the interventions in 16 of 20 comparisons and a positive effect in four comparisons. Two studies at low risk of bias examined cognitive development outcomes. One study[28] found no difference in the mean scores in the intervention and control group for the mental development scores of the Bayley Scale of Infant Development (mean difference -0.81, 95% CI -2.81 – 1.16, p=0.42). The other[50] found no difference in mean scores between intervention and control on cognitive development using the British Ability Scale (mean score in intervention group -0.05 (SD 1.01) and in control group 0.03 (SD 0.99), Hedges g effect size -0.63, 95% CI -0.28 - 0.15, p=0.56).

Social and emotional wellbeing outcomes

These outcomes were examined in 156 comparisons in 18 trials (total participant numbers unknown as one study did not report participant numbers,[58] but was in excess of 5,000). Many different outcomes were examined (see Table C4 in the Web Appendices for details), with most assessed using validated tools (such as the Child Behaviour Checklist, the Infant Behaviour Questionnaire, the Parent-Infant Relationship Global Assessment, the Q-Sort Measure of the Security of Attachment, and social and emotional wellbeing scores from the Ages and Stages Questionnaire), and most focused on behavioural outcomes.

For peer review only - http://bmjopen.bmj.com/site/about/guidelines.xhtml

The quality of the evidence was low. In 15 studies comparing one intervention with usual care, there was no effect in 60 of 69 comparisons. In the three studies which compared two interventions (630 participants), there was no difference between the interventions in 82 of 87 comparisons, a positive effect in four comparisons, and a negative effect in one comparison. Six studies at low risk of bias examined social and emotional wellbeing outcomes, and none found a difference between intervention and control groups. The largest of these[46] found no difference in mean scores between intervention and control for externalising or internalising behaviours measured using the Child Behaviour Checklist at 3, 9 or 21 months post-intervention. For example, the adjusted mean difference for externalising behaviours at 3 months was 0.16 (95% CI -1.01 - 1.33, p=0.79), at 9 months was -0.79 (95% CI -2.27 - 0.69, p=0.30), and at 21 months was -0.80 (95% CI -2.2 - 0.6, p=0.26).

Overall child development outcomes

Four studies including a total of 12 comparisons in 1565 participants examined global estimates of child development. The quality of the evidence was moderate. Three studies (1414 participants) comparing one intervention with usual care found no effect in seven of eight comparisons based on validated measures of global child development (Griffith Mental Development Scale,[25] and mean score from the Schedule of Growing Skills II[41]). Two studies at low risk of bias examined this outcome. In one study[61], there was no difference between SHV and control (risk ratio 0.88, 95% CI 0.39-1.99) or CGS and control (risk ratio 0.57, 95% CI 0.22-1.52) in the mother's perception of whether her child's development was normal. However, mothers in the SHV group had fewer mean number of worries about their child's development than in the control group (mean difference -0.23, 95% CI -0.42 - -0.01), but there was no difference in the mean number of worries about their child's development between CGS and control (mean difference 0.13, 95% CI -0.10 - 0.36). The other study, comparing two interventions (151 participants), found no difference between the interventions in four comparisons (using the mean development score from the Ages and Stages Questionnaire).[30]

For peer review only - http://bmjopen.bmj.com/site/about/guidelines.xhtml

Sub-group effects reported within studies

Sub-groups examined within the individual studies included families of different incomes, and children with different characteristics (for example, low birth-weight versus normal birth-weight infants, see Tables 3 and 4). Some positive effects were seen, but the reporting of these analyses was generally incomplete, with an emphasis on positive intervention effects. No conclusions can therefore be drawn on sub-groups in this review.

Stratification of results across studies by risk of bias and intensity of interventions

Table 4 gives the effect direction plot, summarizing the results for each outcome, ordered by risk of bias and the intensity of the intervention. In the studies at low risk of bias, there was no intervention effect when either low or high intensity interventions were studied. Some positive effects were seen in the two trials of moderate intensity interventions, although in one, this was limited to sub-groups only (children with “disturbed” attachment at baseline),[26] and in the other, positive effects were not consistently seen.[61]

One study was classified as being at high risk of bias, and this examined a low intensity intervention.[44] Inconsistent positive intervention effects were seen in this study, with most of these in one sub-group only. The remaining studies were classified as being at unclear risk of bias, and there is no clear pattern the effects seen in these studies. Programme intensity does not appear to be associated with effectiveness in these studies, in that there is no evidence that higher intensity interventions are associated with more intervention effects.

Table 4 also summarises the uptake and adherence to intervention components. These factors were variable across studies and inconsistently reported. For example, for low intensity interventions, this ranged from only 19% of the women accessing the intervention at all (community support groups[61]) to 83% accessing every session.[25] Patterns of adherence to the moderate and high intensity interventions also varied.

No clear pattern in the results were seen when stratification by the other pre-specified variables was conducted (see Web Appendix D).

For peer review only - http://bmjopen.bmj.com/site/about/guidelines.xhtml

For peer review only

Discussion

The need for interventions to promote child development outcomes in all families has been clearly articulated. Using a broad systematic search of the extensive literature in this field, we found 22 RCTs examining the effect of interventions that enhance health service contacts from the antenatal period to 24 months postpartum. The interventions varied greatly in their content and intensity, and uptake, adherence and fidelity were not consistently reported. The quality of evidence for motor development and overall child development was moderate, and the majority of comparisons showed no intervention effect. The quality of evidence for language development, cognitive development, and social and emotional wellbeing was low. The majority of the comparisons for these outcomes showed no effect, and where positive impacts were observed, within-study effects were inconsistent. Studies that compared one intervention with usual care did not demonstrate more positive intervention effects than studies comparing two interventions. We conclude that there is insufficient evidence to suggest that the interventions reviewed here are effective at improving child development outcomes. The low-to-moderate quality of evidence overall suggests that there is a need for high quality robust trials to inform current health service delivery in this area.

The strength of our review was the broad search strategy, which encompassed many sources of information other than database searching. We are confident that we have identified most relevant studies (including three trials not yet published in peer-review journals). Although it was not possible to conduct a meta-analysis due to the variation in the types of interventions and methods used to measure outcomes, the narrative review – supplemented with the effect direction plot - provides a comprehensive picture of the limited evidence-base in this field.

To our knowledge this is the first systematic review of interventions which aim to enhance health service contacts to improve child development outcomes, including social and emotional wellbeing outcomes. Our conclusion is consistent with other reviews of early years interventions. For example, the Allen review[9] found that none of the interventions designed for universal use in the early years (defined as conception to school) had “best” quality evidence available to support them. A recent rapid review to update the evidence

For peer review only - http://bmjopen.bmj.com/site/about/guidelines.xhtml

for components of the Healthy Child Programme in England also found few studies of interventions aiming to promote child development outcomes in all families with children in the 0-5 age range.[10] We reviewed a larger number of primary studies than either of these previous publications. Previous studies have also examined the effects of programmes such as these on parental knowledge, attitudes or practices. We did not systematically review parental outcomes here, so cannot comment on whether parents benefitted from these interventions. However, we can conclude that – in these studies – any effects on the parents did not, in turn, lead to consistent improvements in child development outcomes.

Understanding how health service contacts can be enhanced to provide support for parents to achieve the best possible developmental outcomes for their children is necessary but challenging. Maternal and child health services consist of many components, many of these untested. Parents also access a wide variety of other forms of support, and the effects of these are poorly understood. Although the evidence-base examined in this review is limited, it does allow us to conclude that there is no convincing evidence that the interventions studied provide an additional benefit to the care currently provided in the settings included in these trials. There was also no evidence that interventions of high intensity confer more benefit than those of lower intensity as no dose-response relationship was evident: programmes of greater intensity (in terms of length, number or type of components) did not show more positive intervention effects than programmes of lower intensity. This is consistent with recent evidence for targeted interventions (such as the recent trial of the Family Nurse Partnership programme in the UK[67]) and has implications for commissioners of early years health services.

Many interventions currently incorporated into health services have not been adequately evaluated, and we recommend further research to generate this evidence. The methodological quality of many of the studies – or the reporting of their methods - was poor (as shown in Figures 2 and 3). Eight of 22 trials provided no detail on how their randomisation sequence was generated, and one reported using an inappropriate method. 13 provided no detail of allocation concealment, and one reported using an inappropriate method. Ten relied on parental reporting of outcomes only, and a further five used a mix of parental reporting and observations. Although blinding of outcome assessment can be a

For peer review only - http://bmjopen.bmj.com/site/about/guidelines.xhtml

challenge in studies that rely on parental reporting of their child's development, validated measures of assessing children's development without using parental report (for example, coding of videotaped interactions as used in [26 54 55 58]) exist and we would encourage their use in research of this kind. We had also hoped that this review would advance our knowledge on the types of social and emotional well-being outcomes that can be influenced by interventions of this kind. However, this was not possible given that the outcomes included were not well-defined or consistent, and mainly measured behaviour. Future studies that aim to measure effects on social and emotional wellbeing in young children need better articulation of their conceptual definitions of the social-emotional domains targeted[68] and the proposed mechanisms of action of the intervention. Finally, 15 studies did not publish a protocol or provide evidence of trial registration, and two did not report on all outcomes described in the Methods section of the paper. Improvements in trial registration and a priori specification of analysis plans are needed in trials in this field.

We also note that adherence was poor across studies, and inconsistently reported. Future research should carefully report uptake, adherence and fidelity (particularly whether parents have received the intervention in sufficient dose), to further develop our understanding of the mechanism of action of these programmes and how to engage and retain families.[69 70] Involvement of parents from the design stage onwards is essential to improve engagement of families within these important research studies.[71] Recent work has shown that monetary incentives can also increase participant retention in RCTs.[72] Research is also needed on whether new delivery platforms (such as technology-assisted interventions[73]) may provide a more engaging, feasible and cost-effective mechanism for providing support to parents.

There have been calls for new public health models of interventions to enhance early child development within existing healthcare systems.[74] As shown in our review, however, the current evidence base for interventions delivered to all families is lacking. It is unclear from the literature reviewed why programmes had limited impact on child developmental outcomes. However, many of the interventions relied on parents to change their behaviours and action in relation to their children and were educational in tone, but did not have a theoretical framework or a sound basis in behaviour change mechanisms.[75] Additionally,

For peer review only - http://bmjopen.bmj.com/site/about/guidelines.xhtml

authors did not always report on a clear formative research phase or logic model. Future studies should follow guidance on the development and evaluation of complex interventions (such as the Medical Research Council's guidance[76]) The results of all phases of intervention development also need to be published alongside trial results, as current studies alone do not allow us to fully understand why interventions have not produced expected effects.

Currently, there is insufficient evidence that, where health services are available to all families with very young children, additional elements or enhancements to these improve child development outcomes. Early intervention to improve child development is a public health priority, but funding is scarce. There is an urgent need for more robust evaluation of existing interventions, and to develop and evaluate novel intervention packages to enhance the offer to all families.

Competing interests

All authors have completed the Unified Competing Interest form at www.icmje.org/coi_disclosure.pdf (available on request from the corresponding author) and declare no financial relationships with any organisations that might have an interest in the submitted work in the previous three years, and no other relationships or activities that could appear to have influenced the submitted work.

Contributorship statement

LH, SP, PL, DW, JW and RL conceived and designed the study. MM designed the search strategy (in consultation with other review authors) and performed the database searches. LH and LJG searched the websites and journal table of contents. LH and LJG selected and reviewed eligible reports. LH, LJG, SG, SP and TP extracted data. LH drafted the paper. All authors commented on and revised the paper, and approved the final version. LH is the guarantor for the paper.

Funding

This work was funded by Public Health Wales. The Director of Policy, Research and Development at Public Health Wales (Professor Mark Bellis) provided expert technical

For peer review only - http://bmjopen.bmj.com/site/about/guidelines.xhtml

advice during discussions on the study design, the interpretation of the results, and the drafting of the paper, and is an author on the paper.

Data sharing statement

This paper reports on a systematic review. No original study data was obtained from the authors of the trials included in the review. Extra data (such as the results of the review stratified by characteristics other than those included in the report) is available by emailing Lisa Hurt.

For peer review only

For peer review only - http://bmjopen.bmj.com/site/about/guidelines.xhtml

References

1. Marmot M, Allen J, Goldblatt P, et al. Fair Society, Healthy Lives: The Marmot Review. London: University College London, 2010.
2. Rough E, Goldblatt P, Marmot M, et al. Inequalities in child health. In: Foyle G, Nathanson V, eds. Growing up in the UK - Ensuring a healthy future for our children. London: British Medical Association, 2013:37-56.
3. Shonkoff, J, Phillips, D (eds). *From Neurons to Neighborhoods: The Science of Early Childhood Development.* Washington, DC.: National Academy Press, 2000.
4. Stewart-Brown S, Fletcher L, Wadsworth M. Parent-child relationships and health problems in adulthood in three UK national birth cohort studies. European Journal of Public Health 2005;**15**(6):640-6.
5. World Health Organization. The Minsk Declaration: The Life-Course Approach in the Context of Health 2020. Denmark: WHO Regional Office for Europe, 2015.
6. Irwin L, Siddiqui A, Hertzman C. Early Child Development: A Powerful Equalizer. Final Report for the World Health Organization's Commission on the Social Determinants of Health. Vancouver, Canada: Human Early Learning Partnership, University of British Columbia., 2007.
7. Jensen B, Currie C, Dyson A, et al. European Review of Social Determinants of Health and the Health Divide in the WHO European Region. Copenhagen, Denmark: WHO Regional Office for Europe, 2013.
8. DiMario S, Basevi V, Gori G, et al. What is the effectiveness of antenatal care? (Supplement). Health Evidence Network report. Copenhage, Denmark: WHO Regional Office for Europe, 2005.
9. Allen G. Early Intervention: The Next Steps. Londo, UK: Cabinet Office, 2011.
10. Axford N, Barlow J, Coad J, et al. Rapid Review to Update Evidence for the Healthy Child Programme 0–5. London, UK: Public Health England, 2015.
11. Barlow J, Smailagic N, Ferriter M, et al. Group-based parent-training programmes for improving emotional and behavioural adjustment in children from birth to three years old. Cochrane Database of Systematic Reviews 2010;**Issue 3.**:Art. No.: CD003680.
12. Britto P. School Readiness: a conceptual framework. New York, USA: United Nations Children's Fund, 2012.

For peer review only - http://bmjopen.bmj.com/site/about/guidelines.xhtml

13. Sanders M, Kirby J. A Public-Health Approach to Improving Parenting and Promoting Children's Well-Being. Child Development Perspectives 2014;**8**(4):250-7.

14. Bayer J, Hiscock H, Morton-Allen E, et al. Prevention of mental health problems: rationale for a universal approach. Archives of Diseases in Childhood 2007;**92**:34-8.

15. Prinz R, Sanders M. Adopting a population-level approach to parenting and family support interventions. Clinical Psychology Review 2007;**27**(6):739-49.

16. Britto P, Lye S, Proulx K, et al. Nurturing care: promoting early childhood development. Lancet 2017;**389**:91-102.

17. Walker S, Wachs T, Grantham-McGregor S, et al. Inequality in early childhood: risk and protective factors for early child development. Lancet 2011;**378**:1325–38.

18. Nutley S, Walter I, Davies H. *Using evidence: How research can inform public services.* Bristol: Policy Press, University of Bristol, 2007.

19. Liberati A, Altman D, Tetzlaff J, et al. The PRISMA statement for reporting systematic reviews and meta-analyses of studies that evaluate health care interventions: explanation and elaboration. PLoS Med 2009;**6**(7):e1000100.

20. Higgins J, Green S, editors. *Cochrane Handbook for Systematic Reviews of Interventions Version 5.1.0 [updated March 2011].* The Cochrane Collaboration, 2011.

21. Baker P, Francis D, Soares J, et al. Community wide interventions for increasing physical activity. Cochrane Database of Systematic Reviews 2015;**Issue 1**:Art. No.: CD008366.

22. Guyatt G, Oxman A, Akl E, et al. GRADE guidelines: 1. Introduction—GRADE evidence profiles and summary of findings tables Journal of Clinical Epidemiology 2011;**64**(4):383–94.

23. Bayer J, Hiscock H, Ukoumunne O, et al. Three-year-old outcomes of a brief universal parenting intervention to prevent behaviour problems: randomised controlled trial. Archives of Diseases in Childhood 2010;**95**:187–92.

24. Beeghly M, Brazelton T, Flannery K, et al. Specificity of preventative pediatric intervention effects in early infancy. Developmental and Behavioral Pediatrics 1995;**16**(3):158-66.

25. Chang S, Grantham-McGregor S, Powell C, et al. Integrating a Parenting Intervention With Routine Primary Health Care: A Cluster Randomized Trial. Pediatrics 2015;**136**(2):272-80.

For peer review only

For peer review only - http://bmjopen.bmj.com/site/about/guidelines.xhtml

26. Cheng S, Kondo N, Aoki Y, et al. The effectiveness of early intervention and the factors related to child behavioural problems at age 2: A randomized controlled trial. Early Human Development 2007;**83**:683–91.

27. Christakis D, Zimmerman F, Garrison M. Effect of Block Play on Language Acquisition and Attention in Toddlers. A Pilot Randomized Controlled Trial. Arch Pediatr Adolesc Med 2007;**161**(10):967-71.

28. Cupples M, Stewart M, Percy A, et al. A RCT of peer-mentoring for first-time mothers in socially disadvantaged areas (The MOMENTS Study). Archives of Diseases in Childhood 2011;**96**:252–58.

29. Doyle O. Barrington Lecture 2011-12. Breaking the Cycle of Deprivation: An Experimental Evaluation of an Early Childhood Intervention. Journal of the Statistical and Social Inquiry Society of Ireland 2012;**XLI**:92-111.

30. Doyle, O. and the Preparing for Life Evaulation Team. Preparing for Life: Early Childhood Intervention. Assessing the Early Impact of Preparing for Life at Six Months. Dublin, Ireland: UCD Geary Institute, 2011.

31. Doyle, O. and the Preparing for Life Evaulation Team. Preparing For Life: Early Childhood Intervention. Assessing the Impact of Preparing For Life at Twelve Months. Dublin, Ireland: UCD Geary Institute, 2012.

32. Doyle, O. and the Preparing for Life Evaulation Team. Preparing For Life:Early Childhood Intervention. Assessing the Impact of Preparing For Life at Eighteen Months. Dublin, Ireland: UCD Geary Institute, 2013.

33. Doyle, O. and the Preparing for Life Evaulation Team. Preparing For Life: Early Childhood Intervention. Assessing the Impact of Preparing For Life at Twenty-four Months. Dublin, Ireland: UCD Geary Institute, 2013.

34. Doyle, O. and the Preparing for Life Evaulation Team. Preparing For Life: Early Childhood Intervention. Assessing the Impact of Preparing For Life at Thirty-six Months. Dublin, Ireland: UCD Geary Institute, 2014.

35. Doyle O, McGlanaghy E, O'Farrelly C, et al. Can Targeted Intervention Mitigate Early Emotional and Behavioral Problems?: Generating Robust Evidence within Randomized Controlled Trials. PLOS One 2016;**11**(6):e0156397.

For peer review only - http://bmjopen.bmj.com/site/about/guidelines.xhtml

36. Drotar D, Robinson J, Jeavons L, et al. A randomized, controlled evaluation of early intervention: the Born to Learn curriculum. Child: Care, Health and Development 2008;**35**(5):643–49.

37. Feinberg M, Kan M. Establishing Family Foundations: Intervention Effects on Coparenting, Parent/Infant Well-Being, and Parent–Child Relations. Journal of Family Psychology 2008;**22**(2):253–63.

38. Feinberg M, Kan M, Goslin M. Enhancing Coparenting, Parenting, and Child Self-Regulation: Effects of Family Foundations 1 Year after Birth. Prevention Science 2009;**10**:276–85.

39. Feinberg M, Jones D, Kan M, et al. Effects of Family Foundations on Parents and Children: 3.5 Years After Baseline. Journal of Family Psychology 2010;**24**:532–42.

40. Feinberg M, Jones D, Roettger M, et al. Long-Term Follow-Up of a Randomized Trial of Family Foundations: Effects on Children's Emotional, Behavioral, and School Adjustment. Journal of Family Psychology 2014;**28**(6):821–31.

41. Griffith N. Evaluating the Incredible Years Toddler programme in disadvantaged areas of Wales. Bangor University, 2011.

42. Gridley N, Hutchings J, Baker-Henningham H. The Incredible Years Parent–Toddler Programme and parental language: a randomised controlled trial. Child: Care, Health and Development 2015;**41**(1):103–11.

43. Guyer B, Hughart N, Strobino D, et al. Assessing the Impact of Pediatric-Based Developmental Services on Infants, Families, and Clinicians: Challenges to Evaluating the Healthy Steps Program. Pediatrics 2000;**105**(3):e33.

44. High P, LaGasse L, Becker S, et al. Literacy Promotion in Primary Care Pediatrics: Can We Make a Difference? Pediatrics 2000;**105**(4):927–34.

45. Hiscock H, Bayer J, Wake M. Preventing Toddler Externalising Behaviour Problems: Pilot Evaluation of a Universal Parenting Program. International Journal of Mental Health Promotion 2005;**7**(2):54-60.

46. Hiscock H, Bayer J, Price A, et al. Universal parenting programme to prevent early childhood behavioural problems: cluster randomised trial. British Medical Journal 2008;**336**:318.

For peer review only - http://bmjopen.bmj.com/site/about/guidelines.xhtml

47. Johnston B, Huebner C, Anderson M, et al. Healthy Steps in an Integrated Delivery System. Child and Parent Outcomes at 30 Months. Arch Pediatr Adolesc Med 2006;**160**:793-800.

48. Landry S, Smith K, Swank P. Responsive Parenting: Establishing Early Foundations for Social, Communication, and Independent Problem-Solving Skills. Developmental Psychology 2006;**42**(4):627–42.

49. Lobo M, Galloway J. Enhanced Handling and Positioning in Early Infancy Advances Development Throughout the First Year. Child Development 2012;**83**(4):1290–302.

50. Miller S, Dunne L. A Randomised Controlled Trial Evaluation of the Lifestart Parenting Programme. Belfast, Northern Ireland: Queen's University, 2015.

51. Minkovitz C, Strobino D, Hughart N, et al. Early effects of the Healthy Steps for Young Children Program. Arch Pediatr Adolesc Med 2001;**155**:470-79.

52. Minkovitz C, Hughart N, Strobino D, et al. A practice-based intervention to enhance quality of care in the first 3 years of life. The Healthy Steps for Young Children Program Journal of the American Medical Association 2003;**290**(23):3081-91.

53. Zuckerman B, Parker S, Kaplan-Sanoff M, et al. Healthy Steps: A Case Study of Innovation in Pediatric Practice. Pediatrics 2004;**114**(3):820–26.

54. Niccols A. 'Right from the Start': randomized trial comparing an attachment group intervention to supportive home visiting. Journal of Child Psychology and Psychiatry 2008;**49**(7):754–64.

55. Niccols A. Immediate and short-term outcomes of the 'COPEing with Toddler Behaviour' parent group. Journal of Child Psychology and Psychiatry 2009;**50**(5):617–26.

56. Santelices M, Guzmán M, Aracena M, et al. Promoting secure attachment: evaluation of the effectiveness of an early intervention pilot programme with mother–infant dyads in Santiago, Chile. Child: Care, Health and Development 2010;**37**(2):203–10.

57. Tsiantis J, Dragonas T, Cox A, et al. Promotion of children's early psychosocial development through primary health care services. Paediatric and Perinatal Epidemiology 1996;**10**:339-54.

58. Tsiantis J, Smith M, Dragonas T, et al. Early Mental Health Promotion in Children Through Primary Health Care Services: A Multi-Centre Implementation. Journal of Public Mental Health 2000;**2**(3):5-15.

For peer review only - http://bmjopen.bmj.com/site/about/guidelines.xhtml

59. Wagner M, Spiker D, Hernandez F, et al. Multisite Parents as Teachers evaluation. Experiences and outcomes for children and families. California, USA: SRI International, 2001.

60. Wagner M, Spiker D, Linn M. The Effectiveness of the Parents as Teachers Program with Low-Income Parents and Children. Topics in Early Childhood Special Education 2002;**22**(2):67-81.

61. Wiggins M, Oakley A, Roberts I, et al. The Social Support and Family Health Study: a randomised controlled trial and economic evaluation of two alternative forms of postnatal support for mothers living in disadvantaged inner-city areas. Health Technology Assessment 2004;**8**(32):1-134.

62. Wiggins M, Oakley A, Roberts I, et al. Postnatal support for mothers living in disadvantaged inner city areas: a randomised controlled trial. J Epidemiol Community Health 2005;**59**:288–95.

63. Hiscock H, Bayer J, Lycett K, et al. Preventing mental health problems in children: the Families in Mind population-based cluster randomised controlled trial. BMC Public Health 2012;**12**:420.

64. Pontoppidan M. The effectiveness of the Incredible Years™ Parents and Babies Program as a universal prevention intervention for parents of infants in Denmark: study protocol for a pilot randomized controlled trial. Trials 2015;**16**:386.

65. Sawyer A, Lynch J, Bowering K, et al. An equivalence evaluation of a nurse-moderated group-based internet support program for new mothers versus standard care: a pragmatic preference randomised controlled trial. BMC Pediatrics 2014;**14**:119.

66. Thomson H, Thomas S. The effect direction plot: visual display of non-standardised effects across multiple outcome domains. Res Syn Meth 2013;**4**:95–101.

67. Robling M, Bekkers M-J, Bell K, et al. Effectiveness of a nurse-led intensive home-visitation programme for first-time teenage mothers (Building Blocks): a pragmatic randomised controlled trial. The Lancet 2016;**387**(10014):146–55.

68. Denham S, Wyatt T, Bassett H, et al. Assessing social-emotional development in children from a longitudinal perspective. J Epidemiol Community Health 2009;**63(Suppl I)**:i37–i52.

69. Ingoldsby I. Review of interventions to improve family engagement and retention in parent and child mental health programs. J Child Fam Stud 2010;**19**(5):629–45.

For peer review only - http://bmjopen.bmj.com/site/about/guidelines.xhtml

780 70. Cullen S, Cullen M-A, Lindsay G. Universal parenting programme provision in England; Barriers to parent engagement in the CANparent trial, 2012-2014. Children and Society 2016;**30**:71-81.

783 71. Hutton E, Coxon K. Involving parents as service users in an interprofessional research project. Journal of Interprofessional Care 2008;**22**(6):661-63.

785 72. Brueton V, Tierney J, Stenning S, et al. Strategies to improve retention in randomised trials: a Cochrane systematic review and meta-analysis. BMJ Open 2014;**4**:e003821.

787 73. Hall C, Bierman K. Technology-assisted interventions for parents of young children: Emerging practices, current research, and future directions. Early Childhood Research Quarterly 2015;**33**:21-32.

790 74. Reilly S, McKean C, Morgan A, et al. Identifying and managing common childhood language and speech impairments. BMJ 2015;**350**:h2318.

792 75. Michie S, van Stralen M, West R. The behaviour change wheel: A new method for characterising and designing behaviour change interventions. Implement Sci 2011;**6**:42.

795 76. Craig P, Dieppe P, Macintyre S, et al. Developing and evaluating complex interventions: the new Medical Research Council guidance. BMJ 2008;**337**:a1655.

For peer review only

For peer review only - http://bmjopen.bmj.com/site/about/guidelines.xhtml

Table 1: Characteristics of the included studies

Study<br>Setting	Type and aim of study	Comparison group	i) Who received intervention, and<br>ii) When?	Sample size<br>i) Randomised<br>ii) In analysis (% of randomised)	Universal or geographically targeted?	Outcome domains measured*
**Beeghly 1995**<br>USA	Individual RCT, to compare the effectiveness of two one-to-one clinic-based interventions (infant-centred vs mother centred) on motor and cognitive development post-intervention	Two interventions compared	i) Mothers and infant<br>ii) When child was 3, 14 and 30 days of age	i) 163<br>ii) 125 (77%)	Universal	Motor<br>Cognitive
**Chang 2015**<br>Antigua, Jamaica & St Lucia	Cluster RCT, to examine the effectiveness of a group-based intervention on language and cognitive development post-intervention	Usual care	i) Mothers<br>ii) When child was 3, 6, 9, 12 and 18 months	i) 30 health centres randomised (501 women enrolled)<br>ii) 426 (85%)	Geographically targeted	Motor<br>Language<br>Cognitive<br>Overall
**Cheng 2007**<br>Japan	Individual RCT, to examine the effectiveness of an individual home-based intervention on social and emotional wellbeing post-intervention	Usual care, with a counselling service made available to all	i) Mothers<br>ii) When child was 5-9 months	i) 95<br>ii) 85 (89%)	Universal	SEWB
**Christakis 2007**<br>USA	Individual RCT, to examine the effectiveness of giving 2 sets of building blocks and a newsletter of activities to complete with them on language development and social and emotional wellbeing post-intervention	Usual care	i) Families<br>ii) When child was 18-30 months	i) 175<br>ii) 140 (80%)	Universal	Language<br>SEWB
**Cupples 2011**<br>UK (Northern Ireland)	Individual RCT, to examine the effectiveness of one-to-one contact with trained peer mentors on motor, cognitive development and social and emotional wellbeing post-intervention	Usual care	i) Mothers<br>ii) From 20 weeks of pregnancy to 12 months postpartum	i) 343<br>ii) 280 (82%)	Geographically targeted (First time mothers only)	Motor<br>Cognitive<br>SEWB
**Doyle 2011**<br>Ireland	Individual RCT, to compare the effectiveness of the "high support" versus "low support" versions of the multi-component "Preparing for Life" programme on motor, language, and cognitive development and social and emotional wellbeing whilst intervention was ongoing	Two interventions compared	i) Parents<br>ii) From pregnancy to school entry	i) 233<br>ii) 173 (74% at 6 months)<br>165 (71% at 12 months)<br>154 (66% at 18 months)<br>166 (71% at 24 months)<br>151 (65% at 36 months)	Geographically targeted	Motor<br>Language<br>Cognitive<br>SEWB<br>Overall
**Drotar 2008**<br>USA	Individual RCT, to examine the effectiveness of the multi-component "Born to Learn" programme on language and cognitive development and social and emotional wellbeing whilst intervention was ongoing	Usual care, plus handouts and offer of a different group meeting	i) Parents and child<br>ii) Recruited between birth and 9 months; programme continued to age 3	i) 527<br>ii) 410 (78%, although inconsistent numbers presented in tables)	Universal	Language<br>Cognitive†<br>SEWB
**Feinberg 2008**<br>USA	Individual RCT, to examine the effectiveness of a group-based intervention ("Family Foundations") on social and emotional wellbeing post-intervention	Usual care plus brochure on childcare options	i) Parents<br>ii) Recruited during pregnancy, continued to age 4-6 months	i) 169<br>ii) 152 (90% at 6 months)<br>154 (91% at 12 months)<br>137 (81% at 36 months)	Universal (First time mothers only)	SEWB†

For peer review only - http://bmjopen.bmj.com/site/about/guidelines.xhtml

Study<br>Setting	Type and aim of study	Comparison group	i) Who received intervention, and<br>ii) When?	Sample size<br>i) Randomised<br>ii) In analysis (% of randomised)	Universal or geographically targeted?	Outcome domains measured*
**Griffith 2011**<br>UK (Wales)	Individual RCT, to examine the effectiveness of a group-based parenting intervention ("Incredible Years Toddler Programme") on social and emotional wellbeing and overall development post-intervention	Usual care (waiting list control group)	i) Parents<br>ii) Children 12-36 months at baseline (mean age 21 months)	i) 89<br>ii) 89 (100%)	Geographically targeted	SEWB<br>Overall
**High 2000**<br>USA	Individual RCT, to examine the effectiveness of a one-to-one clinic-based intervention on language development post-intervention	Usual care	i) Parents<br>ii) Children 5-11 months at baseline	i) 205<br>ii) 153 (75%)	Geographically targeted	Language
**Hiscock 2008**<br>Australia	Cluster RCT, to examine the effectiveness of a group-based intervention ("Toddlers Without Tears") on social and emotional wellbeing post-intervention	Usual care	i) Parents<br>ii) When child was 8, 12 and 15 months	i) 40 maternal and child health centres randomised (733 women enrolled)<br>ii) 672 (92% at 18 months)<br>656 (89% at 24 months)<br>589 (80% at 36 months)	Universal	SEWB
**Johnston 2006**<br>USA	Individual RCT, to compare the effectiveness of the multi-component "Healthy Steps" programme with "Healthy Steps" plus "PrePare" on language development and social and emotional wellbeing post-intervention	Two interventions compared	i) Mothers<br>ii) Recruited during pregnancy, continued to age 3	i) 303<br>ii) 239 (79%)	Universal	Language<br>SEWB
**Landry 2006**<br>USA	Individual RCT, to compare the effectiveness of two different models of home visits, on language and cognitive development and social and emotional wellbeing post-intervention	Two interventions compared	i) Mothers<br>ii) When child was 6-10 months	i) 264<br>ii) 240 (91%)	Geographically targeted	Language‡<br>Cognitive‡<br>SEWB‡
**Lobo 2012**<br>USA	Individual RCT, to compare the effectiveness of a "handling and positioning" intervention with a “social interaction” intervention on motor development whilst the intervention was ongoing and post-intervention	Two interventions compared	i) Parents<br>ii) For 3 weeks, from when child was 2 months of age	i) 28<br>ii) 28 (100%)	Universal	Motor
**Miller 2015**<br>Ireland	Individual RCT, to examine the effectiveness of the multi-component "Lifestart" programme on cognitive development and social and emotional wellbeing whilst intervention was ongoing	Usual care	i) Parents<br>ii) Recruited when child < 12 months, continued to age 5	i) 435<br>ii) 347 (80% at 36 months)	Universal (although parents self-referred)	Cognitive<br>SEWB
**Minkovitz 2003**<br>USA	Individual RCT, to examine the effectiveness of the multi-component "Healthy Steps" programme on social and emotional wellbeing in the long term whilst intervention was ongoing	Usual care	i) Families<br>ii) Recruited at birth or 1st well-child visit, continued to age 3	i) 2235<br>ii) 1593 (71%)	Universal	SEWB

For peer review only - http://bmjopen.bmj.com/site/about/guidelines.xhtml

Study Setting	Type and aim of study	Comparison group	i) Who received intervention, and ii) When?	Sample size i) Randomised ii) In analysis	Universal or geographically targeted?	Outcome domains measured*
**Niccols 2008** Canada	Individual RCT, to examine the effectiveness of a group-based intervention ("Right from the Start") on social and emotional wellbeing post-intervention	Usual care	i) Mothers<br>ii) Children 1-24 months at baseline	i) 76<br>ii) 73 (96% immediately post intervention)<br>64 (84% at 6 months)	Universal (although parents self-referred)	SEWB
**Niccols 2009** Canada	Individual RCT, to examine the effectiveness of a group-based intervention ("COPEing with Toddler Behaviour") on social and emotional wellbeing post-intervention	Usual care (waiting list control group)	i) Mothers<br>ii) Children 12-36 months at baseline (mean age 24 months)	i) 79<br>ii) 74 (94% immediately post-intervention)<br>71 (90% at 1 month)	Universal (although parents self-referred)	SEWB†
**Santelices 2011** Chile	Individual RCT, to examine the efficacy of a multi-component intervention ("Promoting Secure Attachment") on social and emotional wellbeing post-intervention	Usual care, plus one lecture by a psychologist	i) Mothers<br>ii) Recruited during late pregnancy, continued to age 1	i) 100<br>ii) 72 (72%)	Universal (First time mothers only)	SEWB
**Tsiantis 2000** Cyprus, Greece, Portugal, Yugoslavia	Cluster RCT, to examine the effectiveness of training primary health care workers to use semi-structured interviews to promote language development and social and emotional wellbeing whilst intervention is ongoing	Usual care (healthcare workers in this group received one lecture)	i) Mothers<br>ii) Recruited during pregnancy, continued to age 2	i) 80 primary healthcare workers (number of women randomised not reported, "recruitment did not achieve target figures")<br>ii) Not reported	Universal	Language<br>SEWB†
**Wagner 2001** USA	Individual RCT, to examine the effectiveness of a multi-component intervention ("Parents as Teachers") on motor, language, and cognitive development and social and emotional wellbeing whilst intervention is ongoing	Usual care, plus age-appropriate toys "at regular intervals" and an annual child assessment	i) Mothers<br>ii) Recruited during pregnancy, continued to age 2	i) 665<br>ii) 266 (40%)	Geographically targeted	Motor<br>Language<br>Cognitive<br>SEWB
**Wiggins 2004** UK (England)	Individual RCT, to examine the effectiveness of two postnatal social support interventions ("Support Health Visitors" [SHV] and "Community Group Support" [CGS]) on language development, social and emotional wellbeing overall development post-intervention	Both interventions compared with usual care	i) Mothers<br>ii) Recruited when child was ~ 10 weeks, continued to age 1	i) 731 (SHV 183, CGS 184, control 364)<br>ii) SHV comparison:<br>493 (91% at 12 months)<br>443 (81% at 18 months)<br>CGS comparison:<br>492 (90% at 12 months)<br>456 (83% at 18 months)	Geographically targeted	Language§<br>SEWB§<br>Overall§

Abbreviations: RCT = randomised controlled trial; SEWB = social and emotional wellbeing
* Used a validated questionnaire for measuring outcome unless indicated otherwise (although the use of the instrument may not always have been validated in the target population)
† Used a combination of validated questionnaires and coding of videotaped activities and behaviours (no validated coding framework described)
‡ Used coding of videotaped activities and behaviours (no validated coding framework described)
§ No validated measure used; asked parents whether they perceived their child's development to be normal and whether they had worries about specific areas of development (including speech and behaviour)

For peer review only - http://bmjopen.bmj.com/site/about/guidelines.xhtml

Table 2: Description of intervention components and intensity

Study	Description	Components*										Contacts: Number Frequency Duration	Who delivered the intervention?
		1	2	3	4	5	6	7	8	9	10		
**2a. Studies comparing one intervention with usual care**													
**Low intensity**													
**Chang 2015**	3 three-minute films demonstrating “*behaviours central to promoting child development*" shown as women waited for 3, 6, 9, 12 and 18 month vaccine visits, followed by group discussions with a community health worker. Cards given to reinforce messages, plus picture book at 9 and 12 months and puzzle at 18 months.			✓	✓			✓			✓	5<br>Every 3-6 months<br>Over 15 months	Health professionals
**Christakis 2007**	Parents were sent 2 sets of building blocks with accompanying newsletters containing suggested activities in the post				✓	✓		✓				2<br>Bi-monthly<br>3 months	No contact with families post-recruitment
**High 2000**	Paediatricians gave books, handouts and "*literacy promoting anticipatory guidance*" to parents at routine well-child visits		✓		✓			✓				5<br>Every 3 months<br>Over 12 months	Health professionals
**Hiscock 2008**	"*Universal anticipatory guidance*" with strategies for behavioural difficulties: handout at 8 months; 2 two-hour group sessions at 12 and 15 months			✓	✓							3<br>Every 3-4 months<br>Over 7 months	Health professionals
**Wiggins 2004 Intervention 2**	Access given to mothers to community group support that already existed and which provided drop in sessions and/or telephone support and/or home visits (different services provided by each of the 8 groups who agreed to take part in the study); participants selected whether to make contact and attend groups	✓		✓					✓			Variable	Other professionals
**Moderate intensity**													
**Cheng 2007**	5 one-to-one home visits of one hour “*aimed at improving the quality of mother-infant relationship*”; tailored encouragement and advice given following observation of mothers playing with infants	✓										5<br>Monthly<br>Over 5 months	Health professionals
**Feinberg 2008**	Four prenatal and four postnatal interactive group sessions, designed “*to enhance coparenting*”			✓								8<br>Every 6 weeks<br>Over 11 months	Mix of health and other professionals
**Niccols 2008**	8 two-hour group sessions using a "*coping modeling problem solving approach*", to enhance caregiver skills in "*reading infant cues and responding sensitively*" plus homework			✓								8<br>Weekly<br>Over 2 months	Other professionals

* 1 = One-to-one home visits, 2 = One-to-one clinic visits, 3 = Group sessions, 4 = Handouts, 5 = Activities to perform at home, 6 = Developmental screening, 7 = Toys and/or books, 8 = Telephone support, 9 = Access to community resources, 10 = Other

For peer review only - http://bmjopen.bmj.com/site/about/guidelines.xhtml

Study	Description	Components*										Contacts: Number Frequency Duration	Who delivered the intervention?
		1	2	3	4	5	6	7	8	9	10		
Moderate intensity													
**Niccols 2009**	8 two-hour group sessions, using a "*coping modeling problem solving approach*", to train parents on effective parenting styles and strategies, plus homework.			✓		✓						8<br>Weekly<br>Over 2 months	Other professionals
**Santelices 2011**	6 two-hour group sessions during pregnancy on "*maternal sensitivity...and to promote the development of a secure and healthy bond between mother and child*", and 4 hour-long one-to-one sessions postpartum to observe interactions and give feedback	✓	✓	✓								10<br>Variable<br>Over 16 months	Other professionals
**Tsiantis 2000**	Primary health care workers trained to use a semi-structured interview technique during 6-8 routine visits to discuss age-appropriate child development topics.		✓									6-8<br>Variable<br>Over 36 months	Health professionals
**Wiggins 2004 Intervention 1**	Supportive home visits conducted postnatally by five very experienced health visitors, adapted to each woman's needs to address her concerns and questions	✓										12<br>Monthly<br>Over 12 months	Health professionals
High intensity													
**Cupples 2011**	Trained peer mentors provided one-to-one support on "health-related" topics via home visit or phone call	✓							✓			22<br>Every 2-4 weeks<br>Over 17 months	Peer mentors
**Drotar 2008**	One-to-one home visits; monthly parent group sessions; annual developmental and health screening; access to resource network	✓		✓			✓			✓		27<br>Every 2-4 weeks<br>Over 36 months	Other professionals
**Griffith 2011**	12 two-hour group sessions including watching videos, group discussions and role play, to help understand and manage child behaviour; homework tasks to complete.			✓		✓						12<br>Weekly<br>Over 3 months	Mix of health and other professionals
**Miller 2015**	30-60 minute monthly home visit by a Lifestart family visitor, and a monthly magazine ("Growing Child") of age-appropriate activities	✓			✓							35<br>Monthly<br>Over 36 months	Other professionals
**Minkovitz 2003**	Enhanced well-child care (12 visits, including access to Reach Out and Read literacy programme); 6 home visits in 3 years; telephone line; developmental screening; written guidance; monthly parent groups; links to community resources	✓	✓	✓	✓		✓		✓	✓		16<br>Variable<br>Over 36 months	Mix of health and other professionals
**Wagner 2001**	Monthly home visits and parent group meetings to provide information on child development and demonstrate age-appropriate activities. Periodic developmental screening and, if needed, referrals to community services provided.	✓		✓			✓			✓		24<br>Monthly<br>Over 24 months	Other professionals

* 1 = One-to-one home visits, 2 = One-to-one clinic visits, 3 = Group sessions, 4 = Handouts, 5 = Activities to perform at home, 6 = Developmental screening, 7 = Toys and/or books, 8 = Telephone support, 9 = Access to community resources, 10 = Other

For peer review only

For peer review only - http://bmjopen.bmj.com/site/about/guidelines.xhtml

Study	Description	Components*										Contacts: Number Frequency Duration	Who delivered the intervention?
		1	2	3	4	5	6	7	8	9	10		
**2b. Studies comparing two interventions with each other**													
**Beeghly 1995** **1: Low intensity**	3 individual 45 minute sessions where mother observed a Neonatal Behavioral Assessment (NBAS) and discussed findings with a paediatrician (including exploring the care-giving that might promote the mother-child relationship)		✓									3 At 3, 14 and 30 days old	Health professionals
**Beeghly 1995** **2: Low intensity**	3 individual 45 minute sessions, where mother discussed her perceptions of motherhood and concerns with a paediatrician, and was given feedback about an NBAS that was conducted in a different room		✓									3 At 3, 14 and 30 days old	
**Lobo 2012** **1: Moderate intensity**	Parents taught a positioning and handling programme during a home visit by a physiotherapist to be completed for 15 minutes daily for 3 weeks. 6 assessment home visits also completed. Caregivers given manual and a session diary.	✓			✓	✓						6 Every 2 weeks Over 3 months	Intervention delivered by parents after training
**Lobo 2012** **2: Moderate intensity**	Parents asked to engage their child in 15 minutes of face-to-face interaction daily for 3 weeks. This group also had 6 assessment visits.	✓										6 Every 2 weeks Over 3 months	
**Doyle 2011** **1: Moderate intensity**	Access to a support worker; annual packs containing toys and books (worth 100 Euros); facilitated access to one year of preschool; stress control and healthy eating sessions		✓	✓ (2)				✓		✓	✓	Variable Variable Over 36+ months	Other professionals
**Doyle 2011** **2: High intensity**	Home visits from a trained mentor; tip sheets; Triple P Positive Parenting group sessions; baby massage; annual packs containing toys and books (worth 100 Euros); facilitated access to one year of preschool; stress control and healthy eating sessions	✓		✓ (3)	✓			✓		✓	✓	Variable Weekly Over 36+ months	
**Johnson 2006** **Intervention 1: High intensity**	Enhanced well-child care (6 visits, including Reach Out and Read literacy programme); 6 home visits in 3 years; telephone line; developmental screening; written guidance; monthly parent groups; links to community resources	✓	✓	✓	✓		✓		✓	✓		Variable Monthly Over 36+ months	Health professionals
**Johnson 2006** **Intervention 2: High intensity**	As above, plus 3 additional home visits during 2nd half of pregnancy	✓	✓	✓	✓		✓		✓	✓		Variable Monthly Over 36+ months	
**Landry 2006** **Intervention 1: High intensity**	Playing and Learning Strategies: One-to-one home visits of 1.5 hours to discuss the child's current development and behaviour, feedback on videotaped interactions with child; and planning with mothers of how to increase their "*responsive*" behaviours	✓										10 Weekly Over 3 months	Other professionals
**Landry 2006** **Intervention 2: High intensity**	Developmental Assessment Screening: One-to-one home visits of 1.5 hours consisting of developmental screening and discussions on child development. Handouts on common issues (e.g. sleep, feeding) given.	✓			✓		✓					10 Weekly Over 3 months	

* 1 = One-to-one home visits, 2 = One-to-one clinic visits, 3 = Group sessions, 4 = Handouts, 5 = Activities to perform at home, 6 = Developmental screening, 7 = Toys and/or books, 8 = Telephone support, 9 = Access to community resources, 10 = Other

For peer review only

For peer review only - http://bmjopen.bmj.com/site/about/guidelines.xhtml

Table 3: Summary of Findings

**Population:** Parents from antenatal period to 2 years postpartum<br>**Settings:** Universal programmes offered within defined populations<br>**Intervention:** Programmes to improve child development outcomes by enhancing health professional contact<br>**Comparison:** Usual care, or two different interventions compared		
**Number of studies**<br>**Total number of participants**[*]<br>**Total number of comparisons**[†]	**Results**	**Quality of the evidence**
Outcome: Motor Development		
**Comparison:** Intervention with usual care		
3 studies[22 25 56]<br>972 participants, 3 comparisons	**No effect** in the 3 comparisons.<br>**No effect** in the 2 sub-groups examined in 1 study.	MODERATE (downgraded one level because of risk of bias)
**Comparison:** Two interventions		
3 studies[21 27 46]<br>304 participants, 34 comparisons (20 in Doyle, 13 in Lobo)	**No difference** in 25 comparisons; **better outcomes in the more intensive intervention group** in 9 comparisons (all in 1 study).<br>**No difference** in the 2 sub-groups examined in 1 study.	MODERATE (downgraded one level because of risk of bias)
Outcome: Language Development		
**Comparison:** Intervention with usual care		
7 studies[‡ 22 24 33 41 54 56 58]<br>Participant numbers not known[§], 16 comparisons (6 in High)	**No effect** in 10 comparisons; **better outcomes in intervention than control** in 4 comparisons (2 studies); **worse outcomes in intervention than control group** in 2 tests (1 study).<br>Sub-group effects reported in 4 studies, with some better outcomes in intervention than control, but reporting of sub-groups unclear and incomplete.	LOW (downgraded two levels because of risk of bias and inconsistency[††])
**Comparison:** Two interventions		
3 studies[27 44 45]<br>632 participants, 27 comparisons (21 in Doyle)	**No difference between the two interventions** in 23 comparisons; **better outcomes in the more intensive intervention group** in 4 tests (2 studies). No sub-group effects examined.	LOW (downgraded two levels because of risk of bias and inconsistency[††])
Outcome: Cognitive Development		
**Comparison:** Intervention with usual care		
5 studies[22 25 33 47 56]<br>1729 participants, 20 comparisons (16 in Drotar)	**No effect** in 18 comparisons; **better outcomes in intervention than control** in 2 comparisons (2 studies). Sub-group effects examined in 2 studies, with some better outcomes in intervention than controls seen, but reporting of sub-groups unclear and incomplete.	LOW (downgraded two levels because of risk of bias and inconsistency[††])
**Comparison:** Two interventions		
3 studies[21 27 45]<br>516 participants, 20 comparisons (18 in Doyle)	**No difference between the two interventions** in 16 comparisons; **better outcomes in the more intensive intervention group** in 4 comparisons (2 studies).<br>**No significant interaction** in the 4 tests performed in 1 study.	LOW (downgraded two levels because of risk of bias and inconsistency[††])

For peer review only - http://bmjopen.bmj.com/site/about/guidelines.xhtml

Outcome: Social and emotional wellbeing		
**Comparison:** Intervention with usual care		
15 studies[‡ 23-25 33 34 38 43 47 49 51-54 56 58]<br>Participant numbers not known[§], 69 comparisons (7 in Drotar, 14 in Feinberg, 8 in Niccols 2009, 16 in Tsiantis, 5 in Wagner)	**No effect** in 60 comparisons ([**]in 1 study); **better outcomes in intervention than control** in 9 comparisons (2 studies).<br>Sub-group effects examined in 4 studies, with some better outcomes in intervention than control seen, but reporting of sub-groups unclear and incomplete.	LOW (downgraded two levels because of risk of bias and inconsistency[††])
**Comparison:** Two interventions		
3 studies[27 44 45]<br>630 participants, 87 comparisons (78 in Doyle, 7 in Landry)	**No difference between the two interventions** in 82 comparisons; **better outcomes in one intervention compared with another** in 4 comparisons (2 studies); **worse outcome in the more intensive intervention group** in 1 test (in 1 study).<br>Sub-group effects examined in 1 study, with some better outcomes in one intervention compared with another, but reporting of sub-groups unclear and incomplete.	LOW (downgraded two levels because of risk of bias and inconsistency[††])
Outcome: Overall Child Development		
**Comparison:** Intervention with usual care		
3 studies[‡ 22 38 58]<br>1414 participants, 8 comparisons	**No effect** in 7 comparisons; **better outcomes in intervention than control** in 1 comparison (1 study). No sub-groups examined.	MODERATE (downgraded one level because of inconsistency[††])
**Comparison:** Two interventions		
1 study[27]<br>151 participants, 4 comparisons	**No difference between the two interventions** in the 4 comparisons.	MODERATE (downgraded one level because of risk of bias)

[*] As the number of participants can vary within studies (e.g. where outcomes are measured at several different time points), the total number of participants noted here is calculated from the total numbers analysed at the last time point in each study; this is therefore a conservative estimate of the total number of participants for each outcome.
[†] Total number of comparisons performed for the specified outcome in the whole study population across all of the studies; studies in which 5 or more comparisons are conducted on the same outcome (either at one time point or across different time points) are referenced
[‡] Includes both comparisons in Wiggins (supportive health visiting with usual care, and community groups with usual care)
[§] Total participant numbers not reported in Tsiantis
[**] The comparison reported is the ratio or difference in estimates between intervention and control group, or between the two intervention groups, at the specified follow-up point unless otherwise noted ([**] indicates where the difference in change between intervention and control is used instead).
[††] Inconsistency noted where i) positive, negative and no effects are reported for an outcome, and/or ii) there is a different effect seen in more than 30% of comparisons across studies, and/or iii) different effects are reported within a study, and/or iv) most of the positive or negative effects are seen in sub-groups only and the reporting of sub-group effects is incomplete or inconsistent

GRADE Working Group grades of evidence
High quality: Further research is very unlikely to change our confidence in the summary of the effects.
Moderate quality: Further research is likely to have an important impact on our confidence in the summary of the effects.
Low quality: Further research is very likely to have an important impact on our confidence in the summary of the effects.
Very low quality: We are very uncertain about the summary of the effects.

For peer review only - http://bmjopen.bmj.com/site/about/guidelines.xhtml

Table 4: Effect direction plot, ordered by risk of bias and intensity of intervention (key given in footnote)

Study<br>Intervention intensity	Study design	Risk of bias	Adherence	Outcome measurement		Development outcomes					Additional detail on intervention effects or sub-group analyses
				Timing	When?	Motor	Lang	Cogn	SEWB	Overall	
**4a. Studies comparing one intervention with usual care**											
**Hiscock 2008**<br>Low	cRCT	Low	49% of parents attended all sessions	Post	Short				○		
				Post	Medium				○		
				Post	Long				○		
**Wiggins 2004**<br>Low (CGS)	iRCT	Low	19% of women attended a group	Post	Immed					○	
				Post	Short		○		○	○	
**Cheng 2007**<br>Moderate	iRCT	Low	Not reported	Post	Short						For the short-term analysis, only sub-group analyses (by attachment quality) presented; results inconsistent.
				Post	Long				○		
**Wiggins 2004**<br>Moderate (SHV)	iRCT	Low	Mean number of visits = 7 (of 12 planned)	Post	Immed					○	1 of 1 comparison showed improved language outcome and 1 of 1 comparison showed improved overall development in intervention group.
				Post	Short		●		○	●	
**Cupples 2011**<br>High	iRCT	Low	Mean number of contacts = 8.5 (of 22 planned)	Post	Immed	○		○	○		
**Griffith 2011**<br>High	iRCT	Low	60% attended 8 or more sessions (of 12 planned)	Post	Short				○	○	
**Miller 2015**<br>High	iRCT	Low	Adherence data currently being analysed	During	Long			○	○		
**High 2000**<br>Low	iRCT	High	Mean number of visits = 3.4 (of 5 planned)	Post	Short		○				3 of 6 comparisons showed improved language outcomes in intervention group.<br>Sub-group results: no differences seen in 13-17 month olds; 6 of 6 comparisons in 18-25 month olds showed improved language outcomes in intervention group; no test for interaction presented.
**Chang 2015**<br>Low	cRCT	Unclear	83% of mothers attended all visits	Post	Short	○	○	○		○	Improved cognitive outcome in intervention group on adjusting for potential confounders.
**Christakis 2007**<br>Low	iRCT	Unclear	Not reported	Post	Short		○		○		Sub-group results: 2 of 3 comparisons in low income group showed improved SEWB outcomes in intervention group; test for interaction not presented
**Feinberg 2008**<br>Moderate	iRCT	Unclear	80% attended at least 3 of 4 antenatal sessions<br>60% attended at least 3 of 4 postnatal sessions	Post	Short				○		3 of 5 comparisons in short term and 1 of 2 comparisons in medium term showed improved SEWB outcomes in intervention group.<br>Results presented in text suggest there may be interaction effects with gender, but there is incomplete reporting of the sub-group analyses.
				Post	Medium				○		
				Post	Long				○		
**Niccols 2008**<br>Moderate	iRCT	Unclear	58% attended 4 or more sessions (of 8 planned)	Post	Immed				○		
				Post	Short				○		

For peer review only - http://bmjopen.bmj.com/site/about/guidelines.xhtml

**Niccols 2009**<br>Moderate	iRCT	Unclear	Not reported	Post<br>Post	Immed<br>Short				◦<br>•		2 of 4 comparisons immediately post-intervention term showed improved SEWB outcomes in intervention group.<br>3 of 4 comparisons showed improved SEWB outcomes in intervention group in short-term.
**Santelices 2011**<br>Moderate	iRCT	Unclear	Not reported	Post	Short				◦		
**Tsiantis 2000**<br>Moderate	cRCT	Unclear	Not reported	Post<br>Post<br>Post	Short<br>Medium<br>Long		 <br>ø		○<br>○<br>○		2 of 2 comparisons showed poorer language outcomes in the intervention arm in the long-term.<br>1 of 2 comparisons showed poorer SEWB outcomes in the intervention arm in the medium-term.<br>1 of 8 comparisons showed improved SEWB outcomes in the intervention arm in the long-term.<br>Incomplete reporting of the sub-group analyses.
**Drotar 2008**<br>High	iRCT	Unclear	Not reported	During<br>During<br>During	Short<br>Medium<br>Long		 <br>○	 ○<br>○	○<br>○<br>○		1 of 12 comparisons showed improved SEWB outcomes in the intervention arm in the long-term.<br>Incomplete reporting of the sub-group analyses.
**Minkovitz 2003**<br>High	iRCT	Unclear	79% of parents received 4 or more services (of 16)	During	Long				○		
**Wagner 2001**<br>High	iRCT	Unclear	44% of families still receiving services at 2 yrs	During	Long	○	○	○	○		Results also stratified by income; no significant interactions reported.
**4b. Studies comparing two interventions**											
**Beeghly 1995**<br>2 low intensity	iRCT	Unclear	Not reported	Post	Short	○		○			Tested for interaction between intervention and parity, IUGR, “demographic” risk and maternal psychological risk; no significant interactions found.
**Lobo 2012**<br>2 moderate intensity	iRCT	Unclear	Excluded individuals who did not perform intervention on at least 60% of expected days	During<br>Post	Short<br>Short	◦<br>•					4 of 8 comparisons showed improved motor outcomes in the intervention arm in the short-term whilst intervention was ongoing.<br>5 of 5 comparisons showed improved motor outcomes in the intervention arm post-intervention.
**Doyle 2011**<br>1 high & 1 moderate	iRCT	Unclear	High: Mean number of visits = 46<br>Moderate: Not reported	During<br>During<br>During	Short<br>Medium<br>Long	○<br>○<br>○	○<br>○<br>○	○<br>○<br>○	○<br>○<br>○	○<br>○<br>○	3 of 12 comparisons showed improved cognitive outcomes, and 1 of 62 comparisons showed improved SEWB outcomes in the intervention arm, in the long-term whilst intervention was ongoing.
**Johnston 2006**<br>2 high intensity	iRCT	Unclear	Not reported	During	Long		○		○		2 of 4 comparisons showed improved language outcomes, and 1 of 3 comparisons showed poorer SEWB outcomes, in the intervention arm in the long-term whilst intervention was ongoing.
**Landry 2006**<br>2 high intensity	iRCT	Unclear	91% of parents completed all 10 visits plus 2 assessment visits	Post	Short		●	●	○		2 of 2 comparisons showed improved language outcomes, and 1 of 1 comparison showed improved cognatic outcome in the intervention arm.<br>3 of 6 comparisons showed improved SEWB outcomes in the intervention arm.<br>Interaction with birthweight examined, but reporting of results incomplete.

For peer review only - http://bmjopen.bmj.com/site/about/guidelines.xhtml

Key: ● = outcome reported, statistically significant differences in favour of intervention found in 70% or more of comparisons within a study; Ø = outcome reported, statistically significant differences in favour of control found in 70% or more of comparisons; ○ = outcome reported, no statistically significant differences found, or found in <70% of comparisons; ◊ = outcome reported, inconsistent results (defined as in Table 3); [blank box] = outcome not reported

Size of the symbol indicates the total sample size included in analysis: ●Ø○◊ = >500; ●Ø○◊ = 100-500; ●Ø○◊ = <100

Abbreviations: CGS = community groups; SHV = supportive health visiting; cRCT = cluster randomised controlled trial; iRCT = individually-randomised controlled trial; Immed = immediate; Lang = language; Cogn = cognitive; SEWB = social and emotional wellbeing

Outcome measurement i) Timing: During = whilst intervention is ongoing; Post = after intervention is completed

ii) When?: Immediate = <1 month; Short = 1-6 months; Medium = >6-12 months; Long = >12 months

For peer review only

Figure 1: PRISMA flow diagram

Footnote to Figure 1

Reason for exclusion at full text screening:
Ongoing study, n = 3
Quasi-experimental (control group but no randomisation), n = 10
Pre-post test comparison only, n = 5
Not a primary study (reviews, editorials, programme descriptions), n = 67
Not conducted in a high income country, n = 3
Intervention delivered in childcare settings, n = 14
Targeted programme (child factors), n = 37
Targeted programme (adult or family risk factors), n = 88
Mean age of children at intervention >24 months, n = 53
Mean age of children at outcome > 36 months, n = 5
No child development outcomes, n = 17

Figure 2: Risk of bias graph: review authors' judgements about each risk of bias item presented as percentages across all included studies

Figure 3: Risk of bias summary: Review authors' judgements about each risk of bias item for each included study

For peer review only - http://bmjopen.bmj.com/site/about/guidelines.xhtml

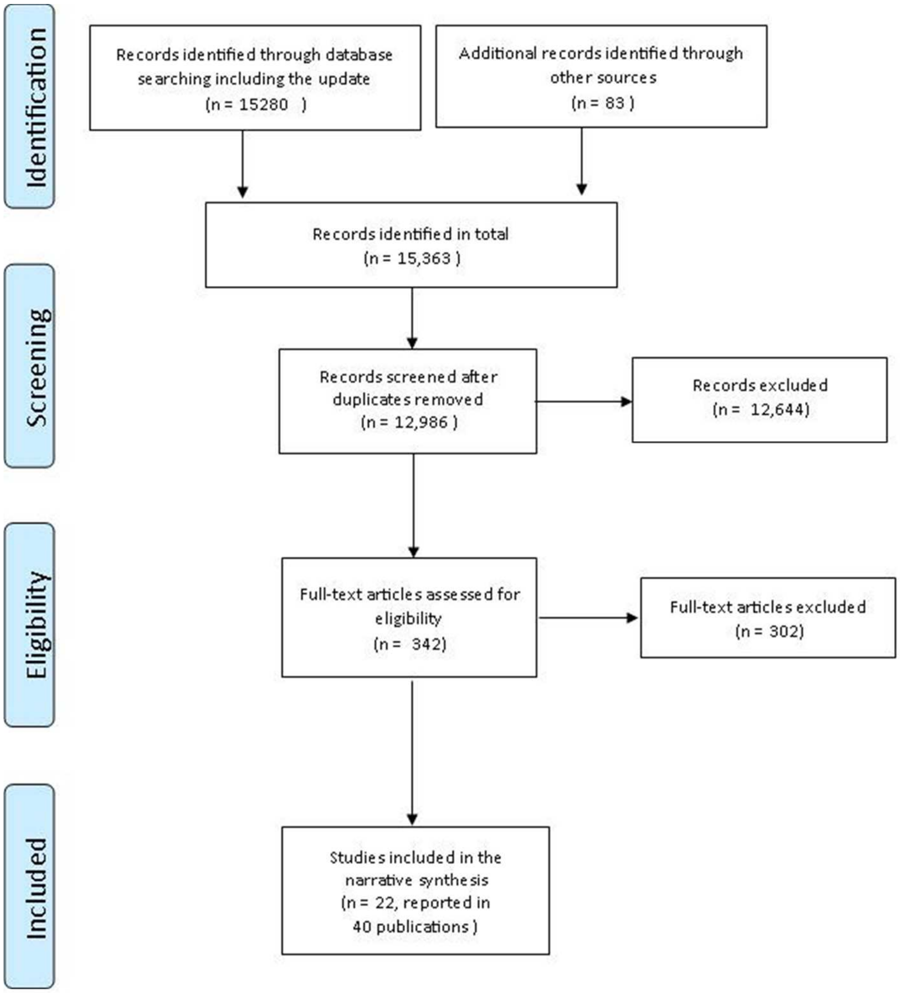

PRISMA flow diagram

173x224mm (96 x 96 DPI)

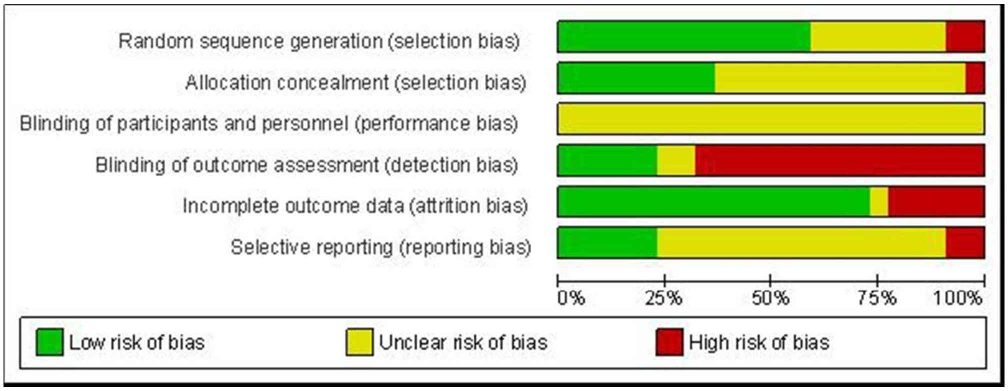

Risk of bias graph: review authors' judgements about each risk of bias item presented as percentages across all included studies

160x61mm (96 x 96 DPI)

For peer review only

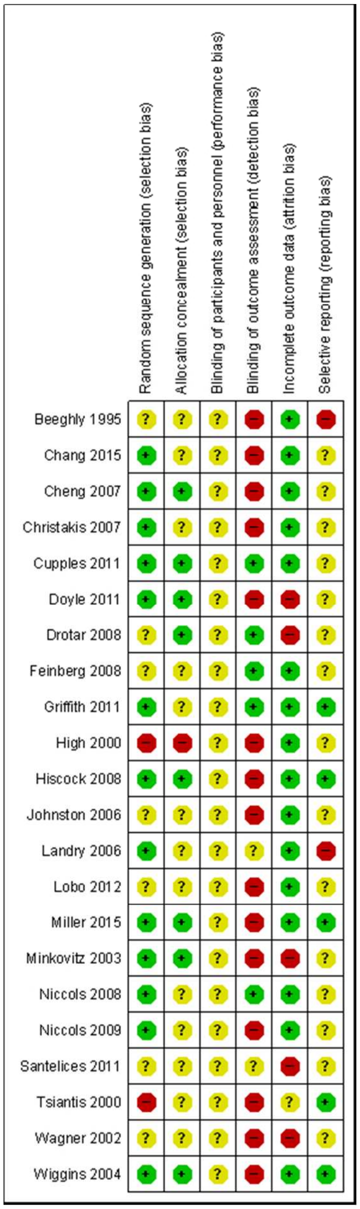

Risk of bias summary: Review authors' judgements about each risk of bias item for each included study

78x265mm (96 x 96 DPI)

Web Appendices

A. Medline search strategy

B. List of programme and organisation websites and journals for electronic journal of contents (eTOC) search

C. Full narrative summary of results

D. Effect direction plots, stratified by other characteristics

For peer review only

Web Appendix A. Medline search strategy

Database: Ovid MEDLINE(R) without Revisions
Search Strategy:
----
1 exp Health Personnel/
2 exp Nurses, Community Health/
3 health professional.tw.
4 exp Physician-Patient Relations/
5 (nurse* or physician* or doctor* or midwife* or midwive* or health visitor*).tw.
6 ((health* or medical or nurs* or hospital) adj (personnel or provider* or professional* or practitioner* or worker* or staff or specialist*)).tw.
7 (general practitioner* or gp or gps or clinician* or hospitalist* or psychologist* or social worker* or welfare worker*).tw.
8 ("Mothers aide" or "mothers help" or "maternity nurse" or "public health nurs*" or "specialist public health nurse" or "nursery nurse" or nanny or "early year educator*" or childcarer).tw.
9 ((paediatric or pediatric) adj clinic*).tw.
10 ((baby or child) adj clinic*).tw.
11 Primary Health Care/
12 exp House Calls/
13 home visit*.tw.
14 day care.mp.
15 clinic visit.tw.
16 surgery visit.tw.
17 clinician-patient interaction.tw.
18 early intervention*.tw.
19 ((parent* or mother*1 or father*1 or mum or dad or family or families) adj (group or support or training or education)).tw.
20 ((carer* or caregiver) adj (group or support or training or education)).tw.
21 ("early years staff" or "early years health professional*" or "early years professional*" or "early years educator*" or "early years practitioner*").tw.
22 (preschool educator or nursery staff).tw
23 parent educator*.tw.
24 (Family support or family support worker or support worker).tw.
25 (Community health worker or lay health worker).tw
26 Public health staff.tw.
27 Breakthrough to Literacy.mp
28 Family Foundations.mp.
29 Incredible Years.mp.
30 Parent Effectiveness Training.mp.
31 Primary Care Triple P.mp.
32 Triple P.mp.
33 Born to Learn.mp.
34 Mellow Parenting.mp.
35 "The Family Partnership Model".mp.
36 The Solihull Approach.mp.
37 "Parent child interaction therapy".mp.
38 "Nurturing Programme".mp.
39 "The Family Partnership Model".mp.
40 "Peers Early Education Partnership".mp.
41 "Webster Stratton Incredible Years".mp.

For peer review only

42 "Community Mothers Program".mp.
43 "Social Support and Family Health".mp.
44 "Limerick Lullaby".mp.
45 or/1-44 (ENHANCED PROFESSIONAL CONTACT TERMS)
46 exp Child Development/
47 exp language development/
48 Object Attachment/
49 Concept Formation/
50 Child Behavior Disorders/
51 ((child or infant) adj development).tw.
52 ((education* or cognitive or mental or language or physical or motor or social or emotional) adj3 (progress or development)).tw.
53 ((physical or emotional or social) adj (wellbeing or health)).tw.
54 (faltering growth or growth faltering or stunting).tw.
55 early child development.tw.
56 ((vocabulary or speech or language) adj development).tw.
57 Child behavio?r.tw.
58 Language/
59 Object Attachment/
60 social competence.tw.
61 social interaction.tw.
62 school readiness.tw.
63 or/46-62 (CHILD DEVELOPMENT TERMS)
64 45 and 63

For peer review only

Web Appendix B. List of programme and organisation websites and journals for electronic journal of contents (eTOC) search

Table B1. List of relevant programmes and organisations for which websites were searched

**Programme name**
1. Al's Pals
2. Breakthrough to Literacy
3. Bright Beginnings
4. Circle of Security
5. Community Mothers Program
6. Curiosity Corner
7. Developing Everyone's Learning and Thinking Abilities (DELTA)
8. Eager and Able to Learn
9. Family Check-Up
10. Family Foundations
11. Family Literacy programme
12. Family Nurturing Network: Family Connections
13. Family Partnership Model
14. Fun and Families
15. HIPPY
16. Holding Hands
17. I Can Problem Solve
18. Incredible Years
19. Infant Behavioral Assessment and Intervention Programme
20. Let's Begin with the Letter People
21. Let's Play in Tandem
22. LifeStart ("Growing Child")
23. Limerick Lullaby
24. Living with Children
25. Mellow Parenting
26. Minding the Baby
27. Noughts to Sixes (From Pram to Primary School), Family Caring Trust
28. Nurturing Programme
29. Parents as First Teachers or Parents as Teachers (Born to Learn)
30. Parent–child interaction therapy
31. Parent Effectiveness Training
32. Parenting Positively
33. Parents Plus Early Years
34. Peers Early Education Partnership
35. Preparing for Life<br>Child Development Initiative
36. Ready, Set, Leap!
37. Ready, Steady, Grow
38. Rock-a- Bye
39. Sheffield Raising Early Achievement in Literacy (REAL) project
40. Solihull Approach

For peer review only

41. Social Support and Family Health
42. Strengthening Families, Strengthening Communities
43. Supporting Parents on Kids Education (SPOKES)
**Organisation name**
1. Basic Skills Agency
2. Department of Education, UK
3. Early Childhood Australia
4. Education Endowment Fund
5. Early Intervention Foundation
6. European Commission (Education and Training)
7. European Early Childhood Education Research Association
8. Harvard Developing Child Programme
9. National Health Service (NHS)
10. National Institute for Health and Care Excellence (NICE)
11. National Institute of Health (USA)
12. Raising Children Network (Australia)
13. Sure Start/Flying Start
14. World Bank Early Child Development
15. World Health Organisation

For peer review only

Table B2. Journals included in electronic table of contents (eTOC) search

Archives of Diseases in Childhood

Child: Care, Health and Development

Child Development

Early Child Development and Care

Early Childhood Research Quarterly

Infant Mental Health Journal

Journal of Early Childhood Research

Pediatrics

For peer review only

Web Appendix C. Full narrative summary of results

Table C1: Motor Development Outcomes

Motor development outcomes		
**Studies comparing one intervention with usual care**		
**Study**	**Measure**	**Results**
Chang 2015 (Short term post intervention)	Griffith Mental Development Scale	**No difference in mean hand/eye coordination score** between intervention and control (mean 95.95, SD 10.25 compared with mean 94.62, SD 9.89); adjusted mean difference 0.73, 95% CI -0.83, 2.29; p value for comparison = 0.89)
Cupples 2011 (Immediately post intervention)	Bayley Scales of Infant Development (Psychomotor Development Index)	**No difference in mean scores** between intervention and control for PDI (MD [non-imputed] 1.64, 95% CI -0.94-4.21, p=0.21)
Wagner 2001 (Long term whilst programme ongoing)	Development Profile II	**No difference in mean physical development scores** between intervention and control in total sample (mean in intervention 2.9, mean in control 3.0, p>=0.05), or in samples stratified by income level (p>=0.05 for all tests)
**Motor development outcomes**		
**Studies comparing two interventions**		
**Study**	**Measure**	**Results**
Beeghly 1995 (Short term post-intervention)	Bayley Scales of Infant Development (Psychomotor Development Index)	**No difference in mean scores** for PDI in infant-centred (mean 109.77, SD 13.05) and mother-centred (mean 108.31, SD 12.43) groups at 3 months post-intervention (when children were 4 months, p>=0.05).
Doyle 2011 (Short term whilst intervention is ongoing)	Ages and Stages Questionnaire	**No differences** between high and low intensity programme using mean scores or cut-offs for 4 comparisons: gross motor scores, gross motor cut-off, fine motor scores, fine motor cut-offs (p values adjusted for multiple testing)
Doyle 2011 (Medium term whilst intervention is ongoing)	Ages and Stages Questionnaire	**No differences** between high and low intensity programme using mean scores or cut-offs for 4 comparisons: gross motor scores, gross motor cut-off, fine motor scores, fine motor cut-offs (p values adjusted for multiple testing)
Doyle 2011 (Long term whilst intervention is ongoing)	Ages and Stages Questionnaire (measured at 18, 24 and 36 months)	**No differences** between high and low intensity programme using mean scores or cut-offs for 4 comparisons at each time point: gross motor scores, gross motor cut-off, fine motor scores, fine motor cut-offs at any of the time points (p values adjusted for multiple testing)
Lobo 2012 (Short term whilst intervention ongoing)	Alberta Infant Motor Scale	**No difference for change in total mean scores** between in handling group compared with social experience group at 2-3 months (scores not given, p>=0.05).<br>**Greater change in mean score** for **prone** subscale in handling group compared with social experience group at 2-3 months of age (Cohen's correlation coefficient **r = -0.45, p<0.01**).<br>**No difference** for **supine** and **sitting** subscale at 2-3 months (scores not given, p>=0.05).<br>**Greater change in total mean score** in handling group compared with social experience group at 5 months of age (Cohen's correlation coefficient r = -0.46, p<0.01).<br>**Greater change in total mean score** for **prone** (r=-0.45, p<=0.01) and **sitting** (r=-0.34, p<=0.05) subscale in

		handling group at 5 months of age.<br>**No difference** for **supine** subscale at 5 months of age (scores not given, p>=0.05).
Lobo 2012<br>(Short term post-intevention)	Parent Milestone Report Form	Intervention group children reached for midlife objects before control children (no data provided, p<=0.05)<br>Intervention group children transferred objects from one hand to another **2.5 weeks earlier** (p<=0.05)<br>Intervention group children crept/crawled **5 weeks earlier** (p<=0.05)<br>Intervention group children walked supported **2.5 weeks earlier** (p<=0.05)<br>Intervention group children walked alone **6 weeks earlier** (p<=0.05)

For peer review only

Table C2: Language Development Outcomes

Language development outcomes		
**Studies comparing one intervention with usual care**		
**Study**	**Measure**	**Results**
Chang 2015 (Short term post intervention)	Griffith Mental Development Scale<br><br>MacArthur Bates Communicative Development Inventory (Short version, CDI)	**No difference in mean language scores** (Griffith scale) between intervention and control (mean 99.78, SD 14.01 compared with mean 99.90, SD 13.58); adjusted mean difference -0.54, 95% CI -2.81, 1.75; p value for comparison = 0.89)<br>**No difference in mean vocabulary scores (CDI)** between intervention and control (mean 38.00, SD 18.42 compared with mean 39.57, SD 20.49); adjusted mean difference -0.94, 95% CI -3.49, 1.61; p value for comparison = 0.89)
Christakis 2007 (Short term post intervention)	MacArthur Communicative Development Inventory	Whole sample: **No difference** between intervention and control (linear regression results: β for intervention variable raw score = 7.52, 95% CI -0.66-15.7; β for intervention variable percentile score = 8.40, 95% CI -3.00-19.90) at approx 4 months post intervention (children were 24 to 36 months).<br>Low and middle income sample: **Higher scores** in the intervention group (linear regression results: β for intervention variable raw score = 12.40, 95% CI 3.0-21.8, p=0.01; β for intervention variable percentile score = 14.94, 95% CI 1.37-27.60, p=0.03) at approx 4 months post intervention (children were 24 to 36 months).<br>High income sample: Results not presented in paper.
Drotar 2008 (Long term whilst intervention ongoing)	Systematic Analysis of Language Transcripts (SALT)	**No difference in mean scores** between intervention and control when children were 36 months (MD -3.91, 95% CI -14.32-6.77, p=0.48).
High 2000 (Short term post-intervention)	Modified MacArthur Communicative Development Inventory (short form)	**Higher mean scores** in intervention group for receptive vocabulary in total sample (p values between 0.008 and 0.003 in 3 tests), but no difference on expressive vocabulary (3 tests)<br>**No difference** in mean scores in receptive and expressive vocabulary between intervention and control groups in children aged 13-17 months at assessment (6 sub-group tests).<br>**Higher mean scores** in intervention group for receptive and expressive vocabulary in children aged 18-25 months at assessment (**p values between 0.01 and 0.003 in 6 sub-group tests**)
Tsiantis 2000 (Immediately post-intervention)	Bzoch-league Receptive Expressive Emergent Language Scale (REEL)	**Better performance in observed language** in comparison group children (no scores presented, but **p<0.0001**)<br>Results analysed by country, but not presented in paper.
Wagner 2002 (Long term whilst intervention is ongoing)	Development Profile II (DPII)	**No difference in mean scores** between intervention and control in communication development scores in whole sample (mean in intervention 4.9, mean in control 4.5, p>=0.05), or when stratified by income group.
Wiggins 2004 (Short term post-intervention)	Question on whether the mother has any worries about their child's speech	**Fewer mothers in the SHV group** expressed a worry about their child's speech than in the control group (risk ratio 0.46, 95% CI 0.23, 0.93)<br>**No difference** in the number of number of mothers expressing worries about speech between CGS and control (risk ratio 1.22, 95% CI 0.78, 1.92)

For peer review only

Language development outcomes		
**Studies comparing two interventions**		
**Study**	**Measure**	**Results**
Doyle 2011<br>(Short term whilst intervention is ongoing)	Ages and Stages Questionnaire	**No differences** between high and low intensity programme using mean scores or cut-offs for 2 comparisons: communication scores, communication cut-off (p values adjusted for multiple testing)
Doyle 2011<br>(Medium term whilst intervention is ongoing)	Ages and Stages Questionnaire<br><br>MacArthur-Bates Communicative Development Inventory	**No differences** between high and low intensity programme using mean scores or cut-offs for 2 comparisons: communication scores, communication cut-off (p values adjusted for multiple testing).<br>**No differences** between high and low intensity programme using mean scores for 4 sub-scores: first signs of understanding, words understood, words produced, first communicative gestures (p values adjusted for multiple testing)
Doyle 2011<br>(Long term whilst intervention is ongoing)	Ages and Stages Questionnaire (measured at 18, 24 and 36 months)<br>MacArthur-Bates Communicative Development Inventory (measured at 18 and 24 months)	**No differences** between high and low intensity programme using mean scores or cut-offs for 2 comparisons at any of the time points: communication scores, communication cut-off (p values adjusted for multiple testing).<br>**No differences** between high and low intensity programme using mean scores for 5 sub-scores at 18 months: first signs of understanding, can combine words, first communicative gestures, words produced, words understood, and 2 sub-scores at 24 months: can combine words, words produced (p values adjusted for multiple testing)
Johnston 2006<br>(Long term post-intervention)	Sub-scales of MacArthur Communicative Development Inventory	**Higher mean vocabulary score** in HS + PP group than HS only group at 24 months (aMD 4.08; 95% CI 0.69, 7.47; $p<0.05$).<br>**Higher percentage** combining >=2 words in HS + PP group than HS only group (rate ratio 1.05; 95% CI 1.02, 1.08; $p<0.05$).<br>**No difference** in length of child's longest phrase or variety of word endings between HS+PP and HS only group.
Landry 2006<br>(Short term post-intervention)	Coding of videotaped interactions between child and mother, and child with examiner	**Higher word usage scores** observed in the PALS group than the DAS group in interactions with mother (p=0.02) and examiner (p=0.02) at 3 months post-intervention when child was 13 months.

For peer review only

Table C3: Cognitive Development Outcomes

Cognitive development outcomes		
**Studies comparing one intervention with usual care**		
**Study**	**Measure**	**Results**
Chang 2015 (Short term post intervention)	Griffith Mental Development Scale	No effect in intervention group in unadjusted results (mean 92.69, SD 11.65 compared with mean 89.52, SD 10.56); unadjusted mean difference 3.12, 95% CI 0.86, 5.39; p value for comparison = 0.07); **higher mean cognitive scores (Griffith scale)** for adjusted mean difference (aMD 3.09, 95% CI 1.31, 4.87, p value = 0.007)
Cupples 2011 (Immediately post-intervention)	Bayley Scales of Infant Development (Mental Development Index, MDI)	**No difference in mean scores** between intervention and control when children were 12 months old (MD [non-imputed] -0.81, 95% CI -2.81-1.16, p=0.42)
Drotar 2008 (Short-medium term whilst intervention ongoing)	Bayley Scales of Infant Development (Mental Development Index, MDI)<br>Assessor rating of mastery motivation (persistence, pleasure and competence with a play task)	**No difference in mean scores** between intervention and control when children were 18 months (MD 0.68, 95% CI -1.15-2.76, p=0.42).<br>**No difference in mean scores** between intervention and control for any of the 3 tasks when children were 18 months.
Drotar 2008 (Long term whilst intervention ongoing)	Bayley Scales of Infant Development (Mental Development Index, MDI)<br><br>Assessor rating of mastery motivation (persistence, pleasure and competence with a play task)<br><br>Kaufman Assessment Battery<br><br>Bracken Basic Concept Scale<br><br>Test of Early Reading Ability-2 (TERA-2)	**No difference in mean scores** between intervention and control for MDI (MD -0.78, 95% CI -3.63-2.32, p=0.67) when children were 24 months. **Higher mean score** in intervention than control in low SES sub-group (p for interaction < 0.003, mean for intervention 89.67 (SD16.20) & mean for control 82.16 (SD 17.24, p < 0.01).<br>**No difference in mean scores** between intervention and control for any of the 3 tasks when children were 24 months. **Higher mean score** for task competence in intervention group for low SES sub-group (p for interaction not shown, mean for intervention 584.49 (SD 37.97) & mean for control 556.21 (SD 57.18), p < 0.02.<br>**No difference** between intervention and control in 2 of 3 tests of mastery motivation (persistence and pleasure) when children were 36 months; **higher mean scores** in intervention group for task competence (MD 6.24, 95% CI 0.16-14.76, p=0.05).<br>**No difference in mean scores** between intervention and control for sequential processing, simultaneous processing or mental processing when children were 36 months (e.g. for mental processing MD 0.23, 95% CI -3.33-2.91, p=0.89).<br>**No difference in mean scores** between intervention and control when children were 36 months (MD 1.24, 95% CI -2.24-4.47, p=0.51).<br>**No difference in mean scores** between intervention and control when children were 36 months (MD 0.54, 95% CI -1.70-2.47, p=0.72).
Miller 2015 (Long term whilst intervention ongoing)	British Ability Scale (BASII)	**No difference in mean scores** between intervention and control on cognitive development (mean in intervention group -0.046 (SD 1.007) and mean in control group 0.018 (SD 0.994), Hedges g effect size -0.634, 95% CI -0.277, 0.149, p=0.557)
Wagner 2001 (Long term whilst intervention ongoing)	Development Profile II (DPII)	**No difference in mean scores** between intervention and control in cognitive development scores (mean in intervention 2.2, mean in control 2.4, p>=0.05), or in samples stratified by income.

For peer review only

Cognitive development outcomes		
**Studies comparing two interventions**		
**Study**	**Measure**	**Results**
Beeghly 1995 (Short term post intervention)	Bayley Scales of Infant Development (Mental Development Index, MDI)	**No difference in mean scores** in infant-centred (mean 109.38, SD 12.88) or mother-centred (mean 107.83, SD 10.69) groups at 3 months post-intervention (when children were 4 months, p>=0.05).
Doyle 2011 (Short term whilst intervention is ongoing)	Ages and Stages Questionnaire	**No differences** between high and low intensity programme using mean scores or cut-offs for 2 comparisons: problem solving score, problem solving cut-off (p values adjusted for multiple testing)
Doyle 2011 (Medium term whilst intervention is ongoing)	Ages and Stages Questionnaire<br><br>Development Profile 3	**No differences** between high and low intensity programme using mean scores or cut-offs (2 comparisons): problem solving score, problem solving cut-off (p values adjusted for multiple testing).<br>**No difference** in mean scores or for the proportion above the average cut-off (2 comparisons, p values not adjusted for multiple testing).
Doyle 2011 (Long term whilst intervention is ongoing)	Ages and Stages Questionnaire (measured at 18, 24 and 36 months)<br>Development Profile 3 (measured at 18, 24 and 36 months)	**No differences** between high and low intensity programme using mean scores or cut-offs for 2 comparisons at any of the time points: problem solving score, problem solving cut-off (p values adjusted for multiple testing).<br>At 18 months, **no difference** in mean scores or for the proportion above the average cut-off (p values not adjusted for multiple testing).<br>At 24 months, **no difference for the proportion above the average cut-off** (p values **not** adjusted for multiple testing); **higher mean standardised score** in high intensity group (mean 116.51 [SD 13.30] vs mean 112.57 [S 16.12], p<0.05, **not** adjusted for multiple testing).<br>At 36 months, **higher mean standardised score** in high intensity group (mean 114.66 [SD 14.39] vs mean 109.82 [SD 14.02], p<0.05, **not** adjusted for multiple testing) and **higher proportion above average cut-off** (0.53 [SD0.50] vs 0.36 [SD 0.48], p<0.05, **not** adjusted for multiple testing)
Landry 2006 (Short term post-intervention)	Coding of videotaped interactions between child and mother, and child with examiner	**Higher scores** for independent goal-directed play in the PALS group than in the DAS group (p=0.04) at 3 months post-intervention when child was 13 months.

For peer review only

Table C4: Social and Emotional Wellbeing Outcomes

Social and emotional wellbeing outcomes		
**Studies comparing one intervention with usual care**		
**Study**	**Measure**	**Results**
Cheng 2007 (Short term post-intervention)	Parent-Infant Relationship Global Assessment Scale	Of 37 mother-infant pairs with a "disturbed" relationship at baseline, a **higher percentage** of intervention group was classified as having an "adapted" relationship (63% [12 pairs] vs 28% [5 pairs], p=0.039) at 1 month post-intervention (when children were 10 months).<br>Of 48 mother-infant pairs with an "adapted" relationship at baseline, **no difference in percentage** of intervention vs control classified as "disturbed" (26% [7 pairs] vs 29% [8 pairs], p=0.821) at 1 month post-intervention (when children were 10 months).
Cheng 2007 (Long term post-intervention)	Child Behaviour Checklist	**No difference** between intervention and control in the odds of being in the upper quartile of the CBCL scores (total score aOR 0.40, 95% CI 0.12-1.43; internalizing score aOR0.60, 95% CI 0.23-1.68; externalizing score aOR 0.50, 95% CI 0.19-1.54) at 15 months post-intervention (when children were 24 months).
Christakis 2007 (Short term post-intervention)	Hyperactivity sub-domain of the Child Behavior Checklist	**No difference** in attention problems (score in 90th percentile or higher) between intervention and control in the whole sample (aOR 0.49, 95% CI 0.13-1.81) or low and middle income sample (aOR 0.48, 95% CI 0.12, 1.83) at approx 4 months post intervention (children were 24 to 36 months).
Cupples 2011 (Immediately post-intervention)	Bayley Scales of Infant Development (BSID-II)	**No difference in the mean scores** between intervention and comparison group (MD [non-imputed] -0.88, 95% CI -4.59-2.81, p=0.64)<br>**No difference in the mean scores** between intervention and control for the 3 sub-scores of the BSID-II behavioural scale.
Drotar 2008 (Short-medium term whilst intervention ongoing)	Bayley Behaviour Rating Scale	**No difference in mean scores** between intervention and control when children were 12 months (MD -0.33, 95% CI -1.95-1.64, p=0.87).
Drotar 2008 (Medium-long term whilst intervention ongoing)	Q-Sort measure of Security of Attachment	**No difference** between intervention and control when children were 18 months (no numerical data presented).
Drotar 2008 (Long term whilst intervention ongoing)	Bayley Behaviour Rating Scale<br><br>Child Behaviour Rating Scale<br>Social Skills Rating System	**No difference in mean scores** between intervention and control when children were 24 months (MD 0.43, 95% CI -1.72-3.15, p=0.56) or 36 months (MD -1.64, 95% CI -3.99-0.83, p=0.20).<br>**No difference** between intervention and control when children were 24 months (no numerical data presented).<br>**No difference in mean scores** between intervention and control when children were 36 months for parent. reported social skills (MD 3.33, 95% CI -0.45-5.90, p=0.09) or teacher reported social skills (MD 2.07, 95% CI -3.19-7.15, p=0.45).
Feinberg 2008 (Immediately post-intervention)	Infant Behaviour Questionnaire	**No difference** in parent-child dysfunctional interaction (beta calculated using linear regression) in intervention versus control group according to mother's report (beta -0.137, Cohen's d 0.34, p<0.10), but **intervention fathers had lower dysfunctional interaction scores** (beta = -0.291, Cohen's d = 0.70, p<0.05).<br>**No difference** in infant soothability score (beta calculated using linear regression) in intervention versus control

For peer review only

		group according to mother's report (beta 0.021, Cohen's d not calculated, p>=0.05), but **intervention children were easier to soothe** (had a higher score than control) according to father's reports (beta = 0.312, Cohen's d = 0.35, p<0.05).<br>Score for duration of attention **higher in intervention versus control infants** according to mother's report (F=4.33, Cohen's d = 0.34, p<0.05). Fathers reports of this variable not given in paper.
Feinberg 2008 (Short term post-intervention)	Observer coding of videotaped behaviours (self-soothing and sustained attention at play)	**More self-soothing behaviours** observed in intervention versus control group when infants were 12 months of age (6 months post-intervention, beta = 0.30, Cohen's d = 0.46, p<0.05).<br>**No difference** in sustained attention noted by coders between intervention and control children (beta = 0.05, Cohen's d = 0.08, p>=0.05).
Feinberg 2009 (Short term post-intervention)	Child Behaviour Checklist	Mean scores and SDs for each of the 7 comparisons presented but no accompanying P values; t tests conducted in Stata (assuming 81% follow-up in each group) indicate **no significant differences**.<br>Written results suggest that the model based estimates of intervention effects were significant but only when an interaction with child gender was included in the model - the presentation of these results is unclear and there appears to be an inconsistency between the results presented in the table and those in the text.
Griffith 2011 (Short term post-intervention)	Dyadic Parent-Child Interaction coding system	**No difference in mean scores** between intervention and control for child deviance behaviours in the parent-child interaction observations (negative physical behaviours, destructive behaviours, small talk; mean in intervention 3.18 (SD 3.40) and control 2.97 (SD 3.43), no p value for this comparison) at 3 months or less post-intervention.
Hiscock 2008 (Short term post-intervention)	Child Behaviour Checklist	**No difference in mean scores** between intervention and control for externalising (aMD 0.16, 95% CI -1.01-1.33, p=0.79) or internalising (aMD 0.49, 95% CI -0.20-1.18, p=0.16) behaviours 3 months post-intervention (when children were 18 months).
Hiscock 2008 (Medium term post-intervention)	Child Behaviour Checklist	**No difference in mean scores** between intervention and control for externalising (aMD -0.79, 95% CI -2.27-0.69, p=0.30) or internalising (aMD 0.19, 95% CI -0.77-1.15, p=0.70) behaviours 9 months post-intervention (when children were 24 months).
Hiscock 2008 (Long term post-intervention)	Child Behaviour Checklist	**No difference in mean scores** between intervention and control for externalising (aMD -0.8, 95% CI -2.2-0.6, p=0.26) or internalising (aMD -0.6, 95% CI -1.5-0.3, p=0.19) behaviours 21 months post-intervention (when children were 36 months).
Miller 2015 (Long term whilst intervention is ongoing)	Bayley Social-Emotional and Adaptive Behaviour Questionnaire	**No difference in mean scores** between intervention and control on socio-emotional development (mean in intervention group 0.087 (SD 0.987) and mean in control group -0.108 (SD 1.016, Hedges g effect size 0.195 (95% CI -0.046, 0.437, p=0.114)
Minkovitz 2003 (Long term whilst intervention is ongoing)	Subscales of Child Behaviour Checklist	**No difference in mean scores** for 3 sub-scores (aggressive behaviour, anxious or depressed, sleep problems) between intervention and control group. For example, for aggressive behaviour, the adjusted mean difference between intervention and control was 0.23 (95% CI -0.29, 0.79, p=0.16, higher scores indicate higher reporting of aggressive behaviour)
Niccols 2008 (Immediately post-intervention)	Attachment Q-set (completed by observers)	**No difference in the change in scores between pre-test and post-test** between intervention and comparison group in the intention-to-treat analysis (no numbers presented, text states "no significant differences")
Niccols 2008 (Short term post-intervention)	Attachment Q-set (completed by observers)	**No difference in the change in scores between pre-test and post-test** between intervention and comparison group in the intention-to-treat analysis 6 months post-intervention (no numbers presented, text states "no significant differences")

For peer review only

Niccols 2009 (Immediately post-intervention)	Eyberg Child Behavior Inventory<br><br>Observed parent-child interaction	**No difference in mean scores** between intervention and control for child behaviour problems (mean 48.87 (SD 6.22) vs mean score 50.68 (SD 7.23), p>=0.05)<br>**Higher mean number of observed** positive child behaviours in intervention group (mean 21.10 (SD 8.59) vs mean score 16.27 (SD 9.25), p<0.05), and **higher ratio** of child compliance to maternal requests in intervention group (mean 0.62 (SD 0.19) vs mean score 0.53 (SD 0.19), p<0.05).<br>**No difference in mean number** of observed negative behaviours in intervention versus control (mean 17.81 (SD 8.84) vs mean score 22.29 (SD 8.85), p>=0.05).
Niccols 2009 (Short term post-intervention)	Eyberg Child Behavior Inventory<br><br>Observed parent-child interaction	**Lower mean score** for child behaviour problems in intervention versus control (mean 48.87 (SD 6.22) vs mean score 50.68 (SD 7.23), p<0.017).<br>**Higher mean number** of observed positive child behaviours in intervention group than control (mean 22.61 (SD 8.97) vs mean score 16.69 (SD 8.40), p<0.05), and **higher ratio of** observed compliance to maternal requests in intervention group (mean 0.61 (SD 0.19) vs mean score 0.53 (SD 0.19), p<0.05).<br>**No difference** in mean number of observed negative behaviours between intervention and control (mean 18.86 (SD 7.53) vs mean score 21.29 (SD 7.78), p>=0.05)
Santelices 2010 (Immediately post-intervention)	Observed Strange Situation Procedure	**No difference** between intervention and control in infant attachment security (% classed as "secure" in intervention 72.1%, % classed as "secure" in control 55.2%, p=0.139)
Tsiantis 2000 (Short term whilst intervention is ongoing)	Bayley Scale of Psychosocial Development<br>Observed mother-child interaction	**No difference in mean scores** between intervention and control (mean 7.03 compared with mean 7.04, no numbers in analysis or statistics presented).<br>**Mother-child interaction rated as better** in intervention group in one of the four countries (no numbers in analysis, or scores presented, p for sub-group effect= 0.02).
Tsiantis 2000 (Medium term whilst intervention is ongoing)	Bayley Scale of Psychosocial Development<br>Observed mother-child interaction	**No difference in mean scores** between intervention and control (mean 6.82 compared with mean 7.06, no numbers in analysis or statistics presented)<br>**Mother-child interaction rated as better in comparison group** when all countries' data combined (no numbers in analysis presented, scores presented but not clear what they represent, p = 0.02)
Tsiantis 2000 (Long term whilst intervention is ongoing)	Bates Infant Characteristics Questionnaire	**No difference in total mean scores** in full sample between intervention and control (no numbers in analysis, scores or statistics presented for mean scores); 2 of 7 individual characteristic scores higher in intervention than control in full sample ("sober" p = 0.02, "factor 7" p <0.06).
Wagner 2001 (Long term whilst intervention is ongoing)	Nursing Child Assessment Satellite Training teaching scale (NCAST) used to observe mother-child interactions<br>Measure = Development Profile II (DPII)<br><br>Adaptive Social Behaviour Inventory	**No difference in mean scores** for observed mother-child interaction between intervention and control in total sample (2 comparisons), or when results stratified by income group (p>=0.05)<br><br>**No difference in mean self help or social development scores** between intervention and control in total sample, or when results stratified by income group (p>=0.05)<br>**No difference in mean scores** between intervention and control in total sample, or when results stratified by income group (p>=0.05)
Wiggins 2004 (Short term post-intervention)	Question on whether the mother has any worries about their child's behaviour	**No difference** in the number of number of mothers expressing worries about behaviour between supportive health visiting and control groups (risk ratio 0.74, 95% CI 0.37, 1.47)<br>**No difference** in the number of number of mothers expressing worries about behaviour between community group support and control groups (risk ratio 0.94, 95% CI 0.51, 1.73)

For peer review only

Social and emotional wellbeing outcomes		
**Studies comparing two interventions**		
**Study**	**Measure**	**Results**
Doyle 2011 (Short term whilst intervention is ongoing)	Ages and Stages Questionnaire<br><br>Child Characteristic Questionnaire	**No differences** between high and low intensity programme using mean scores or cut-offs for 4 comparisons: personal social score, personal social cut-off, social-emotional score, social-emotional cut-off (p values adjusted for multiple testing).<br>**No difference** between high and low intensity programme for score based on a 6 point scale of difficult temperament (p value adjusted for multiple testing).
Doyle 2011 (Medium term whilst intervention is ongoing)	Ages and Stages Questionnaire<br><br>Brief Child Toddler Social and Emotional Assessment<br>Temperament and Atypical Behaviour Score<br><br>Child Characteristic Questionnaire	**No differences** between high and low intensity programme using mean scores or cut-offs for 4 comparisons: personal social score, personal social cut-off, social-emotional score, social-emotional cut-off (p values adjusted for multiple testing)<br>**No differences** between high and low intensity programme using mean scores or cut-offs for 4 comparisons: competence score, competence cut-off, problem score, problem cut-off (p values adjusted for multiple testing)<br>**No differences** between high and low intensity programme using mean scores or binary variable (p values **not** adjusted for multiple testing)<br>**No difference** between high and low intensity programme for score based on a 6 point scale of difficult temperament (p value **not** adjusted for multiple testing)
Doyle 2011 (Long term whilst intervention is ongoing)	Ages and Stages Questionnaire (measured at 18, 24 and 36 months)<br><br>Brief Child Toddler Social and Emotional Assessment (measured at 18, 24 and 36 months)<br>Brief Child Toddler Social and Emotional Assessment subdomains (measured at 24 and 36 months)<br>Child Behaviour Checklist (CBCL, measured at 24 and 36 months)<br><br>Child Behaviour Checklist subdomains (CBCL, measured at 24 and 36 months)	**No differences** between high and low intensity programme using mean scores or cut-offs for 4 comparisons: personal social score, personal social cut-off, social-emotional score, social-emotional cut-off (p values adjusted for multiple testing)<br>**No differences** between high and low intensity programme using mean scores or cut-offs for 4 comparisons at 18 and 24 months: competence score, competence cut-off, problem score, problem cut-off, and for 2 comparisons at 36 months: competence score, problem score (p values adjusted for multiple testing)<br>**No differences in mean scores** between high and low intensity programme for 5 comparisons at 24 or 36 months: dysregulation, internal problems, red flag, external problems, autism (p value adjusted for multiple testing).<br>At 24 months, **no difference** between high and low intensity for 5 comparisons (CBCL total score; internalising problems score and cut-off, externalising problems score and cut-off); **higher proportion in the low intensity group above cut-off for total CBCL score** (0.00 [SD 0.00] vs 0.07 [0.26], p<0.01 adjusted for multiple testing).<br>At 36 months, **no difference** between high and low intensity for 6 comparisons (CBCL total score and cut-off; internalising problems score and cut-off, externalising problems score and cut-off)<br>**No difference** in scores between high and low intensity programmes for 8 comparisons at 24 or 36 months: sleep problems, other problems, emotionally reactive, attention problems, anxious/depressed, somatic complaints, aggressive, withdrawn (p values adjusted for multiple testing).
Johnston 2006 (Long term whilst intervention is ongoing)	Subscales of Child Behaviour Checklist	**Higher mean score** in HS+PP group than HS only group for anxiety or depression at 36 months (aMD 0.40; 95% CI 0.07, 0.73; p<0.05).<br>**No difference** in aggressive behaviour between HS+PP group and HS only group.

For peer review only

Landry 2006<br>(Short term post-intervention)	Coding of videotaped interactions between child and mother, and child with examiner	**No difference** in mean scores for cooperation with mothers (p=0.08) or a novel adults (results not presented).<br>Infants in PALS demonstrated "**significantly greater increase in their use of words**" when interacting with mothers (p=0.02) and a novel adult (p=0.02).<br>**No effect** on affect in interactions with mothers overall (results not presented); **lower negative affect** seen in infants in the PALS group when interacting with a novel adult (results not presented).<br>Also present some results for the interaction with birthweight but presentation of results incomplete (text suggests more positive effects of the PALS intervention in very low birthweight group)

For peer review only

Table C5: Overall Development Outcomes

Overall development outcomes		
**Studies comparing one intervention with usual care**		
**Study**	**Measure**	**Results**
Chang 2015 (Short term post intervention)	Griffith Mental Development Scale	**No difference in mean developmental quotient (Griffith)** between intervention and control (mean 96.14, SD 9.40 compared with mean 94.68, SD 8.25); adjusted mean difference 1.10, 95% CI -0.45, 2.65; p value for comparison = 0.72)
Griffith 2011 (Short term post-intervention)	Schedule of Growing Skills II (SGS II)	**No difference in mean scores** between intervention (mean 99.20, SD 18.27) and control (mean 102.38, SD 17.79) at around 3 months post-intervention (p=0.51)
Wiggins 2004 (Immediately post-intervention)	Question on mother's perception of whether her child's development was normal	**No difference** between "supportive home visiting" and control (risk ratio 0.88, 95% CI 0.39-1.99) or "community groups" and control (risk ratio 0.57, 95% CI 0.22-1.52) in mother's perception of whether her child's development was normal
Wiggins 2004 (Short term post-intervention)	Questions on mother's worries about different aspects of development	Comparison on general development not possible between SHV and control as no mothers expressed concerns in the SHV group.<br>**No difference** in the number of number of mothers expressing worries about general development between CGS and control (risk ratio 0.75, 95% CI 0.15, 1.92)<br>Mothers in the SHV group had **fewer mean number of worries** about their child's development than in the control group (MD -0.23, 95% CI -0.42 to -0.01)<br>**No difference in the mean number of worries** about their child's development between CGS and control (MD 0.13, 95% CI -0.10 to 0.36)

Overall development outcomes		
**Studies comparing one intervention with usual care**		
**Study**	**Measure**	**Results**
Doyle 2011 (Short term whilst intervention is ongoing)	Ages and Stages Questionnaire	**No difference in overall mean score** between high and low intensity programme (p value **not** adjusted for multiple testing) at 6 months
Doyle 2011 (Medium term whilst intervention is ongoing)	Ages and Stages Questionnaire	**No difference in overall mean score** between high and low intensity programme (p value **not** adjusted for multiple testing) at 12 months
Doyle 2011 (Long term whilst intervention is ongoing)	Ages and Stages Questionnaire (measured at 18, 24 and 36 months)	**No difference in overall mean score** between high and low intensity programme (p value **not** adjusted for multiple testing) at any of the time points

For peer review only - http://bmjopen.bmj.com/site/about/guidelines.xhtml

D. Effect direction plot, stratified by other characteristics

D1: Results stratified by age of children at intervention delivery

Study<br>Intervention intensity	Study design	Risk of bias	Adherence	Outcome measurement		Development outcomes					Additional detail on intervention effects or sub-group analyses
				Timing	When?	Motor	Lang	Cogn	SEWB	Overall	
**Studies comparing one intervention with usual care**											
**Intervention during antenatal and/or first year postpartum only**											
**Beeghly 1995**<br>2 low intensity	iRCT	Unclear	Not reported	Post	Short	○		○			Tested for interaction between intervention and parity, IUGR, "demographic" risk and maternal psychological risk; no significant interactions found.
**Cheng 2007**<br>Moderate	iRCT	Low	Not reported	Post<br>Post	Short<br>Long				 ◦		For the short-term analysis, only sub-group analyses (by attachment quality) presented; results inconsistent.
**Cupples 2011**<br>High	iRCT	Low	Mean number of contacts = 8.5 (of 22 planned)	Post	Immed	○		○	○		
**Feinberg 2008**<br>Moderate	iRCT	Unclear	80% attended at least 3 of 4 antenatal sessions<br>60% attended at least 3 of 4 postnatal sessions	Post<br>Post<br>Post	Short<br>Medium<br>Long				○<br>○<br>○		3 of 5 comparisons in short term and 1 of 2 comparisons in medium term showed improved SEWB outcomes in intervention group.<br>Results presented in text suggest there may be interaction effects with gender, but there is incomplete reporting of the sub-group analyses.
**Lobo 2012**<br>2 moderate intensity	iRCT	Unclear	Excluded individuals who did not perform intervention on at least 60% of expected days	During<br>Post	Short<br>Short	◦<br>●					4 of 8 comparisons showed improved motor outcomes in the intervention arm in the short-term whilst intervention was ongoing.<br>5 of 5 comparisons showed improved motor outcomes in the intervention arm post-intervention.
**Santelices 2011**<br>Moderate	iRCT	Unclear	Not reported	Post	Short				◦		
**Wiggins 2004**<br>Moderate (SHV)	iRCT	Low	Mean number of visits = 7 (of 12 planned)	Post<br>Post	Immed<br>Short		 ●		 ○	○<br>●	1 of 1 comparison showed improved language outcome and 1 of 1 comparison showed improved overall development in intervention group.
**Wiggins 2004**<br>Low (CGS)	iRCT	Low	19% of women attended a group	Post<br>Post	Immed<br>Short		 ○		 ○	○<br>○	
**Intervention during first and second years postpartum**											
**Chang 2015**<br>Low	cRCT	Unclear	83% of mothers attended all visits	Post	Short	○	○	○		○	Improved cognitive outcome in intervention group on adjusting for potential confounders.
**Christakis 2007**<br>Low	iRCT	Unclear	Not reported	Post	Short		○		○		Sub-group results: 2 of 3 comparisons in low income group showed improved SEWB outcomes in intervention group; test for interaction not presented
**Doyle 2011**<br>1 high & 1 moderate	iRCT	Unclear	High: Mean number of visits = 46<br>Moderate: Not reported	During<br>During<br>During	Short<br>Medium<br>Long	○<br>○<br>○	○<br>○<br>○	○<br>○<br>○	○<br>○<br>○	○<br>○<br>○	3 of 12 comparisons showed improved cognitive outcomes, and 1 of 62 comparisons showed improved SEWB outcomes in the intervention arm, in the long-term whilst intervention was ongoing.

For peer review only

**Drotar 2008**<br>High	iRCT	Unclear	Not reported	During<br>During<br>During	Short<br>Medium<br>Long		○ (Long)	○ (Medium)<br>○ (Long)	○ (Short)<br>○ (Medium)<br>○ (Long)		1 of 12 comparisons showed improved SEWB outcomes in the intervention arm in the long-term. Incomplete reporting of the sub-group analyses.
**Griffith 2011**<br>High	iRCT	Low	60% attended 8 or more sessions (of 12 planned)	Post	Short				○	○	
**High 2000**<br>Low	iRCT	High	Mean number of visits = 3.4 (of 5 planned)	Post	Short		○				3 of 6 comparisons showed improved language outcomes in intervention group.<br>Sub-group results: no differences seen in 13-17 month olds; 6 of 6 comparisons in 18-25 month olds showed improved language outcomes in intervention group; no test for interaction presented.
**Hiscock 2008**<br>Low	cRCT	Low	49% of parents attended all sessions	Post<br>Post<br>Post	Short<br>Medium<br>Long				○<br>○<br>○		
**Johnston 2006**<br>2 high intensity	iRCT	Unclear	Not reported	During	Long		○		○		2 of 4 comparisons showed improved language outcomes, and 1 of 3 comparisons showed poorer SEWB outcomes, in the intervention arm in the long-term whilst intervention was ongoing.
**Landry 2006**<br>2 high intensity	iRCT	Unclear	91% of parents completed all 10 visits plus 2 assessment visits	Post	Short		●	●	○		2 of 2 comparisons showed improved language outcomes, and 1 of 1 comparison showed improved cognatic outcome in the intervention arm.<br>3 of 6 comparisons showed improved SEWB outcomes in the intervention arm.<br>Reporting of interaction with birthweight incomplete.
**Miller 2015**<br>High	iRCT	Low	Adherence data currently being analysed	During	Long			○	○		
**Minkovitz 2003**<br>High	iRCT	Unclear	79% of parents received 4 or more services (of 16)	During	Long				○		
**Niccols 2008**<br>Moderate	iRCT	Unclear	58% attended 4 or more sessions (of 8 planned)	Post<br>Post	Immed<br>Short				○<br>○		
**Niccols 2009**<br>Moderate	iRCT	Unclear	Not reported	Post<br>Post	Immed<br>Short				○<br>●		2 of 4 comparisons immediately post-intervention term showed improved SEWB outcomes in intervention group.<br>3 of 4 comparisons showed improved SEWB outcomes in intervention group in short-term.
**Tsiantis 2000**<br>Moderate	cRCT	Unclear	Not reported	Post<br>Post<br>Post	Short<br>Medium<br>Long		ø (Long)		○<br>○<br>○		2 of 2 comparisons showed poorer language outcomes in the intervention arm in the long-term.<br>1 of 2 comparisons showed poorer SEWB outcomes in the intervention arm in the medium-term.<br>1 of 8 comparisons showed improved SEWB outcomes in the intervention arm in the long-term.<br>Incomplete reporting of the sub-group analyses.
**Wagner 2001**<br>High	iRCT	Unclear	44% of families still receiving services at 2 yrs	During	Long	○	○	○	○		Results also stratified by income; no significant interactions reported.

For peer review only

D2: Results stratified by whether intervention delivery was delivered to all families or geographically targeted

Study<br>Intervention intensity	Study design	Risk of bias	Adherence	Outcome measurement		Development outcomes					Additional detail on intervention effects or sub-group analyses
				Timing	When?	Motor	Lang	Cogn	SEWB	Overall	
**Studies comparing one intervention with usual care**											
**Delivered to all families**											
**Beeghly 1995**<br>2 low intensity	iRCT	Unclear	Not reported	Post	Short	○		○			Tested for interaction between intervention and parity, IUGR, “demographic” risk and maternal psychological risk; no significant interactions found.
**Cheng 2007**<br>Moderate	iRCT	Low	Not reported	Post	Short						For the short-term analysis, only sub-group analyses (by attachment quality) presented; results inconsistent.
				Post	Long				○		
**Christakis 2007**<br>Low	iRCT	Unclear	Not reported	Post	Short		○		○		Sub-group results: 2 of 3 comparisons in low income group showed improved SEWB outcomes in intervention group; test for interaction not presented
**Drotar 2008**<br>High	iRCT	Unclear	Not reported	During	Short				○		1 of 12 comparisons showed improved SEWB outcomes in the intervention arm in the long-term. Incomplete reporting of the sub-group analyses.
				During	Medium			○	○		
				During	Long		○	○	○		
**Feinberg 2008**<br>Moderate	iRCT	Unclear	80% attended at least 3 of 4 antenatal sessions 60% attended at least 3 of 4 postnatal sessions	Post	Short				○		3 of 5 comparisons in short term and 1 of 2 comparisons in medium term showed improved SEWB outcomes in intervention group. Results presented in text suggest there may be interaction effects with gender, but there is incomplete reporting of the sub-group analyses.
				Post	Medium				○		
				Post	Long				○		
**Hiscock 2008**<br>Low	cRCT	Low	49% of parents attended all sessions	Post	Short				○		
				Post	Medium				○		
				Post	Long				○		
**Johnston 2006**<br>2 high intensity	iRCT	Unclear	Not reported	During	Long		○		○		2 of 4 comparisons showed improved language outcomes, and 1 of 3 comparisons showed poorer SEWB outcomes, in the intervention arm in the long-term whilst intervention was ongoing.
**Lobo 2012**<br>2 moderate intensity	iRCT	Unclear	Excluded individuals who did not perform intervention on at least 60% of expected days	During	Short	○					4 of 8 comparisons showed improved motor outcomes in the intervention arm in the short-term whilst intervention was ongoing. 5 of 5 comparisons showed improved motor outcomes in the intervention arm post-intervention.
				Post	Short	●					
**Miller 2015**<br>High	iRCT	Low	Adherence data currently being analysed	During	Long			○	○		
**Minkovitz 2003**<br>High	iRCT	Unclear	79% of parents received 4 or more services (of 16)	During	Long				○		
**Niccols 2008**	iRCT	Unclear	58% attended 4 or more	Post	Immed				○		

For peer review only

Moderate			sessions (of 8 planned)	Post	Short				○		
**Niccols 2009** Moderate	iRCT	Unclear	Not reported	Post	Immed				○		2 of 4 comparisons immediately post-intervention term showed improved SEWB outcomes in intervention group. 3 of 4 comparisons showed improved SEWB outcomes in intervention group in short-term.
				Post	Short				●		
**Santelices 2011** Moderate	iRCT	Unclear	Not reported	Post	Short				○		
**Tsiantis 2000** Moderate	cRCT	Unclear	Not reported	Post	Short				○		2 of 2 comparisons showed poorer language outcomes in the intervention arm in the long-term. 1 of 2 comparisons showed poorer SEWB outcomes in the intervention arm in the medium-term. 1 of 8 comparisons showed improved SEWB outcomes in the intervention arm in the long-term. Incomplete reporting of the sub-group analyses.
				Post	Medium				○		
				Post	Long		ø		○		
**Geographically targeted**											
**Chang 2015** Low	cRCT	Unclear	83% of mothers attended all visits	Post	Short	○	○	○		○	Improved cognitive outcome in intervention group on adjusting for potential confounders.
**Cupples 2011** High	iRCT	Low	Mean number of contacts = 8.5 (of 22 planned)	Post	Immed	○		○	○		
**Doyle 2011** 1 high & 1 moderate	iRCT	Unclear	High: Mean number of visits = 46<br>Moderate: Not reported	During	Short	○	○	○	○	○	3 of 12 comparisons showed improved cognitive outcomes, and 1 of 62 comparisons showed improved SEWB outcomes in the intervention arm, in the long-term whilst intervention was ongoing.
				During	Medium	○	○	○	○	○	
				During	Long	○	○	○	○	○	
**Griffith 2011** High	iRCT	Low	60% attended 8 or more sessions (of 12 planned)	Post	Short				○	○	
**High 2000** Low	iRCT	High	Mean number of visits = 3.4 (of 5 planned)	Post	Short		○				3 of 6 comparisons showed improved language outcomes in intervention group. Sub-group results: no differences seen in 13-17 month olds; 6 of 6 comparisons in 18-25 month olds showed improved language outcomes in intervention group; no test for interaction presented.
**Landry 2006** 2 high intensity	iRCT	Unclear	91% of parents completed all 10 visits plus 2 assessment visits	Post	Short		●	●	○		2 of 2 comparisons showed improved language outcomes, and 1 of 1 comparison showed improved cognatic outcome in the intervention arm. 3 of 6 comparisons showed improved SEWB outcomes in the intervention arm. Interaction with birthweight examined, but reporting of results incomplete.
**Wagner 2001** High	iRCT	Unclear	44% of families still receiving services at 2 yrs	During	Long	○	○	○	○		Results also stratified by income; no significant interactions reported.
**Wiggins 2004** Moderate (SHV)	iRCT	Low	Mean number of visits = 7 (of 12 planned)	Post	Immed					○	1 of 1 comparison showed improved language outcome and 1 of 1 comparison showed improved overall development in intervention group.
				Post	Short		●		○	●	
**Wiggins 2004** Low (CGS)	iRCT	Low	19% of women attended a group	Post	Immed					○	
				Post	Short		○		○	○	

D3: Results stratified by socio-economic status of the families included

Not enough information in a majority of papers to perform this comparison adequately.

For peer review only

PRISMA 2009 Checklist

Section/topic	#	Checklist item	Reported on page #
**TITLE**			
Title	1	Identify the report as a systematic review, meta-analysis, or both.	6
**ABSTRACT**			
Structured summary	2	Provide a structured summary including, as applicable: background; objectives; data sources; study eligibility criteria, participants, and interventions; study appraisal and synthesis methods; results; limitations; conclusions and implications of key findings; systematic review registration number.	2-3
**INTRODUCTION**			
Rationale	3	Describe the rationale for the review in the context of what is already known.	5-6
Objectives	4	Provide an explicit statement of questions being addressed with reference to participants, interventions, comparisons, outcomes, and study design (PICOS).	6
**METHODS**			
Protocol and registration	5	Indicate if a review protocol exists, if and where it can be accessed (e.g., Web address), and, if available, provide registration information including registration number.	6 and protocol uploaded
Eligibility criteria	6	Specify study characteristics (e.g., PICOS, length of follow-up) and report characteristics (e.g., years considered, language, publication status) used as criteria for eligibility, giving rationale.	6-7
Information sources	7	Describe all information sources (e.g., databases with dates of coverage, contact with study authors to identify additional studies) in the search and date last searched.	8
Search	8	Present full electronic search strategy for at least one database, including any limits used, such that it could be repeated.	Web appendix 1
Study selection	9	State the process for selecting studies (i.e., screening, eligibility, included in systematic review, and, if applicable, included in the meta-analysis).	8
Data collection process	10	Describe method of data extraction from reports (e.g., piloted forms, independently, in duplicate) and any processes for obtaining and confirming data from investigators.	8
Data items	11	List and define all variables for which data were sought (e.g., PICOS, funding sources) and any assumptions and simplifications made.	8
Risk of bias in individual studies	12	Describe methods used for assessing risk of bias of individual studies (including specification of whether this was done at the study or outcome level), and how this information is to be used in any data synthesis.	8

For peer review only

PRISMA 2009 Checklist

Section/topic	#	Checklist item	Reported on page #
Summary measures	13	State the principal summary measures (e.g., risk ratio, difference in means).	N/A (narrative synthesis)
Synthesis of results	14	Describe the methods of handling data and combining results of studies, if done, including measures of consistency (e.g., I^2) for each meta-analysis.	N/A (narrative synthesis)
Risk of bias across studies	15	Specify any assessment of risk of bias that may affect the cumulative evidence (e.g., publication bias, selective reporting within studies).	8
Additional analyses	16	Describe methods of additional analyses (e.g., sensitivity or subgroup analyses, meta-regression), if done, indicating which were pre-specified.	9
**RESULTS**			
Study selection	17	Give numbers of studies screened, assessed for eligibility, and included in the review, with reasons for exclusions at each stage, ideally with a flow diagram.	9 and Figure 1
Study characteristics	18	For each study, present characteristics for which data were extracted (e.g., study size, PICOS, follow-up period) and provide the citations.	9 and Table 1
Risk of bias within studies	19	Present data on risk of bias of each study and, if available, any outcome level assessment (see item 12).	Figures 2 and 3
Results of individual studies	20	For all outcomes considered (benefits or harms), present, for each study: (a) simple summary data for each intervention group (b) effect estimates and confidence intervals, ideally with a forest plot.	12-13 (narrative synthesis)
Synthesis of results	21	Present results of each meta-analysis done, including confidence intervals and measures of consistency.	N/A (narrative synthesis)
Risk of bias across studies	22	Present results of any assessment of risk of bias across studies (see Item 15).	12-13 and Table 3
Additional analysis	23	Give results of additional analyses, if done (e.g., sensitivity or subgroup analyses, meta-regression [see Item 16]).	13 and Web Appendix D

For peer review only

PRISMA 2009 Checklist

**DISCUSSION**			
Summary of evidence	24	Summarize the main findings including the strength of evidence for each main outcome; consider their relevance to key groups (e.g., healthcare providers, users, and policy makers).	14
Limitations	25	Discuss limitations at study and outcome level (e.g., risk of bias), and at review-level (e.g., incomplete retrieval of identified research, reporting bias).	15
Conclusions	26	Provide a general interpretation of the results in the context of other evidence, and implications for future research.	16
**FUNDING**			
Funding	27	Describe sources of funding for the systematic review and other support (e.g., supply of data); role of funders for the systematic review.	17

From: Moher D, Liberati A, Tetzlaff J, Altman DG, The PRISMA Group (2009). Preferred Reporting Items for Systematic Reviews and Meta-Analyses: The PRISMA Statement. PLoS Med 6(7): e1000097. doi:10.1371/journal.pmed1000097

For more information, visit: **www.prisma-statement.org**.

Page 2 of 2

For peer review only
